# Proceedings of the 57^th^ Annual Meeting of the Particle Therapy Cooperative Group (PTCOG): 21-26 May 2018, *Hosted by Cincinnati Children's and the University of Cincinnati, part of the Cincinnati Cancer Center, Cincinnati, Ohio, USA*

**DOI:** 10.14338/2331-5180-5-2-000

**Published:** 2018-11-30

**Table:** 

Table of Contents	
Program Description	pg. 58
2018 Committees	pg. 59
Oral Presentations	pg. 59
Poster Abstracts	pg. 125

## Program Description

### Overview

The main theme of this year's annual meeting is **Translating Science and Technology into Cancer Cures.**

Particle Therapy has always been a multi-disciplinary collaboration. It may be one of the shining examples of how science and technology have been seamlessly fused and applied to medical therapy. Accessibility to Particle Therapy continues to be in a strong growth phase and the modality has shifted from work in national science laboratories to multi- and single-room treatment facilities in hospitals and outpatient clinics. Additionally, the focus is shifting from the application of technology to the application of life sciences, such as biology and concurrent medical oncology therapies, to benefit even more patients.

PTCOG 57 featured plenary talks from internationally nationally recognized experts, more than 50 abstract presentations, hundreds of scientific posters, receptions and networking opportunities among colleagues, exhibits of latest technologies from vendors around the world, and a site visit to the new Cincinnati Children's Hospital/University of Cincinnati Medical Center Proton Therapy Center.

### Target Audience

- Physicians, Radiation Oncologists, Oncologists, Surgeons, Residents, and Fellows
- Medical Physicists, Dosimetrists, and Radiation Therapists
- Radiation Biologists, Accelerator Engineers, and Scientists
- Registered Nurses and Clinical Researchers
- Health Care Policy Makers, Insurance Executives, Industry Personnel, and Hospital Administrators

## Particle Therapy Cooperative Group (PTCOG) Steering Committee

**Jay Flanz, Chair**

Project and Technical Director at the Harvard Massachusetts General Hospital's (“MGH”) Francis H. Burr Proton Therapy Center 

**Tadashi Kamada, Vice-Chair**

Director General of Clinical Research Cluster, National Institute of Radiological Sciences, QST 

**Marco Durante, Vice-Chair**

Director, Trento Institute for Fundamental Physics and Applications (TIFPA), National Institute of Nuclear Physics (INFN) 

**Martin Jermann, Secretary/Treasurer**

Consultant, Directorate Staff, Paul Scherrer Institut (PSI)

## Particle Therapy Cooperative Group 2018 (PTCOG 57) Conference Committee

**John Perentesis, MD, FAAP, Co-Chair**

Directo, Division of Oncology, Cincinnati Children's Hospital Medical Center

**Anthony Mascia, PhD, DABR, Co-Chair**

*Adjunct Assistant Professor, UC Medical Center Proton Therapy Center, Physician, UC Health*.

**John Breneman, MD, Co-Chair**

*Professor of Radiation Oncology and Neurosurgery, UC College of Medicine, Physician, UC Health*.

## Oral Presentations

### O 001: New Range Margin Recommendations Based on Dose Calculation Uncertainties

#### J. Schuemann^1^, H. Paganetti^1^

##### ^1^Massachusetts General Hospital & Harvard Medical School, Rad. Onc., Boston, USA

The distal falloff of proton beams offers sharp dose contrasts to form highly conformal dose distributions. However, due to range uncertainties, margins are applied to ensure target coverage, resulting in unnecessarily irradiated tissue and avoidance of certain beam configurations. One of the main contributions determining range margins is uncertainty from analytical dose calculation algorithms (ADCs). Paganetti (2012) estimated that range margins (neglecting setup uncertainties) should be between 2.7% and 4.6% when using standard clinically used ADCs, depending on the heterogeneity of the patient geometry. Schuemann et al (2014) came to a similar conclusion, estimating that range margins could be reduced for some sites to 2.8% while they should be as high as 6.3% for others; however, that analysis did not focus on target coverage.

We analyzed 1920 treatment fields to provide new recommendations for range margins. Six treatment sites (liver, prostate, breast, head-and-neck, lung and medullo) were sub-divided into 12 categories. For each category, the range margins were estimated for target coverage, assuming a perfect match of dose and range of delivered and ADC fields in water phantoms.

We found that range margins can be reduced to 2.5% for prostate, pure liver and brain treatments. However, when not correcting for loss of dose due to small field sizes, scattering, etc., 3.5% should be used. Prostate fields could also use a constant range margin of 2.5 mm. For heterogeneous geometries, (see table and figure) generic range margins of 5.3% may be necessary.

### O 002: Shortening Delivery Times for PBS Proton Therapy by Reducing the Number of Proton Spots without Compromising Dosimetric Plan Quality

#### M.F. Belosi^1^, S. van de Water^2^, F. Albertini^1^, D.C. Weber^3^, A.J. Lomax^4^

##### *^1^Paul Scherrer Institute, Centre for Proton Therapy, Villigen PSI, Switzerland*, *^2^Unaffiliated, Netherlands*, *^3^Paul Scherrer Institute- University Hospital of Bern and Zurich, Centre for Proton Therapy, Villigen PSI, Switzerland*, *^4^Paul Scherrer Institute- Swiss Institute of Technology ETH, Centre for Proton Therapy, Villigen PSI, Switzerland*

**Purpose/objective:** To investigate, through planning and experimental validation, the quality, deliverability, accuracy, robustness and delivery time reduction of spot-reduced PBS treatment planning.

**Material/methods**: For a head-and-neck patient, conventional and ‘spot-reduced' SFUD plans were generated, with spot-reduced plans being calculated using the ‘pencil beam resampling' technique of Erasmus-iCycle (Erasmus MC Cancer Institute). This involves repeated inverse optimization while iteratively excluding low-weighted proton spots until the plan quality deteriorates. Beam setup was identical for both plans and the resulting dosimetric plan quality was comparable. Both plans were delivered on PBS Gantry 2 at PSI, measuring the delivery time per field and dose profiles in water, and subsequently recalculating dose distributions using the machine log-files. In addition, robustness analysis was performed to assess sensitivity to delivery inaccuracies and errors in patient setup and proton range.

**Results:** The total number of spots for the plan could be reduced by 94% (26069 to 1540), resulting in an average delivery time reduction of 65% per field (Table 1). Measured dose profiles differed from the planned dose by 2.9%-4.3% for the spot-reduced plan and by <2% for the conventional plan. For both plans, the log-file dose reconstruction was within ±1% of the planned dose for all voxels (Figure 1). Spot-reduced plans were slightly more sensitive to delivery inaccuracies, requiring a spot position accuracy within ≤0.5mm, but were surprisingly less sensitive to setup and range errors.

**Conclusion:** Delivery times per field could be reduced by 65% using spot reduction without substantially compromising plan quality, delivery accuracy or robustness.

### O 003: Limitations of Worst Case Robust Optimization: A Comparison with an All-scenario Approach

#### J. Ma^1^, H.S. Wan Chan Tseung^1^, M. Herman^1^, C. Beltran^1^

##### ^1^Mayo Clinic, Department of Radiation Oncology, Rochester, USA

**Purpose:** Worst case optimization has been widely employed in intensity modulated proton therapy (IMPT) to achieve robust optimization. This work evaluates some of the limitations of worst case robust optimization in IMPT, and proposes an all-scenario robust optimization as an alternative to mitigate some of the limitations.

**Method:** Worst case robust optimizations focus on a single scenario at each optimization iteration. The all-scenario approach strives to satisfy constraints under all uncertainty scenarios at each optimization iteration by including dose from all scenarios in the objective function rather than the worst case scenario only. Two different worst case robust optimizations were studied: composite worst case optimization and voxel-wise worst case optimization. The worst case approaches were compared with the all-scenario approach in two different clinical cases: a head and neck case and a brain case. The different optimization approaches were implemented with the same gradient based optimization engine. Plan quality and optimization convergence were compared between approaches. The optimization engine was GPU-accelerated. The dose calculation was based on an in-house GPU-accelerated Monte Carlo.

**Results:** Compared with composite and voxel-wise worst case optimization, the all-scenario robust optimization converged faster, and arrived at solutions with tighter DVH robustness spread, better target coverage and lower OAR dose.

**Conclusion:** The limitations of worst case robust optimization methods should be carefully evaluated for clinical use. The all-scenario robust optimization method has been shown to be a better choice.

### O 004: L1-Spot: Iteratively Constrained Spot Optimization with L1-Sparsity Regularization for Generating Efficient Proton PBS Plans

#### Y. Lin^1^, H. Gao^1^, B. Clasie^2^, F.F. Yin^1^

##### *^1^Duke University Medical Center, Radiation Oncology, Durham, USA*, *^2^Massachusetts General Hospital and Harvard Medical School, Radiation Oncology, Boston, USA*

The initial proton pencil beam scanning (PBS) treatment plans often consist of spots with weights that are too small to be deliverable and thus require post-processing to eliminate spots that fall below the deliverable threshold set by the treatment machine (indicated as Gmin).

The purpose of this work is to develop an efficient spot optimization algorithm to generate PBS plans that eliminate low-weight spots while preserving the original intended dose distribution and also with potentially enhanced delivery efficiency by minimizing the number of spots to be delivered.

The proposed method, namely L1-Spot, is a type of inverse planning algorithm that formulates the problem as a constrained optimization problem with L1 sparsity regularization. The minimum spot constraints are iteratively updated and enforced during the spot optimization, and the solution algorithm is based on alternating direction method of multipliers.

The L1-Spot was compared against two state-of-art spot post-processing methods, round and redistribution (Figure1). The round method rounds spots between Gmin/2 and Gmin up to Gmin, and deletes spots below Gmin/2. The redistribution method interactively redistributes the lowest weight spot to its nearest neighbors until all spots comply with the machine limit (>Gmin).

Its performance was evaluated using plans that use three different spot sizes (8mm, 4.6mm, and 2.3mm), see Table1. The evaluation criteria were the γ-index pass rate that compares the original and processed dose distributions.

We developed a L1-Spot method to generate an efficient deliverable plan for PBS treatment and demonstrated that it was more effective than redistribution at large Gmin and/or small spot size.

### O 005: FROG: A New Calculation Engine for Physical and Biological Dose Investigations at CNAO

#### K. Choi^1,2^, S. Mein^3,4^, B. Kopp^4,5^, G. Magro^1^, S. Molinelli^1^, M. Ciocca^1^, A. Mairani^1,6^

##### *^1^CNAO, Medical Physics, Pavia, Italy*, *^2^Pavia University, Physics, Pavia, Italy*, *^3^Heidelberg University, Physics, Heidelberg, Germany*, *^4^DKFZ, Imaging and Radiation Oncology, Heidelberg, Germany*, *^5^Heidelberg University Clinic, Radiation Oncology, Heidelberg, Germany*, *^6^HIT, Medical Physics, Heidelberg, Germany*

Fast and accurate dose calculation engine for hadrontherapy is important for both research and clinical application. FROG is a GPU-based forward calculation tool developed at CNAO (Centro Nazionale Adroterapia Oncologica) and HIT (Heidelberg Ion Beam Therapy Center) for fast and accurate calculation of both physical and biological dose for proton and carbon ion scanning beams. FROG calculation engine adopts a triple Gaussian parameterization for the description of the lateral dose distributions for a better agreement against FLUKA predictions. FROG provides dose/biological dose 2D maps, profiles and dose-volume-histograms. FROG-based RTDOSE DICOM files are created which can be read in by commercial treatment planning systems. For the benchmark of the FROG calculation engine, 6 Spread Out Bragg Peaks and few patients CNAO carbon treatment cases have been chosen and re-calculated with FROG. As a result, biological dose agreement against FLUKA is approximately within about 2%, while physical dose agreement varies with the beam energy from about 1% up to 3%. Both MKM (Microdosimetric Kinetic Model) and LEM (Local Effect Model) biological dose are implemented and tested in FROG to support the NIRS (National Institute of Radiological Sciences)-based to LEM-based biological dose conversion. FROG calculation engine is accurate and fast enough to re-calculate physical and biological doses applying various biological models.

### O 008: Uncertainties in Proton Stopping Power Ratio (SPR) from a Novel CT/MRI Tissue Classification Model

#### A. Witztum^1^, A. Sudhyadhom^1^, T. Solberg^1^

##### ^1^University of California- San Francisco, Department of Radiation Oncology, San Francisco, USA

**Purpose:** To analyze the uncertainty in SPR calculations based on errors in mean ionization potential (Im) and tissue classification in a four-component tissue classification (4CC) model using CT/MRI imaging.

**Methods:** We proposed a 4CC system to classify molecules in the human body as proportions of water, fat, and protein (by MRI) and hydroxyapatite (HA) by CT so that:

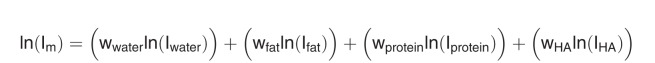
where 'w' is mass content percentage and 'I' is mean ionization potential for each type of molecule. Reference values were obtained for soft tissues (tissue) and cortical bone (bone) from ICRU report 44 and others in the literature.

Errors in mass content percentages were normally distributed between 0-2% (water, HA), and 0-10% (fat). Errors in I were uniformly distributed between +/- 10 eV (water, fat, protein) and +/- 16 eV (HA).

Proton (E = 250MeV) SPR was calculated using Bragg Additivity Rule (BAR) Im as the reference standard and compared to SPR using Im from: the 4CC system without errors SPR (4CC), and mean SPR from the distribution with errors.

**Results:** Table 1 shows that for tissue and bone, the 4CC model SPR is within 0.2% of BAR. Adding the maximum systematic (0.2%) and random (2.4%) errors in quadrature yield a total maximum error of 2.4%.

**Conclusion:** Current SPR calculation methods do not account for uncertainty in I. Even with a conservative error distribution the 4CC method performs well compared to current techniques. Content quantification by CT/MRI using the 4CC model has been demonstrated to be an accurate method for SPR determination.

### O 009: Comprehensive Log-file Based Monte Carlo Validations of a Fast, Analytical Dose Calculation for Pencil Beam Scanned Proton Therapy

#### C. Winterhalter^1^, Y. Tian^1^, G. Meier^1^, A. Bolsi1, M. Dieterle^1^, J. Hrbacek^1^, D. Oxley^1^, D.C. Weber^1^,^2^,^3^, S. Safai^1^, A. Lomax^1^

##### *^1^Paul Scherrer Institute, Center for Proton Therapy, Villigen PSI, Switzerland*, *^2^University Hospital of Zurich, Radiation Oncology Department, Zurich, Switzerland*, *^3^University Hospital of Bern, Radiation Oncology Department, Bern, Switzerland*

**Purpose:** To validate a fast ray casting dose calculation (RCDC) for pencil beam scanned (PBS) proton therapy using log-file based Monte Carlo (LF-MC) calculations.

**Materials:** PBS treatment planning at PSI uses a fast (0.5-5s/field using standard computer hardware) RCDC (Schaffner et al 1999). To validate the accuracy of this, clinical treatment plans have been reconstructed using log-file based Monte Carlo (TOPAS 3.0.p1) simulations.

**Results:** MC parameters have been defined such that beam widths in air and depth doses in water match measurements to within 0.1mm. For all simulations, proton numbers per pencil-beam are directly derived from Faraday cup based monitor unit calibration measurements. As such, MC calculated energy specific absolute doses in water were found to agree to within -2%+/-0.5% to measurements for a range of energies (figure 1). When applied to clinical skull base and lung cases, and after the LF-MC distribution was normalized to the mean PTV dose of the RCDC (LF-MC normalization factors), 99%/92% of dose voxels with dose >10% of the prescription dose agreed to within +/-5% between the LF-MC and RCDC distributions respectively (figure 2). Correcting for the -2% absolute dose offset, the LF-MC normalization factors agreed with those from field specific verifications to between 0.3-1.9% per field for the skull base and 1.8-3.0% for the lung case.

**Conclusion:** The fast RCDC algorithm provides excellent relative agreement to LF-MC reconstructed dose, even when including delivery uncertainties derived from log-files. Absolute dose prediction of the MC is good and matches well with patient specific absolute dose measurements.

### O 010: The Impact of LET/RBE Modelling and Robust Analysis on Skull-Base and Paediatric Craniopharyngioma Proton Plans, Benchmarked against VMAT

#### A. Gutierrez^1^, V. Rompokos^2^, K. Li1, C. Gillies^2^, D. D'Souza^2^, F. Solda^3^, N. Fersht^3^, C. Yen Ching^3^, G. Royle^1^, R. Amos^1^, T. Underwood^1^

##### *^1^University College London, Department of Medical Physics and Biomedical Engineering, London, United Kingdom*, *^2^University College London Hospitals NHS Foundation Trust, Department of Radiotherapy Physics, London, United Kingdom*, *^3^University College London Hospitals NHS Foundation Trust, Department of Clinical Oncology, London, United Kingdom*

**Purpose**: To investigate how physical and biological uncertainties shift the balance between VMAT and IMPT, considering whether IMPT plans remain dosimetrically superior when such uncertainties are considered.

**Methodology**: We retrospectively studied ten cases: four chondrosarcoma, two chordoma and four paediatric craniopharyngioma. VMAT and IMPT plans were created according to modality-specific protocols using a PTV for VMAT and robust optimisation for IMPT. For IMPT we considered (i) variable RBE modelling using the McNamara model for different values of α/β, and (ii) robust analysis with +/-3mm set-up and 3.5% range uncertainties.

**Results**: Comparing the VMAT and nominal IMPT plans, the dosimetric advantages of IMPT were clear: IMPT led to reduced integral dose (especially to the normal brain) and typically, improved CTV coverage given our OAR constraints. When robustness analysis was performed, IMPT doses exceeding the constraints were predicted for small volumes of OARs such as the brainstem and chiasm. However, variable RBE-weighted dose analyses predicted even more substantial hotspots. Within the nominal VMAT and IMPT plans, generally less than 0.1cm3 of the brainstem received the dose constraint, as shown in Figure 1. However, when the IMPT robustness analyses (worst case scenarios assuming RBE=1.1) and variable RBE modelling were performed, substantial increases to the volume receiving the constraint were observed.

**Conclusion**: Both physical and biological robustness analyses should be considered for IMPT: these analyses can substantially affect the sparing of OARs and shift the dosimetric balance relative to VMAT.

### O 011: Impact of Radiotherapy Modality on Neuropsychological Outcomes of Pediatric Brain Tumor Patients

#### J. Gross^1^, S. Powell^2^, F. Zelko^2^, W. Hartsell^3^, S. Goldman^4^, J. Fangusaro^4^, R. Lulla^4^, N. Pillay Smiley^4^, J. Chang^3^, V. Gondi^3^

##### *^1^Northwestern University, Radiation Oncology, Chicago, USA*, *^2^Ann and Robert H. Lurie Children's Hospital of Chicago, Psychology, Chicago, USA*, *^3^Northwestern Medicine Chicago Proton Center, Radiation Oncology, Warrenville, USA*, *^4^Ann and Robert H. Lurie Children's Hospital of Chicago, Pediatric Oncology, Chicago, USA*

**Purpose**: To evaluate predictors of neuropsychological outcomes for pediatric brain tumor patients undergoing X-ray radiotherapy (XRT) or proton radiotherapy (PRT).

**Methods**: 125 patients received treatment for brain tumors. All received age-appropriate neuropsychological assessment of intelligence quotient (IQ), processing speed (PS), visual motor integration (VMI), executive function, memory and parent-reported function at our institution.

**Results**: Median age at diagnosis was 7.0 years; median time from treatment to last assessment was 4.0 years. Patients receiving PRT had higher socioeconomic status (SES), differing distributions of race and tumor locations (Table 1), as well shorter median follow-up time compared to XRT (9.5 vs. 5.0 years, p<0.001). Univariate and multivariate analyses with tests for interaction of treatment group and follow-up identified higher verbal IQ (β=0.84 points/year, p=0.02) and full-scale IQ (β=1.03, p=0.01) for older patients; lower PS (β=-15.9 points, p=0.04) and VMI (β=-14.0, p=0.006) following CSI; and higher full-scale IQ (β=10.6 points, p=0.048), PS (β=12.6, p=0.02), and parent-reported practical function (β=13.8, p=0.049) following PRT relative to XRT. VMI was higher in those with higher SES (β=1.2 points/$10,000 household income, p=0.04), but lower following receipt of vincristine chemotherapy (β=-16.6, p=0.01). Parent-reported practical function was lower in those with posterior fossa tumors (β=-10.8, p=0.048).

**Conclusions**: Neuropsychological outcomes of pediatric brain tumor patients are impacted by age, SES, receipt of CSI or vincristine chemotherapy, and tumor location. Relative to XRT, PRT is associated with favorable neuropsychological outcomes in terms of full-scale IQ, PS, and parent-reported practical function.

### O 012: Comparison of Symptomatic and Asymptomatic Imaging Changes following Pencil-Beam Scanning and Uniform Scanning Proton Therapy for Posterior Fossa Tumors

#### J. Gross^1^, B. Kreydick^2^, E. Chang^2^, M. Pankuch^2^, C. Bregman^3^, J. Kalapurakal^1^, J. Chang^2^, W. Hartsell^2^, V. Gondi^2^

##### *^1^Northwestern University, Radiation Oncology, Chicago, USA*, *^2^Northwestern Medicine Chicago Proton Center, Radiation Oncology, Warrenville, USA*, *^3^Ann and Robert H. Lurie Children's Hospital of Chicago, Radiology, Chicago, USA*

**Purpose**: Strategies to mitigate the RBE effects at the Bragg peak end-of-range have unknown impact on post-treatment imaging changes. We compared treatment-related imaging changes following proton therapy (PT) delivered with uniform scanning (US) without mitigation, US with mitigation (blocking, range feathering, range pushing), and pencil beam scanning (PBS).

**Methods**: MRI and follow-up for all patients with medulloblastoma and infratentorial ependymoma receiving PT between 2011 and 2016 at our institution were reviewed with a Neuroradiologist. New lesions on contrast-enhanced T1- or T2-weighted MRI following PT were recorded and graded as per the Common Terminology Criteria for Adverse Events (CTCAE) v4.0. Comparisons were made with the Kaplan-Meier method and multivariable Cox Proportional Hazards models.

**Results**: 127 patients met inclusion criteria (Table 1). Crude rates of asymptomatic imaging changes were 38.7% for US without mitigation, 48.2% for US with mitigation, and 51.3% for PBS. On time-to-event analysis, PBS was associated with higher rate of asymptomatic imaging changes in the posterior fossa (p<0.001, Figure 1). On multivariable analysis accounting for age, total dose and receipt of chemotherapy, PBS retained significant association with asymptomatic imaging changes in the posterior fossa (HR 10.1, 95% CI 2.7 - 38.4, p<0.001). Symptomatic changes associated with CTCAE grade 2+ events occurred in 5 patients (crude rate 3.9%). No difference in symptomatic imaging changes was observed between cohorts.

**Conclusions**: Following PT for medulloblastoma or infratentorial ependymoma, symptomatic imaging changes are rare and not impacted by PT modality, but PBS is associated with increased asymptomatic imaging changes in the posterior fossa.

### O 013: Factors Associated with Insurance-Related Referral Delays in Children Irradiated with Proton Therapy in the Pediatric Proton Consortium Registry

#### C. Hess^1^, D. Indelicato^2^, A. Paulino^3^, W. Hartsell^4^, C. Hill-Kayser^5^, S. Perkins^6^, A. Mahajan^7^, N. Laack^7^, R. Ermoian^8^, T. Yock^1^

##### *^1^Massachusetts General Hospital, Radiation Oncology, Boston, USA*, *^2^University of Florida, Radiation Oncology, Jacksonville, USA*, *^3^MD Anderson, Radiation Oncology, Houston, USA*, *^4^Northwestern Medicine, Chicago Proton Center, Chicago, USA*, *^5^University of Pennsylvania, Radiation Oncology, Philadelphia, USA*, *^6^Washington University, Radiation Oncology, St. Louis, USA*, *^7^Mayo Clinic, Radiation Oncology, Rochester, USA*, *^8^University of Washington, Radiation Oncology, Seattle, USA*

**Background/Objectives:** We report factors associated with insurance-associated treatment delays in children irradiated with proton therapy in the Pediatric Proton Consortium Registry.

**Design/Methods:** A multi-institutional registry of childhood cancer patients undergoing proton radiation therapy was opened to enrollment in summer 2012. Data was frozen for analysis on October 28, 2017.

**Results:** Of 1,578 children enrolled, 1,304 (83%) had available data regarding insurance-associated delays. A total of 83 of these children (6%) were delayed by insurance: 51/1029 (5%) for private/foreign insurance compared to 32/275 (12%) among Medicaid patients (p<0.001). On average, 28.3% of children with rare tumors were delayed compared to 8.8% with non-rare tumors (p<0.001). Among non-rare tumors, non-CNS tumors were more likely to be delayed than CNS (p=0.001). Neither age nor gender predicted delay. Black, Hispanic, and Asian children made up 6%, 8%, and 5% of the total cohort, 13%, 21%, and 2% of the Medicaid population, and 12%, 16%, and 1% of children with delay, respectively. “Black or Hispanic” children (8%) had higher rates of insurance-related delays compared to “white and non-Hispanic” children (5%) (p=0.001), yet Medicaid insurance status increased delays to 12% and 14% in both groups, respectively. Delays were rare among Asian children (2%).

**Conclusion:** Insurance-related RT delay for proton therapy referral is more common in Black or Hispanic children than white but Medicaid coverage doubles delays across racial/ethnic groups. Rare and non-CNS tumor types are risk factors for delay. Awareness of risk factors for referral delay can guide efforts to address disparities in access to advanced treatments.

### O 014: Craniospinal Irradiation: Evaluation of Proton Range Deviation Using Daily CBCT

#### W. Yao^1^, T. Merchant^1^

##### ^1^St. Jude Children's Research Hospital, Radiation Oncology, Memphis, USA

**Purpose**: Proton range deviation on the surface of the clinical target volume (CTV) provides important information about actual delivered dose. Cone-beam computed tomography (CBCT) accurately reflects patient anatomy relative to the treatment delivery system. CBCT may be used to calculate range deviation to set the appropriate set-up margin. This work focused on range deviation in the clinical setting of craniospinal irradiation (CSI).

**Method**: 396 CBCT data sets from 10 pediatric patients treated with intensity-modulated proton CSI were used to investigate range deviation. The water equivalent thickness along the CTV surface was calculated for each beam using the first day CBCT which was then used as the reference for the remaining treatments. Additional pCTs performed in 2 patients for boost planning were used to check the consistency of the calculated range deviations in CBCT and pCT.

**Results**: The mean range deviation (mean ± RMS) for the 10 study patients was 2.7 ± 1.5 mm in the cranium, 2.3 ± 1.3 mm in the cervical, 1.5 ± 0.7 mm in the upper (thoracic) spine and 1.9 ± 0.9 mm in the lower (lumbar) spine. In the pCTs the mean range deviation for the cranium was 2.4 ± 0.7 mm that underwent consistency evaluation.

**Conclusion**: Range deviation calculated using daily CBCT can be reliably used to monitor range difference in patients undergoing daily CSI. We estimate that an inter-fraction margin of 5 mm for the cranio-cervical, and 3 mm for the upper and lower spine volumes would be sufficient in the absence of personalized set-up margins.

### O 015: Practice Trends for Vertebral Body Coverage of Pediatric Patients Undergoing Proton Craniospinal Irradiation

#### S. Medek^1^, B. De^2^, J. Breneman^1,3^, L. Pater^1,3^, N. Laack^4^, A. Mahajan^4^, S. Wolden^2^, R. Vatner^1,3^

##### *^1^University of Cincinnati Medical Center, Radiation Oncology, Cincinnati, USA*, *^2^Memorial Sloan Kettering Cancer Center, Radiation Oncology, New York, USA*, *^3^Cincinnati Children's Hospital Medical Center, Radiation Oncology, Cincinnati, USA*, *^4^Mayo Clinic, Radiation Oncology, Rochester, USA*

**Introduction**: Craniospinal irradiation (CSI) is an important therapeutic component for pediatric brain tumors with a propensity for dissemination. While the thecal sac is the spinal clinical target volume (CTV), many physicians treat the entire vertebral body (VB) in growing children due to risk of lordosis from partial VB treatment. Currently there is no standard of care for determining VB coverage in proton based CSI, leading to variable practice patterns.

**Materials/Methods**: Pediatric radiation oncologists were identified from membership in the PT-COG pediatric subcommittee or affiliation with U.S. proton centers. Potential participants were contacted by email with link to an IRB approved, anonymized web-based survey distributed on June 21st 2017 with follow-up on October 10th 2017. The survey utilized skip logic and included up to 11 questions regarding practice patterns.

**Results**: Thirty-three radiation oncologists representing all regions of the U.S. responded. Five were excluded for lack of recent pediatric proton CSI experience. Of 28 responses, 23 physicians sometimes cover, five always cover and zero never cover the entire VB. Factors influencing VB coverage included patient age (n=17), bone age (n=12), growth curves (n=7) and parental height (n=6). Most physicians modify anterior CTV margins to protect ventral structures.

**Conclusion**: Vertebral body coverage varies amongst radiation oncologists treating pediatric patients with proton CSI, with multiple factors influencing physician technique. These data suggest the need for a standardized approach to VB coverage in pediatric proton based CSI.

### O 016: Proton versus Photon Radiation Therapy for Pediatric Head and Neck Rhabdomyosarcoma: Disease Control, Overall Survival, and Toxicity

#### D. Casey^1^, L. Wexler^2^, S. Wolden^1^

##### *^1^Memorial Sloan Kettering Cancer Center, Department of Radiation Oncology, New York, USA*, *^2^Memorial Sloan Kettering Cancer Center, Department of Pediatrics, New York, USA*

**Introduction**: Proton therapy (PT) provides promise in sparing late toxicity without compromising clinical outcomes for children with head and neck rhabdomyosarcoma (HNRMS). We compared outcomes after photon versus proton irradiation.

**Methods**: Sixty-six HNRMS pediatric patients (age <25 years) were treated with definitive chemoradiation at Memorial Sloan Kettering. Fifty-one patients were treated with intensity modulated radiation therapy (IMRT) and 15 patients with PT. Locoregional control (LRC), event-free survival (EFS), overall survival (OS), and acute toxicity were compared.

**Results**: Median follow-up was 9.8 versus 1.6 years in the IMRT and PT cohorts, respectively. The cohorts were similar with respect to age, gender, race, tumor site, size, group, and stage. Patients treated with IMRT were more likely to be PAX/FOXO1 fusion-positive (p=0.06) and node-positive (p=0.03). Five-year LRC was 91% in the IMRT cohort versus 85% in the PT cohort (p=0.28). All local failures were in-field; there were no marginal failures. Five-year EFS (71% vs 74%, p=0.97) and OS (75% vs 71%, p=0.54) were similar for both cohorts. There were no grade 4 acute toxicities. Grade 3 mucositis rates were slightly higher in the IMRT cohort (20% versus 13%, p=0.58), while grade 3 dermatitis rates were higher in the PT cohort (33% versus 2%, p<0.0001).

**Conclusions**: IMRT and PT appear to result in similar LRC and survival for pediatric HNRMS. Longer follow-up is needed in the proton cohort prior to fully establishing their equivalence. Given the encouraging short-term outcomes and the potential late morbidity benefits, we recommend PT for HNRMS whenever feasible.

### O 017: Proton Beam Therapy for orbital Rhabdomyosarcomas at West German Proton Therapy Center Essen (WPE)

#### D. Geismar ^1,2,3^, T. Steinmeier^1,3^, S. Nagaraja^1,2,3^, S. Peters^1,2,3^, S. Plaude^1,3^, S. Tippelt^4^, B. Timmermann^1,2,3^

##### *^1^West German Proton Therapy Center Essen WPE- Hufelandstraße 55- 45147 Essen- Germany, University Hospital Essen, Essen, Germany*, *^2^Clinic for Particle Therapy- University Hospital Essen- Hufelandstraße 55- 45147 Essen- Germany, University Hospital Essen, Essen, Germany*, *^3^West German Cancer Center WTZ- Hufelandstraße 55- 45147 Essen- Germany, University Hospital Essen, Essen, Germany*, *^4^University Hospital of Essen- Pediatrics III- Pediatric Hematology and Oncology- Essen- Germany, University Hospital Essen, Essen, Germany*

**Purpose**: Proton beam therapy (PT) is of increasing interest especially in tumors in close proximity of critical structures or in particular sensitive tissues like orbital rhabdomyosarcomas. Initial results of PT at WPE are presented.

**Methods**: Between April 2014 and September 2017, 16 patients (8 male, 8 female, median age 7.4 y (3.1-16.8 y)) with orbital rhabdomyosarcomas were treated at WPE and were prospectively enrolled in the in-house registry KiProReg. Histology types were embryonal rhabdomyosarcoma (87.5%) or alveolar rhabdomyosarcoma (12.5%). 37.5% had a parameningeal involvement. All patients were treated with chemotherapy (CTx) before radiotherapy and in 81.2% concomitant CTx was applied. The median PT dose was 50.4 Gy (41.4-54 Gy) applied in mean 28 fractions (23–30) by using uniform scanning (68.8%), pencil beam scanning (12.5%) or both techniques (18.7%), respectively.

**Results**: The median follow-up after last fraction is 1 year (0.0-2.4 y). 11 patients (68.8%) showed disease control. Local recurrence occurred in 4 patients and 1 patient developed a metastatic disease after treatment. No patients died so far. PT was well-tolerated. New high-grade (CTCAE ≥°3) acute toxicities occurred only in the field of hematological toxicity (n=5). Long-term data for 12 months after PT show a low number of new high-grade (CTCAE °3) toxicities regarding hematological toxicities. No new grade 4 or grade 5 effects occurred.

**Conclusion**: Current data support safety, tolerance and effectivity of PT in orbital rhabdomyosarcomas. However, long-term follow-up data is still needed to assess long-term outcomes.

### O 018: Carbon Ion Radiotherapy for Inoperable Pediatric Osteosarcoma

#### O. Mohamad^1^, R. Imai^2^, T. Kamada^2^, Y. Nitta^2^, N. Araki^3^

##### *^1^National Institute of Radiological Sciences Hospital of Charged Particles and University of Texas Southwestern Medical Center, Department of Radiation Oncology, Chiba, Japan*, *^2^National Institute of Radiological Sciences, Hospital of Charged Particles, Chiba, Japan*, *^3^Ashiya Municipal Hospital, Department of Surgery, Ashiya City- Hyogo, Japan*

**Background**: Osteosarcoma, the most common primary bone malignancy in children, has poor outcomes with conventional treatments especially if inoperable or in truncal sites.

**Methods**: We retrospectively reviewed the records of pediatric and adolescent patients who received carbon ion radiotherapy (CIRT) for inoperable osteosarcoma between 1996 and 2014.

**Results**: The cohort consisted of 26 patients aged 11-20 years (median 16) with inoperable high-grade osteosarcoma of the trunk (24 pelvic, 1 mediastinal and 1 paravertebral) without any other lesion at initial examination. There were 22 primary, 1 locally recurrent and 3 metastatic cases. All patients received prior chemotherapy and only 4 patients received prior surgery. Median CIRT dose was 70.4 Gy RBE (relative biological effectiveness) delivered in 16 fractions. Median follow-up was 32.7 months. Overall survival was 50.0% and 41.7% at 3 and 5 years, respectively. Ten patients survived for more than 5 years (range 5-20.7 years). Local control was 69.9% and 62.9% at 3 and 5 years, respectively. Fourteen cases developed distant metastasis and, thus, progression-free survival was 34.6% at 3 and 5 years (Figure 1). Only largest tumor diameter correlated with 5-year overall survival and local control. All patients tolerated the treatments well and there were 4 grade 3-4 CIRT-related late toxicities and 1 case of fracture in an irradiated bone. No treatment-related mortalities were observed. All patients (except 1) were able to ambulate after CIRT.

**Conclusions**: CIRT was safe and efficacious in the treatment of inoperable osteosarcoma with improved local control, overall survival and adverse events compared to conventional treatments.

### O 019: Gamma Electron Vertex Imaging (GEVI) for Proton Beam Range Measurement: Experimental Results

#### S.H. Kim^1^, J.H. Park^1^, Y. Ku^1^, H.R. Lee^2^, C.H. Kim^1^, S.K. Cho^3^

##### *^1^Hanyang University, Nuclear Engineering, Seoul, Korea Republic of*, *^2^Korea Atomic Energy Research Institute, Neutron Utilization Technology Division, Daejeon, Korea Republic of*, *^3^Samsung Medical Center, Radiation oncology, Seoul, Korea Republic of*

A prototype gamma electron vertex imaging (GEVI) system was developed to monitor the proton beam range in the patient by measuring prompt gammas. In the GEVI system, the prompt gammas from the patient are converted to electrons in the electron converter, and the electrons are then measured by the subsequent detectors (i.e., two hodoscopes and a calorimeter). Measuring the trajectories and the energies of the electrons, the vertices of the prompt gammas can be located, thereby producing a 2D prompt gamma image.

In the present study, prompt gamma distributions were obtained using the prototype GEVI system for the therapeutic proton beams at Samsung Medical Center (SMC) in Korea. The pencil beams, from 90 MeV to 180 MeV in 15 MeV steps, were delivered to a high-density polyethylene phantom. The GEVI system was fixed at a location for all proton energies considered in the study, and 6.24×109 protons were delivered for each measurement case.

Figure 1 shows the 2D prompt gamma images measured using the GEVI system. For all of the proton beams, the positions of the maximum counts on the 2D images were found to be directly in front of the beam range. Using the falloff positions of the GEVI images, a linear correlation between the falloff positions and beam ranges was derived with the coefficient of determination (r2) of 0.999. With this correlation, we believe that the proton beam ranges can be measured accurately, i.e., within ∼3 mm of error, for a wide range of proton beam energies.

### O 020 - How Risky Is 4D Planning? An FMEA-Based Human Error Comparison of 3D/4D PT QA Planning Risks Performed at CPT/PSI

#### F. Emert^1^, D. Pandya^2^, J. Hrbacek^1^, P. Morach^1^, J. Rottmann^1^, S. Zepter^1^, L. Podofillini^2^, A. Lomax^1^, D.C. Weber^1^, V.N. Dang^2^

##### *^1^Paul Scherrer Institute, Center for Proton Therapy, PSI-Villigen, Switzerland*, *^2^Paul Scherrer Institute, Risk and Human Reliability- LEA, Villigen PSI, Switzerland*

**Purpose**: Identification and quantification of human error risks providing failure minimization is of critical importance for patient safety in radiotherapy. This especially applies to new, complex workflows such as 4D-treatment planning. Therefore, a risk analysis comparing traditional 3D- with modern 4D-workflow implementation in proton therapy at Paul-Scherrer-Institute was performed. Overall goal is to compare failure risks of important tasks in a 4D-planning workflow with their counterparts in the 3D-planning process.

**Methods**: 5 tasks (3 specific for each workflow type, 2 common to both; 4D-example, Table1), their failure modes and causes (FC) were identified based on a Human Reliability Analysis (HRA) method developed at PSI and screening analysis by 5 experts who are familiar with 3D/4D-planning techniques. The risk estimation was based on the Failure Mode and Effect Analysis (FMEA) approach outlined in AAPM-TG100 report.

**Results**: Risk Priority Number (RPN) analyses of the tasks revealed a significantly higher risk of 4D- compared to 3D-planning processes (Fig1). RPN ratios [RPN(4D)/RPN(3D)] for both common tasks ranged from 1.2-1.9, for 3 related but workflow specific tasks between 1.3-8.8. The top FCs that received high risks were “less experienced with process” and “high workload” (Table1). High occurrence and low detectability were related to high RPNs.

**Conclusions**: Due to complicated 4D-planning workflow and its relatively newness, significantly higher risks are observed in 4D tasks than their 3D counterparts. By studying complete failure scenarios using the in-house developed HRA method, we will investigate tasks with highest risks and minimize failure rates by optimized distribution of QM/QA resources.

### O 021: Proton Range Verification through Acoustic Measurements

#### W. Nie^1^, K. Jones^2^, S. Petro^3^, A. Kassaee^3^, C. Sehgal^4^, S. Avery^3^

##### *^1^University of Pennsylvania, Department of Radiation Oncology- West Pavilion 2nd Floor. Room CN-495A, Philadelphia, USA*, *^2^Rush University, Radiation Oncology, Chicago- IL, USA*, *^3^University of Pennsylvania, Radiation Oncology, Philadelphia, USA*, *^4^University of Pennsylvania, Radiology, Philadelphia, USA*

Proton radiation therapy requires a simple and accurate method to measure the proton beam Bragg peak (BP) depth for monitoring patient treatment. Protoacoustics - measurement of the pressure waves emitted by thermal expansion resulting from proton dose deposition - may be used to obtain the position of the BP in a phantom by measuring the time-of-flight (TOF) of the maximum amplitude pressure wave. We performed acoustic measurements and developed four different methods for analyzing the protoacoustic signals produced in homogeneous phantoms made from metallic and plastic materials. Analysis shows that among these four methods, the best exhibits minimal error (0.2cm). After comparing multiple materials and shapes, we conclude that the rectangular high density polyethylene phantom is most appropriate for proton range verification due to its durability and convenience. To characterize the ideal detection device, the protoacoustic signals generated in the polyethylene phantom were measured with three different detectors: hydrophone, accelerometer, and laser vibrometer. The accelerometer and vibrometer demonstrate similar sensitivity as the hydrophone: the accelerometer has light weight and small size, therefore it is easy to be attached on the patient exterior; the vibrometer has advantages in multi-spot scanning remotely and simultaneously. Psuedospectral wave-equation simulations (k-Wave MATLAB toolbox) were performed using phantom CT images to study the effect of CT number error on protoacoustic range verification. To reduce the uncertainty in TOF measurements and CT error, we can apply a triangulation algorithm. Simulation study shows we can reduce the uncertainty due to the uncertainty to less than 0.5mm.

### O 023: An Activity to Improve Quality through Dose Inter-comparison by End-To-End Testing in Carbon-Ion Radiation Therapy Centers in Japan

#### H. Mizuno^1^, A. Fukumura^2^, N. Kanematsu^2^, S. Yonai^2^, K. Yusa^3^, T. Yanou^4^, M. Suga^5^, M. Mizota^6^, S. Minohara^7^, T. Kanai^3^

##### *^1^National institute of Radiological Sciences- QST, Dept. of Radiation Measurement and Dose Assessment, Chiba, Japan*, *^2^National institute of Radiological Sciences- QST, Clinical Research Cluster, Chiba, Japan*, *^3^Gunma University, Heavy Ion Medical Center, Maebashi, Japan*, *^4^Hyogo Ion Beam Medical Center, Department of radiation technology, Tatsuno, Japan*, *^5^Hyogo Ion Beam Medical Center, Department of radiation phyiscs, Tatsuno, Japan*, *^6^SAGA HIMAT Foundation, Ion Beam Therapy Center, Tosu, Japan*, *^7^Kanagawa Cancer Center, Department of radiation therapy, Yokohama, Japan*

For multicenter clinical studies of carbon-ion radiation therapy (C-ion RT), dose uniformity among centers is crucial. In the clinical studies of The Japan Carbon-ion Radiation Oncology Study group (J-CROS), dosimetric inter-comparison for all centers participating in clinical studies was performed using the end-to-end test method. All five centers that had performed C-ion RT in Japan in 2017 participated. A dedicated water phantom was produced for the test. CT imaging and treatment plan was performed using the same method as the patient, the physical dose of the isocenter was measured by the ionization chamber dosimeter, and compared with the calculated dose from the treatment planning system. As a result, the difference between the measured and calculated doses in all five facilities were within ± 3%, tolerance level. Uniformity of dose among institutions is guaranteed. In addition, in response to the inter-comparison dosimetry, some centers have begun actions to improve the dosimetry precision. For instance, thermometers and barometers used for absolute dosimetry in one center that had not been calibrated as those mounted in the irradiation system were finally calibrated. In another center, an old type of ionization chamber used as the reference dosimeter was updated to a new type. The team of J-CROS medical physicists continues with this kind of QA activity among C-ion RT centers.

### O 024: Differences in Human Endothelial Cell Activation after Irradiation with Photons vs. Protons in the Proximal and Distal Spread-Out Bragg Peak

#### R. Vatner^1^, H. Shen^2^, J. Goines^2^, D. Ionascu^3^, E. Wolf^3^, W. Kassing^3^, M. Lamba^3^, E. Janssen^4^, A. Mascia^3^, E. Boscolo^5^

##### *^1^University of Cincinnati / Cincinnati Children's Hospital Medical Center, Radiation Oncology, Cincinnati, USA*, *^2^Cincinnati Children's Hospital Medical Center, Cancer and Blood Diseases Institute, Cincinnati, USA*, *^3^University of Cincinnati, Radiation Oncology, Cincinnati, USA*, *^4^Cincinnati Children's Hospital Medical Center, Immunobiology, Cincinnati, USA*, *^5^Cincinnati Children's Hospital Medical Center, Experimental Hematology and Cancer Biology, Cincinnati, USA*

**Purpose/Objectives**: Radiation necrosis is an infrequent but morbid late effect of radiotherapy for brain tumors, with anecdotes of a propensity at the distal end of the spread-out Bragg peak (SOBP). The mechanism is unknown, but a prevailing hypothesis is that radiation necrosis results from inflammation and microvascular dysfunction due to endothelial cell injury. We test the hypothesis that proton irradiation at the distal SOBP induces more endothelial activation than irradiation with photons or proximal SOBP protons.

**Materials/Methods**: Human umbilical vein endothelial cells (HUVECs) were treated to 0Gy, 2Gy, or 8Gy with 0.662MeV photons, or 70-240MeV protons in the proximal or distal 2cm of 10cm SOBP. Cells were incubated at 37C for 24h and inflammatory cytokine and chemokine expression was tested using the Human Angiogenesis Array C1000 (RayBiotech). At 48h flow cytometry was performed using antibodies for MHC Class I, ICAM-1 and HCAM.

**Results**: Proximal and distal SOBP proton treated cells had diminished viability compared with photon treated cells. There was a dose dependent increase in expression of MHC-I and CD54/ICAM-1, and decreased CD44/HCAM after proton vs. photon treatment. Differences in inflammatory cytokines and chemokines were also dose dependent with a trend toward greater effects in proton vs. photon treated groups, and no significant difference between cells treated with proximal vs. distal SOBP.

**Conclusion**: Proton and photon radiation activate a dose dependent inflammatory profile in HUVECs. Proton radiation induces a stronger inflammatory response compared with photons. No significant differences were detected in effects between proximal and distal SOBP.

### O 025: Drosophila Melanogaster may Be a Useful Model Organism to Study the Biological Effects of Proton Radiation

#### K. Nakajima^1,2^, H. Iwata^1,2^, M. Naito^3^, S. Hirai^3^, K. Kume^4^, G. TianXiang^4^, C. Omachi^5^, J.E. Mizoe^1,6^, H. Ogino^1^, Y. Shibamoto^2^

##### *^1^Nagoya Proton Therapy Center- Nagoya City West Medical Center, Department of Radiation Oncology, Nagoya, Japan*, *^2^Nagoya City University Graduate School of Medical Sciences, Department of Radiology, Nagoya, Japan*, *^3^Aichi Medical University, Department of Anatomy, Nagakute, Japan*, *^4^Nagoya City University, Department of Neuropharmacology, Nagoya, Japan*, *^5^Nagoya Proton Therapy Center, Department of Proton Therapy Physics, Nagoya, Japan*, *^6^Osaka Heavy Ion Therapy Center, Department of Radiation Oncology, Osaka, Japan*

**Background**: Biological effects of proton beams have been well investigated in vitro. In contrast, however, their effects in in vivo systems are not sufficiently understood. Drosophila melanogaster is an organism well-suited for genetic analysis and commonly used in medical research. This is a study to demonstrate the usefulness of the fly as a model organism to investigate the biological effects of proton beams.

**Materials/Methods**: Third-instar larvae of wild-type Drosophila melanogaster (Oregon-R) were irradiated with 6-MV X-rays or passive proton beams at the center of the spread-out Bragg peak. They were exposed to single doses of 0-50 Gy and observed for up to 48 hours after eclosion. RT-qPCR for mRNA was performed using 40-Gy irradiated samples.

**Results**: The surviving fraction within 48 hours was: control (0 Gy), 0.87; 10 Gy, 0.75/0.92 (photon/proton); 20 Gy, 0.81/0.73; 30 Gy, 0.73/0.37; 40 Gy: 0.52/0.30; and 50 Gy, 0.12/0.05. Two-way ANOVA revealed significant differences between the radiation types (f1) and doses (f2); p-values were 0.023 for f1, <0.001 for f2, and 0.015 for f1f2. In the analysis of mRNA expression of 26 genes, remarkable differences were observed between photons and protons in genes associated with DNA repair, such as Ku70, Ku80 and Rad51.

**Conclusions**: To date, this is the first study using Drosophila to investigate the biological effects of proton beams. We demonstrated the differences between photons and protons in both phenotypes and genotypes in the in vivo system. This experimental model may be useful in elucidating biological aspects of proton beams.

### O 026: Radiobiological Impact of Mixed Field from Target Fragments in Proton Treatment Plans

#### V.E. Bellinzona^1^, E. Scifoni^1^, M. Krämer^2^, F. Tommasino^1,3^, G. Petringa^4^, P. Cirrone^4^, F. Romano^4,5^, L. Grzanka^6^, T. Friedrich^2^, M. Durante^1^

##### *^1^INFN - Istituto Nazionale di Fisica Nucleare, TIFPA-Trento Institute for Fundamental Physics and Applications, Trento, Italy*, *^2^GSI Helmholtzzentrum für Schwerionenforschung GmbH, Biophysics, Darmstadt, Germany*, *^3^Università degli Studi di Trento, physics, Trento, Italy*, *^4^INFN - Istituto Nazionale di Fisica Nucleare, LNS - Laboratori Nazionali del Sud, Catania, Italy*, *^5^National Physical Laboratory, CMES - Medical Radiation Science, Teddington, United Kingdom*, *^6^Institute of Nuclear Physics Polish Academy of Sciences, Physics and Astronomy, Cracow, Poland*

Among the various radiobiological effects of proton beams, the impact of target fragments on Relative Biological Effectiveness (RBE) is an open issue and TPS would possibly benefit from an implementation accounting for this effect. Recently this topic has become timelier since new experiments like FOOT (FragmentatiOn Of Target) promise to get relevant physics data with unprecedented details. In this scenario, we ask: Is this correction actually needed in clinical practice? Within the MoVe¬IT project, we aim to answer, analyzing he overall mixed field arising in the irradiated medium and the RBE composed by all the produced secondary fragments compared to the pure primaries effect. By using Monte Carlo methods (Geant4), we have produced the spectra of those fragments for different initial proton energies in water to implement them in TRiP98 and to evaluate their impact with a combination of LEM IV model and mixed field algorithm, in order to account the biological dose contribution of each single fragment and reconstruct the overall RBE. First steps of the analysis of the spectra indicate a sensible contribution of physical dose of the target fragments, including those with Z>2 and thus considering their low energy, a possible correction on the biological dose in treatment plans is expected. The fragmentation spectra data will be validated against experimental data from the partner experiment FOOT on a second step. Several scenarios of TRiP98 computed plans to investigate the dose variation by accounting for the RBE variation due this effect will be presented.

### O 027: Differential Inflammatory and Metabolic Gene Expression Profiles of Murine and Human Cancer Cell Lines after Proton vs. Photon Irradiation

#### S. Singhaviranon^1^, H. Shen^2^, M. Sertorio^2^, S. Langevin^3^, D. Ionescu^4^, W. Kassing^4^, M. Lamba^4^, A. Mascia^4^, E. Janssen^5^, R. Vatner^6^

##### *^1^University of Connecticut School of Medicine, Immunotherapy of Cancer and Infectious Diseases, Farmington- CT, USA*, *^2^Cincinnati Children's Hospital Medical Center, Cancer and Blood Diseases Institute, Cincinnati- OH, USA*, *^3^University of Cincinnati, Department of Environmental Health, Cincinnati- OH, USA*, *^4^University of Cincinnati, Department of Radiation Oncology, Cincinnati- OH, USA*, *^5^Cincinnati Children's Hospital Medical Center, Division of Immunobiology, Cincinnati- OH, USA*, *^6^University of Cincinnati / Cincinnati Children's Hospital Medical Center, Department of Radiation Oncology, Cincinnati, USA*

**Purpose**: The biological effects of proton and photon radiation are assumed to be qualitatively equivalent with only the quantitative difference in relative biological effectiveness of 1.1 CGE/Gy of proton physical dose. However, the physical interactions between these modalities and cells are fundamentally different. Using whole transcriptomic analysis, we test the hypothesis that proton and photon radiation elicit qualitatively different biological responses in cancer cells.

**Methods**: Murine embryonal rhabdomyosarcoma (M3-9-M) and human hypopharyngeal squamous cell carcinoma (FaDu) cell lines (50-80% confluent) were treated to a dose of 0, 2, or 8 Gy with photons (6 MV or 0.662 MeV) or protons (70-240 MeV SOBP), and incubated 37C for 2 or 24 hours. RNA-seq (75bp paired-end, 40M reads) was performed from cDNA libraries constructed from isolated mRNA using a HiSeq2500 (Illumina). Differential expression and pathway enrichment analysis was performed using AltAnalyze and WikiPathways, and induced genes were cross referenced against expression data from irradiated human and murine leukemia cell lines.

**Results**: Differential gene expression patterns induced by proton vs. photon irradiation involve multiple pathways, including cell cycle control, DNA repair, inflammation, metabolism and cholesterol biosynthesis. Photons induce a pronounced relative increase in oxidative stress genes with concordant down-regulation of the cholesterol biosynthesis, consistent with shunting of NADPH towards the glutathione pathway for management of oxidative stress.

**Conclusion**: Proton and photon radiation induce differential patterns of gene expression in both human and mouse cancer cell lines, with a pattern suggestive of a relative increase in oxidative stress induced by photons vs. protons.

### O 028: Comparison of the Effect of X-ray, Carbon Ion Beam and Proton Beam on Metastatic Potential

#### R. Kondo^1^, T. Adachi^1^, K. Minami^2^, M. Koizumi^1^

##### *^1^Osaka University Graduate School of Medicine, Department of Medical Physics and Engineering, Suita- Osaka, Japan*, *^2^Osaka University Graduate School of Medicine, Department of Radiation Oncology, Suita- Osaka, Japan*

Metastasis, the biggest threat to survival for patients with solid tumors, is the spread of tumor cells from the original growth to the other sites in the body. In the clinic, ionizing radiation has been established as a highly effective modality used in the local control of tumor growth. Recently, particle beam therapy using carbon ion or proton beam has good local control to radio-resistant carcinoma too. However, there are little known that effects of these radiations for cancer metastasis. Therefore, in this study, we investigated about effects of three types of radiations (X-ray, carbon ion beam and proton beam) on metastatic potential via in vitro and in vivo.

Cell migration and invasion capability were increased by sublethal dose X-ray. On the other hand, carbon ion beam was decreased them dose-dependently. In the case of proton beam, metastatic potentials were not changed on sublethal dose and were decreased on higher dose. in vivo, X-ray irradiation resulted in a 1.2 fold increase in the number of metastatic lung nodules in mice as compared to mice injected with untreated cells. However, a significant suppression of lung metastases was observed in cells irradiated with carbon ion or proton beam. As a cause of these differences, we focused on the effect of radiations on cytoskeletal related protein.

### O 030: Is Deposited Energy a Useful Metric in the Quality Assurance of Pencil-Beam-Scanning Plans?

#### R. Slopsema^1^, S. Flampouri^1^

##### ^1^University of Florida Health, University of Florida Health Proton Therapy Institute, Jacksonville, USA

Summation of the MU-weighted energy of all spots, over all beams in a PBS plan yields the total deposited energy. The locally deposited energy is calculated by limiting the integration to energies above the lowest spot-energy in the beam (Fig1). Because of margins and beam penumbrae, only a fraction of this energy is deposited inside the target volume. The fraction of the energy deposited outside the target volume is defined as the spill factor SF. For any PBS plan the spill factor can be calculated from the energies and MUs in the plan combined with the target volume in cc and prescribed dose. SF depends on the target volume and site-specific planning margins.

Per treatment site we parameterized the SF as function of target volume by fitting to multiple treatment plans. The line in Fig2 shows this parameterization for lumbar-chordoma plans. Next we evaluated the quality of plans by determining the deviation of the SF from the parameterization. Plans that undercover the target had a SF lower than expected; plans that over-cover larger than expected. The effect of deliberate planning errors on the SF was evaluated (green/red points). This SF-analysis was performed for 125 PBS QA plans for 11 treatment sites.

Overall 89% of the plans had a SF within ±10% of expected. For sites with regular-shaped targets and standardized protocols (spinal-axis, brain, prostate) the agreement with the fit was best and deliberate planning errors could be detected, indicating that for these sites deposited energy can be a useful QA metric.

### O 031: Proton Beam Ruler: A Fast Proton Range Measurement Tool Using a Scintillator Block and Camera

#### D. Robertson^1^, X. Ding^1^, M. Bues^1^

##### ^1^Mayo Clinic Arizona, Radiation Oncology, Phoenix, USA

**Introduction**: Proton beam range measurement is important, but it is also resource-intensive. Scintillator-based detectors can quickly and accurately measure proton range with high spatial resolution. We describe the “proton beam ruler”, a scintillator-based proton range measurement device that is optimized for clinical use. We report on its accuracy and measurement efficiency.

**Methods**: The proton beam ruler comprises a 10x10x40 cm3 block of plastic scintillator imaged by a digital camera. Self-calibration marks are inscribed into the scintillator, which is placed in a light-blocking housing and indexed on the treatment couch (Figure 1a). Camera acquisition is triggered using signals from the synchrotron. Range measurement for 97 clinical beam energies was performed during a single irradiation using 0.1 monitor units per energy (Figure 1b). The water-equivalent thicknesses (WET) of tissue-equivalent inserts from a commercially available CT Hounsfield Unit phantom were also measured.

**Results**: The difference in measured ranges between the proton beam ruler and a water tank with Bragg peak chamber was 0.01 mm (σ=0.05 mm) with 0.18 mm maximum deviation for scintillator measurements repeated over 5 days (Figure 2a). Setup time was 5 minutes, and measurement time for 97 beam ranges was 5 minutes. WET measurements of the Hounsfield Unit phantom inserts differed by 0.12 mm (σ=0.05 mm) from water tank measurements (Figure 2b). Each WET measurement required 10 seconds of beam time.

**Conclusions**: The proton beam ruler can quickly and accurately measure proton range and the WET of clinical devices, decreasing quality assurance time requirements and facilitating more comprehensive beam testing.

### O 032: Highlights from the Second International Proton Treatment Efficiency Workshop Sponsored by the PTCOG Treatment Efficiency Subcommittee

#### C. Beltran^1^, D. Mundy^1^

##### ^1^Mayo Clinic, Radiation Oncology, Rochester, USA

**Purpose**: The purpose of the 2nd International Proton Treatment Efficiency Workshop with a focus on Quality Assurance and Treatment Planning was to inform each other of our current clinical practices, identify current limitations, and discuss new approaches and techniques with the goal of improving the safety and efficiency in these areas.

**Methods**: A 2.5 day meeting was held in Knoxville Tn. Short presentations on Daily, monthly, and patient specific quality assurance (PSQA) followed by in depth group discussion/debate were done on the first day. The second day covered treatment planning including robust optimization, robust analysis, verification CT imaging/planning, and image guidance for proton therapy. The last half day was used to summarize and document the discussions.

**Results**: Nearly30 participants attended the workshop, spanning the US and Europe, some with facilities under construction and others that have well established facilities. Discussion on QA and limitations of current vended solutions were expressed. There was consensus on the merit of log based PSQA however, many proton system vendors do not allow access to the log files. Recommendation that access to meaningful log files should be negotiated upfront before purchase was made. Use of robust optimization was encouraged; limitations on speed and usability were expressed. Surface imaging was stated as a large efficiency gain for breast patients.

**Conclusion**: Vended solutions for proton QA are still lacking and access to log files should be a priority. Daily imaging, particularly surface imaging for breast, can lead to efficiency gains.

### O 033: In-Room Characterization, Using an Anthropomorphic Phantom, of a Novel Monitor Exploiting Secondary Charged Particles Emission in Light Ion PT Treatments

#### A. Sarti^1,2,3^, M. De Simoni^4,5^, Y. Dong^6,7^, C. Mancini Terracciano^4,5^, M. Marafini^2,5^, R. Mirabelli^4,5^, S. Muraro^8^, G. Traini^4,5^, S.M. Valle^6,7^, I. Mattei^7^

##### *^1^Università di Roma “La Sapienza”, Scienze di Base e Applicate per l'Ingegneria, Rome, Italy*, *^2^Museo Storico della Fisica e Centro Studi e Ricerche “E. Fermi”, Fisica, Rome, Italy*, *^3^Istituto Nazionale di Fisica Nucleare, Laboratori Nazionali di Frascati, Frascati, Italy*, *^4^Università di Roma “La Sapienza”, Fisica, Rome, Italy*, *^5^Istituto Nazionale di Fisica Nucleare, Sezione di Roma, Rome, Italy*, *^6^Università di Milano, Fisica, Milan, Italy*, *^7^Istituto Nazionale di Fisica Nucleare, Sezione di Milano, Milan, Italy*, *^8^Istituto Nazionale di Fisica Nucleare, Sezione di Pisa, Pisa, Italy*

The use of C, He and O as beam particles when administering Particle Therapy (PT) treatments is getting more and more widespread as a consequence of the enhanced Relative Biological Effectiveness and Oxygen Enhancement Ratio of such projectiles. The advantages in the tumour control probability, related to the improved efficacy of the incoming radiation, require an accurate online monitor of the dose release spatial distribution.

The monitor main purpose is to prevent unwanted damage to the tissues surrounding the tumour that can arise, for example, due to morphological changes occurred in the patient during the treatment with respect to the initial CT scan. PT treatments with C, He and O ions can be monitored by detecting the secondary radiation produced by the primary beam interactions with the patient body along the path towards the target volume. Secondary charged fragments (produced mainly by the projectile fragmentation) can be emitted at very large angles with respect to the incoming beam direction and can be detected with high efficiency in a nearly background free environment. The Dose Profiler (DP) detector, developed within the INSIDE project, is a scintillating fibre tracker that allows an online charged fragments reconstruction and backtracking.

The construction and preliminary tests performed on the DP, carried out using the 12C ions beam of the CNAO treatment centre using a RANDO® anthropomorphic phantom as a target, will be reviewed in this contribution.

A discussion of the results implications for a pre-clinical trial on CNAO patients, foreseen in 2018, will be made.

### O 034: Scintillator-Based System for Transversal Dose Pro Files Reconstruction

#### R. Catalano^1^, G. Petringa^1,2^, G. Cuttone^1^, M. Durante^3^, G. Pitta^4^, S. Puglia^1^, L. Raffaele^5^, E. Scifoni^3^, F. Tommasino^3^, P. Cirrone^1,6^

##### *^1^Istituto Nazionale di Fisica Nucleare, Laboratori Nazionali del Sud, Catania, Italy*, *^2^Università degli Studi di Catania, Dipartimento di Fisica e Astronomia, Catania, Italy*, *^3^INFN, Trento Institute for Fundamentals Physics and Applications, Trento, Italy*, *^4^DE.TEC.TOR., Devices and Technologies Torino s.r.l., Torino, Italy*, *^5^Università degli Studi di Catania, Azienda Ospedaliero-Universitaria Policlinico, Catania, Italy*, *^6^Institute of Physics ASCR, ELI-Beamlines Project, Prague, Czech Republic*

A fast and reliable system to measure transversal charged particles relative dose profiles is desirable in any hadron therapy facility, being the basis for an accurate treatment quality assessment procedure. For this purpose a system was developed at the “Laboratori Nazionali del Sud” of Italian Institute for Nuclear Physics (INFN-LNS, Catania, I) consisting of a plastic scintillator screen (50 × 50 mm2, variable in thickness from 0.5 mm to 1.0 mm), mounted perpendicularly to the beam axis and coupled with a highly sensitive cooled CCD detector (resolution 1928 × 1452 pixels) in a light-tight box. In-house custom software for real-time data acquisition and processing was also developed in the LabView 2016 environment.

In this work the characterization of the system is reported: transversal dose profile in terms of FWHM, field lateral penumbra, flatness, symmetry, reproducibility and linearity with beam current have been investigated.

A comparison with other common quality control devices able to perform transversal beam profiles reconstruction (Gafchromic films, silicon diodes, Lynx detector and Timepix detector) has been also carried out both in small- and large-field proton beams at the INFN-LNS proton therapy facility (Catania, Italy). No significant differences have been observed in respect to conventional systems at both the INFN-LNS proton therapy facility (Catania, Italy) and the Trento Institute for Fundamental Physics and Applications (TIFPA, Trento, I). Results will be reported and discussed.

### O 035: Implementation of a Non-Invasive Online Beam Monitor at a 60 Mev Proton Therapy Beamline

#### R. Schnuerer^1^, J.S.L. Yap^1^, H. Zhang^1^, G.J. Haefeli^2^, O. Girard^2^, M. Hentz^3^, T. Szumlak^4^, C.P. Welsch^1^

##### *^1^Cockcroft Institute/ University of Liverpool, Physics, Warrington, United Kingdom*, *^2^École Polytechnique Fédérale de Lausanne, Physics, Lausanne, Switzerland*, *^3^University College London, Physics, London, United Kingdom*, *^4^Akademia Górniczo-Hutnicza, Physics, Krakow, Poland*

Online beam monitoring in medical accelerators is essential in assuring patient safety as well as the high quality and efficacy of cancer treatment. In clinical practice for proton therapy, currently used ionization chambers are interceptive devices, degrading both the beam profile and its energy spread. Therefore, a new non-interceptive approach of online beam monitoring is highly desirable.

The Vertex Locator (VELO) detector is a multi-strip silicon detector used in the LHCb experiment at CERN. The semi-circular design and position of its sensitive silicon detector offers a non-invasive way to measure the beam intensity through a precise measurement of the beam halo without interfering with the beam core. To be integrated to the specific conditions of the clinical environment in a proton therapy center, VELO was adapted in a standalone system.

In this contribution, Geant4 Monte Carlo simulations of the beam parameters including the beam profile, energy spread and halo to dose correlations were performed to investigate the integration of the detector in the treatment line and behavior of the beam during delivery.

Furthermore, initial results with the VELO detector integrated at the 60 MeV proton therapy beamline at the Clatterbridge Cancer Centre (CCC), UK are presented. Synchronized with a locally constructed Faraday Cup, the quality of the beam monitor is assessed by measuring the beam current at different dose rates and by monitoring the beam halo profile at different positions along the beamline. Future beam and halo propagation studies will look into further application for the VELO standalone online monitor.

### O 036: Prospective Study of 3 Fraction Pencil-Beam Scanning Partial Breast Irradiation: Early Provider and Patient-Reported Outcomes of a Novel Regimen

#### K. Jethwa^1^, K. Gonuguntla^1^, T. Whitaker^1^, S. Park^1^, T. Hieken^2^, L. McGee^3^, K. Ruddy^4^, K. Corbin^1^, N. Remmes^1^, R. Mutter^1^

##### *^1^Mayo Clinic, Radiation Oncology, Rochester- MN, USA*, *^2^Mayo Clinic, Surgery, Rochester- MN, USA*, *^3^Mayo Clinic, Radiation Oncology, Scottsdale- AZ, USA*, *^4^Mayo Clinic, Medical Oncology, Rochester- MN, USA*

**Purpose/Background**: The optimal dose and fractionation for proton accelerated partial breast irradiation (APBI) is not known. Herein, we report dosimetry, early adverse effects (AEs), cosmetic, and patient-reported outcomes (PROs) of a prospective study of 3-fraction pencil-beam scanning (PBS) APBI.

**Methods**: Eligibility criteria included women age ≥ 50 years with estrogen receptor positive (ER+), sentinel lymph node negative invasive or in-situ breast cancer measuring ≤ 2.5 cm. The prescription was 21.9 Gy (RBE 1.1) in 3 daily fractions to the post-operative tumor bed with a 1 cm expansion. Common toxicity criteria for adverse effects (CTCAE v 4.0), 10-point linear analog scales (LASA), patient-reported outcomes version of the CTCAE (PRO-CTCAE), and Harvard Breast Cosmesis Scale (HBCS) were utilized for provider and patient-reported assessments.

**Results**: 76 women were treated between 2015 and 2017. The mean age was 66 (SD 8.6) years. Most patients had grade 1-2 (92%) invasive breast cancer (80%), of ductal histology (92%), measuring ≤2 cm (95%). A median of 2 (range 1-3) treatment fields were used to achieve favorable target coverage and normal tissue dosimetry (table 1). Mean follow-up was 6 months (SD 5.8). Physician-assessed AEs and PROs are shown in table 2. At last follow-up, no treatment-related grade ≥ 2 AEs have been observed, and all patients are alive without relapse.

**Conclusions**: 3-fraction PBS-APBI is well tolerated with low rates of physician and patient-reported adverse effects. Follow-up is ongoing to assess the late toxicity and disease control outcomes of this novel strategy for APBI.

### O 037: Improved Long-Term Patient-Reported Health and Well-Being Outcomes of Early-Stage Breast Cancer Treated with Partial Breast Proton Therapy

#### S. Teichman^1^, S. Lum^2^, J. Slater^1^, D. Bush^1^

##### *^1^Loma Linda University Medical Center, Radiation Medicine, Loma Linda, USA*, *^2^Loma Linda University Medical Center, Surgical Oncology, Loma Linda, USA*

**Background**: Long-term quality of life (QoL) is important in assessing outcomes of breast conservation therapy for patients with early-stage breast cancer. This study compares patient-reported QoL outcomes among women with stage 0-II disease treated via lumpectomy followed by whole-breast photon (WBI) or partial-breast proton therapy (PBPT).

**Methods**: Subjects receiving WBI or PBPT were recruited from institutional research database and prior phase II clinical trials. Participants evaluated QoL several years post-treatment by responding to subjective instruments, including established scalar questionnaires and self-report measures. Responses were averaged between the two groups for statistical analysis.

**Results**: 129 subjects completed QoL evaluations, 57 following WBI and 72 following PBPT. At 6.5 years (median) post-diagnosis, participants' demographic and clinical characteristics were similar. Patient-reported outcomes were reported as mean scale scores for the two groups, all displaying significant differences favoring proton irradiation: Cosmesis (p<0.001); pain/sensitivity(p<0.05); breast texture or shape (p<0.001); clothing fit (p<0.001); fatigue (p<0.001); daily-life fatigue impact (p<0.05 to p<0.001); self-consciousness (appearance dissatisfaction) (p<0.05); attractiveness self-opinion (p<0.05); contentedness (p<0.01); fear of recurrence (p<0.001); and happiness with treatment choice (p<0.001).

**Conclusion**: Patients' responses suggest that partial-breast proton radiotherapy is associated with higher overall QoL compared with WBI. These self-perceptions prevail up to >10 years post-treatment. Proton therapy may enhance QoL in a cascade of ways.

### O 038: Initial Experience with Barrier Film Dressing for Dermatitis Prophylaxis in Breast Proton Therapy

#### K. Corbin^1^, K. Roberts^1^, S. James^1^, S. Park^1^, E. Yan^1^, K. Klein^1^, J. Lubahn^1^, C. Loprinzi^1^, I. Petersen^1^, R. Mutter^1^

##### ^1^Mayo Clinic, Radiation Oncology, Rochester, USA

**Purpose/Background**: Radiation dermatitis is a common acute toxicity of breast cancer radiotherapy. Mepitel film (MF) appears to reduce acute radiation dermatitis (RD) in photon patients. We report the feasibility and initial outcomes of PMRT patients treated with proton beam (PBT) and MF.

**Methods**: Patients were treated with multi-field optimized pencil-beam scanning IMPT to the chest wall and regional lymph nodes, most commonly with a 2-field oblique beam arrangement, per institutional standards. The median prescription dose was 50 Gy (RBE 1.1) in 25 fractions. Skin dose was individualized, with typical planning goals of D90>90% and D1cc < 105%. MF was applied by nurses prior to treatment and replaced approximately weekly. Common toxicity criteria for adverse effects (CTCAE v 4.0), patient-reported outcomes (PRO) version of the CTCAE (PRO-CTCAE), and 10-point linear analog scales (LASA) were collected prospectively to evaluate skin toxicity.

**Results**: 33 patients underwent proton PMRT with MF. 20 were reconstructed with tissue expanders and 13 were non-reconstructed. Four of 33 patients discontinued for pruritis (2), film non-adherence (1), and rash unrelated to MF (1). Median skin D1cc was 103.5% (Range: 95.8-120%). Physician assessed and patient reported outcomes are shown in Table 1. Maximal physician reported RD was grade 1 -2 in 97% at completion. Just 9 patients reported severe or very severe radiation burns.

**Conclusions**: Post mastectomy IMPT with MF was feasible, resulting in promising patient and physician reported acute toxicity. Based on this preliminary experience, we are planning a randomized trial of MF in PMRT.

### O 039: Proton Beam Therapy for Hepatocellular Carcinoma Associated with Inferior Vena Cava Tumor Thrombus

#### Y. Sekino^1^, N. Fukumitsu^1^, T. Okumura^1^, H. Numajiri^1^, M. Mizumoto^1^, K. Ohnisihi^1^, T. Aihara^1^, H. Ishikawa^1^, K. Tsuboi^1^, H. Sakurai^1^

##### ^1^University of Tsukuba, Radiation Oncology, Tsukuba, Japan

**Background**: Patients with hepatocellular carcinoma (HCC) associated with inferior vena cava tumor thrombus (IVCTT) have poor prognosis. The purpose of this study is to evaluate the safety and effectiveness of proton beam therapy (PBT) for patients with HCC associated with IVCTT.

**Methods**: Fifteen patients who had HCC with IVCTT were treated with PBT at University of Tsukuba between 2005 and 2014. Median tumor size was 9 (range 4-20) cm. Tumor thrombus extended to right atrium in 10 patients. Portal vein tumor thrombosis was found in 4 cases. Total delivered dose ranged from 50 to 74 Gray equivalent (GyE) and the most frequently used dose-fractionation was 72.6 GyE in 22 fractions. Seven patients were treated with curative intent. The local tumor control rates (LC), overall survival rates (OS), and toxicities were evaluated.

**Results**: Median follow-up was 20.1 months. The OS and LC rates at 1 year were 66.7% and 85.1%, respectively. For patients with curative intent, median OS was 25.1 months and LC was achieved in all cases. Recurrence was observed in 12 patients, intrahepatic recurrence in 5 patients and lung metastases in 5 patients. Lung metastases occurred within 3 months in 4 cases. No patients were suffered from radiation-induced liver disease or other severe toxicity of grade 3 or greater. One patient had grade 2 gastrointestinal hemorrhage.

**Conclusions**: PBT showed favorable effectiveness and safety, and can play a role in treatment for HCC associated with IVCTT.

### O 040: Proton Radiotherapy for Locally Advanced Pancreatic Cancer (LAPC) – Limiting Duodenal Toxicity

#### P. Vitek^1^, J. Kubes^1^, V. Stepan^1^, V. Vondracek^2^, J. Kvech^1^, B. Ondrova^1^, K. Dedeckova^1^, A. Pasztorova^1^, G. Kasacova^1^

##### *^1^Proton Therapy Center Czech, Proton radiotherapy, Praha 8, Czech Republic*, *^2^Proton Therapy Center Czech, Dept. of Physics, Praha 8, Czech Republic*

**Introduction**: Dose escalation and concomitant chemotherapy for LAPC may improve local control and prolong survival. The duodenum, adjacent to pancreas head, potentially is the most critical organ. If the regional lymph-nodes (LNN) are encompassed to CTV, the substantial volume of D1-D4, receives the full prescribed dose. The dosimetry of pencil beam scanning (PBS) proton radiotherapy was analysed and correlated to the observed toxicity.

**Methods**: Proton PBS radiotherapy was administered in 30 patients with inoperable LAPC with concomitant chemotherapy (capecitabine or gemcitabine), hypofractionated regimen – 54 GyE/18 fractions. The regional lymph nodes - peripancreatic, coeliac, portal, and upper paraaortic group were encompassed CTV. It extended caudally beyond lower-most margin of duodenum - to lower edge of L2 or L3.

**Results**: The median volume of delineated duodenum was 53 ccm (24 – 118) median of Dmean in duodenum 37 GyE, median of V45GyE 29 ccm (2,8 -50 ccm). The medians of V45GyE for stomach and small intestine were 21,5 and 8,0 ccm respectively. (Table 1). Signs of acute duodenitis developed in 6 patients, duodenal ulceration in 3 patients, perforation in 1, with a fatal outcome. The time of ulceration onset was 3-6 months after radiotherapy. Other side effects did not exceed grade 2.

**Conclusions**: Duodenal toxicity limits the dose and extent of PTV for LAPC. The anatomy of pancreas and LNN allows only limited saving of duodenum. Low duodenal dose may be maintained if LLN are included in CTV. A separate dose constraint of duodenum and small intestine for normo and hypofractionated radiation is warranted.

### O 041: Is Proton SBRT an Attractive Option for Liver Metastases? Results of a 5-Year Observational Study for 80 Hepatic Lesions

#### D. Sufficool^1^, P. McGee^1^, J. Slater^1^, G. Yang^1^

##### ^1^Loma Linda University, Radiation Medicine, Loma Linda, USA

**Purpose/Objectives**: To evaluate local control outcomes of liver metastases treated with proton stereotactic body radiotherapy (SBRT). Secondary objectives include evaluation of toxicity.

**Materials/Methods**: Patients diagnosed with metastatic disease to the liver treated with proton SBRT between 2012 and 2017 were included. Tumor motion was accounted for using either respiratory management or 4D CT scan.

In-field local control was evaluated according to RECIST 1.1 criterion. Radiation induced liver disease was evaluated using CTCAE 5.0 criterion.

**Results**: A total of 41 patients with 80 lesions were included in this analysis with a median age of 65.5 years (range 33-87). Dose/fractionation included 50GyE / 5fx (19%), 30GyE / 5fx (16%), and 30GyE / 3fx (16%). The primary cancer site was 51% colorectal, 17% breast, and 37% other. The number of metastases treated ranged from 1-5 with mean tumor diameter of 2.7 cm ± 1.6 cm (range 0.7 – 7.3).

With a mean follow up of 14.5 months (range 1 – 44), local control at 6 months and 1 year was 92% and 75%, respectively. Grade 1 and grade 2 toxicities were 38% and 6% respectively. No patient developed grade 3 or higher toxicity. No radiation induced liver disease was detected.

**Conclusions**: Proton SBRT for liver metastases demonstrated a high rate of local tumor control with minimal toxicity. An ongoing institutional phase II trial to further validate current findings will also be updated at time of presentation.

### O 042: Innovative Strip Silicon Detectors for Proton Beam Monitoring: Preliminary Results

#### A. Vignati^1^, O. Hammad Ali^2^, A. Attili^1^, M. Donetti^3^, F. Fausti^1^, S. Giordanengo^1^, L. Manganaro^2^, V. Monaco^2^, R. Sacchi^2^, R. Cirio^2^

##### *^1^National Institute for Nuclear Physics INFN, Turin division, Turin, Italy*, *^2^National Institute for Nuclear Physics INFN- Università degli Studi di Torino, Physics Department, Turin, Italy*, *^3^Fondazione CNAO, Medical Physics, Pavia, Italy*

Unlike the legacy gas ionization chambers, solid state detectors offer large granularity and sensitivity to single protons and would ideally be suited for beam monitoring in therapy applications. However, signal pileup, radiation damage and the readout complexity prevented their use so far on high flux therapeutic beams.

Innovative silicon low-gain avalanche detectors optimized for time resolution (Ultra Fast Silicon Detectors - UFSDs) were recently proposed, where sensors as thin as 50 μm provide signals of ∼ 1ns time duration with time resolutions of tenths of ps, and large enough signal-to-noise ratio to efficiently discriminate the proton signal.

The MoVeIT project of the INFN is investigating the use of this technology in Particle Therapy by developing two monitoring devices, one to directly count individual protons at high rates, the second to measure the beam energy with time-of-flight techniques. This requires the design of custom UFSD sensors as well VLSI readout electronics.

From the simulations' results and the first beam tests with UFSD pads, strip detectors were produced by FBK in Trento, with two geometries (30 mm and 15 mm length) and different doping modalities to improve radiation hardness. In parallel, prototypes of a new TERA10 readout chip have been submitted to the foundry.

The aim of this contribution is to review the advancement of the project and to report on the results of the tests of UFSD strip sensors with the therapeutic proton beam of CNAO.

### O 043: Dynamic Beam Current Control for Improved Dose Accuracy in PBS Proton Therapy

#### C. Bula^1^, M. Eichin^1^, J. Hrbacek^1^, M.F. Belosi^1^, D. Meer^1^

##### ^1^Paul Scherrer Institut, Center for Proton Therapy, Villigen, Switzerland

The step-and-shoot method of pencil beam scanning applies the dose on a three-dimensional grid in the target volume, with one dimension defined by the proton energy. While the spot dose may vary substantially within an iso-energy layer, the beam current typically remains constant. In this static operation mode, the inherent latency of the beam switch-off mechanism results in a lower limit for the deliverable spot dose, which may conflict with part of the low-weighted spots prescribed by the treatment planning system.

To overcome this limitation, we enhanced the control system of the PSI Gantry 2 with a direct link to the vertical deflector located at the center of the cyclotron. This connection allows much faster beam current changes (∼ 0.1 ms) and hence opens the possibility of temporarily reducing the current for individual low-dose spots.

This new dynamic operation mode improved the accuracy of the delivered dose compared to the planned distribution without compromising treatment time. Figure 1 shows an example field where 4% of the spots (0.4% of dose) were skipped in the static mode, while the dynamic mode allowed to deliver all spots and reduced the maximum missing dose per voxel from 2.3% to 1.3%. The method was successfully commissioned and is in clinical operation since fall 2017.

We consider dynamic beam current control to be a valuable contribution to cyclotron-based spot scanning technology, especially in the context of new modalities such as rescanning and high-intensity deliveries, where the number of low-weighted spots is even more pronounced.

### O 044: Commissioning of a Unique Penumbra Sharpening Adaptive Aperture of HYPERSCAN

#### M. Kang^1^, H. Chen^1^, R. Cessac^2^, D. Pang^1^

##### *^1^Georgetown University Hospital, Radiation Medicine, Washington DC, USA*, *^2^Mevion Medical System, Engineering, Littleton- MA, USA*

**Purpose:** To present the commissioning of a unique adaptive aperture(AA) on the penumbra sharpening for proton pencil beam scanning treatment.

**Materials and Methods**: The AA is essentially a mini-MLC consisting of 7 pairs of leafs made of nickel. The inner 5 and the outer 2 pairs have width of 0.5 and 2 cm, and the thickness of the leaves in the beam direction is 10 cm(Fig1(a)). The leaves follow the scanning spots and trim the spots on the periphery of a field to sharpen the penumbra. Both single spot and uniform spot maps of square fields were created with and without AA for typical therapeutic energies. A PTW Octavius ion chamber array was used to measure the absolute dose and field profiles at the Isocenter plane.

**Results and Discussion**: Fig1 (b) shows the ratio of the absolute dose between with and without AA can be 5% different, which is energy, field size and nozzle position dependent. Fig 2(a)-(c) show that the penumbra and dose reduction by using AA for single spots. AA can reduce the penumbra by 2 mm to 12 mm from highest energy 227 to 64MeV for square fields. The significant penumbra reduction for low energy beams make it feasible to use very low energy scanning proton to achieve conformal dose distribution without using any external range shifter. However, the Raystation TPS currently cannot model the actual MLC motion patterns in planning so the final delivery MUs should be verified from measurement and adjusted by using appropriate correction factors.

### O 045: External Beam Radiotherapy for Ocular Melanoma: A Comparative Study Low Energy Protons vs. Pencil Scanning High Energy Protons vs. Cyberknife

#### A. Gerard^1^, M.L. Peyrichon^1^, C. Peucelle^1^, M. Vidal^1^, A. Claren^1^, S. Wolfgang^2^, A. Carnicer^1^, G. Angellier^1^, J. Thariat^3^, J. Herault^1^

##### *^1^Centre Antoine Lacassagne, Department of Radiation Oncology, Nice, France*, *^2^Strahlenklinik- University Hospital, Department of Radiation Oncology, Essen, Germany*, *^3^Centre François-Baclesse, Department of Radiation Oncology, Caen, France*

The Centre Antoine Lacassagne (CAL) hosts all three facilities potentially competitive for radiotherapy of ocular tumors. Therefore, CAL can identify the advantages and disadvantages of each type of facility.

Based mainly on radiochromic films irradiation, the study analyzes dosimetric data for both proton facilities and Cyberknife using respectively one and multiple beam incidences. Two clinical cases were considered: a conjunctival melanoma and a posterior uveal melanoma. The contributions of beam modifiers as well as dosimetric planning data were analyzed. The doses from primary and secondary particles received by distant organs were studied. Beyond static image views, the influences of eye positioning systems were also analyzed. Always related to positioning, the irradiation duration, the tracking system as well as eyelids sparing were compared. The study is carried out disregarding clinical results as well as economic imperatives and is based solely on dosimetric data and positioning quality.

Based on dosimetric and positioning criteria, stereotactic photon irradiation delivered for example by Cyberknife is not suitable for the treatment of ocular malignancies. Irradiation with proton beams leads to a more favorable dose distribution. The use of a high-energy proton pencil beam scanning however requires additional collimator and positioning accessory equipment and fails to compete with both distal (6.7 vs. 0.6 mm) and lateral (8.7 vs. 1.6 mm) 20-80% penumbras of a low energy proton beam. In addition, its prohibitive treatment duration for 15 GyEBR (5 min vs. 10 s) jeopardizes an effective movement limitation resulting larger irradiated volumes and higher doses to OARs.

### O 046: Utilization of an Isocentric Rotating Chair for Treating Head/Neck Cancer at Seating in Fixed Particle Beamline

#### W.C. Hsi^1^, R. Zhou^2^, Z. Wang^2^, F. Yang^2^, X. Zhang^2^, J. Sun^1^, S. Yinxian^1^, H. Xue^3^, M. Wang^3^, F. Liu^3^

##### *^1^Shanghai Proton and Heavy Ion Center, Medical Physics, Shanghhai, China*, *^2^Sichunan University, College of physical science and technology, Chengdu, China*, *^3^Jiangsu Supersense Technology Co. LTD, Technology, Suzhou, China*

**Purpose:** Present systematic evaluation of chair with six-degree-freedom (6DoF) isocentric movement for head/neck cancers at seating positioning in fixed carbon-ion and proton beamline.

**Methods and Materials:** When patients are well re-positioned at chair, a movement around isocenter required only rotation along vertical axis with a translation in IEC fixed coordinate; referred beam-angle vector. Any re-positioning error required additional 6 DoF displacement on IEC patient-support coordinate; referred as a correction vector. A patient-support coordinate is rotated accordingly to required beam angle. In Fig. 1 for built chair, mechanisms of a 360rotating platform for beam-angle movement, and a six-pod 6DoF movement for correction vector was utilized. The mathematical presentation for combined beam-angle movement and correction vector was evaluated to be coincided with mathematical formula used in X-ray based image-guiding positioning system during acceptance tests.

**Results:** In Fig. 2, a 4x4 matrix to execute a defined sequence rotation (3x3 matrix) of 3 axes followed by a translation vector (3x1 vector) was found to a unique solution for any 6 DoF movement in fixed coordinate. This 4x4 matrix was also proved to equivalently present a translation following by a sequence rotation around 3-rotated axes in patient-support coordinate. Any movement combined with beam-angle movement and correction vector could be presented by six-parameter set in fixed coordinate. However, accuracy of immobilization was evaluated by extracted six-parameter set for correction vector.

**Conclusions:** With understanding of mathematical characteristics of beam-angle movement and correction vector in separated mechanisms used in chair, treated patients can be correctly positioned.

### O 047: Clinical Implementation of the Patient-Specific Apertures Used in Commercial Proton Pencil Beam Scanning Systems

#### C. Chen^1^, H. Liu^1^, S. Luckman^1^, E. Readdy^1^, K. Hall^1^, Z. Han^1^, N. Ju^1^, N. Schreuder^2^, D. Mah^1^

##### ^1^ProCure Proton Therapy Center, Medical Physics, Somerset, USA, *^2^ProVision Center for Proton Therapy, Medical Physics, Knoxville, USA*

**Purpose**: Patient-specific apertures for proton pencil beam scanning (PBS) were commissioned using commercially available beam delivery and treatment planning systems (TPS). We performed: 1. collinearity tests amongst the X-ray imaging, spot steering and the aperture mount; 2. dose verification of the Monte Carlo algorithm including aperture transmission and scatter; 3. refinement of the patient-specific QA procedure to verify overall implementation.

**Materials and Methods:** The gantry-dependent spot steering was tested using star-shot films. A uniform PBS field with a single centralized spot was generated and over-scanned on a square aperture (Fig. 1) to measure the isocenter offsets in between the PBS spot positioning and aperture center. The measured depth-doses and lateral profiles of aperture-shaped fields were compared with TPS calculations. Finally, clinical PBS aperture-collimated plans were generated and patient-specific QA procedure was refined to account for the larger ion chamber spacing on the 2D array.

**Results**: The star-shot diameters were 0.14 mm and 0.34 mm for gantry rotated clockwise and counterclockwise respectively (Fig. 2). The offsets in between the spot positioning and aperture center were <1 mm. Acceptable dose agreements were found in the dose comparisons for the aperture-shaped fields. It was noticed that the TPS created heavily weighted spots abutting the aperture edge to ensure coverage.

**Conclusions**: The use of PBS aperture-shaped fields reduces the penumbra significantly at shallow depths permitting sparing of OARs for selected cases. The alignment in between the spot positioning and aperture shaping should be carefully verified to ensure accurate delivery.

### O 048: A Novel Deliver Sequence and Efficiency Optimization Algorithm for Spot-Scanning Proton Arc Therapy

#### X. Ding^1^, X. Li^1^, J. Zhou^1^, C. Stevens^2^, Y. Di^2^, P. Kabolizadeh^1^

##### ^1^Beaumont Health, Radiation Oncology Proton Therapy Center, Royal Oak, USA, ^2^Beaumont Health, Radiation Oncology, Royal Oak, USA

**Purpose**: Spot-Scanning Proton Arc therapy (SPArc) has been a great interest of the society because of the improved dosimetric outcome. However, there are a lot of technique challenges to implement it in the clinical settings especially the delivery efficiency. Due to the hysteresis and technical difficulties in changing the beamline magnetic field, it costs significant time in the energy layer switching especially switching from low to high energy. Thus, we presented a new energy sequence optimized SPArc algorithm(SPArc_seq) to shorten the delivery time of the SPArc.

**Material and Methods**: SPArc_seq includes an energy layer sorting and control point re-sampling mechanism taking into account of delivery sequence through the gantry rotation. It is optimized for high to low energy delivery sequence instead of random layer switching. Both SPArc and new SPArc_seq were tested on 5 prostate patients. Both plans were delivered at a fixed 0 degree gantry angle. Total actual delivery time was recorded and dose measurements were performed using MatriXXONE at 3cm depth.

**Results**: The result showed that with similar plan quality, SPArc-seq (mean beam-on-time: 330 seconds) was able to successfully reduce about 56% of delivery time compared to SPArc (mean beam-on-time: 756 seconds) (p<0.01). Absolute dose measurements showed within 2% difference compared to the plans. 2D Gamma Index(3%/3mm) showed more than 97% passing rate in both SPArc and SPArc_seq.

**Conclusion**: This is the first study to test the SPArc delivery feasibility at a fixed gantry angle. SPArc_seq algorithm could effectively reduce the total treatment delivery time while keeping the similar plan quality.

### O 049: Superconducting Gantry for Proton Therapy with a Large Momentum Acceptance

#### K.P. Nesteruk^1^, C. Calzolaio^1^, A. Gerbershagen^2^, D. Meer^1^, V. Rizzoglio^1^, M. Seidel^1^, J.M. Schippers1

##### 1Paul Scherrer Institut, Large Research Facilities GFA, Villigen, Switzerland, 2CERN, Engineering Department, Geneva, Switzerland

Utilization of superconductivity for proton therapy gantries allows them to be much lighter and somewhat smaller. These features bring obvious advantages, especially for new hospital-based facilities. In our design we have included the degrader into the gantry. Since such a gantry will not need an external degrader and energy selection system, a substantial footprint reduction can be achieved in new cyclotron-based facilities. In addition to this, superconducting magnets deployed for a gantry can also significantly improve the treatment process. For our development of a new SC gantry, we have designed specific beam optics with a very large momentum acceptance, enabled by using superconductivity. This achievement allows a wide energy spectrum to be transported so that a typical depth range in the order of +/- 30% can be covered without changing the magnetic field of these magnets. Such a property enables a decrease of the treatment time. In particular, the large momentum acceptance combined with a ridge-filter instead of a degrader, would give a possibility of using multiple energies in the gantry at the same time, and consequently, would allow an SOBP to be delivered at once. Moreover, with the potentially achievable treatment time in the order of a few seconds, various new techniques can be employed to deal with organ motion.

### O 051: 4D Robust Optimization Including Time Structures Can Reduce the Interplay Effect in Proton Pencil Beam Scanning Radiotherapy

#### E. Engwall^1^, A. Fredriksson^2^, L. Glimelius^1^

##### ^1^RaySearch Laboratories AB, Physics, Stockholm, Sweden, ^2^RaySearch Laboratories AB, Research, Stockholm, Sweden

Interference between the delivery time structure and organ motion can create large distortions in the dose distributions of proton pencil beam scanning radiation therapy, and is known as the interplay effect. Standard methods for mitigating this effect include abdominal compression, rescanning and gating. We propose a new method (implemented in a research version of the RayStation treatment planning system), where the time structures of the delivery and the organ motion are included in a 4D robust optimization. The time structures are used to distribute the pencil beams over the different breathing phases and partial beam doses delivered to each phase are calculated. The partial beam doses are accumulated on a reference phase using deformable image registration. Uncertainties in the delivery and organ motion are handled by the inclusion of multiple scenarios in the robust optimization implemented by means of minimax optimization. In this study, we limit the uncertainties to variations in the breathing pattern.

The method is evaluated for three different non-small cell lung cancer patients with different motion amplitudes. The ability of the method to reduce dose distortions from interplay is compared to results from standard 4D robustly optimized plans with and without rescanning (see Figure 1). Our study shows that the new method is beneficial, especially for large tumor motion, where rescanning alone cannot mitigate the interplay effect. The most efficient mitigation is achieved when our method is combined with rescanning.

### O 052: Robust Treatment Planning with 4D Intensity Modulated Particle Therapy for Multiple Targets in Stage IV Non-Small Cell Lung Cancer

#### M. Wolf^1^, C. Graeff^1^, K. Anderle^1^

##### ^1^GSI Helmholtz Centre for Heavy Ion Research, Biophysics, Darmstadt, Germany

**Purpose**: Recently, we introduced a 4D IMPT optimization approach handling multiple targets and including all motion states of a 4DCT. This method was expanded by a robust non-linear biological optimizer for carbon ions – accounting for setup and range uncertainties – to explore its potential in improving plan robustness and sparing of critical organs.

**Methods**: The implemented worst case scenario method considers 9 different scenarios: nominal scenario, under- and overestimation of particle ranges (±3.5%) and isotropic shifts of patient's isocenter (±3 mm in the 6 major anatomical directions). Within the 4D IMPT optimization approach, robust IMPT on CTV is compared to conventional IMPT with 3 mm margins using 4D dose calculation and robustness analysis with 21 different uncertainty cases. Rescanning is used for motion mitigation.

**Results**: Both optimization methods are compared in two lung cancer cases with multiple lesions in proximity to critical structures, like smaller airways (SA). By using robust IMPT the given constraints for SA could be fulfilled in 98.4% of all regarded cases compared to just 55.5% with conventional IMPT. This was enabled by reducing average target coverage (see D99 values in Table 1) which decreased the D0.5cc for SA from 56.8±4.1% to 46.6±2.4% averaged over all cases. Additionally, the uncertainty bands in the DVHs could be reduced as well (Fig.1).

**Conclusion**: The study above showed that plan robustness could be improved by using robust optimization in 4D IMPT. Focusing on improved OAR sparing implicates a decrease in target coverage in the majority of the regarded uncertainty scenarios.

### O 053: Evaluation of 4D Plan Robustness Using Lung 4DCT(MRI)

#### M. Krieger^1,2^, J. Käser^2^, R.L. Perrin^1^, M. Peroni^1^, O. Bieri^3,4^, Z. Celicanin^3,4^, D.C. Weber^1,5^, A.J. Lomax^1,2^, Y. Zhang^1^

##### ^1^Paul Scherrer Institute, Center for Proton Therapy, Villigen PSI, Switzerland, ^2^ETH Zürich, Department of Physics, Zürich, Switzerland, ^3^University of Basel Hospital, Department of Radiology- Division of Radiological Physics, Basel, Switzerland, ^4^University of Basel, Department of Biomedical Engineering, Basel, Switzerland, ^5^University Hospital Zürich, Department of Radiology, Zürich, Switzerland

**Purpose**: 4D planning for lung cancer is typically performed using a single 4DCT. Here we demonstrate the potential variability in 4D dose-distributions for PBS lung treatments under variable breathing conditions by using the 4DCT(MRI) approach applied to lung cancer cases.

**Materials**: Lung motion deformation fields from 10 breathing cycles have been extracted from 4DMRI datasets of 2 volunteers. From these, 10 synthetic 4DCT(MRI) datasets, one for each extracted breathing cycle, were generated for each motion scenario by deforming the end-exhale phase of a patient 4DCT with the extracted 4DMRI motion fields. A 2Gy(RBE), 3-field PBS plan was optimised to the geometric ITV on the static CT, and 4D dose calculations (4DDCs) were performed on the different 4DCT(MRI) datasets. For comparison, 4DDCs were also performed assuming perfectly periodic motion by repeating the first breathing cycle of each 4DCT(MRI) in the 4DDC.

**Results**: Figure 1 shows the median SI motion of the lung for the two volunteers, comparing the repeated motion to the actual (variable) motion, with the differences in the resulting 4D dose-distributions shown in Figure 2. Dose differences in the ITV of close to +/-20% (mean absolute difference (SD): 3(9)%) are observed between periodic and variable motions from the same volunteer (Figure 2b), and of up to 50% (mean absolute difference (SD): 20(40)%) between the two different motion scenarios (Figure 2c and d).

**Conclusion**: Calculating a lung plan under different motion scenarios is important to capture the range of possible effects of breathing. 4DCT(MRI) could be a valuable tool for this purpose.

### O 054: Alternating Intra-Field Scan Direction in Rescanning for Improved Motion Mitigation

#### G. Fattori^1^, G. Klimpki^1^, Y. Zhang^1^, M. Krieger^1^, J. Hrbacek^1^, D.C. Weber^1^, A. Lomax^1^, S. Safai^1^

##### ^1^Paul Scherrer Institute, Center for Proton Therapy, Villigen, Switzerland

For moderate organ motion, rescanning is effective in averaging dose distortions due to interplay. However, unsought positional and temporal correlations between patient breathing and the dynamics of rescanning may arise, undermining its efficacy. Here we investigate the effectiveness of systematically changing meander direction within the field to increase interplay mitigation due to rescanning. Alternation of the meander path for rescanning can be performed by either switching the primary direction of scanning between each energy layer or between each volumetric rescan, both of which have been experimentally investigated.

Using a platform-mounted ionisation chamber array, measured doses were compared to those of a stationary delivery. The detector was moved to replicate a cranio-caudal target displacement (ca. 6 mm) of a liver carcinoma patient (PTV 76.59 cm3), and the conventionally generated control files modified to scan either parallel or orthogonally to the motion, or to alternate between energy layers (EE) or between each rescan (ER).

Results from central plane measurements demonstrate that, to achieve a high gamma pass rate (∼90% at 1%/1mm), a substantially smaller number of rescans was required when using ER (4x) compared to best-case conventional (non-alternating) rescanning (8x), and that ER was marginally more effective than EE. When introducing additional random amplitude and breathing fluctuations however, agreement was compromised for all scenarios, but was still consistently higher for the EE and ER scenarios (87.2%/95.7% pass rates for 8x) than conventional rescanning (best case 71.7% for parallel re-scanning). In conclusion, alternating scanning directions during re-scanning can further help mitigate interplay effect.

### O 055: Feasibility of Noninvasive Cardiac Ablation Utilizing Intensity Modulated Proton Therapy to Treat Ventricular Tachycardia

#### S.M. Goddu^1^, J. Hilliard^1^, N. Knutson^1^, T. Zhao^1^, P. Samson^1^, G. Hugo^1^, S. Mutic^1^, J. Bradley^1^, P. Cuculich^2^, C. Robinson^1^

##### ^1^Washington University, Radiation Oncology, Saint Louis, USA, ^2^Washington University, Cardio-vascular Division, Saint Louis, USA

**Introduction**: We have recently demonstrated safety and feasibility (Cuculich et al, NEJM-2017) of using noninvasive single-fraction photon-based-SBRT to treat patients with refractory, life threatening, ventricular-tachycardia (VT). Proton Therapy has the potential to reduce dose to non-target-heart-tissue (NT-HT). We evaluated the feasibility and potential dosimetric improvements using proton-beam-therapy for noninvasive treatment of VT.

**Method**: Sixteen patients, who underwent single-fraction VMAT-SBRT (3-5 arcs) to arrhythmogenic focus on a prospective phase I/II trial, were retrospectively re-planned using Varian's ProBeam-IMPT and compared against their respective clinical plans. Four non-coplanar-beams were selected by minimizing the irradiated volumes of the surrounding NT-HT, stomach, esophagus and bowel while maximizing plan robustness. A single-fraction dose of 35Gy to ITV and 25Gy to PTV were prescribed. Varian pencil-beam model in Eclipse Treatment-Planning-System was used to optimize the plans.

**Results**: Typical isodose distributions and dose-volume-histograms are shown in Figure-1. Esophagus and/or stomach were adjacent to PTVs in 25% of patients, which limited the PTV coverage. However, all IMPT plans showed adequate target coverage (V95%Rx=>99%) while meeting the OAR objectives/constraints. Although conformity-index was similar, excellent NT-HT sparing and reduction in V50%Rx was observed in IMPT plans. NT-HT volume reduction at different dose-levels is shown in Table-1.

**Conclusion**: The advantage of distal dose-fall-off by protons is highly promising for this group of patients where cardiac toxicity may play a vital role in long-term survivors. However, delivery accuracy in the presence of tissue-motion should be tested before clinical use. Furthermore, this may open doors to a new set of patients otherwise treated using catheter ablation.

### O 056: Plan Analysis Tools to Quantify Proton IMPT Complexity

#### A. Gosling^1^, A. Warry^1^, C. Gillies^1^, V. Rompokos^1^, A. Poynter1, D. D'Souza^1^

##### ^1^University College London Hospitals, Radiotherapy Physics, London, United Kingdom

QA tasks take up a significant amount of time for any proton therapy centre. The United Kingdom's National Health Service (NHS) model for a standard treatment day at the new proton therapy centres in London and Manchester allocate 12.5% of the total available treatment time to patient specific QA. However, there are currently no agreed upon metrics that measure the complexity of a proton plan to offer an indication of its likelihood of passing such QA.

In preparation for the opening of the NHS proton therapy centre at University College London Hospitals (UCLH), we are developing a suite of tools to extract and analyse data from DICOM ION plan files. These tools will generate metrics to quantify the complexity of plans, and display these in easily interpreted plots; thus allowing planners to determine plan modulation levels and quantitatively compare plans designed using different techniques.

We generate metrics and plots based on the MUs in beams and individual spots, their change over the course of beam delivery, as well as the derivatives of these values. Once we begin treatment operation, we plan to correlate these metrics with the outcomes of patient plan QA performed using the beamline. From this we hope to determine threshold levels at which we will be confident of a plans passing QA. In the long term this may allow us to reduce the amount of patient specific QA performed and therefore improve patient throughput and working time efficiency.

### O 058: The Development of a Unique Co-Linearity Set-Up and Test Phantom for a Patient Positioner Mounted Image Guidance System

#### N. Schreuder^1^, D. Hu^2^, J. Shamblin^2^, J. Treffert^2^, D. Slater^2^, P. Bagwell^2^, L. Derenchuk^2^

##### ^1^Provision Cares Proton Therapy Center - Knoxville, Medical Physics, Knoxville, USA, ^2^ProNova Solutions, ProNova, Knoxville, USA

The ProNova SC360 system facilitates image guidance Radiotherapy (IGRT) with a cone-beam CT (CBCT) system mounted on the patient positioner. Classic IGRT systems acquire images independently from the patient positioner relative to the beam isocenter allowing a simple relationship between the beam and the IGRT system. A new method was required to test co-linearity between beam, PPS and IGRT systems for the SC360. A precision phantom containing four 2 mm diameter steel BBs rigidly mounted, with one at isocenter, and several precision laser tracker targets was developed. The isocenter BB is approximately aligned to the beam isocenter. The dose delivery system generates beam spots centered on the BBs. The BB shadows in the beam spots are clearly visible on EBT Gafchromic film or a scintillation detector placed behind the phantom (figure 1). The BB shadows relative to the center of the beam spots are digitally analyzed and the PPS adjusted accordingly to bring the isocenter BB precisely to the beam isocenter. The laser tracking system is used to determine the location of the isocenter BB in the room coordinate system. A CT scan of the phantom is used to create a treatment plan with beam spots centered on the BB's. The IGRT system generates a 3D CBCT image and uses automatic registration to generate all required corrections. The spot pattern is delivered and any deviations between the beam spot and the BBs indicates a calibration error between the PPS mounted IGRT system and beam and room coordinate systems.

### O 060: Utilizing Imaging Thresholds to Improve Setup Efficiency for Proton Therapy Patients

#### S. Petro^1^, B. Robison^1^, J. Renegar^1^, M. Blakey^1^, M. Artz^1^, N. Schreuder^1^

##### ^1^Provision CARES Proton Therapy Center, Medical Physics, Knoxville, TN, USA

Proton therapy relies on image guidance to position the patient, due to protons'sensitivity to changes in the treatment pathway. Typically, therapists align to “zero”, i.e. exact alignment between the DRR and acquired X-ray. This is rarely achievable, leading to increased imaging time and dose and reduced throughput. To improve imaging efficiency, we developed thresholds to provide therapists an objective measure of sufficient patient alignment based on the patient's robust plan evaluation.

We perform image guidance using pairs of pre-treatment orthogonal X-rays. Prior to using thresholds, each patient typically received 2-4 pairs of X-rays. To reduce imaging time and dose, we implemented thresholds. Each patient undergoes a robust evaluation by a physicist, who perturbs the plan with isocenter shifts. The perturbed shift is based on the patient's immobilization and imaging, e.g. boney anatomy or fiducials. Imaging thresholds are 2/3 of the perturbation. For example, a breast patient, immobilized with a VacLoc bag and aligned to external BBs, is shifted 5mm and rotated 3deg. The thresholds would then be 3mm in X, Y, and Z and 2deg of rotation. During pre-treatment imaging, therapists acquire an X-ray pair. If shifts are less than the thresholds, therapists are to move on to treatment without shifting or re-imaging.

Since the introduction of imaging thresholds, each patient typically receives only 1-2 pairs of X-rays and therapy reports improved efficiency.

Utilizing imaging thresholds based on each patient's individual setup has improved pre-treatment imaging efficiency and reduced imaging time and dose.

### O 061: Which Proton Imaging Setup Should We Use in a Proton Therapy Facility?

#### N. Krah^1^, F. Khellaf^1^, J.M. Létang^2^, S. Rit^1^, I. Rinaldi^3^

##### ^1^CNRS, Creatis, Villeurbanne, France, ^2^INSA Lyon, Creatis, Villeurbanne, France, ^3^CNRS, Ipnl, Villeurbanne, France

Proton imaging has been proposed to reduce range uncertainties in the treatment planning by circumventing or improving the conversion from Hounsfield Units to relative stopping power (RSP). In the last decades, different types of proton imaging setups have been developed, but not yet integrated into the clinical workflow.

In this contribution, we present a comprehensive comparison of four types of proton imaging setups. For this purpose, we develop a mathematical framework to quantify the spatial resolution achievable with each of them. We find that the spatial resolution in setups combining pencil beam scanning with X-ray flat panel detectors is 10% better than in setups with pixel-less detectors such as multilayer ionisation chambers. In both setup types, performance can be significantly improved by reducing the pencil beam size down to 2-3 mm FWHM. In this case, the achievable spatial resolution is only 50% lower than in considerably more complex single proton tracking setups. Our results show that imaging setups combining double scattering with a pixel detector provide sufficient spatial resolution only under very stringent conditions.

We further investigate the setups' integrability in a proton center and analyse their performance in the low dose regime based on characteristic detector sensitivities and image acquisition scenarios. Finally, we develop methods to extract physical properties of the imaged object complementary to the RSP and validate the results against experimental data.

Our results indicate that detector hardware already available in proton centers for quality assurance provide well performing proton imaging setups.

### O 062: Estimation of the Risk for Sequelae Following Radiosurgery of Liver Metastases

#### G. Mondlane^1^, A. Ureba1, M. Gubanski^2^, P. Lind^2,3^, A. Siegbahn^4^

##### ^1^Stockholm University, Department of Physics - Medical Radiation Physics, Stockholm, Sweden, ^2^Karolinska Institutet, Department of Oncology and Pathology, Stockholm, Sweden, ^3^Södersjukhuset, Department of Oncology, Stockholm, Sweden, ^4^Stockholm University, Department of Physics – Medical Radiation Physics, Stockholm, Sweden

**Purpose**: The aim of this study was to estimate the risk of side-effects following photon- and proton-beam based radiosurgery of liver metastases.

**Materials and Methods**: Ten liver metastases patients previously treated with photon-beam radiosurgery were selected. Intensity-modulated proton therapy (IMPT) plans were thereafter created by performing a CTV-based selective robust optimisation. Setup and range uncertainties were considered and a simultaneous PTV-based conventional optimisation was also performed. A robustness criterion was defined for the CTV (V95%>98% for at least 10 of the 12 simulated scenarios). The dosimetric values and the NTCPs obtained with the clinically used photon plans were compared with those obtained with IMPT. A generic proton RBE of 1.1 was assumed.

**Results**: For all patients, the robustness criterion was respected. Similar dose coverage of the PTV was obtained with the photon and proton plans. An improved dosimetric sparing of the healthy part of the liver, right kidney, lungs, spinal cord and the skin was achieved with IMPT. However, similar NTCP values (median = 0 %) were obtained for all the OARs, except for the healthy part of the liver, for which lower NTCPs were obtained with IMPT (Figure 1: NTCP values obtained for the healthy part of the liver with the photon plans and the IMPT plans).

**Conclusions**: Use of the selective robust optimisation approach in the proton radiosurgery planning carried out for liver metastases patients could improve the plan quality in terms of target-dose coverage, overall OAR sparing and reductions of the normal liver toxicity.

### O 063: Normal Tissue Complication Probability Models in Plan Evaluation of Children with Brain Tumours Referred to Proton Therapy

#### C. Stokkevag^1^, D.J. Indelicato^2^, H. Magelssen^3^, M.E. Evensen^3^, M. Ugland^1^, M. Brydoy^1^, T. Nordberg^1^, G.M. Engeseth^1^, P. Brandal^3^, L.P. Muren^4^

##### ^1^Haukeland University Hospital, Department of Oncology and Medical Physics, Bergen, Norway, ^2^University of Florida, Department of Radiation Oncology, Jacksonville, USA, ^3^Oslo University Hospital, Department of Oncology, Oslo, Norway, ^4^Aarhus University Hospital, Department of Medical Physics, Aarhus, Denmark

**Purpose**: Children with brain tumours are at particular risk of radiation-induced morbidity and are therefore routinely considered for proton therapy (PT) aiming at reducing the dose to healthy tissues. The aim of this study was to apply normal tissue complication probability (NTCP) models derived specifically for children when evaluating the gain of PT compared to contemporary photon-based radiotherapy (VMAT).

**Methods**: The study included sixteen patients (2-16 years) referred from two Norwegian institutions to PT abroad (2014-2016). All patients received cranial passive modulation PT, with CTV-dose prescriptions of 50.4-59.4 Gy(RBE). All cases were re-planned with VMAT to match target coverage of the delivered PT plan using original CT-images and structure sets. PT and VMAT plans were compared by relevant dose/volume metrics and NTCP for organs associated with growth hormone levels, auditory toxicity, visual impairment, and neurocognitive outcome.

**Results**: Low-dose volumes were considerably reduced with the delivered PT plans compared to VMAT re-plans, while the high-dose volumes were comparable (Fig.1). For low-to-intermediate risk levels for VMAT, PT risk was close to baseline, while for individual organs at intermediate-to-high dose levels, some cases favoured VMAT (Fig.2).

**Conclusions**: Most of the investigated parameters resulted in lower median values for the delivered PT plans compared to VMAT re-plans, with statistical significant differences for predicted audiometric toxicity. For normal tissue within and immediately adjacent to the target volume, NTCP estimates resulted in comparable elevated risk, independent of modality. This suggests that further toxicity reduction may depend on systematic de-escalation of the prescription dose in conjunction with technological advancement.

### O 064: Helical Tomotherapy Alone versus Helical Tomotherapy + Proton Beam Therapy Combination in Nasopharynx and Oropharynx Cancer: Comparison of Acute Toxicity

#### S.G. Park^1,2^, Y.C. Ahn^1^, D. Oh^1^, J.M. Noh^1^, C.S. Hong^1^, S.G. Ju^1^, D. Kwon^1^, K. Jo^1^, E. Chung^1^, W. Lee^1^

##### ^1^Samsung Medical Center- Sungkyunkwan University School of Medicine, Department of Radiation Oncology, Seoul, Korea Republic of, ^2^Keimyung University Dongsan Medical Center- Keimyung University School of Medicine, Department of Radiation Oncology, Daegu, Korea Republic of

In this contribution, we evaluate feasibility of combining helical Tomotherapy (HT) and proton beam therapy (PBT) in nasopharynx (NPC) and oropharynx (OPC) cancer.

From January 2016 till October 2017, 158 patients (90 NPC, 68 OPC) received definitive radiation therapy (92.4% with concurrent chemotherapy). Using simultaneous integrated boost and adaptive re-plan, 68.4 Gy to GTV and 36.0∼60.0 Gy to CTV were delivered in 30 fractions: initial 18 fractions by HT in all, and, after rival plan evaluation, later 12 fractions by HT in 111 (70.3%) or by PBT in 47 (29.7%). Acute toxicities and analgesic usage were evaluated.

Patients receiving HT/PBT presented more frequently with ipsilateral neck involvement and lower stage (p<0.001 and <0.001). With median 11 months' follow-up, locoregional and distant failures occurred in 7 (5.8%) and 12 (10.0%) without difference by technique or primary disease. Among all, grade ≥2 weight loss was less frequent in HT/PBT group (52.3% vs 27.7%, p=0.004). Among NPC patients, grade ≥2 mucositis was less frequent in HT/PBT group (69.4% vs 42.9%, p=0.017). Among OPC patients, grade ≥2 dermatitis was more frequent in HT/PBT group (18.4% vs 52.6%, p=0.005). Non-regular analgesic usage was more frequent in HT/PBT group (34.2% vs 48.9%, p=0.083), which was significant among NPC patients (45.2% vs 67.9%, p=0.046).

With very high disease control rates, HT/PBT combination, when compared with HT alone, was advantageous with respects to grade ≥2 weight loss in all, grade ≥2 mucositis and analgesic usage in NPC patients, but was disadvantageous in grade ≥2 dermatitis in OPC patients.

### O 065: Cost Comparison of Hepatocellular Carcinoma Treatment with Either Proton Radiotherapy or Transarterial Chemoembolization as Assessed from a Randomized Clinical Trial

#### D. Bush^1^, J. Smith^2^, M. Volk^3^, M. Reeves^4^, M. de Vera^5^

##### ^1^Loma Linda University Medical Center, Radiation Medicine, Loma Linda, USA, ^2^Loma Linda University Medical Center, Radiology, Loma Linda, USA, ^3^Loma Linda University Medical Center, Hepatology, Loma Linda, USA, ^4^Loma Linda University Medical Center, Surgical Oncology, Loma Linda, USA, ^5^Loma Linda University Medical Center, Transplant Institute, Loma Linda, USA

**Objective**: To determine the costs associated with treatment and post-treatment care for hepatocellular carcinoma (HCC) patients treated with either proton beam therapy (PT) or transarterial chemoembolization (TACE)

**Methods**: Eligible subjects had either clinical or pathologic diagnosis of HCC and met either Milan or San Francisco transplant criteria. Patients were randomly assigned to TACE or PT. Proton beam radiotherapy was delivered to all areas of gross disease to a total dose of 70.2 Gy in 15 daily fractions over 3 weeks. Treatment delivery costs of each treatment was determined by collecting billable CPT codes to calcutate medicare allowable charges. Post treatment costs were assessed by tabulating days of hospitalization within 30 days of treatment completion using medicare allowable charges associated with the admitting diagnostic code.

**Results**: At the time of this analysis 69 subjects were available for analysis. 36 were randomized to TACE and 33 to proton. The 36 TACE patients received 63 courses of TACE as primary HCC treatment, treatment of persistent disease, or for new HCC in other parts of the liver. The 33 PT patients received 38 courses of PT. (treatment costs to be presented at meeting). Total days of hospitalization within 30 days of TACE/proton was 166 and 24 days respectively (p<0.001). Costs of hospitalization following treatment was significantly less with PT which when combined with treatment delivery costs, made PT a less costly method of treatment.

**Conclusion**: This analysis indicates the overall cost PT is less than TACE potentially making it a more cost effective treatment for primary HCC.

### O 066: Stereotactic Proton Radiotherapy in the Treatment of Low and Intermediate Risk Prostate Cancer – 2-Year Results

#### J. Kubes^1^, S. Sláviková^1^, P. Vítek^1^, K. Dědečková^1^, V. Vondráček^1^, S. Vinakurau^1^, B. Ondrová^1^, A. Pasztorova^1^, G. Kasáčová^1^, J. Kvěch^1^

##### ^1^PTC Prague, Proton therapy dept., Prague, Czech Republic

**Purpose**: Stereotactic radiotherapy of prostate cancer is a common modality in photon therapy. Pencil beam scanning (PBS) in similar fractionation allows better dose distribution and makes proton therapy more available for such patients.

**Material and methods**: 200 patients with early stage prostate cancer were treated with IMPT (intensity modulated proton therapy), stereotactic schedule (36.25 GyE in 5 fractions) between February 2013 and December 2015. Mean age was 64,3 years, mean value of PSA before treatment was 6,83 μg/l (0,6-17,3 μg/l). 93 patients (46,5%) were in risk group 1, 107 patients (53,5%) were in risk group 2, 29 patients (14,5%) had neoadjuvant hormonal therapy, no patients had adjuvant hormonal therapy. Acute toxicity, early late toxicity and short term results were evaluated.

**Results**: All patients finished radiotherapy without interruptions. Median of follow up time is 25,7 months. Mean treatment time was 9.5 days (median 9 days). Acute toxicity (CTCAE-v.4) was: GI G1-17%, G2-3,5%, GU G1-40%,G2-19%, no G3 toxicity was observed. Late toxicity was: GI G1-19%, G2-5,5%; GU G1-17%,G2-4%, no G3 toxicity was observed. PSA relapse was observed in one patient (1,03%) from risk group 1 (pelvic lymph node involvement was detected) and in 7 patients (6,5%) from risk group 2 (3 lymph node metastasis, 2 lymph node and bone metastasis, 2 PSA relapses). No patient died from prostate cancer, 3 patients died from other reasons. No local recurrence in prostate was observed.

**Conclusion**: Proton beam radiotherapy of prostate cancer is feasible with low rate of acute toxicity and promising late toxicity and effectivity.

### O 067 - Prospective Patient-Reported Quality-of-Life Outcome of the Initial 100 Prostate Cancer Treated With the First Clinical Image-Guided Compact Pencil-Beam Proton Unit

#### J. Wang^1^, R. Guarisco^2^, P.D. Dang^1^, J.B. Wilkinson^3^, S. Katz^1^, M. Durci^1^, H.T. Wu^1^, L. Rosen^1^

##### ^1^Willis-Knighton Proton Center, Radiation Oncology, Shreveport, USA, ^2^LSU-Shreveport, School of Medicine, Shreveport, USA, ^3^Provision Proton Therapy Center, Radiation Oncology, Knoxville- TN, USA

**Introduction**: Although proton therapy for prostate cancer has evolved, quality-of-life data for patients treated with modern techniques remains limited.

**Materials/Methods**: Men with localized prostate cancer treated consecutively on a single-gantry compact pencil-beam scanning (PBS) proton unit (ProteusOne, IBA) between 2014 and 2017 were enrolled on an IRB-approved registry protocol. Patients' quality-of-life (QoL) questionnaires were prospectively collected at baseline and subsequent follow-ups. Patients without any follow-up or missing baseline assessment were excluded. Urinary symptoms and erectile dysfunction (ED) were assessed by the American Urological Association (AUA) Symptom Index and the Sexual Health Inventory For Men (SHIM) Score, respectively. Data was analyzed using two-tail Fisher's exact test comparing each time point to their baseline.

**Results**: Of the total of 113 patients, median age was 68.4 (47.6-89.8), PSA 5.9 (0.012-382), and Gleason score 7 (6–9). Low, intermediate, and high risk patients were 15.0%, 57.4%, and 32.7%, respectively. 36% received androgen deprivation therapy. Median follow-up was 9 months. At baseline, 58.0%, 34.5%, and 7.6% had mild, moderate, and severe urinary symptoms. Most patients with initial moderate or severe urinary symptoms reported improvement at their last follow up (88% and 100%, respectively). Approximately half (52%) of patients reported being sexually active at baseline, with no changes in percentage of patients experiencing moderate-to-severe ED symptoms (15-36%, p=0.2872-1.000) at any given time point.

**Conclusion**: Modern PBS proton radiotherapy possibly improves urinary symptoms and preserves sexual QoL in patients with localized prostate cancer. Long-term follow up is needed to confirm these favorable outcomes.

### O 068: Peripheral Lymphocyte Subpopulation Variation and Hematologic Changes after Carbon Ion Radiotherapy in Patients with Prostate Cancer

#### Z. Yang^1^, Z. Ning^1^, Z. Qing^1^, F. Shen^1^

##### ^1^Fudan University Shanghai Cancer Center FUSCC/Shanghai Proton and Heavy Ion Center SPHIC, Radiation Oncology Dept, Shanghai, China

**Purpose**: We aimed to assess peripheral lymphocyte subpopulations variations and hematologic changes after carbon ion radiotherapy (CIR) in patients with prostate cancer for 3 years.

**Experimental Design**: Eligible patients who had not undergone previous radiotherapy, were pathologically confirmed localized prostate adenocarcinoma. CIR was administered in daily fractions of 2.74GyE with a total dose of 63-66 GyE. Variations in lymphocyte subset counts were investigated pre-radiotherapy, during radiotherapy, post-radiotherapy, 1 month after CIR, and 3-year follow-up. Erythrocyte and platelet levels were evaluated pre-radiotherapy, during radiotherapy, post-radiotherapy, and in 6-month, 12-month, 24-month, and 36-month follow-up. CIR-related parameters were calculated using the Syngo system.

**Results**: An increase in CD8+ was observed in 3-year follow-up(P<0.01) while CD8+ remained stable during radiotherapy; (P<0.05 for both). An decrease in CD4+ (P<0.01) and the CD4/CD8 ratio(P<0.01) was observed in 3-year follow-up while increase in the CD4+ and CD4/CD8 ratio during treatment during treatment and 1-month follow-up (P<0.05 for both). NK remained stable during radiotherapy and follow-up; CD19+ gradually decreased during radiotherapy (P<0.01) but increased in 1-month follow-up, then remained stable.

Erythrocyte, leukocyte and platelet levels decreased during radiotherapy and in 3rd year (P<0.05), while they recovered to the pre-treatment level after completing CIR, then gradually increased during 2nd year of follow-up.

**Conclusion**: Prostate CIR has a small but significant effect on the blood count and peripheral lymphocyte subpopulations in different ways. Endocrine therapy might be related to peripheral lymphocyte subsets variation after 3 years of CIR.

### O 069: Minimal Acute Toxicity in Major Salivary Gland Patients after Proton Beam Therapy: Outcomes from the Proton Collaborative Group REG001-09 Trial

#### M. Chuong^1^, W. Hartsell^2^, H. Tsai^3^, G. Larson^4^, S. Badiyan^5^, G. Laramore^6^, L. Rosen^7^, C. Vargas^8^

##### ^1^Miami Cancer Institute, Radiation Oncology, Miami, USA, ^2^Northwestern Medicine Chicago Proton Center, Radiation Oncology, Warrenville, USA, ^3^ProCure Proton Therapy Center - New Jersey, Radiation Oncology, Somerset, USA, 4ProCure Proton Therapy Center - Oklahoma City, Radiation Oncology, Oklahoma City, USA, ^5^Maryland Proton Treatment Center, Radiation Oncology, Baltimore, USA, ^6^University of Washington, Radiation Oncology, Seattle, USA, ^7^Willis-Knighton Cancer Center, Radiation Oncology, Shreveport, USA, ^8^Mayo Clinic, Radiation Oncology, Scottsdale, USA

**Background**: Proton therapy (PBT) reduces normal organ dose compared to intensity modulated radiation therapy (IMRT) for major salivary gland patients. It is not well described whether this dosimetric advantage is clinically meaningful.

**Methods**: We evaluated treatment parameters and acute toxicity outcomes of patients with major salivary gland cancers enrolled on the Proton Collaborative Group REG001-09 trial (NCT01255748).

**Results**: Of 717 head and neck trial patients, 105 were treated for parotid (N=90) or submandibular gland (N=15) tumors across 7 institutions from 2010-2017. Median age was 61 years. The most common histologies were mucoepidermoid and squamous cell carcinomas. Most were T1/T2 (54%), N0 (57%), and all were M0. Treatment prior to PBT included surgery alone (55%) or surgery followed by X-ray therapy (11%), most commonly 66 Gy in 33 fractions. Median PBT dose was 66.5 GyE (range 14.8-70.9) in 33 fractions (range 4-66); only 1 patient was prescribed less than 50 GyE. Uniform scanning (45%) was used more than pencil beam scanning (33%). Target volume details were not available. Chemotherapy was given concurrently with PBT to 20%. Median followup was 14.3 months (range 0.8-60.3). Acute grade 2 or higher nausea (1.5%), dysgeusia (4.8%), xerostomia (7.6%), mucositis (10.5%), and dysphagia (10.5%), were uncommon (Table 1).

**Conclusions**: These are the first prospective data to demonstrate that major salivary gland patients who receive PBT experience less acute grade 2 or higher toxicity than is expected from IMRT. Longer follow up is needed to more accurately characterize late toxicity outcomes.

### O 070: Boron Neutron Capture Therapy for Locally Recurrent Head and Neck Squamous Cell Carcinoma: A Retrospective Analysis of Dose Response

#### H. Koivunoro^1,2^, L. Kankaanranta^1^, A. Haapaniemi^3^, A. Mäkitie^3^, H. Joensuu^1^

##### ^1^University of Helsinki and Helsinki University Hospital, Department of Oncology, Helsinki, Finland, ^2^Neutron Therapeutics Finland Oy, Medical Physics, Helsinki, Finland, ^3^University of Helsinki and Helsinki University Hospital, Department of Otorhinolaryngology - Head and Neck Surgery, Helsinki, Finland

Boron neutron capture therapy (BNCT) is based on accumulation of a boron carrier in cancerous tissues followed by external neutron irradiation. Boron fission due to neutron capture releases high-LET α-particles and lithium-nuclei, which have a range <10 μm, depositing most of the absorbed dose in cancer. Tissue boron uptake can only be estimated, and therefore the delivered radiation dose remains uncertain. In this study we examined the relationship between the estimated tumor dose and treatment response.

Seventy-nine patients (49 men, 30 women; median age, 62 yrs), with inoperable, recurrent head and neck squamous cell carcinoma (rHNSCC) were treated with BNCT once or twice. Seventy-five patients (95%) received BNCT as salvage treatment after radiotherapy failure.

Median minimum tumor dose from BNCT was 22 Gy(W) and tumor volume 105 cm3. Twenty-five (36%) patients had a complete response (CR), 22 (32%) partial response, 17 (25%) stable disease for a median of 4.2 months (range, 2.2-19.2), and 5 (7%) progressed. The median survival was 10 months (range, 0.1-124) and time to local progression 10.8 months (range, 0.1-124). A small median tumor volume (53 cm3) was associated with achieving CR. A minimum tumor dose >30 Gy (W) was associated with achieving CR, more favorable survival and a longer time to local progression.

The tumor volume and the minimum tumor dose are prognostic factors for achieving CR and for survival in patients with rHNSCC. Accelerator-based neutron sources have recently became available for hospital installation, and will enable conducting of new prospective trials to confirm our findings.

### O 071: Comparison of Dose-Response of Acute Mucositis after C-ion RT for Patients Treated at NIRS and CNAO

#### J.E. Dale^1,2^, P. Fossati^3,4^, A. Hasegawa^4^, S. Molinelli^5^, A. Mairani^5,6^, O. Dahl^1,2^, N. Matsufuji^7^, T. Ohno^8^, T. Kamada^9^

##### ^1^Haukeland University Hospital, Department of Oncology and Medical Physics, Bergen, Norway, ^2^University of Bergen, Faculty of Medicine, Bergen, Norway, ^3^MedAustron, Carbon Ion Program, Wiener Neustadt, Austria, ^4^National Center for Oncological Hadrontherapy, Clinical radiotherapy unit, Pavia, Italy, ^5^National Center for Oncological Hadrontherapy, Medical physics unit, Pavia, Italy, ^6^Heidelberg Ion-Beam Center, Medical physics, Heidelberg, Germany, ^7^National Institute of Radiological Sciences, Department of Accelerator and Medical Physics, Chiba, Japan, ^8^Gunma University Graduate School of Medicine, Department of Radiation Oncology, Gunma, Japan, ^9^National Institute of Radiological Sciences, Research Center for Charged Particle Therapy, Chiba, Japan

**Objective**: To investigate to which extent institution-specific treatment factors other than the applied RBE-model (delivery technique, no. of fields per day, etc.) affect the dose-response of acute mucositis in patients treated with C-ion RT at NIRS and CNAO.

**Methods**: Palate was contoured as a surface structure of 5 mm thickness in 24 patients treated at CNAO and 27 patients treated at NIRS. For patients treated at CNAO, both the Local effect model (LEM) and NIRS clinical dose (NIRS clin) were applied to the physical dose by using the open-source TPS matRad. The Lyman-Kutcher-Burman (LKB) method was used to derive dose-response curves for CTCAE grade ≥ 3 palate mucositis for the groups: 1) NIRS patients with NIRS clin dose (NIRSNIRS clin), 2) CNAO patients with LEM dose (CNAOLEM) and 3) CNAO patients with NIRS clin dose (CNAONIRS clin).

**Results**: Parameters for the LKB-model and the corresponding dose-response curves are presented in table 1 and figure 1. Changing only the RBE-model (CNAOLEM vs. CNAONIRS clin) resulted in a steepening of the dose-response curve and a 10% decrease in TD50. In contrast here was a more pronounced steepening of the curve and a 36% decrease in TD50 when comparing the toxicity taking all institution-specific factors into account (CNAOLEM vs. NIRSNIRS clin).

**Conclusion**: Although this study is based in a small sample size, the results suggest that the different RBE-models only partially explain the observed difference in dose-response. These findings highlight the difficulties involved in comparing treatment outcome between different C-ion RT centers.

### O 072: Patient and Public Involvement in Design of a Phase III Trial Comparing IMPT versus IMRT in Low Risk Oropharyngeal Cancer

#### C. Hague^1^, B. Foran^2^, E. Hall^3^, R. Moule^4^, C. Nutting^5^, S. Parsons^6^, R. Prestwich^7^, N. Slevin^1^, C. West^8^, D. Thomson^1^

##### ^1^The Christie NHS Foundation Trust, Department of Head and Neck- Clinical Oncology, Manchester, United Kingdom, ^2^Weston Park Hospital, Clinical Oncology, Sheffield, United Kingdom, ^3^The Institute of Cancer Research, Clinical Trials and Statistics Unit, London, United Kingdom, ^4^The University College London Hospital, Clinical Oncology, London, United Kingdom, ^5^The Royal Marsden NHS Foundation Trust, Clinical Oncology, London, United Kingdom, ^6^Manchester University NHS Foundation Trust and The University of Manchester, Public Programmes Team, Manchester, United Kingdom, ^7^St James' University Hospital, Clinical Oncology, Leeds, United Kingdom, ^8^The University of Manchester- The Christie NHS Foundation Trust-, Division of Cancer Science- Manchester Academic Health Science Centre, Manchester, United Kingdom

**Objective**: The Christie Hospital, Manchester and University College London Hospital are developing the UK's high energy proton beam service due to open in August 2018. We report the results of public and patient involvement in the design of the first UK trial of Intensity Modulated Proton Therapy (IMPT) versus Intensity Modulated Radiotherapy (IMRT) for low risk oropharyngeal cancer.

**Material and Methods**: Focus groups were held in Manchester, Sheffield and Leeds to understand patients' views of proton beam therapy, randomisation within the trial, willingness to travel and stay in Manchester for treatment, trial design and endpoints. Fifteen consecutive patients previously treated by IMRT for low risk oropharyngeal cancer from each centre were invited. Focus groups consisted of discussions around the questions shown in Table 1. Information was recorded on laminates, questionnaires, audio recordings and by telephone/email contact with a sample of patients. Data were analysed using thematic analysis.

**Results**: Thirty three patients and eight relatives attended. Existing public knowledge of protons and the differences with IMRT was good. Beliefs about randomisation included disappointment if not receiving IMPT but willingness to participate to help future patients. Feedback on travelling and staying near the proton beam centre for treatment were generally positive, accepting the need for family and clinical support. The patient pathway and proposed trial outcomes were viewed favourably.

**Conclusion**: This was an important piece of work to understand people's perceptions on the proton trial which has helped input into the trial design. The trial grant application is in process and due to be submitted in the near future.

### O 073: Commissioning of the ProNova SC360 PBS for First Patient Treatment at the Provision CARES Proton Therapy Center

#### L. Derenchuk^1^, J. Shamblin^1^, R. Moore^1^, J. Volk^1^, H. Li^1^, A. Xia^1^, M. Heminway^1^, P. Osucha^1^, J. Matteo^2^

##### ^1^ProNova Solutions- LLC, Research and Development, Knoxville, USA, ^2^ProNova Solutions- LLC, ProNova, Knoxville, USA

The ProNova SC360 is a multi-room proton therapy system utilizing permanent and superconducting magnets to reduce cost and size. The SC360 fixed beam treatment room at the Provision CARES Proton Therapy Center in Knoxville has been calibrated to meet or exceed clinical performance of existing proton therapy systems. A novel and highly automated tool set has been developed incorporating a calibration phantom that links imaging, positioning, and beam isocenters. Daily QA tests verify the calibration as well as beam performance data. The SC360 span of ranges from 4 cm through 32 cm WET has been commissioned with spot position accuracy of +/- 0.5 mm throughout the full 25 cm x 25 cm scanning extent (Figure 1). Dose linearity is within +/- 1% down to 2.5 × 106 protons per spot (Figure 2a, 2b). Day-to-day reproducibility and inter-day dose reproducibility are demonstrated to be better than 0.5%. Scanning performance, positioning precision and positioning reproducibility are measured in daily QA and are demonstrated to be robust with respect to changes of the isochronous cyclotron performance (Figure 1c). Treatment delivery times for clinical prostate treatment plans are less than one minute. Daily automated data collection and processing can be completed efficiently and generates a summary report of data graphically displayed.

### O 074 - Implementation of Clinical Helium Beams at the Heidelberg Ion Therapy Centre

#### T. Haberer^1^, A. Mairani^2^, J. Debus^3^

##### ^1^Heidelberg Ion Therapy Center, Directorate, Heidelberg, Germany, ^2^Heidelberg Ion Therapy Centre, Biophysics, Heidelberg, Germany, ^3^Heidelberg University Hospital, Radio-Oncology, Heidelberg, Germany

About 4.500 patients were treated with Protons or Carbon ions at the Heidelberg Ion Therapy Centre (HIT) using the fully active 3D-dimensional intensity-controlled rasterscan technique [^1, 2^]. An optimized linac-synchrotron combination generates libraries of energy-, focus- and intensity-variable pencil-beams for the dose-delivering scanning systems at two horizontally-fixed beam lines and a scanning ion gantry.

The availability of low-LET and high-LET beams at HIT ranging from protons to oxygen under identical conditions optimally supports clinical trials aiming to clarify the question of which particle species is best suited for what indication. In order to pave the way for new therapy protocols HIT comprises a laboratory infrastructure and a dedicated research beam line offering low-LET proton and Helium beams and Carbon and Oxygen beams at the high-LET end.

Comprehensive pre-clinical studies [^3, 4^], i.e physical beam characterization, radiobiological experiments, biophysical modelling and treatment plan comparisons in realistic clinical settings, the evident potential of Helium ions for the use in particle therapy triggered an implementation project for this new modality at HIT.

This paper will report about the approach and status of HIT's Helium treatment project.

**References**:

^1^Th. Haberer et al., NIM A330, 296-305 (1993).

^2^Th. Haberer et al., Radiotherapy and Oncology, Vol. 73 (Supplem. 2), p186-190, (2004).

^3^A. Mairani et al., Phys. Med. Biol., 61/11, 4283-4299 (2016).

^4^T. Tessonier et al., Phys. Med. Biol., 62, 3958-3982 (2017).

### O 075: Commissioning of the First Mevion HYPERSCAN Proton Pencil Beam Scanning System

#### M. Kang^1^, H. Chen^1^, R. Cessac^2^, D. Pang^3^

##### ^1^Georgetown University Hospital, Radiation Medicine, Washington- DC, USA, ^2^Mevion Medical Systems, Engineering, Littleton- MA, USA, ^3^Georgetown University Hospital, Radiation Medicine, Washington, USA

The Mevion HYPERSCAN is the first and only single-room compact proton system with a superconducting synchrocyclotron mounted on a rotational gantry to deliver scanned proton beams. It eliminates the beam transport employed in all other proton systems and builds the scanning magnets, the beam monitoring dosimetry system, the Energy Selection and the spot trimming Adaptive Aperture into a nozzle of only 2-m in length from the synchrocyclotron exit to the nozzle exit window. Such a unique design was predicted to produce beams uncharacteristic of the traditional beam-line based proton systems. In this report, we present our experience of commissioning the first HYPERSCAN PBS proton system, discuss the mechanical and radiation beam accuracy as well as the unique beam characteristics such as the invariant Bragg peak widths and distal fall-off at all energies, the spot size and profile penumbra variation with energy and air gap with and without application of the Adaptive Aperture. We further present dosimetric plan examples to demonstrate the potential clinical implications of such beam characteristics.

### O 077: Clinical Integration of RayStation, ARIA, and adaPT for Pencil Beam Scanning Proton Therapy

#### S. Rana^1^, C. McKenzie^1^, J. Bennouna^1^, F. Escobar^2^, M. Montour^2^, L. Higgins^3^, A. Gutierrez^1^

##### ^1^Miami Cancer Institute, Radiation Oncology, Miami, USA, ^2^IBA, Information Technology, Miami, USA, ^3^Miami Cancer Institute, Information Technology, Miami, USA

**Purpose**: Our new proton center employs multi-vendor equipment and software including RayStation TPS (v6.1.1.2), Varian ARIA (v13.7), and beam delivery (adaPT-Deliver, v11.0.0) and imaging system (adaPT-Insight, v2.10b) from IBA. To our best knowledge, our center is the first one to be clinical with a unique combination of RayStation, ARIA, and adaPT. The purpose of this work is to highlight the importance of devising a comprehensive plan to validate the interface and connectivity among these systems.

**Methods**: The tests were conducted by a team of physicists and IT staff with input from vendors. The tests are grouped into: (I) RayStation→ARIA, (II) ARIA↔adaPT-Deliver↔ adaPT-Insight, (III) adaPT-Insight→ARIA. For each group, tests were developed mimicking different clinical situations to ensure the integrity of data transfer and stable connectivity among RayStation, ARIA, and adaPT.

**Results:**

1. RayStation→ARIA: A special filter was needed in RayStation to make the RT Plans importable in ARIA. A 2-step import was established.
2. ARIA↔adaPT-Deliver↔ adaPT-Insight: A number of items were identified in order to make the treatment plan deliverable through adaPT-Deliver. A procedure was established to resume treatment in the event of termination of adaPT-Deliver.
3. adaPT-Insight→ARIA: A number of imaging related parameters including changes in software configurations were identified to ensure a stable connection and data transfer (CBCT and kV-kV images) from adaPT-Insight to ARIA.

**Conclusion**: The integration of RayStation, ARIA, and adaPT was non-trivial with a number of in-house solutions to ensure safe and efficient integration among systems.

### O 078: Design and Dosimetric Characterization of 3D Printed Small Animal Immobilization and Collimator for Pencil Beam Scanning Proton Research Room

#### M. Batie^1^, E. Lee^2^, M. Sertorio^3^, R. Vatner^4^, A. Mascia^2^

##### ^1^Cincinnati Children's Hospital Medical Center, Clinical Engineering, Cincinnati, USA, ^2^University of Cincinnati Medical Center- Proton Therapy Center, Radiation Oncology, Cincinnati, USA, ^3^Cincinnati Children's Hospital Medical Center, Center for Blood Disease Institute, Cincinnati, USA, 4University of Cincinnati, Radiation Oncology, Cincinnati, USA

The Cincinnati Children's Hospital proton therapy research facility contains a 360-degree pencil beam scanning gantry with image guidance system, laboratory space and animal housing. In order to utilize this facility for small animals, an immobilization system and radiation collimation system is required. This study proposes and characterizes a solution. We designed and characterized an immobilization system (Figure 1) for mice and rats that allows for gas anesthesia and standard small animal workflow. The immobilization system is made out of ABS plastic, and manufactured using a 3D printer. The system also includes a clamping mechanism for targeting of flank tumors. Integrated into the immobilization is a tray, positioned approximately 2.0cm above the small animal, with options for a half-beam block brass collimator or a fixed aperture system. The system is indexed to the gantry tabletop, and is modular in that multiple animals may be easily irradiated sequentially. The device is compatible with modern imaging systems, and we find no significant artifact produced with kilovoltage imaging. Using a planning simulation CT, a CT of the system was imported to the treatment planning software and the half-beam block collimator was modeled. Both a pass-through and SOBP treatment plan was created using the half-beam block. The dose profile was further characterized using a 2D scintillator detector with native resolution of 0.5mm (see Figure 2). This immobilization system allows for reproducible and efficient irradiation of small animals for research purposes while also using modern radiotherapy techniques like 3D treatment planning and image guidance.

### O 079: A strategy to Minimize Commissioning Time for a Pencil-Beam-Scanning Proton System

#### W.C. Hsi^1^, L. Coutinho^1^, S. Rana^1^, J. Bennouna^1^, J. Yu^1^, A. Gutierrez^1^

##### ^1^Miami Cancer Institute, Medical Physics, Miami, USA

**Purpose**: To establish a strategy by combining pre-commissioned acceptance test (AT) /validation-&-verification (VnV) to commission a gantry-based pencil-beam-scanning (PBS) proton beamline within 6-8 weeks.

**Method**: Because a mature PBS system provides stable beam delivery at its pre-commissioning AT/VnV phase, tasks in AT/ VnV were jointly performed by engineers and physicists. Developed dedicated dosimeters were utilized to accurately and efficiently measurements. Therefore, we could efficiently focus routinely and patient-specific quality assurance (QA) during commissioning.

**Results**: Tasks of AT/VnV were prepared into four categories with corresponding reports in electronic Bugzilla (Bug) system (Fig. 1). System experts were on-site to assistant operations of beam delivery, imaging systems, and dosimeters during commissioning. Characteristics of spots at various angles were extracted from VnV ‘Bug' records in Fig. 2. Percentage depth doses were accurately and efficiently measured with a multi-element array with calculated ones of TPS. Similar accurate measurements of integrated depth doses and lateral profiles of single spot by dosimeters, including single large-area chamber in scanning water phantom and optical planer dosimeters. Proper protocols of cone-beam CT and kV patient imaging were also established with geometric and patient-like phantoms. Also, daily and patient-specific QA were also established with dedicated dosimeters and imaging devices.

**Conclusion**: The strategy of involving physicists in pre-commissioning AT/VnV phases with dedicated dosimeters and on-site expert collaboration enables one to successfully commission a PBS beamline efficiently. Shortages of this strategy such as to conduct critical tasks paralleled in AT/VnV phases were reviewed to improve this strategy.

### O 080: System Architecture and Design Principles for Cost Effective Design of a Compact Proton Therapy System

#### M. Jones^1^, T. Zwart^2^, S. Rosenthal^3^, M. Tajima^4^

##### ^1^Mevion Medical Systems, Engineering, Littleton, USA, ^2^Mevion Medical Systems, Advanced Development, Littleton, USA, ^3^Mevion Medical Systems, Clinical Systems, Littleton, USA, ^4^Mevion Medical Systems, Product Management, Littleton, USA

As the accessibility to Proton Therapy increases, growth is still limited by the perception of the higher initial capital and operational costs of this modality leading to higher patient costs, compared to other treatment modalities. The MEVION S250 Proton Therapy System was designed around specific design principles and metrics in order to reduce the capital and operational cost of proton therapy. Application of these design principles and metrics has lead to a novel system architecture that reduced capital and operating cost by eliminating system complexity, improving system reliability, minimizing system footprint, and reducing construction costs.

We present the design principles and metrics that guided the approach to the MEVION S250 system and their impact on design choices throughout the system architecture. Each subsystem is assessed for their impact on equipment cost, reliability, or construction cost. The inherent cost advantage of this architecture as compared with legacy approaches will be analyzed as a full system. Furthermore, we outline areas for potential further improvement and cost reduction.

### O 082: Proton Minibeam Radiation Therapy Widens the Therapeutic Window for Gliomas

#### Y. Prezado^1^, W. Gonzalez^1^, A. Patriarca^2^, G. Jouvion^3^, C. Guardiola^1^, C. Nauraye^2^, M. Juchaux^1^, L. Jourdain^4^, C. Sebrie^4^, F. Pouzoulet^5^

##### ^1^CNRS, Imagerie et modelisation pour la Neurobiologie et la Cancerologie, Orsay, France, ^2^Institut Curie, Orsay Proton Therapy Center, Orsay, France, ^3^Institut Pasteur, Human histopathology and animal models, Paris, France, ^4^Universite Paris Sud, Ir4m, Orsay, France, ^5^Institut Curie, Experimental Radiotherapy Platform, Orsay, France

The morbidity of normal tissues continues to be the main limitation in radiotherapy. To overcome it, we proposed a novel concept: proton minibeam radiation therapy (pMBRT) [^1^]. We have recently demonstrated that pMBRT leads to a significant increase of normal tissue tolerances [^2^] with respect to standard proton therapy. This opens the door to a dose escalation in the tumour. Along this line, we have performed a series of experiments where RG2 glioma bearing rats were irradiated. Four groups (n=10/each) were considered: i) non-irradiated controls; ii) a group receiving conventional proton therapy (PT), with a dose prescription of 25 Gy/one fraction iii) a group receiving pMBRT, with a homogeneous dose distribution in the tumour (25 Gy/one fraction); iv) and a group receiving pMBRT with heterogenous dose distribution (30 Gy mean dose). In all cases, the contralateral hemisphere was also irradiated to evaluate possible side effects. A 60 % and 20% of long-term survivals (> 6 months) were obtained in the pMBRT homogeneous and heterogeneous dose groups, respectively. No significant lesions were observed in the pMBRT groups. These results indicate that pMBRT widens the therapeutic window for gliomas and might offer a curative option. The fact that a significant tumor control is achieved with inhomogeneous dose distributions contradicts the classical paradigm of standard radiotherapy and points at the participation of distinct radiobiological mechanisms.

**References**:

^1^Prezado et al. Med. Phys. 40, 031712, 1–8 (2013).

^2^Prezado et al., Nat. Scie. Reports 7, 14403 (2017).

### O 083: Anti-Tumor and Anti-Metastasis Effects of Carbon-Ion Radiotherapy Combined with Anti-CTLA4 Antibody

#### L. Ma^1,2^, A. Takahashi^1^, T. Shimokawa^2^

##### ^1^Gunma University Heavy Ion Medical Center, Gunma University, Maebashi, Japan, ^2^National Institute of Radiological Sciences, National Institutes for Quantum and Radiological Science and Technology, Chiba, Japan

**Background**: Carbon-ion radiotherapy (CIRT) is an advanced effective radiotherapy in tumors due to its biological properties and excellent dose distribution. Although CIRT has shown good outcomes to local tumor control, distant tumor, especially metastasis control is an important matter as with any type of cancer treatment.

**Purpose**: To improve the therapeutic effectiveness of CIRT, we aimed to find an optimal combination therapy for CIRT. In this study, we evaluated anti-CTLA4 antibody immunotherapy, as a partner for CIRT.

**Materials and Methods**: Mouse carcinoma cell lines (Colon-26 and LM8) were grafted into double hind legs of syngeneic mice (BALB/c and C3H/He). Seven days later, right side tumors were irradiated with carbon-ions (290 MeV/n, 6-cm spread-out Bragg peak, 1 or 2 Gy). From 1 day after irradiation, anti-CTLA4 antibody was administrated intraperitoneally into mice every 3 days for 4 times. Tumor diameter of both legs were measured three times per week after irradiation. The number of lung metastases was evaluated within four weeks after irradiation.

**Results**: (1) Irradiated tumor: in LM8-bearing C3H/He mouse model, carbon-ion and anti-CTLA4 antibody combined therapy significantly suppressed tumor growth compared to single treatment group. (2) Distant tumor (un-irradiated tumor): in Colon-26-bearing BALB/c mouse model, combined therapy effectively suppressed tumor growth than carbon-ion group. (3) Distant metastasis: in Colon-26-bearing BALB/c mouse model, combined therapy showed no enhanced anti-metastasis effect. As contrast, in LM8-bearing C3H/He mouse model, combined therapy effectively inhibited distant lung metastases.

**Conclusions**: The combination of CIRT and anti-CTLA4 antibody immunotherapy has potential ability to enhance anti-tumor and anti-metastasis effects.

### O 084: Targeting Mitochondrial Fission for the Sensitization of Lymphoma Cells to Proton Therapy

#### M. Sertorio^1^, T. Kupneski^1^, A. Hinge^1^, L.A. Runck^1^, A. Mascia^2^, J.P. Perentesis^1^, M.D. Filippi^1^, Y. Zheng^1^, S.I. Wells^1^

##### ^1^Cincinnati Children's Hospital Medical Center, Cancer and Blood Diseases Institute, Cincinnati, USA, ^2^University of Cincinnati, Department of Radiation Oncology, Cincinnati, USA

For decades, the metabolism of tumor cells has been described as highly glycolytic. A metabolic switch from mitochondrial oxidative phosphorylation to aerobic glycolysis (Warburg effect) was initially attributed to defective mitochondria. However, not all tumors depend on aerobic glycolysis for energy metabolism, and mitochondria can be critical for cancer cells survival. Inhibition of mitochondrial biogenesis or function can decrease tumor development or growth. Ionizing radiation including proton therapy induces oxidative stress which in turn causes chromatin damage. Interestingly, mitochondria harbor circular DNA and are also sensitive to oxidative stress. Ionizing radiation can damage mitochondrial DNA directly. To clear the damage, mitochondria undergo a cycle of fission-fusion through mitochondrial biogenesis and mitophagy (autophagic clearance of defective mitochondria), for which targeting may be a radiation sensitizer. Our RNA sequencing data suggests proton therapy de-regulates mitochondrial gene expression in lymphoma cells. Photon and proton irradiation lead to differential metabolic reprogramming suggesting mitochondrial involvement. Furthermore, in mouse embryo fibroblasts (Mef) from mice that expressed a mitochondrially targeted fluorophor (Mito-Dendra), proton radiation induced a rapid increase in mitochondrial mass and fission (Fig.1). Surviving cancer cells harbored a normal mitochondrial network, suggesting selection for baseline mitochondrial activities. The efficacy of a mitochondrial fission inhibitor mdivi-1 was determined in murine J3D and human Bl41 lymphoma cells, with or without proton radiation (Fig.2). Increased apoptosis was achieved in both cell lines when compared to mDIVI-1 or proton radiation alone. Modulating mitochondrial dynamics fusion-fission can be a novel approach to enhance proton therapy efficacy in lymphoma.

### O 085: Nano-Molar Concentrations of Duocarmycin SA Significantly Enhances the Effects of Proton Irradiation on Human Glioblastoma Cells

#### M. Vazquez^1^, K. Boyle^2^

##### *^1^Loma Linda University Medical Center, Radiation Medicine, Loma Linda, USA*, *^2^Loma Linda University, School of Pharmacy, Loma Linda, USA*

The duocarmycin SA (DSA) is a cell cytotoxin that possesses ultra-potent activity against cancer cells and in experimental animals. DSA derive its remarkable activity via a sequence selective alkylation of adenine-N3 in AT-rich sites present in DNA, which initiates a cascade of cellular events leading to apoptosis. Alkylating agents such as DSA, enhance the cytotoxic effects of photon irradiation. It is unknown whether of DSA alters the efficacy of proton irradiation in human tumor cell lines. Therefore, we investigated the impact of DSA on the responses to proton irradiation (SOBP, 250 MeV) using two human glioblastoma cell lines (GBMs), U-138 and LN-16. Cell toxicity was assessed by colony formation, proliferation and apoptosis assays after combination treatment with nM concentrations of DSA and single fraction of proton irradiation (LET of 0.4 keV/um). In this study, we found that concentrations as low as 0.001 nM of DSA enhanced the sensitivity of proton irradiation on GBMs. Co-administration of DSA and proton exposures resulted in a significant decrease in cell survival, increase in apoptosis and a marked inhibition of cell growth. Mechanistically, DSA and proton exposure synergistically can increase the complexity of DNA damage; impair repair processes via alkylation process, increase of intra-cellular level of ROS and activation of apoptotic pathways, which account for the decrease in cell survival, cell apoptosis and proliferation inhibition. Together, these data implicate that ultra-low concentrations of DSA combined with proton irradiation at therapeutic doses represents an effective therapy regimen against GBMs.

### O 086: Radiosensitization Effect of Talazoparib, a PARP inhibitor, on Glioblastoma Stem Cells Exposed to X-rays and Carbon Ions

#### Y. Saintigny^1^, P. Lesueur^2^, E. El-Habr^3^, M.P. Junier^3^, H. Chneiweiss^3^, L. Castera^4^, E. Muller^4^, D. Stefan^2^, F. Chevalier^1^

##### ^1^CEA, IRCM - Institut François Jacob, Caen, France, ^2^Centre François Baclesse, Radiation Therapy unit, Caen, France, ^3^Sorbonne Universities, INSERM - Neuroscience Seine - IBPS, Paris, France, ^4^Centre François Baclesse, Plateforme de Séquençage, Caen, France

Glioblastoma, the most common and deadly glioma subtype in adults, is a radioresistant tumor. Despite continuous improvements in treatment, tumor recurrence and therapy resistance still occur in a high proportion of patients. One underlying reason for this radioresistance might be the presence of glioblastoma cancer stem cells (GSCs), which feature high DNA repair capability. PARP protein plays an important cellular role by detecting the presence of damaged DNA and then activating signaling pathways that promote appropriate cellular responses. Thus, PARP inhibitors have recently emerged as potential radiosensitizing agents.

In this study, we investigated the preclinical efficacy of talazoparib, a new PARP inhibitor, in association with low linear energy transfer (LET) and high LET irradiation in two GSC cell lines. Reduction of GSC fraction, impact on cell proliferation, and cell cycle arrest were evaluated for each condition. All combinations were compared with a reference schedule—photonic irradiation combined with temozolomide (Stupp combination). The use of PARP inhibitors combined with carbon beam irradiation drastically reduced the GSC frequency of GBM cell lines in vitro. In particular, talazoparib combined with photonic irradiation induced a marked and prolonged G2/M block, whereas talazoparib combined with 2-Gy carbon irradiation reduced the GSC fraction by up to 98%. These results show that talazoparib is a new candidate that effects radiosensitization in radioresistant GSCs, and its combination with high LET particle therapy is promising.

### O 088: Molecular Mechanism of PD-L1 Upregulation in Cancer Cells after Irradiation

#### H. Sato^1^, A. Niimi^2^, T.B.M. Permata^1^, Y. Hagiwara^1^, N. Endang^1^, Y. Yoshimoto^1^, K.D. Held^3^, T. Nakano^1^, S. Atsushi^4^

##### ^1^Gunma University Graduate School of Medicine, Department of Radiation Oncology, Maebashi, Japan, ^2^Gunma University Initiative for Advanced Research, Research Program for Heavy Ion Therapy- Division of Integrated Oncology Research, Maebashi, Japan, ^3^Massachusetts General Hospital / Harvard Medical School, Department of Radiation Oncology, Boston, USA, ^4^Gunma University Graduate School of Medicine, Education and Research Support Center, Maebashi, Japan

**Background**: Immune checkpoint inhibitors have recently demonstrated marked clinical efficacy in cancer treatment. Anti-programmed death 1 (PD-1) antibody blocks interaction between the PD-1 receptor and its ligand, programmed death ligand-1 (PD-L1). Previous reports reveal that PD-L1 expression is upregulated in cancer cells after irradiation. However, the molecular mechanism underlying PD-L1 upregulation after irradiation is not fully elucidated. In this study, we hypothesized that DNA damage signaling, particularly DNA double strand break (DSB) repair, is involved in PD-L1 upregulation following irradiation.

**Methods**: U2OS cells were irradiated to induce DSB, and PD-L1 expression was examined by immunoblotting, real-time PCR, and immunofluorescence assay. Specific inhibitors against Ataxia telangiectasia mutated (ATM), ataxia telangiectasia and Rad3 related protein (ATR), or Chk1 were examined for their involvement in PD-L1 upregulation after DSBs. To identify the genes inducing PD-L1 upregulation after X-irradiations, siRNA screen targeting DSB repair was conducted by immunoblotting.

**Results**: PD-L1 upregulation was observed after irradiation. This upregulation requires ATM/ATR/Chk1 kinase activities. siRNA screen revealed that depletion of either BRCA2 or Ku80 enhanced PD-L1 upregulation after X-irradiation. This upregulation required Chk1 kinase activity in BRCA2 depleted cells and DNA end resection followed by Chk1 activation in Ku80-depleted cells. DSBs also activate STAT1 and STAT3 signaling, and IRF1 is required for DSB-dependent PD-L1 upregulation.

**Conclusions**: Our study reveals that DNA repair pathways are involved in the upregulation of PD-L1 in response to irradiation. These data suggest that the efficacy of anti-PD-1/PD-L1 therapy is further enhanced in DSB repair defective cancer cells, particularly when radiotherapy is combined.

### O 091: Multicellular 3-Dimensional Spheroid Models to Study Tumor-Stroma-Immune Cell Response to Proton Radiation

#### M. Sertorio^1^, L.A. Runck^1^, A. Mascia^2^, J.P. Perentesis^1^, Y. Zheng^1^, S.I. Wells^1^

##### ^1^Cincinnati Children's Hospital Medical Center, Cancer and Blood Diseases Institute, Cincinnati, USA, ^2^University of Cincinnati, Department of Radiation Oncology, Cincinnati, USA

Head and neck cancer (HNC) is a leading cancer type with dismal outcomes and poor quality of life survivors. Conventional culture models have led to new insights into molecular features of HNC, but have limitations for the study of complex tumor responses to chemo-radiation. Most in vitrostudies are carried out with a homogeneous cell population in the exponential growth phase where every cell has unlimited access to nutrients/oxygen. Tumor growth in vivo represents a more complex system, with an extracellular gradient of nutrients/oxygen towards the tumor core. Importantly, such tumors consist of cells at different stages of growth, differentiation and stemness, and are often infiltrated by stromal and immune cells which modulate key tumor phenotypes. Since conventional models have a high rate of failure for outcome predictions in clinical trials, we generated 3D spheres from the SCC1 head and neck cancer cells, recreating physiologically relevant tumor characteristics. To model stroma, we embedded fibroblasts into tumor spheres which increased sphere size compared to controls. To model immune contributions and microenvironment, THP-1 monocytes were allowed to invade the sphere. Flow cytometric (Fig 1A) and confocal (Fig 1B) detection of THP-1 cells reproducibly quantifies immune cell invasion, and reveals a requirement for fibroblasts for monocyte infiltration and also shows diminished infiltration of monocytes following radiation. This model is currently being used to analyze the differential cellular and molecular responses to proton vs. X-ray irradiation and to explore immune cells activity/differentiation upon proton irradiation in the context of a physiologically relevant tumor microenvironment.

### O 092: Innovative Device for Online In-Vivo Dose Monitoring Based on Charge Unbalance in Patients Undergoing Radiotherapy

#### P. Cirrone^1^, G. Petringa^1^, A. Amato^1^, G. Cuttone^1^, A. Mazzaglia^1^, G. Privitera^2^, L. Raffaele^2^, V. Salamone^2^, C. Spatola^2^

##### ^1^INFN, Laboratori Nazionali del Sud, Catania, Italy, ^2^Ospedale Universitario 'Policlinico-Vittorio Emanuele', Radiology, Catania, Italy

A completely new on-line, non-invasive, bias-free detector for relative and absolute in-vivo dose monitoring, has been realized to be employed for patients undergoing charged particles radiotherapy. The basic idea is to use the patient as a Faraday cup, collecting the current injected from the beam directly from a section of its skin, far from the beam entering point and using a conductive electrode. Such new dosimeter has been tested in-vitro, in an electrically-isolated phantom irradiated with 62 MeV clinical proton beams at CATANA facility of INFN-LNS (Catania, Italy) [^1^]. The proton beam current has been collected from an electrode immersed in a water phantom and positioned outside the irradiation field. The charge measurements resulted in accordance with the theoretical prevision. The detector response has been studied as a function of the dose released in water, dose rate and irradiation field. In all cases, the experimental data have been compared with the theoretical results.

The acquired data demonstrate the usefulness of the proposed approach as in-vivo beam monitoring during a charged particles irradiation. Preliminary tests have been also carried out by using electron and carbon beams. In-vivo tests, carried out on protontherapy patients demonstrated the clinical applicability of the developed as on-line dose monitoring. The system has been protected by a National Italian patent, N 102017000087851 while submission for international patent is ongoing.

**References**:

^1^G.A.P. Cirrone et al. A 62-MeV proton beam for the treatment of ocular melanoma at Laboratori Nazionali del Sud- INFN. IEEE Trans. Nucl. Sci. 2004; 51: 860-864.

### O 093: Monte Carlo Calculated Correction Factors for a Proton Calorimeter in Clinical Proton Beams

#### F. Romano^1^, D. Shipley^1^, H. Palmans^1,2^

##### ^1^National Physical Laboratory, Chemical- Medical & Environmental Science Department, Teddington, United Kingdom, ^2^EBG MedAustron GmbH, Medical Physics Group, Wiener Neustadt, Austria

Calorimetry is the only fundamental method for measuring the absorbed dose according to its definition. A calorimeter measures the temperature rise resulting from the irradiation of an absorber (an insulated core in the case of a graphite calorimeter). The National Physical Laboratory (NPL) is currently commissioning a portable graphite calorimeter as a primary standard of absorbed dose to water for clinical proton beams with the aim of achieving an uncertainty on reference dosimetry for protons of around 2% (at 95% confidence level) based on an IPEM Code of Practice under development.

The aim of this work is to determine two key corrections required to obtain absorbed dose to graphite from a calorimeter measurement in a range of mono-energetic and clinical proton beams.

A Monte Carlo application was developed with TOPAS (v3.1) based on Geant4 to determine the gap (kgap) and volume averaging (kvol) correction factors for the calorimeter. kgap and kvol were determined (1) for a range of mono-energetic proton energies between 60 MeV and 230 MeV, (2) for clinical SOBPs. The simulation results indicate that kgap is within 0.1% from unity for mono-energetic proton beams (and different amounts of buildup) when the beam diameter is large enough so that lateral charged particle equilibrium is established. kvol was found to be dependent on beam energy (changing by 0.6%) but not significantly on the amount of buildup in front of the calorimeter. For the reference clinical SOBP, both correction factors are within 0.1% from unity which is ideal for reference dosimetry.

### O 094: Towards Prompt Gamma Spectroscopy for C-12 Range Control: Development of a Synchrotron-Dedicated System

#### P. Magalhaes Martins^1,2,3^, R. Dal Bello^1,2,4,5^, G. Hermann^6^, T. Kihm^6^, M. Laorden^7^, M. Seimetz^7^, J. Seco^1,4^

##### ^1^German Cancer Research Center - DKFZ, Biomedical Physics in Radiation Oncology, Heidelberg, Germany, ^2^Authors contributed equally, Germany, ^3^Instituto de Biofísica e Engenharia Biomédica - IBEB, Faculdade de Ciências da Universidade de Lisboa, Lisbon, Portugal, ^4^University of Heidelberg, Department of Physics and Astronomy, Heidelberg, Germany, ^5^International Max Planck Research School - IMPRS, Quantum Dynamics in Physics - Chemistry and Biology, Heidelberg, Germany, ^6^Max Planck Institute for Nuclear Physics, Heidelberg, Germany, ^7^CSIC/UPV, Instituto de Instrumentación para Imagen Molecular - I3M, Valencia, Spain

Particle therapy with protons and ions of Helium, Carbon and Oxygen features the highest clinical potential in terms of efficacy and effectiveness. Among those ion species, C-12 beams present enhanced features such as reduced lateral spread and increased biological effect. The Bragg peak guarantees that healthy organs distal to this peak receive no radiation, reducing significantly side effects. This however requires a superior range control with reduced range margins. Conversely to other range verification techniques (e.g., post-treatment PET), the prompt gamma radiation allows for a real-time range monitoring due to its nearly-instantaneous emission. Prompt gamma spectroscopy has been demonstrated for proton range verification in a cyclotron-based facility. We aim at optimizing this technique for C-12 beams accelerated by synchrotron-based facilities. The different acceleration process raises challenges due to the irregular microscopic time-structure of the delivered beam. In this work, we present a dedicated system for such synchrotron-based facilities. The prototype is composed by scintillating fibers that provide the system trigger, a CeBr3 detector (Ø 1.5′' × 3′') coupled to a Hamamatsu R13089 photomulti¬plier tube and an FADC module for high-rate data acquisition. The results obtained with C-12 beams at clinical intensities show that such prototype resolves the arrival time of single particles in a bunch with many particles (Picture 1). We were able to differentiate the prompt gamma component from the one induced by secondary particles (Picture 2). The excellent time resolution of the CeBr3 detectors leads to a robust noise reduction in the energy spectra.

### O 095: Detection of Ionacoustic Signal from Low Intensity Proton and Carbon Ion Beams

#### W. Wang^1^, K. Shahnazi^1^, Z. Chen^1^, Y. Sheng^1^

##### ^1^Shanghai Proton and Heavy Ion Center, Department of Medical Physic, Shanghai, China

**Purpose**: Our preliminary acoustic detection system was designed to detect the ionacoustic waves created by low intensity proton and carbon ion Bragg-peaks (BPs).

**Method**: An acoustic detector (frequency span: 20 to 400 Hz) was attached to water phantoms downstream of the beam. The isocenter of the beam was aligned to the center of detector front surface. Single spot beams with water equivalent range of 80 mm and 135 mm were used with maximum intensity 2.6x109/ s for proton and maximum intensity 6.5x107/s for carbon ion. The created pulses lasted from 50 ms to 1000 ms. By using different water phantoms, all the BPs were 3 mm in front of the detector. To investigate the BP to detector distance (BTD) where this detector could obtain stronger signal, the beam ranges of both proton and carbon ion were decreased by 2 mm placing the BPs 5 mm in front of the detector.

**Results**: The detector position at 3 mm in front of proton BP gained the acoustic signal while another detector position did not. The signals of positions at 3 mm and 5 mm in front of carbon ion BP were comparable. At the same position irradiated by the same ion type, the amplitude of signal was related to the intensity of the beam.

**Conclusion**: This acoustic detection system can detect the ionacoustic waves from low intensity proton and carbon ion BP. Due to special dosimetric characteristic of carbon ion, Its BTD is longer than proton BTD. However, further investigation still ongoing.

### O 096: Evaluation of the Particle Transport Accuracy in the FLUKA Monte Carlo Code for Proton Therapy Dosimetry Applications

#### A. Lourenco^1,2^, D. Shipley^2^, H. Bouchard^3^, G. Royle^4^, H. Palmans^2,5^

##### ^1^University College London, Medical Physics and Biomedical Engineering, London, United Kingdom, ^2^National Physical Laboratory, Chemical- Medical & Environmental Science Division, London, United Kingdom, ^3^Université de Montréal, Département de Physique, Montréal, Canada, ^4^University College London, Department of Medical Physics and Biomedical Engineering, London, United Kingdom, 5EBG MedAustron GmbH, Medical Physics Group, Wiener Neustadt, Austria

Ionization chamber perturbation factors are assumed to be unity in current dosimetry protocols for proton dosimetry. Monte Carlo modelling of these detectors allow the accurate determination of such factors. The aim of this work was to assess the accuracy of the particle transport in the FLUKA Monte Carlo code by performing a Fano cavity test. The latter is an important step to find optimal parameters that allow computing the ionization chamber response accurately. The Fano test was performed for proton energies ranging from 60 MeV to 250 MeV. A source routine was written to randomly generate protons uniformly by mass. The full geometry of the chamber was simulated and chamber materials were overridden to water-property materials but their original densities were kept. The accuracy of the code was tested by comparing the dose scored in the different mass density regions where the dose is expected to be uniform because of charged particle equilibrium conditions predicted by Fano's theorem. FLUKA was found to pass the Fano test within 0.15% when all particles (including electrons) are transported across the therapeutic energy range from 60 MeV to 250 MeV which is in line with the accuracy of other Monte Carlo codes. Future work will aim to calculate perturbation factors with the optimal parameters established by this work in a composite field defined to cover what is termed a Standard Test Volume (STV) for the new IPEM Code of Practice for proton beam dosimetry.

### O 097: A Comprehensive Evaluation of Air Gaps in Pencil Beam Scanning Proton Therapy: Dosimetric Impact and Clinical Tolerance

#### Z. Xiao^1^, Y. Zhang^1^, T. Phan^2^, E. Lee^1^, D. Ionascu^2^, M. Lamba^2^, A. Mascia^1^

##### ^1^Proton Therapy Center, Cincinnati Children's Hospital/University of Cincinnati Medical Center, Cincinnati, USA, ^2^Department of Radiation Oncology, University of Cincinnati Medical Center, Cincinnati, USA

**Purpose**: The air gap between range shifter and patient surface can greatly impact the in-air spot size. The purpose of this work is to validate the dosimetric impact of air-gap deviations, and thus establish clinical guideline for a more flexible PBS plans delivery.

**Methods**: Using treatment planning, single spot profiles were first calculated with range shifter at different air gaps and energies to model the impact on in-air spot sizes. Treatment plans were optimized in phantom at varying energies and snout positions. Then, each plan was subsequently delivered at different air gaps by varying snout positions relative to the planned snout positions. In addition, real patient plans were also evaluated to validate the tolerances of air gap variations.

**Results**: The air gap has greater impact on the spot size at low energy than higher energy with 5cm range shifter. When snout retracts away from patient, the penumbra increases to worst-case scenario of 1.66 cm with 37cm airgap difference for 90MeV.The flatness of isocenter planar dose could varyfrom 0.86% to 19.5% depending on the air gap deviation from nominal. Such trend depends on both energy and spot spacing. Based on this study, a 5cm air gap clinical tolerance was established. This tolerance results in an absolute dose change below 1.0% and a penumbra change of less than 0.3cm, despite in-air spot size difference of as high as 20%.

**Conclusion**: Through studying the impact of air gap variation both in phantom and in patient, we established and validated a clinical air gap tolerance for PBS treatment delivery.

### O 098: Intensity-Modulated Proton Therapy with Small Spots and Volumetric-Modulated Arc Therapy in the Treatment of Stage III Non-Small Cell Lung Cancer

#### *C. Liu^1^*, S.E. Schild^1^, J. Shan^2^, T.B. Daniels^1^, W.G. Rule^1^, X. Ding^1^, M. Bues^1^, T.T. Sio^1^, N.Y. Yu^1^, W. Liu^1^

##### ^1^Mayo Clinic, Radiation oncology, Phoenix, USA, ^2^Arizona State University, Biomedical Informatics, Tempe, USA

**Purpose**: To compare the dosimetric performance of volumetric-modulated arc therapy (VMAT) and small spot intensity-modulated proton therapy (ssIMPT) for stage III non-small cell lung cancer (NSCLC).

**Methods and Materials**: We selected 24 NSCLC patients. Among them 12 patients were treated by ssIMPT and the remaining 12 were treated by VMAT at our institution. Both ssIMPT and VMAT plans were generated by delivering prescription doses to internal target volumes (ITV) on averaged 4D-CTs. The dose-volume-histograms (DVH) band method was used to quantify plan robustness. In-house software was developed to evaluate interplay effects with randomized starting phases of each field per fraction. DVH indices were compared using Wilcoxon rank sum test.

**Results**: Compared with VMAT, ssIMPT delivered significantly lower cord Dmax, heart Dmean, and lung V5Gy[RBE] with comparable ITV dose coverage, homogeneity, hot spots, and protection of other OARs. In terms of plan robustness, the ssIMPT plans were statistically better than VMAT plans in heart Dmean, but were statistically worse in target dose coverage, cord Dmax, lung Dmean, V5Gy[RBE], and V20Gy[RBE]. For other DVH indices, they were comparable. The ssIMPT plans still met the standard clinical treatment requirement with interplay effects considered.

**Conclusions**: ssIMPT improves cord, heart, and lung sparing compared to VMAT and achieves clinically acceptable plan robustness and interplay effects. Our study supports the usage of ssIMPT to treat lung cancer patients, which may result in less toxicity and better quality of life for patients. These can be determined with direct comparisons of clinical outcomes in the future clinical trials.

### O 099: Preliminary Observation of Carbon Ion Radiotherapy for Tracheal Adenoid Cystic Carcinoma

#### J. Chen^1^, J. Mao^2^, N. Ma^1^, J. Lu^1^, G. Jiang^2^

##### ^1^Shanghai Proton and Heavy Ion Center, Radiation Oncology, Shanghai, China, ^2^Shanghai Proton and Heavy Ion Center- Fudan University Cancer Hospital, Radiation Oncology, Shanghai, China

**Objective**: Complete surgical resection for primary tracheal adenoid cystic carcinoma (TACC) is difficult, and photon irradiation isn't sensitive enough. Here we report the short-term effect and adverse effects after carbon ion radiotherapy (CIRT) for TACC.

**Methods**: From March 2016 to October 2017, a total of 10 patients with TACC were treated using CIRT. Among them, three patients had recurrent disease (two after surgery, and one after brachytherapy), one received a complete resection for stage I disease, and the other 6 had locally advanced disease (3/6 received endoscopic treatment before CIRT). All patients received CIRT using pencil-beam scanning technique. Except that the patient with recurrent disease after brachytherapy received 60GyE/20Fx, the postoperative prophylactic patient and one recurrent patient after surgery received 66GyE/22Fx, all other patients received 69GyE/23Fx.

**Results**: At the last follow-up in December 2017 with a median follow-up time of 5.5 (1.5∼16.4) months, among the 9 patients with gross tumors, 3 patients achieved near complete remission, 2 achieved partial response, and 4 remained stable disease per RECIST 1.1 criteria. The patients received prophylactic CIRT had no evidence of disease. Except 1 patient experienced grade 4 tracheal stenosis who relieved after stent implantation, no other grade ≥3 adverse effects were observed. Grade 2 acute toxicities included 1 hoarseness and 1 neutropenia, both relieved after CIRT. Hypothyroidism in one patient was the only observed grade 2 late toxicity.

**Conclusion**: CIRT is safe and effective in the management of TACC during a short-time observation. Promising outcomes are to be expected after longer follow up.

### O 100: Definitive Proton Based Radiotherapy for Unresected Chordomas: Local Results, Tolerability, and Patterns of Progression

#### Y. Chen^1^, R. Miao^2^, G. Maquilan^1^, F. Hornicek^3^, T. DeLaney^1^

##### ^1^Massachusetts General Hospital, Radiation Oncology, Boston, USA, ^2^Massachusett General Hospital, Radiation Oncology, Boston, USA, ^3^UCLA- formerly MGH, Orthopedic Oncology, Boston, USA

**Purpose**: The best reported results for chordoma involve surgical resection with neoadjuvant or adjuvant particle based therapy. We report the outcomes of a large cohort of primary spine chordoma patients treated with definitive proton based RT after only an initial biopsy without any further surgical intervention.

**Methods**: IRB approved analysis was performed on 52 patients with unresected chordoma treated with primary photon/proton radiotherapy. Kaplan-Meier survival analysis was used to estimate local control (LC) and overall survival (OS) rates.

**Results**: The patients had median f/u 2 years, median age 67 years (5–89), 52% females, 48% male, 10% C spine, 2% T spine, 4% L spine, 67% sacrum, and 17% overlapping, median GTV dose 77.4 GyE, and median CTV dose encompassing microscopic extension into extraosseous soft tissue or spinal canal. The three and five-year LC, DMFS, and OS were 86% and 75%, 85% and 73%, 86% and 81%, respectively. Complications included: 13 sacral insufficiency fractures, 2 fibrosis, 2 foot drop, 1 erectile dysfunction, 1 urinary retention, and 3 rectal bleeding. Mapping MRI of LF onto RT plan showed variable patterns of progression: some primarily within the CTV whereas others entirely within the original GTV.

**Conclusion**: While high dose definitive proton based therapy is an option for unresected spine chordomas, longer follow up is warranted for durability. Overall toxicity is acceptable and nerve preservation rate is high. Both expansion of the high dose region as well as escalation of dose may be necessary to improve local control with definitive high dose proton based therapy.

### O 101: Proton-Based High-Dose Pre-Operative Radiation Is Associated with Higher Local Control than Low-Dose Pre-Operative Radiation with Equivalent Rate of Wound Complication

#### G. Maquilan^1^, A. Bhatt^2^, R. Miao^1^, F. Hornicek^3^, T. DeLaney^1^, Y.L. Chen^1^

##### ^1^Massachusetts General Hospital, Radiation Oncology, Boston, USA, ^2^The Ohio State University, Radiation Oncology, Columbus, USA, ^3^Ronald Reagan UCLA Medical Center, Orthopaedic Surgery, Santa Monica, USA

**Purpose**: Proton-based pre-operative radiation (pre-op RT) to 19.8–50.4 GyRBE, complete resection, and then post-operative radiation (post-op RT) to ≥70 GyRBE total results in high local control rates for spine chordomas. However, the ideal pre-op RT dose is not known. We investigated the impact of high- vs. low-dose proton-based pre-op RT on local control and wound complication rates in patients with sacrococcygeal chordoma.

**Methods**: We retrospectively reviewed 103 patients; 39 patients received ≤30 GyRBE (“low dose,” median: 19.8 GyRBE) and 64 patients received >30 GyRBE (“high dose,” median: 50.4 GyRBE). Major wound complication was defined as wound dehiscence and/or infection within a 3-month post-operative period.

**Results**: Mean age at diagnosis was 55 years, mean tumor size was 8.1 cm, and 35.9% of patients were female. At median follow up of 76.2 and 47.6 months, respectively, 5-year local control rates for the low- and high-dose groups were 69% and 92% (p=0.03). Factors related to better local control: high pre-op RT dose, addition of post-op RT. Factors associated with worse local control: dedifferentiated histology, lymph node positive disease, R2 resection. Higher pre-op RT doses were associated with higher rates of R0 resection (81.3% vs. 56.4%, p=0.03). There was no difference in wound complication rates (low vs. high: 43.7% vs. 42.2%, p=0.84).

**Conclusions**: Proton-based high-dose pre-op RT is associated with higher rates of R0 resection and local control and equivalent wound complication rates when compared to low-dose pre-op RT. Based on these results, we recommend use of higher dose pre-op RT for sacrococcygeal chordomas, followed by resection and post-op RT.

### O 102: Integration of Electrocardiogram-gated CT with Coronary Angiography (E-CTA) and Pencil Beam Scanning Proton Therapy for the Treatment of Mediastinal Lymphomas

#### S. Lester^1^, K. Taparra^2^, A. Hunzeker^1^, A. Tasson^1^, J. Martenson^1^, R. Funk^1^, M. Blanchard^1^, T. Whitaker1, N. Laack^1^

##### ^1^Mayo Clinic, Department of Radiation Oncology, Rochester, USA, ^2^Mayo Clinic, Mayo Medical School, Rochester, USA

**Purpose**: E-CTA enables accurate visualization of cardiac substructures in time and space. We report our experience with integrating E-CTA into our proton therapy clinic.

**Methods**: A prospective observational study utilizing E-CTA was initiated in 2014, and in June of 2015 our pencil beam scanning proton therapy center opened and we began enrolling patients undergoing IMPT for comparison. Simulation was performed using a DIBH technique, followed by E-CTA in the treatment position. The E-CTAs were co-registered and 8 cardiac substructures were delineated in systole, diastole, then combined into single PRVs. Involved site principles were used for target volume delineation. Radiation plans were generated with/without use of the cardiac substructures during optimization. Selection of the plan for treatment was at the discretion of the treating physician.

**Results**: After the proton center opened, 15 patients were enrolled and 14 treated with PBT using E-CTA. One patient was treated with IMRT because of technical difficulties minimizing uncertainty at the chestwall-lung interface. Mediastinal adenopathy > 9 cm: 9 patients. Median prescription: 30 Gy in 15 fractions. Median follow: 1.9 years. To date no patient has experienced a relapse. Median dosimetric parameters to cardiac substructures are listed in Table 1. 12 of 14 PBT patients were treated with plans optimized with substructures. Reasons for not selecting the substructure plan: the inferior extent of the CTV was superior to the substructures (n=1) and extenuating logistical circumstances (n=1).

**Conclusions**: Integration of pencil beam scanning proton therapy with E-CTA was technically feasible and facilitates quantification of IMPT dose to cardiac substructures.

### O 103: Clinical Results of Phase I Trial of Preoperative IMPT with Simultaneously Integrated Boost (SIB) to the High-Risk Margin for Retroperitoneal Sarcomas (RPS)

#### T.F. DeLaney^1^, Y.L. Chen^1^, E.H. Baldini^2^, D. Wang^3^, B.Y. Yeap^4^, S.M. Hahn^5^, G.P. Nielsen^6^, E. Choy^7^, J.T. Mullen^8^, S.S. Yoon^9^

##### ^1^F.H. Burr Proton Center- Massachusetts General Hospital- Harvard Medical School, Radiation Oncology, Boston, USA, ^2^Dana-Farber Cancer Institute/Brigham and Women's Hospital/Harvard Medical School, Radiation Oncology, Boston, USA, ^3^Rush University Medical Center, Radiation Oncology, Chicago, USA, ^4^Massachusetts General Hospital- Harvard Medical School-, Biostatistics/Department of Medicine-, Boston, USA, ^5^M.D. Anderson Cancer Center, Radiation Oncology, Houston, USA, ^6^Massachusetts General Hospital- Harvard Medical School, Pathology, Boston, USA, ^7^Massachusetts General Hospital- Harvard Medical School, Medical Oncology Section/Department of Medicine, Boston, USA, ^8^Massachusetts General Hospital- Harvard Medical School, Surgical Oncology Section/Department of Surgery, Boston, USA, ^9^Memorial Sloan Kettering Cancer Center, Surgery, New York City, USA

**Purpose**: To review clinical outcome in phase I IMPT arm of non-randomized, phase I/II trial of IMRT or IMPT to escalate preoperative radiation dose to RPS tumor CTV2 at high risk for positive margins to decrease local recurrence (LR).

**Methods and Materials**: Patients aged >18 years with primary/locally recurrent RPS were treated with preoperative IMPT, 50.4 GyRBE/28 fractions, to CTV1 (GTV + subclinical disease) with SIB to CTV2 to doses of 60.2, 61.6, and 63.0 GyRBE/28 fractions of 2.15, 2.20, and 2.25 GyRBE, respectively. The primary objective of the phase 1 study was to achieve the maximum planned target dose of 63 GyRBE to CTV2, to then be tested in phase 2 study.

**Results**: Eleven patients were treated without acute dose limiting toxicity. Acute toxicity was generally mild with no radiation interruptions. Nine patients went to surgery, while 2 patients did not because of interval development of metastases. No unexpected perioperative morbidity was noted. One patient whose ureter was dissected off tumor and received 57.5 GyRBE developed late hydronephrosis that was treated by stent. Retained ureters were subsequently constrained to 50.4 GyRBE without further problem. With 29-61 months (median 44 months) follow-up, there are no LR and 7/9 patients remain NED.

**Conclusions**: IMPT dose escalation to CTV2 to 63 GyRBE was achieved without acute dose limiting toxicities. Local control is encouraging. Phase 2 study of IMPT is currently accruing at that dose. Parallel IMRT phase 1 arm is currently accruing. Retained ureters are now constrained to 50.4 GyRBE to avoid ureteral stricture.

### O 104: Calculating Dose Weighted Mean Lineal Energy for Input into the Microdosimetric Kinetic Model for Predicting Proton RBE

#### M. Newpower^1^, O. Vassiliev^1^, D. Patel^1^, L. Bronk^2^, F. Guan^1^, D. Grosshans^2^, R. Mohan^1^

##### ^1^UT MD Anderson Cancer Center, Radiation Physics, Houston, USA, ^2^UT MD Anderson Cancer Center, Radiation Oncology, Houston, USA

The microdosimetric kinetic model (MKM) has been used for decades to model relative biological effectiveness (RBE) of protons and heavy ions. The microdosimetric quantity dose-weighted mean lineal energy (yD) must be measured or calculated for input into the MKM to predict RBE. In this study we calculate the lineal energy distribution function, f(y) at 131 different proton energies using Geant4 DNA version 10.2.0. yD is then calculated using a fluence-weighted averaging technique from the proton energy spectra obtained using Geant4 at the water equivalent depths corresponding to the experimental investigations. Preliminary RBE values from MKM are compared to the data from two recent proton RBE experiments in Figs. 1 and 2. The fitting parameter rd, (the radius of the domain), used in the MKM, was optimized to yield a best fit when compared to the experimental RBE values. Overall, this study accurately predicted RBE values, typically within 15% across dose averaged linear energy transfer (LETD) values ranging between 1 to 19.5 keV/μm, except for three data points in the Guan and Bronk et al data. The Monte Carlo model was optimized by Patel and Bronk and shows even better agreement with MKM predictions. In the Bragg peak and region beyond, this study improves proton RBE predictions when compared to existing phenomenological models. Figure 1: RBE predictions (circles) compared to RBE data measured by Guan and Bronk et al (2015). Figure 2: MKM RBE predictions in black, compared to measurements done by Patel and Bronk et al (2017), in blue.

### O 105: Monte Carlo Simulations of Direct DNA Damage on Gold Nanoparticle Enhanced Proton Therapy

#### M. Sotiropoulos^1^, N.T. Henthorn^1^, J.W. Warmenhoven^1^, R.I. Mackay^2^, K.J. Kirkby^1,3^, M.J. Merchant^1,3^

##### ^1^University of Manchester, Division of Cancer Sciences- Faculty of Biology- Medicine and Health, Manchester, United Kingdom, ^2^The Christie NHS Foundation Trust, Christie Medical Physics and Engineering, Manchester, United Kingdom, ^3^The Christie NHS Foundation Trust, The Christie, Manchester, United Kingdom

Gold nanoparticles (GNPs) have demonstrated a radiosensitization potential under proton irradiation. Initially the radiosensitization effect was attributed to physical interactions of radiation with the gold and the production of secondary electrons that induce DNA damage. However, experiments have revealed that biological and chemical mechanisms may contribute to the radiosensitization effect.

To understand the underlying physical mechanisms of radiosensitization and DNA damage induction at the cellular level, a computational cell model with detailed nuclear DNA structure was implemented in the Geant4 Monte Carlo simulation toolkit. A realistic GNP distribution was incorporated, allowing for the formation of clusters of vesicles filled with GNP. Clinically relevant gold concentrations for the GNP size of 6, 15, and 30 nm were considered. Protons with linear energy transfer values found in a spread out Bragg peak (1.3-26.9 keV/μm) were simulated. The locality of the effect was also addressed. To quantify the physical contribution to the DNA damage the formation of single (SSB) and double strand breaks (DSB), and break complexity were scored. For a dose fraction of 2 Gy, each scenario was repeated 1000 times to get an average number of SSB and DSB numbers.

Our model was able to reproduce SSB and DSB yields similar to the literature. However, for the combinations of GNP size and proton energies studied in the present work, no significant increase in the SSB and DSB formation was observed. As GNP enhanced proton therapy have been proven experimentally, our results suggest a stronger contribution from alternative mechanisms.

### O 106: In-silico Modelling of Non-Homologous End Joining and Homologous Recombination Can Demonstrate Mechanistic Interactions between Repair Pathways

#### S. Ingram^1,2^, J. Warmenhoven^1^, N. Kirkby^1,3^, R. Mackay^2^, K. Kirkby^1,3^, M. Merchant^1,3^

##### ^1^University of Manchester, Division of Molecular and Clinical Cancer Sciences- Faculty of Biology- Medicine and Health, Manchester, United Kingdom, ^2^The Christie NHS Foundation Trust, Christie Medical Physics and Engineering, Manchester, United Kingdom, ^3^The Christie NHS Foundation Trust, Manchester, United Kingdom

Particle therapy is known to cause characteristic locoregional damage patterns on the nanoscale level of DNA. The complexity of the damage produced is believed to impact directly the ensuing DNA repair pathways. Resultant variations between the chosen fast Non-Homologous End Joining (NHEJ) and slow Homologous Recombination (HR) repair pathways impact the cellular-scale fidelity of repair. The repair of radiation damage is a key concept in current clinical practice; fractionation regimes and fractionation gaps are both influenced by repair, and largely dictate treatment durations.

Mathematical models are a proven investigatory tool in radiotherapy, allowing for the development of multi-scale models that strive to relate physical dose to biological effect. To date, a missing link in the chain of multi-scale models for radiation response has been a mechanistic Monte Carlo model of repair pathway choice. By building mechanistic repair pathway models onto established DNA damage models, it is possible to examine theoretical concepts which are difficult to explore in traditional experiments.

A computational framework for modelling both NHEJ and HR repair pathways following Monte Carlo radiation track simulation of DNA damage has been developed within Geant4-DNA. The sensitivity to three scenarios for repair pathway selection (Figure 1) has been assessed using literature data for repair kinetics, over a range of radiation qualities. The long-term aim is to couple this framework to higher scale models of radiation response to examine the effects on fractionation in particle therapy and assess the theoretical assumption of sub-lethal repair.

### O 107: Infidelity in Proton Therapy: Getting Double Strand Breaks Back Together

#### N. Henthorn^1^, J. Warmenhoven^1^, M. Sotiropoulos^1^, R. Mackay^2^, N. Kirkby^1,3^, K. Kirkby^1,3^, M. Merchant^1,3^

##### ^1^University of Manchester, Division of Cancer Sciences, Manchester, United Kingdom, ^2^The Christie NHS Foundation Trust, Medical Physics and Engineering, Manchester, United Kingdom, ^3^The Christie NHS Foundation Trust, Manchester, United Kingdom

The biological effectiveness of protons can be described by the cellular response to the induced DNA damage. Failure to repair damage can result in residual or misrepaired double strand breaks (DSBs), both of which can be toxic to the cell. In this work, we present the results of track structure simulations, with realistic DNA geometries, coupled to a diffusion-controlled model of the Non-Homologous End Joining (NHEJ) repair pathway. With these simulations, we are able to predict DSB complexity, spatial distribution, and how they affect repair efficacy. We show how these factors depend on the physical parameters of the beam, dose and linear energy transfer (LET). The DSB complexity and proximity both depend on LET, while the initial DSB yield depends on both LET and dose. We show that, due to the tethered diffusion of broken DNA ends, proximity determines DSB misrepair, depending linearly on the number of neighbouring DSBs and predict that the probability of residual DSBs is constant after 24 hours of repair, but due to the dependency of DSB yield on dose and LET the absolute yield of residuals increases linearly with these factors. Correlations are developed from these detailed simulations, allowing for prediction of biological outcomes from dose and LET alone. This is applied to the clinically relevant case of a Spread-out Bragg peak. Here we show increasing biological effect with proton depth. The results of this work can easily be incorporated into treatment planning systems, leading to biologically optimised treatments.

### O 108: Is There a Clinical Indication of the Need of Variable Proton RBE in Patients with Hypothyroidism?

#### P. Yepes^1^, L. Chen^2^, S. Frank^3^, M. Jomaa^3^, D. Mirkovic^4^, A. Mohamed^5^, R. Mohan^6^

##### ^1^Rice University, Physics and Astronomy, Houston, USA, ^2^University of Texas MD Andeson Cancer Center, Radiation Oncology Department, Houston, USA, ^3^University of Texas MD Anderson Cancer Center, Radiation Oncology Department, Houston, USA, ^4^University of Texas MD Anderson Cancer Center, Radiation Physics, Houston, USA, ^5^University of Texas MD Anderson Cancer Center, Radiation Oncology, Houston, USA, ^6^University of Texas MD Anderson Cancer Center, Radiation Physics, Houston- TX, USA

**Purpose**: Our knowledge of the Relative Biological Effectiveness for protons comes from in-vitro experiments. In addition to the clinically utilized simplest model of assuming constant RBE=1.1, a variety of proton RBE models exist based on such experiments. We attempt to discriminate among the various models analyzing proton and photon patients with hypothyroidism.

**Methods**: A cohort of over 500 head-and-neck photon and over 100 proton patients with radiation dose in the thyroid was analyzed. Patients were evaluated for elevated thyroid-stimulating hormone TSH. An NTCP model was developed for photons, which has the thyroid volume and mean dose as parameters. For proton patients the mean dose in the thyroid was estimated with five models: fixed RBE, Wilkins-Oelfke, McNamara, Wedenburg, and RMF. All the models used the dose-averaged LET and dose as calculated with the Fast Dose Calculator. The NTCP models fit with the photon patients was applied to the proton patients with dose values calculated with the various variable RBE models. If proton RBE was perfect, the photon NTCP model should also explain the proton results.

**Results**: The photon NTCP explained the proton hypothyroidism results with variable RBE better than for fixed RBE.

**Conclusions**: An indication of the need of variable RBE was found in the analysis of hypothyroidism patients.

### O 109: SDD: A Standardised Data Format To Record DNA Damage And Facilitate Collaboration

#### J.W. Warmenhoven^1^, N. Henthorn^1^, M. Merchant^1,2^, K. Kirkby^1,2^, A. McNamara^3^, J. Schuemann^3^, H. Paganetti^3^, K. Prise^4^, S. McMahon^4^

##### ^1^University of Manchester, Division of Cancer Sciences, Manchester, United Kingdom, ^2^The Christie NHS Foundation Trust, n/a, Manchester, United Kingdom, ^3^Massachusetts General Hospital & Harvard Medical School, Department of Radiation Oncology, Boston, USA, ^4^Queen's University Belfast, Centre for Cancer Research and Cell Biology, Belfast, United Kingdom

Computational modelling is a powerful tool for investigating complex multi-scale biological systems and enables investigations in ways not possible by experiment. One such system that is of critical importance in radiation oncology is the interplay between DNA repair pathways and the damage caused by radiation. Many groups have developed models of DNA damage and repair, but they are often self-contained or only connected to other models with ad-hoc interfaces. The lack of a common standard to connect models prevents robust inter-model comparison and evaluation of the dependencies, uncertainties, and assumptions made in the modelling chain.

We propose a new Standard to record DNA Damage (SDD) that facilitates interfacing between models. A header (Table 1) describes the damage model, simulation geometry, and irradiation conditions. Row-by-row descriptions of individual breaks (Table 2) can be populated with levels of detail at the user's discretion. At a minimum the spatial location and types of breaks must be provided, with possible detail up to the level of DNA sequences and break structure in more detailed models.

An international collaboration between Queen's University Belfast, University of Manchester, and the Massachusetts General Hospital has implemented this format, and demonstrated its effectiveness in cross-comparison of damage and repair models. We report how these results reveal the emergent mechanisms responsible for the convergent behaviour of these structurally different model sets. We share this format with the aspiration of gaining consensus for a standard interface which we believe will aid collaboration and comparison between investigators of biological response to radiation.

### O 110: Nano-vesicles Are a Potential Tool to Monitor Therapeutic Efficacy of Carbon Ion Radiotherapy in Prostate Cancer

#### Q. Yu^1,2^, S. Wu^1,2^, Y. Zhang^1,2^, X. Chen^1, 2^, Q. Zhang^1^, S. Fu^1,2,3^

##### ^1^Department of Radiation Oncology- Shanghai Proton and Heavy Ion Center, Fudan University Cancer Hospital, Shanghai, China, ^2^Department of Oncology- Shanghai Medical College, Fudan University, Shanghai, China, ^3^Key Laboratory of Nuclear Physics and Ion-beam Application MOE, Fudan University, Shanghai, China

Exosomes are nano-vesicles that contribute to the effectiveness of many treatments. The aim of this study was to identify profiles of microRNA (miRNA) contained in serum exosomes that are differentially regulated in patients with prostate cancer undergoing carbon ion radiotherapy (CIRT). RNA was extracted from serum exosomes of eight patients with localized prostate cancer before and after CIRT, and miRNA was analyzed by the next generation sequencing. Kyoto Encyclopedia of Genes and Genomes (KEGG) pathway analysis showed that the major signaling pathways associated with the proliferation of prostate cancer cells, such as MAPK, PI3K-AKT, mTOR, and AMPK may be implicated in the mechanism of CIRT action. Notably, 57 miRNAs present in serum exosomes were significantly altered after application of CIRT. A high pre-CIRT expression level of specific miRNAs (miR-493-5p, miR-323a-3p, miR-411-5p, miR-494-3p, miR-379-5p, miR-654-3p, miR-409-3p, miR-543, and miR-200c-3p) predicted therapeutic benefit of CIRT (p < 0.05). Post-CIRT expression of miR-654-3p and miR-379-5p was also associated with CIRT efficacy (p < 0.05). These results suggest that the anti-prostate cancer mechanisms elicited by CIRT at the molecular level may involve exosomal miRNAs. Furthermore, specific miRNAs in serum exosomes, particularly miR-654-3p and miR-379-5p, may serve as promising non-invasive biomarkers predicting efficacy of CIRT for prostate cancer.

### O 111: MRI Radiomic Features to Predict Treatment Response in Carbon Ion Treated Prostate Cancer Patients

#### S. Wu^1,2^, Y. Jiao^3^, X. Ren^3^, Q. Yu^1,2^, Q. Zhang^1^, Y. Zhang^1,2^, X. Chen^1,2^, Q. Wang^3^, S. Fu^1,2,4^

##### ^1^Department of Radiation Oncology-Shanghai Proton and Heavy Ion Center, Fudan University Cancer Hospital, Shanghai, China, ^2^Department of Oncology- Shanghai Medical College, Fudan University, Shanghai, China, ^3^School of Biomedical Engineering, Shanghai Jiao Tong University, Shanghai, China, ^4^Key Laboratory of Nuclear Physics and Ion-beam Application MOE, Fudan University, Shanghai, China

**Purpose**: Radiomics could noninvasively aid therapeutic response assessment and prognosis prediction in multiple cancers. This study aims to investigate the predictive role of radiomics in treatment response for prostate cancer patients receiving carbon ion radiotherapy (CIRT). As prostate cancer is multifocal, radiomic features of tumor and whole prostate were both analyzed.

**Materials and Methods**: Forty localized prostate cancer patients treated with CIRT from Jun. 2014 to Sep. 2017 were enrolled. Patients received 63-66 GyE over 23-24 fractions or 59.2 GyE over 16 fractions. Patients were divided into poor and good response group based on plasma levels of prostate-specific antigen (PSA) after CIRT. PSA less than 0.5ng/ml was defined as good response. MRI images performed before and after CIRT were collected and analyzed.

1. For 17 patients, whole prostate was contoured as region of interest (ROI) on T2-weighted and apparent diffusion coefficient (ADC) images. And radiomic features were extracted. Changes of radiomic feature values were compared between two groups.
2. For 23 patients, tumor was contoured as ROI. After radiomic features extraction, support vector machines (SVM) with nested leave-one-out cross-validation (LOOCV) were applied to predict treatment response.

**Results**: The changes of 2836 radiomic feature values from prostate before and after CIRT showed significantly difference (P < 0.05) between poor and good response group. The tumor features based model achieved accuracy of 0.70 for predicting treatment response.

**Conclusion**: The preliminary results indicate radiomics could be potentially used to estimate treatment response in prostate cancer patients receiving CIRT.

### O 113: Online Plan Adaptation of Head and Neck IMPT Treatments Based on Cone Beam CT Imaging and GPU Monte Carlo Simulations

#### P. Botas^1,2^, J. Kim^1,3^, B. Winey^1,4,^ H. Paganetti^1,4^

##### ^1^Massachusetts General Hospital, Radiation Oncology, Boston, USA, ^2^Heidelberg University, Faculty of Physics, Heidelberg, Germany, ^3^Yonsei Cancer center, Radiation Oncology, Seoul, Korea Republic of, ^4^Harvard Medical School, Radiation Oncology, Boston, USA

Our purpose is to demonstrate an online IMPT plan adaptation algorithm based on GPU Monte Carlo and cone beam CT imaging to improve the daily dose distribution aiming to recover the original plan quality.

Replicating an adaptive workflow, IMPT plans of 4 patients were each evaluated with GPU MC on 6 weekly scatter-corrected CBCTs. The evaluation in the weekly geometry was done on contours warped with a vector field (VF) calculated between the CT and the weekly scan using deformable image registration. The prescribed beamlets were raytraced on the planning CT to obtain distal reference positions (endpoints). Beamlets were shifted following the VF at the endpoints and raytraced in the weekly CBCT to adjust their energies. Four modes were considered for positions and energy corrections: shifts were left unconstrained, constrained to an isocenter shift, constrained to a range shifter and constrained to isocenter shift and range shifter. The adapted plans were again evaluated on the CBCTs.

The plan was improved at every fraction for patients 2 to 4 with isocenter shift: from average V98 across fractions of 86.0, 85.7 and 87.9% to 93.7, 96.6 and 96.4% per patient. Averages of D2-D98 improved from 16.3, 16.2 and 10.2 Gy(RBE) to 9.9, 9.0 and 7.5 Gy(RBE). OARs were also better spared. The unconstrained mode performed similar to the isocenter shift. The other modes performed worse. The algorithm was unable to recover good plan quality for patient 1. An online adaptation algorithm was developed that significantly improved the plan quality in 18 out of 24 fractions.

### O 114: Functional MRI for Early Changes Detection and Adaptive Planning in Proton-Therapy: Preliminary Investigations

#### G. Buizza^1^, C. Paganelli^1^, G. Fontana^2^, S. Molinelli^3^, L. Anemoni^4^, A. Iannalfi^5^, L. Preda^6^, G. Baroni^1,2^

##### ^1^Politecnico di Milano, Department of Electronics Information and Bioengineering DEIB, Milan, Italy, ^2^National Center of Oncological Hadrontherapy CNAO, Bio-Engineering Unit, Pavia, Italy, ^3^National Center of Oncological Hadrontherapy CNAO, Medical Physics Unit, Pavia, Italy, ^4^National Center of Oncological Hadrontherapy CNAO, Radiology Technicians Unit, Pavia, Italy, ^5^National Center of Oncological Hadrontherapy CNAO, Radiotherapy Unit, Pavia, Italy, ^6^National Center of Oncological Hadrontherapy CNAO, Diagnostic Imaging Unit, Pavia, Italy

**Purpose**: To deepen the understanding of functional MRI parameters as guidance for detecting early changes in proton-therapy and for an envisioned functional adaptive planning.

**Methods**: Ten patients affected by meningioma were enrolled at CNAO (Pavia) for a dedicated MRI protocol in proton-therapy, based on various imaging sessions (Figure 1). RBE-weighted dose maps [Gy(RBE)] were computed from the baseline CT using the clinical treatment planning system. Voxels in each gross tumor volume (GTV) were grouped into three regions (DoseDOWN, DoseMID and DoseUP) depending on whether the dose was below 95%, between 95% and 105% or above 105% of the prescribed dose. Friedman test (α=0.05) was used to compare mean MRI values along time.

**Results**: Apparent diffusion coefficient (ADC) showed significant changes only at follow-up, whereas true diffusion (D) and perfusion fraction (f) between t0 and baseline or intra-treatment exams. By analysing D at different dose groups, significance was found comparing t0-baseline and t0-t20 in any group; additionally, for D(DoseUP) significance was found comparing t0-t10. For f, significant changes were only found in DoseMID comparing t0-baseline and t0-t20.

**Conclusions**: From preliminary results, ADC could be used for detecting changes after the end of the treatment whereas D and f for detecting early changes, thus supporting MRI-based functional adaptive planning. The analysis of D and f parameters with respect to dose suggests that D is more sensitive to the dose delivered. However, this could be affected by the different number of samples across the dose groups and related to planning constraints in the GTV.

### O 115: A Method to Evaluate the Clinical Utility of Proton Radiography for Geometric Patient Alignment

#### M. Pankuch^1^, E. Dejongh^2^, F. DeJongh^2^, V. Rykalin^2^, N. Karonis^3,4,5^, C. Ordonez^5^, J. Winans^5^, C. Sarosiek^6^, G. Coutrakon^6^, J. Welsh^7^

##### ^1^Northwestern Medicine Chicago Proton Center, Medical Physics, Warrenville, USA, ^2^ProtonVDA Inc, Physics, Batavia, USA, ^3^Northern Illinois University, Computer Science, DeKalb, USA, ^4^Argonne National Laboratory, Mathematics and Computer Science Division, Argonne, USA, ^5^Northern Illinois University, Center for Research Computing and Data, DeKalb, USA, ^6^Northern Illinois University, Physics, DeKalb, USA, ^7^Stritch School of Medicine- Loyola University - Chicago, Radiation Oncology, Maywood, USA

Proton Radiography is a process in which protons that have traversed an object are back-projected to reconstruct an image of the object. The reconstructed image will not only possess information on the geometric alignment of the object (which can be used for patient set-up), but will also convey information on water equivalent path-length of the protons (which can be used for range verification). This investigation defines a process for evaluating the accuracy of proton radiographs for patient alignment and compares these results to the current clinical standard of X-ray radiography alignment.

Reconstructed proton radiographs were created using Monte Carlo simulation in TOPAS. The objects are a group of 20 CT image sets that are offset from a nominal position with known translations and rotations. The Monte Carlo simulation modeled the proton radiography system that is currently in development by ProtonVDA. This system uses an incident position detector, an exit position detector, and a calorimeter. Incident and exit proton positions and the residual energy of individual protons are scored at the resolution of the detectors. These data are then processed through an iterative, most likely path back-projection algorithm.

The simulated proton radiographs are used to obtain correction vectors for patient misalignment. A similar process is done in parallel with TPS generated digitally reconstructed X-ray radiographs on the offset CT images sets. Direct comparisons in accuracy to X-ray alignments can be made. Preliminary results demonstrate that proton radiography may offer acceptable contrast and resolution for patient alignment for many commonly treated sites.

### O 116: Online IMPT Adaptation Using Fast, Automatic and Robust Dose Restoration

#### K. Bernatowicz^1^, X. Geets^2^, E. Sterpin^3^, J. Guillaume^4^

##### ^1^Université catholique de Louvain, Molecular Imaging- Radiotherapy and Oncology, Brussels, Belgium, ^2^Cliniques universitaires Saint-Luc UCL, Radiotherapy and Oncology, Brussels, Belgium, ^3^KU Leuven, Department of Oncology, Leuven, Belgium, ^4^Ion Beam Applications, Louvain-la-Neuve, Belgium

IMPT offers excellent dose conformity and healthy tissue sparing, but it can be substantially compromised in the presence of anatomical changes. A major dosimetric effect is caused by density changes, which alter the planned proton range in the patient. Three different methods, which automatically restore an IMPT dose on a daily CT image were implemented and compared: (1) simple dose restoration (DR) using optimization objectives of the initial plan, (2) voxel-wise dose restoration (vDR), and (3) iso-dose volume dose restoration (iDR). Dose restorations were calculated on different clinical cases, selected to test large range adaptation, complex dose distributions and robust re-optimization. All dose restorations were obtained in <5 min, without manual adjustments of the optimization settings. The evaluation of initial plans on repeated CTs showed large dose distortions, which were substantially reduced after restoration (fig.1). All dose restoration methods improved DVH-based scores in propagated target volumes and OARs. Analysis of local dose differences showed that, although all dose restorations performed similarly in high dose regions, iDR restored the initial dose with higher precision and accuracy in the whole patient anatomy (fig.2). High quality dose restoration is essential to minimize or eventually by-pass the physician approval of the restored plan, as long as dose stability can be assumed. Motion (as well as setup and range uncertainties) can be taken into account by including robust optimization in the dose restoration. Restoring clinically-approved dose distribution on repeated CTs does not require new ROI segmentation and is compatible with an online adaptive workflow.

### O 117: Short-Lived Positron Emitters for In-Beam Positron Emission Tomography (PET) Verification of Helium Therapy

#### I. Ozoemelam^1^, E.R. van der Graaf^1^, S. Brandenburg^1^, P. Dendooven^1^

##### ^1^University of Groningen, KVI-Centre for Advanced Radiation Technology, Groningen, Netherlands

**Objective**: Previous studies (^1,2^) have shown the feasibility of in-beam PET monitoring of proton therapy using very short-lived positron emitters such as 12N (T1/2 = 11 ms). The extension of this approach to helium ions demands information on the production yield of these positron emitters. We present the first results on the production of these very short-lived positron emitters for monitoring of helium therapy.

**Methods**: The cyclotron at KVI-CART was used to bombard water, graphite and calcium phosphate targets with 3He and 4He beams (Rangewater = 22 mm). The production of positron-emitting nuclides was assessed via the detection of the 511 keV annihilation gammas using a sodium iodide detector (NaI). The beam was delivered in pulses to identify the PET nuclides via half-life analysis.

**Results**: The production of short-lived PET nuclides was observed in all irradiated targets. The irradiation of water and graphite targets shows a decay with a half-life of about 10 ms, indicating the production of 12N and/or 13O (fig. 1). The production of this short-lived component is 8.3×10-4 per 4He and 1.2×10-3 per 3He. On the water target also 18Ne (T1/2 = 1.67 s) and 19Ne (T1/2 = 17.2 s) were observed with 4He beam (fig. 2), whereas only 18Ne is seen with 3He.

**Conclusions**: The large production of a 10 ms component, about 50% higher than for protons1, is promising for real-time verification of helium therapy.

**References:**

^1^P. Dendooven et al., Phys. Med. Biol.,60 (23), pp.8923-8947

^2^H.J.T. Buitenhuis et al., Phys. Med. Biol.,62 (12), pp.4654-4672

### O 118: Transducer Specifications for Thermoacoustic Range Verification of Pencil Beams Delivered by a Synchrocyclotron

#### S. Patch^1^, R. Lindert^1^, T. Zhao^2^, D. Hoff^3^, T. Webb^4^, L. Sobotka^5^

##### ^1^UW-Milwaukee, Physics, Milwaukee, USA, ^2^Washington University St. Louis, Radiation Oncology, St Louis, USA, ^3^Washington University St. Louis, Chemistry, St Louis, USA, ^4^Washington University St. Louis, Physics, St Louis, USA, ^5^Washington University St. Louis, Chemistry and Physics, St Louis, USA

Thermoacoustic range verification offers the potential of online range verification and correlation with patient anatomy, if ultrasound imaging arrays can be customized with transducer elements sensitive to thermoacoustic emissions.

Amplitudes of thermoacoustic emissions depend upon proton beam intensity and pulse duration. Instantaneous deposition of 1 cGy in water induces O(1 Pa) pressure increase, which decays as it propagates.

We developed transducer specifications for pencil beam proton therapy accounting for proton pulse duration. Thermoacoustic emissions were simulated using TRIM Monte Carlo and k-Wave acoustic software. 90K protons with Gaussian lateral profile upon entry into a water target and 230 MeV energy (Fig 1). 3D k-Wave propagated emissions due to a Gaussian pulse with 6 us FWHM, as delivered by a Mevion S250 at low beam current. Fig. 1: Simulations assume a water target with transducer 5 cm distal (yellow square).

For a transducer located 5 cm distal to the treatment spot, thermoacoustic emissions were bandlimited below 150 kHz and had peak amplitude of 43 mPa/cGy assuming instantaneous proton delivery (Fig. 2). 6 us delivery time reduced bandwidth to 100 kHz and amplitude to 28 mPa/cGy. Intense proton beams are expected to increase pulse duration to 10 us, reducing bandwidth to 85 kHz and thermoacoustic efficiency to 19 mPa/cGy. Fig. 2: Thermoacoustic pulses (a) and spectra (b) assuming Gaussian pulse durations with FWHM=0, 5, 10 microseconds.

To detect weak thermoacoustic emissions, custom ultrasound imaging arrays with additional receive elements, such as PVDF transducers or optical microphones, will be required.

### O 120: Virtual 4DCT for Image Guided Proton Therapy of Moving Tumors

#### G. Janssens^1^, W.S. Ingram^2^, S. Huang^2^, M. Kang^3^, K. Souris^4^, K. Teo^2^, L. Lin^2^

##### ^1^Ion Beam Applications SA, Research, Louvain-la-Neuve, Belgium, ^2^UPENN, radiation oncology, Philadelphia, USA, ^3^Georgetown University, Radiation Oncology, Washington DC, USA, ^4^Université catholique de Louvain, MIRO- IREC institute, Louvain-la-Neuve, Belgium

Daily cone-beam CT (CBCT) imaging can be used to assess the impact of anatomical changes during treatment. This can be done using a virtual CT combining the intensities from the planning CT and the anatomy from the CBCT. In the case of liver and lung tumor patients treated with pencil beam scanning proton therapy (PBSPT), motion and interplay can be included in the computation of the delivered dose. This requires the creation of a virtual 4DCT.

Ten (5 lung and 5 liver) patients were studied with one planning 4D-CT (p4D-CT) and 2-3 CBCT. CBCT projections were sorted into 8 breathing phases using the Amsterdam Shroud method. Two different 4D-CBCT reconstruction algorithms from RTK were then compared. The MA-ROOSTER method uses a priori velocity fields extracted from the 4D-CT to improve the 4D-CBCT reconstruction, while the Conjugate Gradient iterative reconstruction only uses the sorted projections. The velocity fields (Vel4D-CT and Vel4D-CBCT) that characterize the motion between each phase were generated with deformable image registration (DIR) in OpenReggui. Mid-Position 3D images (MP-CT and MP-CBCT) were created from p4D-CT and 4D-CBCTs, respectively. The virtual MP-CT (vMP-CT) was then generated by deforming MP-CT on MP-CBCT with DIR. This vMP-CT can eventually be deformed to each phase using Vel4D-CBCT in order to generate a virtual 4D-CT (v4D-CT).

Results show that both reconstruction methods led to similar superior-inferior motion estimation. However, due to the motion artifacts, the Conjugate Gradient method led to higher variability in motion estimation in left-right and anterior-posterior directions, resulting in distorted v4D-CT.

### O 121: Investigation of Helium as a Range Probe in Carbon Ion Therapy

#### C. Graeff^1^, N. Saito^2^, C. Schuy^1^, U. Weber^1^, L. Volz^3^, P. Piersimoni^3^, J. Seco^3^, M. Krämer^1^

##### ^1^GSI, Biophysics, Darmstadt, Germany, ^2^DKFZ, Medical Physics, Heidelberg, Germany, ^3^DKFZ, BioMedical Physics, Heidelberg, Germany

Range uncertainty is a major uncertainty in particle therapy, especially in extracranial irradiations. As helium has the same magnetic rigidity but more than twice the range at the same velocity as Carbon, it is possible to mix beams, using Carbon for therapy while simultaneously detecting Helium exiting the patient to assess beam range.

A single field irradiation was simulated on a lung cancer patient using the GSI in-house TPS TRiP98, using a field consisting of 90% carbon and 10% helium. Target dose was optimized to 2 Gy(RBE). Figure 1 shows the dose distribution of both fields in the patient, with an artificial water block added behind the patient to illustrate the detection. Figure 2 shows a dose profile along the beam axis with dose displayed in a log scale. The 10% helium deposit less than 0.5% of the target dose while passing through the patient, notably also less than the Carbon fragment tail within the patient. The maximum energy in this patient 255 MeV/u, corresponding to ranges of 12.8 and 39.2 cm H2O for helium and carbon, respectively.

In order to detect Helium ions beyond the patients, all secondary nuclear fragments must be separated from primary Helium ion. MC simulations and experiments will be used to study the best combination of simultaneous Carbon-therapy and Helium-imaging. Fast detection systems could be used to check ranges online. In moving tumors, range-based motion detection or gating would be possible, especially in the lung with large density differences of tumor and lung.

### O 122: Phase I/II Randomized Study of Proton Beam Irradiation With Anti-Vascular Endothelial Growth Factor for Exudative Age-Related Macular Degeneration

#### L. Mukkamala^1^, K. Mishra^2^, I. Daftari^3^, A. Moshiri^4^, S.S. Park^4^

##### ^1^Vitreoretinal service- Department of Ophthalmology & Vision Science, University of California Davis Eye Center, Sacramento, CA, USA, ^2^University of California San Francisco, Radiation Oncology, San Francisco, USA, ^3^Department of Radiation Oncology, University of California San Francisco, San Francisco, CA, USA, ^4^Vitreoretinal service- Department of Ophthalmology & Vision Science, University of California Davis Eye Center, Sacramento, CA, USA

**Purpose**: To report outcomes of a phase I/II prospective, randomized, sham-controlled, double-blinded, single center study of low dose proton beam radiation treatment (PBT) and intravitreal anti-vascular endothelial growth factor (anti-VEGF) therapy for exudative age-related macular degeneration (eAMD).

**Methods**: Participant eyes with newly diagnosed eAMD with vision between 20/40 and 20/400 were included. Exclusion criteria included diabetes or other ocular comorbidities affecting vision. Patients were randomized equally into 3 groups: (a) sham radiation, (b) 16GyE total, or (c) 24GyE total PBT (PBT given over 2 fractions). All patients underwent 3 monthly intravitreal anti-VEGF treatments (ranibizumab or bevacizumab), and additional anti-VEGF injections as needed based on ophthalmologic follow-up examinations.

**Results**: A total of 34 subjects (34 eyes) were enrolled of which 22 completed monthly follow-up for 12 months and 16 subjects completed 24 months follow-up [roughly equally distributed among groups: (a) 6; (b) 4; (c) 6]. Mean VA was similar among groups at baseline and follow up. Eyes that received 24GyE required significantly fewer injections at 1 and 2 years compared to sham (p=0.006, p=0.017, respectively). Suspected mild radiation retinopathy was seen in 3 cases [group (b) 1 and (c) 2], and none were visually significant. No eye had severe vision loss.

**Conclusion**: PBT significantly reduced the need for anti-VEGF injections in eyes with newly diagnosed eAMD during a 2 year follow-up. Longer follow-up and a larger multicenter study would be needed to further investigate the safety and efficacy of combination PBT and anti-VEGF therapy for eAMD.

### O 123: Treatment of Conjunctival Carcinomas

#### J. Thariat^1^, J. Caujolle^2^, C. Maschi^2^, S. Baillif^2^, J. Salleron^3^, S. Lassalle^4^, A. Gerard^5^, J. Herault^5^, A. Santoni^2^

##### ^1^Centre Lacassagne, Radiation Oncology, Nice, France, ^2^Chu, Ophthalmology, Nice, France, ^3^Cav, Biostatistics, Nancy, France, ^4^Chu, Pathology, Nice, France, ^5^Cal, Radiation Physics, Nice, France

**Introduction**: Conjunctival melanomas are treated with surgery, mitomycin and proton therapy. We evaluated prognostic factors for ocular surface squamous neoplasia (OSSN) including in invasive squamous cell carcinomas (SCC) and micro-invasive in situ carcinomas (micCIS) in comparison with in situ carcinomas (CIS).

**Methods**: Treatments, complications and recurrences of OSSN patients treated in Nice from 2002 to 2017 were collected. Recurrence was defined as any new clinical lesion.

**Results**: in 109 patients (110 eyes), OSSN were most common in males, median age 67 years. Symptoms were present in 29% of cases. Lesions were small (64%), located on interpalpebral bulbar conjunctiva with limbal involvement (72%), with corneal invasion (51%). There were 51%, 36% and 13% micCIS+CIS, SCC and dysplasia, respectively. Lateral surgical margins were positive in 34%. Proton therapy was performed in SCC but not in micCIS+CIS. Mitomycin was delivered in micCIS+CIS but in 17% of SCC. With mean follow-up of 36.4 months, recurrence occurred in 21.5% of patients within 12 months, including 20% of CIS, 25% of micCIS and 13% of SCC. Prognostic factors for recurrence were symptoms (p=0.040), large tumors (p=0.009) and positive margins (p=0.031). Among micCIS+CIS, none had proton therapy and relapse rates were similar between micCIS and CIS (HR 1.4, p=0.56). Regional and systemic metastases were rare and specific deaths represented 1.8%.

**Conclusion**: Aggressive multimodal local treatment of SCC including proton therapy yielded best outcomes despite more aggressive biologic behavior. Despite no indication that micCIS and CIS should be treated differently, this might be worth larger multicentric series.

### O 124: Eye Tracking System for Patient Positioning in Ocular Proton Therapy

#### R. Via^1^, F. Hennings^2^, G. Fattori^2^, M. Peroni^2^, A. Pica^2^, A. Lomax^2^, D.C. Weber^2^, G. Baroni^1^, J. Hrbacek^2^

##### ^1^Politecnico di Milano, Department of Electronics- Information and Bioengineering, Milano, Italy, ^2^Paul Scherrer Institut, Center for Proton Therapy, Villigen, Switzerland

**Purpose**: An eye tracking system (ETS) for non-invasive eye localization has been developed for ocular treatments at PSI. Eye position and orientation is estimated by 3D referencing of pupil and cornea curvature centres (P-point and C-point) through stereoscopic optical imaging and recognition of corneal reflections produced by multiple-source infrared illumination. Here, the performance of this ETS for tumour-beam alignment has been assessed.

**Methods**: ETS data from 111 fractions delivered to 31 uveal melanoma patients has been analysed, with ETS performance being simulated by comparing measured P- and C-points to the X-ray determined treatment geometry. Patient-specific adaption of the ETS measurement to the ocular anatomy was additionally developed and evaluated. Accuracy was assessed by comparing the eye position attained from ETS-based or clip-based guidance to the planned treatment geometry. Additionally, treatment plans were evaluated assuming an ETS navigated eye position, using the percentage of the tumour volume covered by 95% of the prescribed dose (V95) as the metric.

**Results**: Table 1 and 2 summarise the results. Mean 3D positioning accuracies of 1.46mm (1.29mm interquartile range) could be achieved with the ETS, compared to 0.46(0.39)mm for clip-based/X-ray positioning. This would translate into 83.8% of evaluated patients having a target coverage of V95 > 95%, compared to 100% for the clip based approach.

**Conclusions**: The accuracy of an ETS system for a clip-less approach to ocular proton therapy has been assessed. Despite very promising results, the ETS approach still needs to be optimized to achieve the accuracy of clip based treatments.

### O 127: Proton Therapy for Iris Melanoma: A Series from Institut Curie Proton Therapy Center

#### R. Dendale^1^, F. Meniai-Merzouki^1^, F. Goudjil^1^, L. Lumbroso-LeRouic^2^, I. Pasquie^1^, C. Nauraye^1^, C. Levy^2^, A. Mazal^1^, L. Desjardins^2^, N. Cassoux^2^

##### ^1^CPO - Institut Curie, Radiation oncology, Orsay, France, ^2^Institut Curie, Ophthalmology, Paris, France

**Background**: Melanoma of the iris is a rare tumour with an estimated frequency of between 2 and 5% of all uveal tumours. Radiotherapy is a conservative treatment which can preserve a functional eye. This study reports the results of an Iris melanoma series treated by proton beam irradiation in Institut Curie Proton therapy Center.

**Results**: From 05/1999 to 05/2017, 98 patients received 60GyEBR in 4 fractions for iris melanoma. Median follow up was 6.4 yrs. 4 pts (4%) had a local recurence at 2 months, 1, 2 and 5 years post treatment. 94pts (96%) had a tumor local control at last follow up. 3 pts developped metastases at 2, 3 and 4 years post irradiation. 62% of the patients had a stable ou increasing visual acuity at last of follow up. 26 patients developed a cataract, but only 11 pts from them underwent a lens surgery.

**Conclusion**: Proton therapy is an efficient, conservative treatment for iris melanoma.

### O 128: Increase of Pseudoprogression and Treatment Related Effects in Low-Grade Glioma Patients Treated with Proton Radiation and Temozolomide

#### M. Dworkin^1^, W. Mehan^2^, A. Niemierko^3^, S. Kamran^4^, J. Dietrich^5^, M. Martinez-Lage^6^, K. Oh^3^, T. Batchelor^5^, J. Loeffler^3^, H. Shih^3^

##### ^1^Duke University, School of Medicine, Durham, USA, ^2^Massachusetts General Hospital, Radiology, Boston, USA, ^3^Massachusetts General Hospital, Radiation Oncology, Boston, USA, ^4^Massachusetts General Hospital, Harvard Radiation Oncology Program, Boston, USA, ^5^Massachusetts General Hospital, Stephen E. and Catherine Pappas Center for Neuro-Oncology, Boston, USA, ^6^Massachusetts General Hospital, Pathology, Boston, USA

Chemoradiation with temozolomide (TMZ) can be associated with pseudoprogression (PsP) in glioblastoma. The occurrence of early or delayed treatment effects and pseudoprogression is less well understood in low-grade gliomas (LGG). We hypothesized that adding TMZ to radiation might increase the incidence of pseudoprogression. Chart review and volumetric MRI-analysis was performed on 109 chemotherapy-naïve patients with grade I-II or IDH1-mutant grade III glioma treated with proton radiotherapy (RT) between 2005-2015. Progression was defined by new chemotherapy-initiation or histology. Post treatment related effects (PTRE) were defined as increase in T2/FLAIR or new/increase in abnormal enhancement without evidence of tumor progression or growth 6-12 months later. PsP was defined if a lesion meeting criteria for PTRE was suspicious for progression or volumetrically increased at least 40% from baseline. Late-PsP was defined as PTRE occurring more than 12 months after RT. Pearson's chi-squared test was used for statistical analysis. Median RT-dose was 54 Gy (RBE) (range: 45-60). PsP was not more common with concurrent-TMZ versus adjuvant-TMZ (PsP: 18/27, 10/12; p=0.29; enhancing-PsP: 14/27, 7/12; p=0.710). PsP was significantly more common with TMZ+RT versus RT alone (PsP: 28/39, 27/70; p<0.001; enhancing-PsP: 21/39, 24/70; p=0.047). PsP was significantly more common in presence of p53-mutation (PsP: 31/50, 9/24; p=0.048; enhancing-PsP: 24/50, 7/24; p=0.124), but unrelated to IDH1-mutant status (PsP: 37/63, 8/21; p=0.101; enhancing-PsP: 27/63, 9/21; p=1.0) and MGMT promoter methylation (PsP: 8/12, 8/17; p=0.296; enhancing-PsP: 6/12, 8/17; p=0.876). Treatment with TMZ and p53-mutation status were associated with increased PsP in LGGs treated with proton radiotherapy.

### O 129: Patterns of Care in Proton Beam Radiotherapy for Clival Chordoma

#### L. Pater^1,^ M. Stock^2^, A. Mascia^1^, E. Hug^2^

##### ^1^University of Cincinnati, Radiation Oncology, Cincinnati, USA, ^2^MedAustron Ion Therapy Center, Radiation Oncology, Wiener Neustadt, Austria

**Purpose**: Clival chordomas are uncommon tumors presenting a particular challenge to manage. Surgery is frequently limited in degree of resection. Recurrence rates following resection remain high. Radiotherapy is commonly recommended following complete resection and standard of care following incomplete resection. Proton beam therapy is the standard of care for patients requiring radiotherapy due to improved dose profile over photon therapy. We determined to establish patterns of care in the treatment of clival chordoma with proton beam therapy.

**Patients and Methods**: Members of the PTCOG CNS/Skull Base Subcommittee created a survey on the patterns of care in the treatment of clival chordomas. This was distributed to PTCOG Executive Committee Members. Potential participants were contacted by email with link to a 38 question web-based survey distributed on December 27th, 2017 with data collected on February 5th, 2018.

**Results**: Fully completed surveys were submitted from 15 institutions representing the United States, Europe and Asia. There is minimal homogeneity in dosing regimens with range from 70-77.4GyRBE. Majority of centers prescribe 95% prescription dose coverage to 100% of the CTV. Most centers define the brainstem, cervical cord and optic apparatus as structures mandating compromised tumor coverage if constraints cannot be met. Optic chiasm max point dose tolerated ranged from 51-67GyRBE. Brainstem D2 tolerated ranged from 54-63GyRBE. Significant variability exists in treatment approach and plan optimization.

**Conclusions**: There is considerable variability in the pattern of care for the treatment of clival chordoma amongst PTCOG member institutions. These data suggest the need for a standardized approach to the treatment of clival chordoma.

### O 130: Proton Therapy for Low Grade Gliomas: A Prospective Swedish Multicentre Study

#### P. Witt Nystrom^1,2^, A. Flejmer^3^, P. Bergström^4^, T. Herlestam^5^, F. Jakobsson^6^, K. Werlenius^7^

##### ^1^The Skandion Clinic, Oncology, Uppsala, Sweden, ^2^Danish Centre for Particle Therapy, Oncology, Aarhus, Denmark, ^3^Linköping University Hospital, Oncology, Linköping, Sweden, ^4^Umeå University Hospital, Oncology, Umeå, Sweden, ^5^Karolinska University Hospital, Oncology, Stockholm, Sweden, ^6^Örebro University Hospital, Oncology, Örebro, Sweden, ^7^Sahlgrenska University Hospital, Oncology, Gothenburg, Sweden

In Sweden, approximately 300 patients are diagnosed with low grade glioma (LGG, WHO grade II) yearly. LGG patients with good prognostic markers and hence an expected long-term survival are considered potential candidates for proton therapy (PT) as an alternative to photon radiotherapy to reduce long-term toxicity. The Skandion Clinic (SC) is a national public proton clinic based on distributed competence, i.e. the patients are prepared for PT at one of the seven university hospitals in Sweden and thereafter referred to the SC for PT, and then re-referred to their home hospital for follow-up. The LGG patients were identified and allocated for RT at regional neuro-oncological multi-disciplinary tumour board. Comparative photon plans were made additionally. The patients were treated with pencil beam scanning PT, with Eclipse (Varian) as treatment planning system. The patients were assessed prospectively for toxicity, and first MRI were performed 3 months after PT. Since clinical start of the SC in August 2015, 110 adult patients with a LGG grade II have been treated at the clinic. All have been treated to a total dose of 50.4-54.0 Gy(RBE). Dominating acute side effects have been alopecia, erythema and fatigue grade I-II. A comparison between different potential treatment plans showed a large significant reduction of radiation dose to several organs at risk, including brain (>30%). Proton therapy of LGG shows a well-tolerated treatment. Noticeable is several cases of asymptomatic pseudo-progression on MRI 3 months post treatment.

### O 131: Clinical Application of Dual-Energy Computed Tomography Improves Stopping-Power Prediction

#### P. Wohlfahrt^1,2^, C. Möhler^3,4^, S. Greilich^3,4^, C. Richter^1,2,5,6^

##### ^1^OncoRay – National Center for Radiation Research in Oncology, Faculty of Medicine and University Hospital Carl Gustav Carus- Technische Universität Dresden- Helmholtz-Zentrum Dresden - Rossendorf, Dresden, Germany, ^2^Helmholtz-Zentrum Dresden - Rossendorf, Institute of Radiooncology - OncoRay, Dresden, Germany, ^3^German Cancer Research Center DKFZ, Department of Medical Physics in Radiation Oncology, Heidelberg, Germany, ^4^National Center for Radiation Research in Oncology NCRO, Heidelberg Institute for Radiation Oncology HIRO, Heidelberg, Germany, ^5^Department of Radiotherapy and Radiation Oncology, Faculty of Medicine and University Hospital Carl Gustav Carus- Technische Universität Dresden, Dresden, Germany, ^6^German Cancer Consortium DKTK, partner site Dresden, Dresden, Germany

**Purpose/Objective**: Assessment of accuracy and robustness of treatment planning on dual-energy CT (DECT) in a multi-step validation and clinical implementation scheme (Fig.1,2).

**Material/Methods**: To ensure reliable translation of DECT into routine clinical application, scan settings and stopping-power prediction methods were optimized and validated using 13 different animal tissues and an anthropomorphic ground-truth phantom. The clinical relevance of DECT-based stopping-power prediction was evaluated on dual-spiral DECT scans of 102 brain-, 25 prostate- and 3 lung-tumor patients treated with protons. DECT-derived voxelwise correlations of CT number and stopping-power ratio (SPR) were used for adapting the clinically applied CT-number-to-SPR conversion (HLUT) and quantifying intra- and inter-patient variability.

**Results**: The accuracy of DECT-based stopping-power prediction was within 0.3% stopping-power and 1mm range uncertainty of validation measurements. Clinically relevant mean range shifts (±1SD) of 1.2(±0.7)% for brain-, 1.7(±0.5)% for prostate- and 2.2(±1.2)% for lung-tumor patients were obtained between dose calculations using HLUT or DECT-derived SPR. These deviations were significantly reduced (p<<0.001, two-sample t-test) below 0.3% by HLUT refinement based on patient-specific DECT information. Still, the remaining large intra-patient soft tissue diversity of approx. 6% (95% CI) and age-dependent inter-patient bone variability of 5% cannot be considered by any HLUT-based range prediction.

**Conclusion**: Additional tissue information provided by DECT allows for accurate stopping-power prediction and incorporation of patients' tissue diversity in treatment planning. These advantages can contribute to reduce CT-related range uncertainty and have been gradually translated in our clinical routine: (1) DECT-derived pseudo-monoenergetic CT dataset with generic HLUT, (2) DECT-based HLUT adaptation and soon (3) patient-specific DECT-basted stopping-power prediction.

### O 132: Using Realistic and Animal Tissue Phantoms to Validate the Raystation Monte Carlo Dose Calculation Algorithm

#### N. Schreuder^1^, L. Rigsby^1^, M. Blakey^1^, J. Renegar^1^, M. Artz^1^, S. Hedrick^1^, B. Robison^1^, S. Petro^1^, A. Meek^1^, B. Wilkinson^1^

##### ^1^Provision Cares Proton Therapy Center - Knoxville, Medical Physics, Knoxville, USA

RaySearch recently released a Monte Carlo Dose calculation (MDC) engine in the RayStation treatment planning system replacing the Analytical Pencil Beam Algorithm (APB). The three major problems that the MDC is attempting to solve are the accuracy of lung dose calculations and improved dose calculations for apertures and range shifters.

Three types of phantoms were used to validate the MDC engine in near realistic clinical settings. We used animal neck, water breast and realistic lung phantoms. The lung phantom comprises of a real rack of lamb to constitute the patient's ribs, followed by layers of cork to simulate lung tissue. The tumor was simulated with two halve domes of 70% lean ground meat. Dose distribution measurements in the phantoms were made using the MatriXXPT device, EBT GafChromic film and ionization chambers.

The measured data compared exceptionally well with both the APB and MDC for the neck and breast phantoms while the MCD produced extremely accurate calculations in lung. For range shifters, the MDC doses agreed with the measured doses within + 1% while the APB algorithm overestimated the dose by up to 10%. The average difference between measured and expected ranges for APB and MDC were 0.6% and 0.5% for the neck phantoms and 2.9% and 0.9% for the breast phantom and 0.8% for the MDC in lung. The APB calculation in lung was not accurate enough to derive a range uncertainty.

This work revealed extremely important findings. APB calculations should be used with great caution in the clinic.

### O 133: Harmonization of the Treatment Planning Approach for Head-And-Neck Cancer Using Pencil Beam Scanning: First Report of the IPACS Collaboration Group

#### M. Stock^1^, E. Hug^2^, T. Kajdrowicz^3^, K. Kisielewicz^4^, K. Skowrońska^3^, V. Vondracek^5^, J. Kubes^6^, Z. Poulova^5^, A. Bäck^7^, M. Gustafsson^7^, M. Sooaru^7^, E. Gora^4^, M. Ptaszkiewicz^3^, J. Gora^1^, A. Perpar^2^, T. Björk-Eriksson^8^

##### ^1^EBG MedAustron GmbH, Medical Physics, Wiener Neustadt, Austria, ^2^EBG MedAustron GmbH, Radiation Oncology, Wiener Neustadt, Austria, ^3^The Henryk Niewodniczanski Institute of Nuclear Physics, Cyclotron Centre Bronowice, Krakow, Poland, ^4^Maria Sklodowska-Curie Memorial Cancer Center and Institute of Oncology, Radiation Oncology, Krakow, Poland, ^5^Proton Therapy Centre Czech, Medical Physics, Prague, Czechia, ^6^Proton Therapy Centre Czech, Radiation Oncology, Prague, Czechia, ^7^Sahlgrenska University Hospital and Skandion Clinic, Department of Therapeutic Radiation Physics, Uppsala, Sweden, ^8^Sahlgrenska University Hospital and Skandionclinic, Regionalt Cancercentrum Väst, Uppsala, Sweden

**Background and purpos**e: This IPACS collaboration has the goal of harmonizing proton therapy (PT) with the aim of increasing the possibility to draw conclusions from upcoming multicenter studies. This abstract reports on the collaborative network amongst proton centers in Ialy, Poland, Austria, Czech Republic and Sweden and the aim to harmonize PT treatment planning for head-and-neck cancer.

**Methods**: Five CT-data sets of patients previously treated at PTC Prague and suffering from head and neck tumors were included. During several on-site meetings and video conferences the treatment planning protocol was developed. The protocol included objectives of target coverage and organ at risk (OAR) sparing together with their mutual priorization as well as a definition of robustness evaluation. Each center used its own TPS and planning approach. Plans from all centers were discussed and plan parameters were compared. In addition, VMAT photon plans were created by one center for comparison.

**Results and Conclusion**: Despite the very detailed treatment planning protocol, differences in the dose distribution and dose parameter values were identified (e.g. see Fig. 1&2). Different interpretation of robustness and its evaluation was the most discussed issue. The most homogeneous parameter was the median dose to the targets. Cases of poor target coverage for the boost volumes were caused by center specific concerns of robust OAR sparing (see Fig. 2). This is the first report of the IPACS group demonstrating current status of harmonization efforts in treatment planning for head-and-neck cancer.

### O 134: Testing Dose Calculations with Stopping Power Ratio Images Constructed from Dual-Energy CT in a Commercial Treatment Planning System

#### J. POLF^1^, S. Mossahebi^1^, H. Chen^1^, U. Langner^1^, M. Zhu^1^, P. Wang^1^, J. Eley^1^, K. Langen^1^, D. Strauss^1^, M. Michelle^1^

##### ^1^University of Maryland School of Medicine, Maryland Proton Treatment Center- Radiation Oncology, Baltimore, USA

**Purpose**: To investigate the use of stopping power ratio (SPR) images, created from electron density (re) and effective atomic number (zeff) images (from dual-energy CT (DECT) images) for proton dose calculation in a clinical proton treatment planning system (TPS).

**Methods**: SPR images of 26 tissue surrogate plugs inserted into a tissue equivalent phantom were constructed and imported into a clinical TPS using the procedure shown in Figure 1. A single field treatment plans was created using both the SPR and SECT images for each tissue surrogate plug scan. To assess accuracy of the dose calculation on SPR images (and the SECT images), the treatment plans were delivered to each phantom/plug, and ion chamber and 2D planar dose measurements were compared to a dose reference point and 2D planar doses in the treatment plans as described in Figure 1.

**Results**: For all tissue surrogate materials, TPS calculated dose on the SPR and SECT images were within 1.0% and 1.5% of ion chamber measurements, respectively, except for lung-exhale (1.5% SPR, 4.5% SECT).SPR and SECT plan 2D-gamma pass rates (±3% and 3-mm criteria) were greater than 90% for both SPR and SECT plans for all tissues expect bone (∼55%).

**Conclusion**: The use of SPR maps, constructed from DECT images, for proton dose calculation with a clinical TPS was shown to be accurate within clinical criteria (±3%/3 mm) for most tissue surrogates studied. TPS plans in heterogeneous phantoms showed clinically relevant improvements when using SPR-maps compared to standard SECT images.

### O 135: Experimental Validation of Dual Energy CT Based Proton Stopping Power Ratio Estimation in Organic Tissues

#### V. Taasti^1^, G. Michalak^2^, D. Hansen^1^, A. Deisher^3^, J. Kruse^3^, B. Kraus^4^, L. Muren^1^, J. Petersen^1^, C. McCollough^2^

##### ^1^Aarhus University / Aarhus University Hospital, Department of Medical Physics, Aarhus, Denmark, ^2^Mayo Clinic, Department of Radiology, Rochester, USA, ^3^Mayo Clinic, Department of Radiation Oncology, Rochester, USA, ^4^Siemens Healthineers, Siemenstraße 1, Forchheim, Germany

**Purpose**: Dual-energy CT (DECT) has been shown, in theoretical and phantom studies, to improve the stopping-power ratio (SPR) estimation compared to single-energy CT (SECT). This study validated DECT-based SPR estimation in organic tissues by comparison to proton measurements.

**Methods**: Fourteen tissue samples, including fat, muscle and high-density femur bone, were tightly packed in plastic boxes to avoid air pockets. The tissue samples were CT scanned at clinical dose levels, with each sample scanned separately, immersed in a water tank. Four DECT scanners (Siemens Healthcare, Forchheim, Germany) with different dual-energy acquisition modes were used, Dual Source, Consecutive Scanning, and Split-Filter. In total, six DECT-based SPR estimations were performed for each sample using the Siemens syngo.via Rho/Z algorithm for extracting electron densities and effective atomic numbers. SECT images were also acquired, and SECT-based SPR estimations were derived using a clinical conversion curve. Subsequently, the reference SPRs for the samples were measured using 185 MeV proton pencil beams and a Multi-Layer Ionization Chamber.

**Results**: The root-mean-square-errors (RMSEs) of the DECT-based SPR estimates across tissue samples ranged from 0.9% to 1.5%, compared to 2.8% for SECT. Split-Filter DECT showed a mean error of 1.3% (positive bias). The highest accuracy was obtained using Dual Source DECT. The largest errors were found for the high-density cortical bone from a beef femur.

**Conclusion**: This study showed that DECT improved the SPR accuracy for organic tissues compared to SECT, indicating that DECT can reduce range uncertainty margins in proton treatment planning.

### O 136: Radiation Dose-Painting with Protons vs. Photons for Head-And-Neck Cancer

#### K. Håkansson^1^, B. Smulders^1^, L. Specht^1^, M. Zhu^2^, J. Friborg^1^, J. Rasmussen^3^, S. Bentzen^4^, I. Vogelius^1^

##### ^1^University Hospital of Copenhagen / Rigshospitalet, Department of Oncology, Copenhagen, Denmark, ^2^University of Maryland School of Medicine, Department of Radiation Oncology, Baltimore, USA, ^3^University Hospital of Copenhagen / Rigshospitalet, Department of Otorhinolaryngology- Head & Neck Surgery and Audiology, Copenhagen, Denmark, ^4^University of Maryland School of Medicine, Division of Biostatistics and Bioinformatics- University of Maryland Greenebaum Cancer Center and Department of Epidemiology and Public Health, Baltimore, USA

**Background and purpose**: Dose-painting has recently been investigated in early-phase trials of head-and-neck cancer (HNC) with the aim of improving local tumor control. At the same time proton therapy has been reported as potentially capable of decreasing toxicity. Here, we investigate whether protons could be applied in a dose-painting setting by comparing proton dose distributions with delivered photon plans from a phase-I trial of FDG-PET based dose-painting at our institution.

**Material and Methods**: Fifteen patients from the recently conducted phase I trial were used for comparison of proton and photon dose-painting techniques. Robust optimization was used for proton plans. Plan robustness and difference in dose metrics to targets and organs at risk were evaluated.

**Results**: The proton plans fulfilled target dose constraints, while having lower non-tumor dose than photon plans (body-minus-CTV, mean dose 3.9Gy vs 7.2Gy, p=0.004). Despite the use of robust proton planning for max dose, photon plan max doses were more robust (p=0.006). There was no significant difference in parotid dose between proton and photon plans (p=0.745).

**Conclusions**: Proton dose-painting for HNC seems feasible and can reduce the non-tumor dose overall, however not to certain organs close to the target, such as the salivary glands. Great care should be taken in terms of max dose robustness.

### O 137: Alignment and Tracking Algorithms for the MIMOSA28 Detectors for Ion Radiotherapy

#### C.A. Reidel^1,2^, C. Finck^3^, C. Schuy^1^, U. Weber^1^

##### ^1^GSI, Biophysics, Darmstadt, Germany, ^2^Strasbourg University, Physics, Strasbourg, France, ^3^IPHC, Hadrontherapy, Strasbourg, France

Heavy charged particles as proton and carbon (12C ion) are used for the treatment of deep-seated tumors due to their favorable depth-dose profile (Bragg curve). In proton therapy, target fragments are produced with a low energy and a short range. For 12C ion, the projectile fragmentation creates long range fragments which induce a dose in healthy tissues after the tumor.

For an accurate measurement of the angular distribution of the produced fragments, a tracking with high resolution is needed. The MIMOSA28 is a pixel detector based on CMOS technology. It is composed of 928 rows x 960 columns with a pixel size of 20.7μm and has a readout time of 186.5μs.

To reach a track resolution under 5μm, the sensors need first to be aligned. An alignment algorithm has been implemented in the data analysis with the minimization of a complex chi square, based on a linear regression analysis. This algorithm was tested with proton beam in the range of 70 to 220MeV and has shown very encouraging results (Figure 1 - Beam position measured with 6 sensors before and after the alignment procedure).

Additionally, good track efficiency and low fake track reconstruction are important. Even with a small sensor thickness (<50μm) and placing the sensors as close as possible, the scattering of the particles stays significant. The sensors placement influences the track reconstruction. To minimize this influence, a tracking algorithm, taking into account the scattering of the particle, has been implemented. The first tests have shown promising results.

### O 138: Feasibility Evaluation of Proton-based, Spatially Fractionated Radiotherapy (SFRT) Using a GRID Technique

#### J. Yu^1^, D. DeBlois^1^, M. Hall^1^, M. Chuong^1^, M. Mehta^1^, M. Fagundes^1^, A. Gutierrez^1^

##### ^1^Miami Cancer Institute, Radiation Oncology, Miami, USA

**Purpose**: One of the limitations of the well-established SFRT using a GRID technique is the increased dose to proximal normal tissue. This study is to investigate the feasibility of a pencil beam scanning (PBS) proton GRID SFRT without a physical grid and its potential dosimetric benefits.

**Methods**: A patient with an iliac lesion was treated with a compensator-based GRID radiotherapy with a single 6 MV field. The prescribed dose was 15 Gy (1Fx) at dmax. Using the same CT data, a single-field proton PBS plan was created without a physical grid, which eliminates the creation of secondary neutrons. The proton and photon plans were compared in terms of dose distribution, accumulative DVH, differentiate DVH, and dose profiles perpendicular to the beam.

**Results**: The proton PBS GRID plan mimicked the dose distribution of the photon plan (Figure 1a & b). Cumulative DVHs show higher dose to the GTV for the proton plan and significant reduction in dose to the proximal normal tissue and total integral dose (Figure 2(a) and Table 1). For the proton plan, the differential DVH indicates higher spatial fractionation (Figure 2(b)), and a dose profile across the center of the GTV validates the improved contrast of dose peaks and valleys (Figure 2(c)).

**Conclusions**: The proton PBS system is able to generate a GRID-like dose distribution similar to the photon GRID plan, but with higher target dose coverage, improved peak- valley dose fall-off, and lower normal tissue dose compared to photon-based GRID. Further studies are warranted to better characterize the proton GRID technique.

### O 139: MR-only IMPT Planning for Prostate Cancer: A Proof of Concept

#### N. Depauw^1^, J. Keyrilainen^2^, S. Suilamo^2^, K. Bzbusek^3^, L. Warner^4^, C. Olsen^1^, H. Kooy^1^

##### ^1^Massachusetts General Hospital, Radiation Oncology, Boston, USA, ^2^Turku University Hospital, Department of Medical Physics, Turku, Finland, ^3^Philips Healthcare, Philips Radiation Oncology Systems, Fitchburg- WI, USA, ^4^Philips Healthcare, Clinical Science MR Therapy, Vantaa, Finland

Treatment planning for proton therapy requires the relative proton stopping power ratio (RSP) information of the patient for accurate dose calculations. RSP are conventionally obtained after careful mapping of the Hounsfield units (HU) from a calibrated patient CT. One or multiple CT are needed for a given treatment which represents additional, undesired dose to the patient. Not only MRI offers dose-less imaging but, for prostate cancer, it also provides better contrasts, hence higher contouring accuracy.

MRCAT (Magnetic Resonance for Calculating ATtenuation) is a Philips-developed technology which produces a simplified CT-simile image consisting of four HU from a specific set of MR acquisitions. For ten patients, an MRCAT set was created along with MR-based contours. An intensity modulated proton plan was generated on the MRCAT for each patient. Plans were produced such that they fulfill the prostate protocol in use at Massachusetts General Hospital (MGH). The plans were then recomputed onto the nominal “real” planning CT for each patient. Robustness analyses (5 mm setup shifts and 3.5 % range uncertainties) were also performed. Figure 1: MRCAT (top left/bottom right) overlaid on planning CT (bottom left/top right).

Comparison of MRCAT plans and their recomputation onto the planning CT plan showed excellent agreement. Likewise, dose perturbations due to setup shifts and range uncertainties were well within clinical acceptance. This novel approach highlights the possibility for IMPT treatment planning of prostate cancer using MR-only information, hence providing higher contouring accuracy without the need for additional imaging dose. Figure 2: Nominal DVH (solid) versus recomputation (dashed).

 

## Poster Presentations

### P 003: The Difference of Radiation Sensitivity between X-Ray and Carbon Ion Beam in Castration-Resistant Prostate Cancer

#### M. Iwanaga^1^, H. Kawamura^1^, N. Kubo^1^, T. Mizukami^1^, H. Sato^1^, Y. Sekine^2^, K. Suzuki^2^, R. Kawabata^3^, M. Nishiyama^3^, T. Nakano^1^

##### ^1^Gunma University Graduate School of Medicine, Radiation Oncology, Maebashi, Japan, ^2^Gunma University Graduate School of Medicine, Urology, Maebashi, Japan, ^3^Gunma University Graduate School of Medicine, Molecular Pharmacology and Oncology, Maebashi, Japan

**Objective**: Castration-resistant prostate cancer (CRPC) shows resistance to not only androgen deprivation therapy but also X-ray therapy. However, the mechanism has not been elucidated. Carbon ion beam (CIB) has a high biological effect and is used for various cancers showing resistance to X-ray therapy. The purposes of this study were to clarify the difference in sensitivity of X-ray and CIB to CRPC and to examine its mechanism.

**Method**: The androgen-independent prostate cancer cell line LNCaP-LA established by culturing androgen-sensitive prostate cancer cell line LNCaP for 2 years in androgen-free medium. Colony formation assay was carried out to investigate the sensitivity of X-ray and CIB. DNA mutation analysis on 409 cancer-related genes and comprehensive transcriptome analysis (RNA-seq) were performed with the next generation sequencer.

**Results**: Lethal Dose 50 % (LD 50) of X-rays in LNCaP and LNCaP-LA were 1.4 Gy and 2.8 Gy, respectively (p <0.01). The LD 50 for CIB were 0.9 Gy and 0.7 Gy, respectively (p = 0.09). In DNA mutation analysis, AR mutation was observed specifically in LNCaP-LA. From RNA-seq, 181 genes showing significant expression (FDR < 0.10, p < 0.00076) in LNCaP-LA were extracted. Based on these results, pathway analysis suggested that NRF2-pathway, which is involved in intracellular oxidative stress prevention, was activated in LNCaP-LA.

**Conclusion**: LNCaP-LA showed X-ray resistance compared with LNCaP and showed sensitivity to CIB. The AR mutation and NRF2-Pathway were suggested as a cause of resistance.

### P 004: Mitochondrial Fragmentation Level in Hela Cells Exposed to X-Rays and Carbon Ions and Its Influence on Cellular Apoptosis

#### X. Jin^1^

##### ^1^Institute of Modern Physics IMP-Chinese Academy of Sciences CAS, Division of Radiation Medical Physics, Lanzhou, China

DNA damage is usually considered to be the main consequence of cancer cells exposed to ionizing radiation. Although mitochondria are known to play an important role in radiation-induced cellular damage, the mechanisms by which ionizing radiation modulates mitochondrial dynamics are largely unknown. In this study, human cervical carcinoma cell line HeLa demonstrated two different modes of mitochondrial network in response to different quality radiations. Mitochondria became fragmented into punctate and clustered upon carbon ion irradiation in a dose-dependent manner, which was associated with apoptotic cell death. In contrast, low-dose X-ray irradiation promoted mitochondria fusion while mitochondria fission was detected until the radiation dose was more than 1 Gy. Inhibition of mitochondrial fragmentation using Drp1 inhibitor suppressed the radiation-induced apoptosis and thus enhanced the resistance of cells to carbon ions and high-dose X-rays, but not for cells irradiated with X-rays at the low dose. Our results suggest that radiations of different qualities cause diverse changes of mitochondrial dynamics in cancer cells, which play an important role in determining the cell fate.

### P 005: Physical Model and Molecular Probe toward the Origins of RBE: Physics to Chemistry to Biology

#### K. Kinoshita^1^, E. Merino^2^, Y. Zabarmawi^1^, G. Premnauth^2^, M. Lamba^3^

##### ^1^University of Cincinnati, Physics, Cincinnati, USA, ^2^University of Cincinnati, Chemistry, Cincinnati, USA, ^3^University of Cincinnati, Radiology, Cincinnati, USA

We present a new and fundamental statistical model of ionization response to charged particle radiation that predicts rates of clustered ionization in DNA. The model is promising as a vehicle toward understanding processes of biological damage, from ionization/excitation to biological outcome, through direct and detailed validation with experimental data at intermediate points, from naked DNA to tissue in vivo. We are developing relevant measures of DNA damage using Liquid Chromatography-Mass Spectrometry and will analyze the results in the context of the model.

The effects of charged particle radiation on biological systems vary primarily according to ionization density, or Linear Energy Transfer (LET). It is well known that permanent impairment to DNA requires more than a single ionization, due to the remarkable resilience of the molecule and cellular repair mechanisms. Physical modeling of more complex ionization phenomena has failed to consistently predict biological endpoints. Historically, clustering models have been based on low-LET observations, likely inadequate for high LET. In addition, different metrics of biological effect give different RBE values, indicating that different endpoints are triggered by different criteria. Our model distinguishes by cluster size, thus providing a rich framework for exploring relationships of various metrics to dose, LET, and dose rate. We discuss the experimental conditions under which measurements are interpretable under our model and analyze the correspondence for several examples, including published data and preliminary results from exposures of dry DNA, and cells to photons and protons at the Cincinnati Children's Hospital/UC Health Proton Therapy Center.

### P 006: Repair of Radiation Induced Damages of V79 Cells after Carbon Ions Exposure with Different LET

#### E.V. Koryakina^1^, M.V. Troshina^1^, V.I. Potetnya^1^, R.M. Baikuzina^1^, M.N. Efimova^1^, S.N. Koryakin^1^, A.A. Lychagin^1^, S.E. Ulyanenko^1^

##### ^1^A. Tsyb Medical Radiological Research Center – branch of the National Medical Research Radiological Center of the Ministry of Health of the Russian Federation, Radiation Biophysics, Obninsk, Russian Federation

High effectiveness of carbon ion radiotherapy has been clinically demonstrated. However, normal tissues responses to differing radiation spectra along a carbon ions Bragg curve, including its plateau, peak, post peak, are of great interest regarding both early and late possible effects. Repair of radiation-induced cell damages after carbon ions exposure is one of the most considerable radiobiological and clinical parameters. It depends on number of damages and LET of radiation and determines cell killing or cell surviving. Post-irradiation repair of damages induced by carbon ions (454 MeV/nucleon, U-70 accelerator, IHEP, Protvino) in Chinese hamster V79 cells was studied. Flasks with cells monolayer in the stationary growth phase were irradiated in a water phantom either in the plateau (LET ∼10-12 keV/μm) or in the peak (LET ∼180 keV/μm) of the pristine Bragg curve, thus simulating damaging normal tissues and tumor cells, respectively. The carbon doses were 6 Gy for the plateau and 1, 2, 4 Gy for the Bragg peak. During and after irradiation cells were in pure medium, after irradiation they were kept at 37°C for 0, 0.5, 1, 2, 4, 6, 8, 10, 12 h before reseeding in full medium, and incubated for 8-10 days for colony formation. No repair of damages induced by carbon ions in stationary V79 cells was observed during incubation times studied for each dose both at the plateau and the Bragg peak. The plateau data might be due to non optimal conditions for post-irradiation repair and are of interest for future studies.

### P 007: Difference in SLDR and DNA Damage Repair after Split-Irradiation with Proton Beams and X-Rays

#### Y. Matsumoto^1^, K. Ando^2^, T. Kato A^3^, Y. Sekino^1^, T. Sakae^1^, H. Ishikawa^1^, K. Tsuboi^1^, H. Sakurai^1^

##### ^1^University of Tsukuba, Proton Medical Research Center, Tsukuba, Japan, ^2^Gunma University, Heavy Ion Medical Center, Maebashi, Japan, ^3^Colorado State University, Department of Environmental & Radiological Health Sciences, Colorado, USA

Proton beam can accumulate the dose to tumor with an excellent depth-dose distribution (Bragg peak), and is beginning to spread worldwide as the most advanced radio-therapeutic modality. However, fundamental biological study, for example, sub-lethal damage recovery (SLDR) after proton clinical beam is not known yet. We here conducted split dose experiments (20-360 min intervals) to clarify SLDR kinetics, and also compared the kinetics between cells with different repairability of DNA double-strand breaks. CHO and 51D1 cell lines but not V3 cell line showed significant SLDR, which reached plateau in 4-6 h. The recovery rates and repair halftime of SLDR after X-rays were significantly higher and shorter than proton beams for CHO and 51D1 cells, respectively. Additionally, the frequency of remained γ-H2AX foci after 2 fractions was remarkably higher for X-rays than proton beams. These data suggest that there is a difference between proton beam and X-rays in SLDR and the retained DNA double strand breaks after split-dose irradiation.

### P 008 - Cellular and Molecular Examinations of Squamous Cell Carcinoma Cell Lines of the Head and Neck to 12C Irradiation

#### B. Sishc^1^, L.H. Ding^1^, D. Saha^1^, A. Aroumougame^1^, B. Chen^1^, A. Facoetti^2^, M. Ciocca^3^, A. Pompos^1^, A. Davis^1^, M. Story^1^

##### ^1^UT Southwestern Medical Center, Radiation Oncology, Dallas- TX, USA, ^2^National Center of Oncological Radiotherapy, Radiation Biology, Pavia, Italy, ^3^National Center of Oncological Radiotherapy, Physics, Pavia, Italy

Squamous Cell Carcinoma (SCC) is a histologically distinct form of cancer originating from epithelial cells within the lining of hollow organs of the body or the skin, including the head and neck, esophagus, lung, prostate, cervix and bladder amongst others. SCCs can be very aggressive and generally have a poor prognosis even after surgery followed by radiation and chemotherapy. One rationale for the use of 12C radiotherapy is the treatment of radioresistant disease. We therefore examined the 12C radioresponse of six head and neck SCC cell lines, whose surviving fraction at 2 Gy ranged from average to resistant when compared to a larger panel of 49 cell lines, to determine whether 12C irradiation can overcome X-ray radioresistance and whether there may be tumor biomarkers predictive of 12C radioresponse. Therefore, these cell lines were also chosen for their genetic backgrounds that included mutations in the TP53 and LIG4 genes and the expression of EGFR, amongst other alterations. Cells were irradiated with 12C using a spread-out Bragg peak with an average LET of 75 keV/μm generated at the CNAO (Pavia, Italy) facility. RBE values at 10% survival ranged from 1.8-2.6 with an average RBE of 2.21. The initial survival results under hypoxic conditions revealed an oxygen enhancement ratio of ∼2 at 10% survival. DNA damage foci resolution suggested that unrepaired, complex double strand breaks contribute to an enhanced RBE and decreased OER compared to photons. A comparative analysis of gene expression post-12C exposure vs g-Rays is ongoing and will be reported.

### P 009: Impact of BRCA1 Deficiency and Combination with PARP Inhibitors on the Response of Breast Cancer Cells to Proton Radiation

#### M. Sertorio^1^, F. Zhang^1^, L.A. Runck^1^, A. Mascia^2^, J.P. Perentesis^1^, S.I. Wells^1^, Y. Zheng^1^, P.R. Andreassen^1^

##### ^1^Cincinnati Children's Hospital Medical Center, Cancer and Blood Diseases Institute, Cincinnati- OH, USA, ^2^University of Cincinnati, Radiation Oncology, Cincinnati- OH, USA

BRCA1 is a key mediator of homologous recombination (HR) and is the most frequent cause of hereditary breast and ovarian cancer when mutated. Loss of function of BRCA1 is also a determinant of the response of tumor cells to therapy, including photon radiation and poly (ADP-ribose) polymerase (PARP) inhibitors. Our goals here are to determine whether BRCA1 is recruited to sites of DNA damage in response to protons in breast cancer cells, to elucidate whether mutation of BRCA1 increases the sensitivity of breast cancer cells to protons, as a basis for personalized medicine, and to explore the effectiveness of combining protons and PARP inhibitors (PARPi). For this purpose, we utilize HCC1937 BRCA1-deficient breast cancer cells, with or without genetic complementation, and a panel of breast cancer cells in which we knock-down BRCA1 using a shRNA. In a side-by-side comparison, our results indicate that BRCA1 is recruited to DNA damage foci in response to protons but that these foci may persist longer than foci induced by photons. Importantly, initial studies using colony growth assays suggest that deficiency for BRCA1 in various breast cancer cells causes increased sensitivity to protons. Thus, proton radiation could be particularly effective in treating breast cancers with genetic deficiencies for HR genes, including BRCA1. Finally, results from measuring levels of DNA double-strand breaks, as determined using γH2AX foci (Fig. 1), indicate that combining proton radiation and PARPi may offer an effective personalized treatment which exploits BRCA1 deficiencies that result from either germline or somatic mutations.

### P 010 - Radio-sensitization of Chondrosarcoma Cells by PARP-inhibitors

#### F. Chevalier^1^, M. Césaire^1^, Y. Saintigny^1^

##### ^1^CEA, DRF - iRCM - LARIA / GANIL, Caen, France

Chondrosarcoma is a malignant tumor arising from cartilaginous tissue and presenting radio- and chemo- resistances to conventional treatments. The main treatment first consists to a surgical resection, which may conduct to severe disabilities for the patient; in addition, this procedure may not be possible for inoperable locations such as the skull base. Carbon-ion irradiation (hadron-therapy) is proposed as an alternative treatment, due notably to a higher biological effectiveness and a better ballistic as compared with conventional radiotherapy with X-rays. This study aimed to examine the cellular responses of chondrosarcomas to conventional radiotherapy and hadrontherapy, associated with PARP-inhibitors in order to better understand the biological effects of combined treatments. Three human chondrosarcoma cell lines of different grades and displaying differential radio-sensitivities, were irradiated with photons, proton or carbon ions, in association with different PARP inhibitors.

To better understand the PARP-inhibition process, we first analyzed Poly-ADP ribose chains formation using western blotting, and we observed a maximum of signal after irradiation with CH2879 cells. As attempted, clonogenic assays confirmed the better biological effectiveness of carbon ion over X-rays; the resistance to radiation of each cell line was calculated from the corresponding survival curves. PARP-inhibitors increase radio-sensitivity, with a factor depending of the dose and irradiation quality. Cell death was increased following treatments, as observed by flow cytometry and western blotting experiments.

This study demonstrates the capacity of PARP-inhibitors in radio-sensitizing chondrosarcoma cells, using conventional photon irradiation as well as using proton beam and carbon beam irradiation.

### P 011: Genistein Sensitizes Glioblastoma Cells to Carbon Ions via Inhibiting DNA-Pkcs Phosphorylation and Subsequently Repressing NHEJ and Delaying HR Repair Pathways

#### X. Liu^1^

##### ^1^Institute of Modern Physics- Chinese Academy of Sciences, Medical physics, Lanzhou, China

DNA-dependent protein kinase catalytic subunit (DNA-PKcs) plays a critical role in non-homologous end joining (NHEJ) of DNA double-stand break (DSB) repair. Previously, we found genistein could sensitize cancer cells to low linear energy transfer (LET) X-rays via inhibiting DNA-PKcs activities. Especially, high-LET heavy ion produces more DNA DSBs than low-LET radiation. Thus, it is more important to study the detailed molecular mechanisms of genistein on sensitizing cancer cells to heavy ions. Colony formation assays showed that genistein sensitized DNA-PKcs proficient but not deficient glioblastoma cells to high-LET carbon ions. The sensitizer enhancement ratio of genistein was similar to that of DNA-PKcs specific inhibitor Nu7026 treatment or siRNA knockdown of DNA-PKcs. Immunofluorescence and western blot experiments were performed to examine the DNA DSBs and their repair kinetics in two DNA-PKcs proficient glioblastoma cell lines. The current studies showed that genistein delayed the clearance of γ-H2AX foci induced by carbon ions; moreover, it physically bound to DNA-PKcs and functionally inhibited the level of p-(T2609)-DNA-PKcs. So the kinase of DNA-PK was not activated and the NHEJ repair pathway would be prevented. Furthermore, although genistein did not decrease Ku70/80 induced by carbon ions, p-(T714)-Ku80 decreased significantly. The Ku70/80 heterodimer preferentially bound to but not dissociated from the DSB site, which delayed the recruitment of Rad51 to the DSB ends, so that the homologous recombination (HR) repair was repressed. Thus, our study demonstrated that genistein holds promise as a radiosensitizer for enhancing the efficacy of carbon ion radiotherapy via inhibiting NHEJ and delaying HR repair.

### P 012: Proton-Boron Fusion Reaction Increases Proton Radiobiology Effectiveness

#### G. Petringa^1,2^, L. Manti^3,4^, F.P. Cammarata^1,5^, G. Cuttone^1^, G. Korn^6^, D. Margarone^6^, P. Pisciotta^1,2^, A. Picciotto^7^, L. Giuffrida^6^, G.A.P. Cirrone^1,6^

##### ^1^Istituto Nazionale di Fisica Nucleare INFN, Laboratori Nazionali del Sud LNS, Catania, Italy, ^2^Università degli Studi di Catania, Dipartimento di Fisica e Astronomia, Catania, Italy, ^3^Istituto Nazionale di Fisica Nucleare INFN, Sezione di Napoli- Complesso Universitario di M. S. Angelo, Naple, Italy, ^4^Università degli Studi Federico II di Napoli, Dipartimento di Fisica “E. Pancini”, Naple, Italy, ^5^National Research Council CNR, Institute of Molecular Bioimaging and Physiology IBFM, Catania, Italy, ^6^ELI-Beamline Project, Inst. Physics- ASCR- PALS Center, Prague, Czech Republic, ^7^Fondazione Bruno Kessler, Microtechnology Laboratory, Trento, Italy

Proton therapy is a pillar in the battle against cancer but the higher Relative Biological Effectiveness (RBE) of 12C-ions can overcome cancer radioresistance. Thus, enhancing proton RBE is desirable. To this end, we exploited the 11B(p,a)2a nuclear fusion reaction to generate high-LET alpha particles with a clinical proton beam. To maximize the reaction rate, we used sodium borocaptate (BSH) with natural boron content. Boron-treated cells were irradiated at mid-SOBP (Spread Out Bragg Peak) depth and assayed for clonogenic survival and DNA damage induction We recorded significantly increased cellular lethality and occurrence of chromosome aberrations. Specifically, we proved that, if human cells are irradiated with a given amount of 11B the interaction with protons results in an increase of almost a factor 2 in cell killing compared to boron-free irradiated controls (see Figure 1). The findings suggest that the effect is due to the generation of low-energy, high LET alpha particles since we measured a marked increase in the complexity of DNA damage. The alphas produced have a sufficient range to come to a halt and release almost their entire energy in the cell nucleus by which severe damage is induced to the DNA, thereby enhancing the biological efficacy of the proton beam.

The presence of Boron atoms and of the associated gamma-prompt emissions generated from their interaction with protons, could also give an added value to this approach giving the possibility of the treatment on-line verification. (Figure1: Clonogenic dose response curves of DU145 irradiated with protons in the presence of BSH at mid-SOBP).

### P 013: The Radiosensitizing Effects of Differently-Modified Gold Nanoparticles on B16-F10 Cells at the Same Cellular Uptake Concentration under Carbon Ion Irradiation

#### P. Zhang^1^, Y. Liu^1^, X. JIn^1^, W. Chen^1^, Q. Li^1^

##### ^1^Institute of Modern Physics- Chinese Academy of Sciences, Department of Medical Physics, Lanzhou, China

Cellular uptake has a great significance for the radiosensitizing effect of gold nanoparticles (AuNPs). Similarly, differently-modified AuNPs at the same gold cellular uptake also influence the radiosensitizing effect. In our recent study, we investigated the cellular uptake of different AuNPs capped with citrate,11-mercaptoundecanoic acid (11MUA) and glutathione (GSH) in mouse melanoma B16-F10 cells and compared the radiosensitizing effects of differently-modified AuNPs at the same cellular uptake concentration after exposure to carbon ions (LET=50keV/μm). B16-F10 cells were co-cultured with citrate-, 11MUA- and GSH-capped AuNPs at various concentrations. 24h after the co-culture, the cells were washed with PBS buffer three times and harvested using trypsin. The cell number was counted using a particle counter. Then, ICP-AES was used to calibrate the concentration of gold in cells after the cells were centrifuged and dissolved by aqua regia. The gold concentration was normalized to single cells by using the cell number. In this way, the dependence of cellular uptake on the co-culture concentration of the differently-modified AuNPs in B16-F10 cells was obtained (Fig.1). B16-F10 cells showed the same gold cellular uptake 2pg/cell at 6, 13 and 30μg/ml co-culture concentrations for 11MUA-, citrate and GSH-capped AuNPs, respectively. Moreover, B16-F10 cells were co-cultured with the differently-capped AuNPs at the equal gold cellular uptake 2pg/cell for 24h, and then exposed to carbon ion irradiation. After irradiation, colony formation assays were conducted and cell survival data were acquired (Fig.2). This work provides a substantial basis for exploring the mechanisms underlying the radiosensitizing effects of differently-modified AuNPs.

### P 014: Study of Bystander Effects in Chondrosarcoma Cells Irradiated In Vitro with X-Rays and Carbon Ions

#### F. Chevalier^1^, C. Lepleux^1^, A. Marie-Brasset^1^, Y. Saintigny^1^

##### ^1^CEA, DRF - iRCM - LARIA / GANIL, Caen, France

Hadrontherapy with carbon ions seems to be a good alternative for the treatment of cancer resistant to conventional radiotherapy. Chondrosarcoma is a candidate for hadrontherapy because this cancer is radio and chemo resistant, and can be un-operable, when located in the skull base. However, it is necessary to evaluate the secondary effects of this irradiation type, especially the interaction between irradiated and non-irradiated cells. Among these effects, the radiation-induced bystander effect involves stress signals emitted by irradiated cells adjacent or very close to non-irradiated cells; bystander molecules can induce a biological response with damages usually observed with irradiated cells.

To study this phenomenon, we used a protocol of medium transfer. Cells are irradiated with X-rays or carbon ions and then the bystander supernatant, containing the signals emitted by irradiated cells, is transferred on non-irradiated cells.

Chondrosarcoma cells and chondrocytes were analyzed as emitting and/or receptor cells of bystander signals. We use different technical strategies, such as clonogenic assay, to study the survival cells fraction after treatment; multiplex Elisa analysis of conditioned medium for the identification of bystander factors and flow cytometry for cell cycle analysis of irradiated and bystander cells.

Our results showed a significant reduction of chondrocyte survival after transfer of conditioned medium from chondrosarcoma cells irradiated with low doses of X-rays and C-ions. By diluting this medium, the phenomenon decreased proportionally, confirming the presence of bystander factors. Some of these factors were partially observed using multiplex analysis of cell cytokines.

### P 015: Side Effects of Scattered Versus Scanned Proton Beams on Normal Tissues in Total Body Irradiated Mice: Preliminary Results

#### S. Chaouni^1^, L. De Marzi^2^, F. Pouzoulet^3^, J.L. Habrand^4^, F. Sichel^1^, D. Stefan^4^, D. Lecomte^4^, C. Laurent^5^

##### ^1^ABTEEA4651-ToxEMAC, Université de Caen- CLCC François Baclesse, Caen, France, ^2^Institut Curie, Centre de Protonthérapie, Orsay, France, ^3^Institut Curie, Département de Recherche Translationnelle- Plateforme de Radiothérapie Expérimentale, Orsay, France, ^4^CLCC François Baclesse, Radiotherapy, Caen, France, ^5^ARCHADE-SAPHYN- ABTEEA4651-ToxEMAC, Université de Caen- CLCC François Baclesse, Caen, France

Protons are now widely used instead of photons due to their ballistic properties which should allow to treat tumors localized near organs at risk (OAR) without leading to toxicities in these OAR. Nowadays, the state of knowledge is limited mainly to relative biological efficiency (RBE) on observables related to cell death. Moreover, difficulty of access to beam line facilities, initially dedicated to physics research, and their inadequacy with radiobiology experiments have limited the quantity and the quality of available and homogenous biological data. Therefore, only few studies have been performed on proton effects on normal tissues or cells in comparison with the large number of studies on conventional radiotherapy. In this way, there is a lack of data relative to biological effects of scattered versus scanned proton beams on normal tissues. Our aim is to evaluate normal tissue response after irradiation in mice which are able to develop side effects. For this purpose, C57Bl6 mice were total body irradiated by DS (Double Scattering) or PBS (Pencil Beam Scanning) at different proton doses and blood and organs were collected 3 months after irradiation. First results showed differences between both types of proton delivery in terms of survival but also for DNA damage, biomarkers of oxidative stress and inflammation. Experiments are still in progress and localized irradiation will be next performed.

### P 016 - Circular RNA CBL.11 Mediates the Proliferation of Colon Cancer Cells via Sponging up Mir-6778-5p and Regulating the YWHAE Expression

#### H. Li^1^, X. Jin^1^, Q. Li^1^

##### ^1^Institute of Modern Physics- Chinese Academy of Sciences, Department of Medical Physics, Lanzhou, China

Noncoding RNAs (ncRNAs) play important roles in several cancer cellular processes. Accumulating evidence indicates that ncRNAs are widely involved in various oncology treatments, providing potential targets for cancer intervention. Radiotherapy is one of the most effective therapeutic strategies for colon cancer patients. However, the expression profile and function of ncRNAs in radiotherapy for human colon cancer remain to be investigated. We performed a comprehensive study of microRNA (miRNA) in human colon cancer HCT116 cells using microarrays, and found that the expression of miR-6778-5p was downregulated in response to carbon ions. Bioinformatic and luciferase reporter analyses showed that YWHAE (14-3-3e), a member of the 14-3-3 protein family, was the direct target of miR-6778-5p, which mediated the function of miR-6778-5p in proliferation of colon cancer cells through inhibiting the activation of p38 MAP kinase. Furthermore, we found that circRNA CBL.11 was significantly up-regulated in HCT116 cells after irradiation, and functioned as an endogenous miR-6778-5p sponge to inhibit the miR-6778-5p activity. Silencing circRNA CBL.11 in HCT116 cells could down-regulate YWHAE, resulting in the inhibition of cell proliferation. In addition, the tumor inhibition effect of circRNA CBL.11 silencing was blocked by miR-6778-5p inhibitor. Taken together, we conclude that circRNA CBL.11 may function as a competing endogenous RNA to regulate the YWHAE expression through sponging up miR-6778-5p and exert regulatory functions for colon cancer. Thus, circRNA CBL.11 might be a potential target for colon cancer radiotherapy.

### P 017: Assessment of Out-Of-Field DNA Damage and the Impact of Neutrons on Secondary Cancer Risk in Proton Therapy

#### C. Vandevoorde^1^, P. Beukes^1^, E. de Kock^1^, S. Chiriotti^2^, A. Parisi^2^, M. De Saint-Hubert^2^, L. Tran^3^, E. Debrot^3^, A. Rosenfeld^3^, J. Slabbert^1^

##### ^1^NRF iThemba LABS, Medical Directorate, Cape Town, South Africa, ^2^Belgian Nuclear Research Centre SCK•CEN, Radiation Protection Dosimetry and Calibrations expert group RDC, Mol, Belgium, ^3^University of Wollongong Australia, Centre for Medical Radiation Physics, Wollongong, Australia

Despite the dose sparing properties of protons, they do have the potential to produce an unwanted dose outside the primary field. Secondary particles, most importantly neutrons, are inevitably produced, especially in older passive double-scattering proton therapy (DSPT) set-ups. While previous studies measured and simulated the dose deposited outside the primary proton field, no radiobiological evaluation has been reported so far. To date, large uncertainties remain on the existing RBE models and weighting factors for neutrons and if they are appropriate for secondary cancer risk estimation in PT. This study used an interdisciplinary approach to evaluate out-of-field DNA damage attributable to stray radiation in a DSPT.

Whole blood samples were irradiated at four fixed lateral and distal positions outside the primary proton field in addition to two positions in the primary field. Dosimetric measurements were performed at the same positions with neutron bubble detectors, Li6 and Li7 enriched TLDs and a silicon-on-insulator microdosimeter (MicroPlusTM). In addition, room mapping was performed with a TEPC at 11 locations around the isocenter. Irradiations were performed with a modulated clinical 200 MeV proton beam at iThemba LABS.

This study illustrates that there is an exponential decrease in absorbed dose out-of-field, while there is an increase in neutron contribution to the total dose as a function of the field edge, resulting in higher RBE values. The latter is particularly important for pediatric patients, since the reduction of secondary cancer risk is in fact one of the principal reasons for the shift from photon-based therapy towards PT.

### P 018: Study on the RBE Prediction for Carbon Scanning Irradiation Using a SOI Detector

#### K.B. Kim^1^, S. Han^2^, S.H. Yoo^3^, E.H. Kim^4^, A.B. Rozenfeld^5^, T. Kanai^6^

##### ^1^Korea Institute of Radiological & Medical Sciences, Radiation Oncology, Seoul, Korea Republic of, ^2^Korea Institute of Radiological & Medical Sciences, Division of Radiological & Medical Physics, Seoul, Korea Republic of, ^3^Hong Kong Sanatorium & Hospital, Medical Physics and Research Department, Happy Valley, Hong Kong, ^4^Korea Institute of Radiological & Medical Sciences, Division of Radiation Cancer Science, Seoul, Korea Republic of, ^5^University of Wollongong, Center for Medical Radiation Physics, nsw, Australia, ^6^Gunma University, Heavy Ion Medical Center, Maebashi, Japan

In this study, the prediction of RBE for carbon scanning beam using a SOI detector was investigated. On carbon ion radiotherapy, the relative biological effectiveness (RBE) related to beam quality makes a big difference from traditional radiotherapy and verification of RBE is important part on clinical commissioning. In our study, we report RBE prediction results for the scanning carbon beams compared to the broad beam case. SOI detector was used for measurement of lineal energy distribution (microdosimetric quantity) in carbon beam and modified MKM model was used for calculation of RBE. We show that the effects of noise are critical for the prediction of RBE and the difference of RBE between prediction value and TPS value is within 1.5% in our experiments. The SOI detector can be useful tools for dosimetric verification process of fast biological QA in scanning carbon beams.

### P 019: Effect of Mean Dose or Voxel-Wise Calculation in Prediction of Radiation-Induced Secondary Cancers

#### A. Madkhali^1,2^, D. Gasic^3^, C. Timlin^4^, T.T. Sio^5^, R.C. Miller^6^, M. Partridge^7^, P. Munck af Rosenschöld^8^, C. Hanquist Stokkevåg^9^

##### ^1^University of Oxford, Department of Oncology, Oxford, United Kingdom, ^2^King Saud University, Department of Medicine, Riyadh, Saudi Arabia, ^3^Rigshospitalet, Oncology - Section for Radiotherapy, Copenhagen, Denmark, ^4^University of Oxford, PTCRi, Oxford, United Kingdom, ^5^Mayo Clinic, Radiation Oncology, Phoenix- AZ, USA, ^6^Mayo Clinic, Radiation Oncology, Jacksonville- Florida, USA, ^7^St Ola, Orkney Islands, St Ola, United Kingdom, ^8^Skåne University Hospital, Radiation Physics, Lund, Sweden, ^9^Haukeland University Hospital, Department of Oncology and Medical Physics, Bergen, Norway

**Objective**: Increasing survival rates in cancer patients make it important to study late side-effects, including secondary radiation-induced cancers. Although a number of predictive models exist, the absolute accuracy of these models in the radiotherapy dose range is limited partly due to scarcity of long-term follow-up data. A challange faced when applying models to the highly spatially varying dose distributions produced in modern radiotherapy is dose heterogeneity. In this work we investigate the difference between using mean dose (MD) and high-resolution voxel-by-voxel dose (VbV) maps for calculating malignant induction probability (MIP).

**Material and Methods**: 12 3DCRT and 12 actively scanned proton plans for patients with medulloblastaoma were used. Treatment plans were exported to CERR and subsequently analysed in MATLAB and the MIP is calculated for each patient's plan using linear-quadratic (LQ), and linear (LIN) models with in-house developed code. The whole body MIPs calculated using the mean dose to the organs and VbV dose maps are compared

**Results**: Significant differences were observed between MIP by using MD and by using VbV maps for the 3DCRT plans (LQ:p < 0.001,LIN:p < 0.001) and proton plans (LQ:p < 0.001, LIN:p < 0.001) (Fig 1.a,1.b,2.a,2.b). MIPRelative was found to be significantly different between the two dose handling methods (LQ:p < 0.001, LIN:p < 0.001) (Fig 1.c,2.c).

**Conclusion**: The results demonstrate large systematic differences between the risk estimates produced using either MD or VbV. Although the relative relation between MIP3DCRT and MIPProton remains broadly consistent, using MD in heterogeneous dose distributions potentially overestimates MIP and, by association, secondary cancer risk.

### P 020: Interest of the EUD Concept for Dose Modelling Approach to Compare Proton vs Photon Treatment Plans

#### A. Chaikh^1^, J. Thariat^2,3^, T. Tessonnier^4^, J.M. Fontbonne^5^, P.Y. Bondiau^6^, J. Balosso^3,7^

##### ^1^LPC Caen, Applications médicales et induistrielle, Caen, France, ^2^UNICAEN, Radiation Oncology, Caen, France, ^3^Centre François Baclesse, Radiation Oncology, Caen, France, ^4^Centre François Baclesse, Medical Physics, Caen, France, ^5^CNRS/IN2P3, LPC Caen, Caen, France, ^6^Institut Méditerranéen de ProtonThérapie, Radiation Oncology, Nice, France, ^7^University Grenoble Alpes, Medicine, Grenoble, France

The use of normal tissue complication probability (NTCP) models to guide medical decisions is impeded by absolute uncertainties and predictive values rather limited to severe complications, beyond the scope of present day's radiotherapy. Here is tested in-silico the interest of a comprehensive estimation of the biological equivalent dose, EUD, for decision making instead of NTCP.

Fourteen paediatric cranio-spinal irradiations were evaluated. For each case, two treatment plans were generated using photon and proton with the same dose prescriptions. Three NTCP models were used for lung: LKB, EUD and mean lung dose (MLD) models. The lung radiation damages were considered as late effect. The α/β ratio was taken from QUANTEC report. To estimate NTCP values and generate dose-response curves, the cDVHs were converted into dDVHs. The physical dose of each DVH bin was converted into EQD2 using the LQ model. Wilcoxon paired test was used to calculate p-value.

A significant dose reduction was obtained with protons, with lower MLD and EUDs values. Lung NTCPs were significantly lower with protons, but the absolute NTCPs' and ΔNTCP values were to low to convince for technical shift, suggesting considering a readjustment for radiobiological parameter setting, especially to introduce the scope of lower grade (< 2) toxicities. The best NTCP prediction was found with MLD as input in NTCP model, which could be used thus as the dose tolerance limit for lung. The EUD reduction could confirm a true dose reduction and thus be used as indicator to estimate the potential benefit from protons.

### P 021: The development of a Biologically-Relevant Pre-Clinical Radiotherapy Dosimetry Phantom

#### E. Biglin^1^, A. Aitkenhead^2^, G. Price^3^, A. Chadwick^1^, K. Kirkby^1,4^

##### ^1^University of Manchester, Division of Molecular Clinical Cancer Studies- Faculty of Biology- Medicine and Health, Manchester, United Kingdom, ^2^The Christie NHS Foundation Trust, Christie Medical Physics and Engineering, Manchester, United Kingdom, ^3^University of Manchester- Manchester Academic Health Science Centre, The Christie NHS Foundation Trust, Manchester, United Kingdom, ^4^The Christie NHS Foundation Trust, The Christie, Manchester, United Kingdom

Preclinical radiotherapy studies using small animals are an indispensable step in the pathway from in vitro experiments to clinical implementation. As radiotherapy techniques advance in the clinic it is important preclinical models evolve to keep in line with these developments. So far this includes the use of orthotopic tumour sites, small animal image-guided radiotherapy platforms that mimic clinical treatment delivery and the development of tissue equivalent mice phantoms.

The path of radiation is affected by the density of the tissue that the radiation passes through, therefore affecting the delivered dose. Tissue-equivalent phantoms have been found to produce inconsistent results compared to euthanized mice and rats implying that current tissue equivalent and water phantoms are not suitable substitutes for heterogeneous mouse densities. In this study we utilise the capability of 3D printing to produce a phantom of varying density to mimic the heterogeneous tissue densities in a mouse to create an anthropomorphic phantom which reflect the size, physiological features, tissue and bone densities of a real mouse (Figure 1: 3D printed mouse phantom, based on a CBCT scan of a nude mouse.).

To further adapt this phantom for the purpose of replacing a real mouse it may be important to specifically design pockets to hold tumour or normal cell lines formed into 3D matrices for the assessment of biological dose. These matrices can be placed in their corresponding locations to represent targeted radiotherapy.

### P 023: Adjuvant Particle Radiotherapy for Orbital Tumor Patients Received Eye-Sparing Operation

#### W. Hu^1^, J. Lu^2^, L. Kong^1^, J. Hu^1^, J. Gao^1^, J. Yang^1^, X. Qiu^3^

##### ^1^Shanghai Proton and Heavy Ion Center, Radiation Oncology, Shanghai, China, ^2^Shanghai Proton and Heavy Ion Center, Radiation Oncology, Shanghai, USA, ^3^Shanghai Proton and Heavy Ion Center, Radiation Oncolgy, Shanghai, China

**Purpose**: We report the first clinical outcomes and toxicity data of adjuvant proton and/or carbon ion radiotherapy (CIRT) for orbital tumor after eye sparing surgery.

**Methods and Materials**: Between November 2015 and October 2017, 18 patients underwent adjuvant particle radiotherapy were analyzed. The LPFS, RPFS, DMFS and OS rates were calculated using Kaplan-Meier method. Acute and chronic toxicities were scored using the CTCAE 4.03.

**Results**: Fourteen patients received CIRT only (median dose: 63 GyE, range 60-69 GyE), 4 patients were treated with mixed proton and CIRT (3 patients with proton 56 GyE and CIRT 15 GyE; 1 patient with proton 22 GyE and CIRT 48 GyE). The median time between surgery and particle radiotherapy was 2.03 (range 0.57-6.13) months. The median follow-up time was 16.5 (range 3.83-28.37) months. At the last follow-up, 1 patient had local progression and died; 1 had regional recurrence, and 3 developed distant metastases. The 12-month LPFS, RPFS, DMFS and OS rates were 94.4%, 91.7%, 86.2% and 92.3%, respectively. Ten patients (55.6%) experienced acute grade 1 and 2 radiation side effect including 6 periorbital edema, 1 dry eyes, 1 epiphora, 1 conjunctival congestion and 1 decreased vision, no grade 3 and above acute toxicity was observed. For chronic toxicities, 1 patient developed grade 1 radiation-induced brain injury, 2 patients had grade 1 dry eyes, 1 developed grade 2 visual decrease, 1 had grade 3 visual decrease and 1 patient lost vision.

**Conclusion**: Adjuvant particle radiotherapy could provide satisfactory survivals and limited toxicities at 1 year for patients with orbit malignancies.

### P 024 - Proton versus Photon Radiation Therapy for Primary Gliomas: An Analysis of the National Cancer Data Base

#### J. Jhaveri^1^, E. Cheng^2^, M. Chowdhary^1^, S. Tian^1^, Y. Liu^2^, T. Gillespie^1^, B. Eaton^1^, H.K. Shu^1^, K. Patel^3^, M. McDonald^1^

##### ^1^Emory University, Radiation Oncology, Atlanta, USA, ^2^Emory University, Biostatistics and Informatics, Atlanta, USA, ^3^Yale University, Radiation Oncology, New Haven, USA

**Purpose**: To investigate the impact of proton radiotherapy (PBT) on overall survival (OS) and evaluate PBT usage trends for patients with gliomas in the National Cancer Data Base (NCDB).

**Methods**: Patients with a diagnosis of World Health Organization (WHO) Grade I-IV glioma treated with definitive radiation therapy (RT) between the years of 2004-13 were identified. Patients were stratified based on WHO Grade and photon radiotherapy (XRT) versus PBT. Univariate (UVA) and multivariable analysis (MVA) with OS were performed by Cox proportional hazards model and log-rank tests. Propensity score (PS) matching was utilized to account for differences in patient characteristics and to minimize selection bias.

**Results**: There were a total of 49,687 patients treated with XRT and 175 patients treated with PBT. Median follow-up time was 62.1 months. On MVA, the following factors were associated with receipt of PBT (all p < 0.05): WHO Grade I-II gliomas, treatment at an academic/research program (OR 3.90), west geographic facility location, and surgical resection. After PS matching, all patients treated with PBT were found to have superior median and 5-year survival than patients treated with XRT: 45.9 months vs. 31.9 (p = 0.0160) and 47.1% vs. 38.6% (p = 0.0160), respectively.

**Conclusions**: PBT is associated with improved OS compared to XRT for patients with gliomas. This finding warrants verification in the randomized trial setting in order to account for potential patient imbalances not adequately captured by the NCDB, such as tumor molecular characteristics and patient performance status.

### P 025: Harmonization of Treatment Planning Approach for Base of Skull Malignancies Using Pencil Beam Scanning: Second Report of the IPACS Collaboration

#### G. Mierzwińska^1^, C. Algranati^2^, A. Bäck^8,9^, T. Björk-Eriksson^3^, M. Cianchetti^2^, J. Góra^4^, M. Gustafsson^3^, P. Georg^5^, T. Kajdrowicz^1^, K. Kisielewicz^10^, J. Kubeš^5^, A. Perpar^6^, Z. Poulova^7^, K. Skowrońska^1^, M. Stock^4^, V. Vondráček^7^, P. Witt-Nyström^9,11^

##### ^1^Institute of Nuclear Physics Polish Academy of Sciences, Cyclotron Centre Bronowice, Kraków, Poland, ^2^Azienda Provinciale per i Servizi Sanitari, Proton Therapy Department, Trento, Italy, ^3^Sahlgrenska Academy at the University of Gothenburg, Institute of Clinical Sciences, Gothenburg, Sweden, ^4^EBG MedAustron GmbH, Medical Physics, Wiener Neustadt, Austria, ^5^Proton Therapy Center, Radiation Oncology, Prague, Czechia, ^6^EBG MedAustron GmbH, Radiation Oncology, Wiener Neustadt, Austria, ^7^Proton Therapy Center, Medical Physics, Prague, Czechia, ^8^Sahlgrenska Academy at the University of Gothenburg, Department of Therapeutic Radiation Physics, Gothenburg, Sweden, ^9^Skandion Clinic, Medical Physics, Uppsala, Sweden, ^10^Maria Sklodowska-Curie Memorial Cancer Center and Institute of Oncology, Radiation Oncology, Krakow, Poland, ^11^Danish Centre for Particle Therapy, Radiation Oncology, Aarhus, Denmark

The purpose of the work is to present and evaluate an inter-institutional proton treatment planning (TP) study developed by IPACS group (Italy, Poland, Austria, Czech Republic, Sweden), appointed in 2014. The study was based on five clinical cases of patients suffering from malignancies located in the base of skull region. The analysis and conclusions of the study aim to unify the delivery of proton radiotherapy and increase its scientific basis.

The study was based on anonymised medical data provided by PTC: five patients with inoperable or partially resected chordomas or chondrosarcomas, located in the proximity of brainstem, spinal cord, chiasm and optic nerves. Before the planning phase all centres established a common TP-protocol including planning technique, beam arrangement, calculation parameters, prioritization list for target volumes and organs at risk and plan evaluation. The protocol was developed based on experience gained from the first IPACS study (head and neck malignancies) and contained issues and definitions which are common for conventional radiotherapy (e.g. homogeneity and conformity indexes, near-minimum and near-maximum doses) and proton-specific ones (robustness analysis). CT scans with contoured targets and organs at risk along with dose prescriptions were sent to each centre for TP.

As a result, all centres prepared their treatment plans based on individual experience and according to boundary conditions deriving from the agreed TP-protocol. Plan parameters were then collected and are now being compared quantitatively between participants. This comparison will become a starting point for qualitative discussion. Detailed results and conclusions drawn will be presented at PTCOG 57.

### P 026: Quantitative Comparison between Different Strategies of Patient Positioning in Ocular Proton Therapy

#### A. Pella^1^, R. Via^2^, M. Ciocca^1^, M.R. Fiore^1^, B. Tagaste^1^, A. Giorgetto^1^, R. Ricotti^1^, F. Valvo^1^, G. Baroni^1,2^, R. Orecchia^3^

##### ^1^CNAO, Clinical Department, Pavia, Italy, ^2^Politecnico di Milano University, Dipartimento di Elettronica- Informazione e Bioingegneria, Milan, Italy, ^3^CNAO, Scientific Directorate, Pavia, Italy

At the Centro Nazionale di Adroterapia Oncologica (CNAO) in Pavia, Italy, ocular proton therapy (OPT) treatments started in September 2016, making the most of the available active scanning horizontal fixed beamline. Treatment geometry verification is performed through stereoscopic radiographic imaging for the localization of radiopaque tantalum clips previously sutured close to the intra-ocular lesion. Detected clip positions are compared to the corresponding reference configuration coming from the treatment planning system (Varian, Eclipse). A set-up correction vector is computed through a point-based rigid registration procedure (Verisuite PT, MedCom, Germany). Corrections are administered using a robotic chair with high mechanical accuracy (0.3 mm, 0.1°).

The aim of this preliminary study was to compare patient set-up accuracy achieved when 6 vs 3 degrees of freedom point based registration correction is applied.

At CNAO X-ray images are acquired prior and during irradiation for target-beam alignment and verification purposes, respectively. Five patients, and corresponding X-ray images acquired at the beginning of each treatment session were included in this study. Two point-based rigid registrations were compared: by excluding rotations and by including rotations. Residual clips 3D distances with respect to the nominal positions are reported in Table 1.

Median discrepancies lower than 0.4 mm were observed in the cohort of patients considered. The extension of the analysis to a larger population and a corresponding dosimetric evaluation of the effect of such geometrical deviations are required, in order to confirm that a 3 DOF set-up correction procedure may be sufficient to grant the required accuracy in OPT.

### P 027 - The evaluation of early tolerance in patients treated with proton radiotherapy (PRT)

#### T. Skóra^1^, K. Kisielewicz^2^, E. Pluta^3^, E. Góra^2^, A. Chrostowska^1^, R. Kopeć^4^, A. Patla^3^, D. Wojton-Dziewońska^1^, D. Kabat^2^, B. Sas-Korczyńska^1^

##### ^1^Maria Skłodowska-Curie Institute - Oncology Center, Department of Oncology, Kraków, Poland, ^2^Maria Skłodowska-Curie Institute - Oncology Center, Department of Medical Physics, Kraków, Poland, ^3^Maria Skłodowska-Curie Institute - Oncology Center, Department of Radiotherapy, Kraków, Poland, ^4^Institute of Nuclear Physics Polish Academy of Sciences, Bronowice Cyclotrone Centre, Kraków, Poland

The cooperation between the Maria Skłodowska-Curie Institute - Oncology Center in Kraków (medical side) and the Henryk Niewodniczański Institute of Nuclear Physics (Proteus-235 cyclotron owner) has been enabled PRT for patients since November 2016.

**Purpose**: To evaluate the early tolerance in patients completed PRT by the end of September 2017.

**Methods**: The study group comprised 59 patients with tumors of: skull base, vertebral, paranasal sinuses or low-grade brain gliomas. PRT was administered by pencil beam scanning and total dose was: 70 and 74 Gy(RBE) for chondrosarcomas and chordomas, respectively, 54 Gy(RBE) for brain gliomas and 70 Gy(RBE) for paranasal sinuses tumors.

Early toxicity was evaluated and scored according to CTCAE v. 4.0. Additionally, prognostic factors determining severity of toxicity (i.a. primary tumor site, SOBP value, PTV proximity to the skin, mucosa and air cavities) were assessed.

**Results**: We recorded 101 side effects (SEs) which intensity were as follows: G1 - 67 SEs, G2 - 26 SEs and G3 - 8 SEs. The most frequent SEs were dermatitis (64.4% patients) and oral/pharyngeal mucositis (39% patients). We observed the significant correlation between: (i) ≥G2 SEs and value of SOBP, (ii) frequency of dermatitis and tumor location at skull base or paranasal sinuses, and (iii) that SOBP value and air cavities proximity to PTV were strong predictors for G3 SEs.

**Conclusions**: Having regard to the short-term follow-up, presented results refer only to the early toxicity of PRT. Generally, PRT was well tolerated – only 8 SEs (8.1%) were in G3 and they were developed in 7 patients (11.9%).

### P 028: Utilization of Proton Beam Therapy among Adults with Primary Brain Tumors in the United States

#### W. Stross^1^, M. Waddle^1^, R. Miller^1^, A. Quinones-Hinojosa^2,^ A. Mahajan^3^, J. Peterson^4^, D. Trifiletti^4^

##### ^1^Mayo Clinic Jacksonville, Radiation Oncology, Jacksonville, USA, ^2^Mayo Clinic Jacksonville, Neurological Surgery, Jacksonville, USA, ^3^Mayo Clinic Rochester, Radiation Oncology, Rochester, USA, ^4^Mayo Clinic Jacksonville, Radiation Oncology- Neurological Surgery, Jacksonville, USA

**Purpose**: To investigate the utilization of proton beam radiation therapy (PBRT) in the treatment of adults with primary brain tumors (APBT) and characterize the patient population.

**Methods**: The National Cancer Database was queried for PBRT as the primary treatment modality of APBT between 2004 and 2015. International Classification of Diseases for Oncology codes for each patient were stratified into six histology categories; high-grade gliomas, medulloblastomas, ependymomas, other gliomas, other malignant, or other benign intracranial tumors. Demographics of the treatment population were also analyzed.

**Results**: A total of 1,296 patients received PBRT during the 11-year interval for treatment of their primary brain tumor. High-grade glioma, medulloblastoma, ependymoma, other glioma, other malignant, and other benign intracranial histologies made up 39%, 20%, 13%, 12%, 13%, and 2% of the cohort, respectively. The number of patients treated increased from 34 to 300 in years 2004 to 2015, encompassing 3% and 23%, respectively, of the entire cohort. Histologies varied over the 11-year interval with high-grade gliomas comprising 75% and 45% at years 2004 and 2015, respectively. The majority of the patient population were 18-29 years of age (59%), Caucasian race (73%), had median reported income of over $63,000 (46%), were privately insured (68%), and were treated at an academic institution (70%).

**Conclusions**: This is the first study to characterize the APBT patient population treated with PBRT. Our data from 2004-2015 illustrates a marked increase in the utilization of PBRT in the treatment of APBT and shows variability in the tumor histology treated over this time.

### P 029: Particle Therapy in Australia and New Zealand (ANZ): Beginning the Journey

#### V. Ahern^1^

##### ^1^Crown Princess Mary Cancer Centre, Radiation Oncology, Sydney, Australia

The Australian Federal and South Australian governments announced in mid-2017 a funding commitment for the first ANZ particle therapy facility, in Adelaide. Australia and New Zealand (ANZ) are first-world nations with dual government-funded public health care and private health systems. With a combined population of 29 million spread over a vast geographical area, the cost of providing proton therapy has been prohibitive. Estimating the number of ANZ patients who may benefit from proton therapy is challenging, varying from as few as 750 to as many as 8,500 annually.

Three additional consortia have business cases for particle therapy near-complete, one in each major cities in Australia (Sydney, Melbourne, Brisbane). One includes carbon ions in addition to protons. All propose a research room and links to universities.

The Royal ANZ College of Radiologists released a Position Statement on particle therapy in late 2015. An RANZCR Particle Therapy Special Interest Group has now been established bringing together radiation oncologists to ensure particle therapy is introduced in a coordinated and collaborative approach, to benefit patients. A National Particle Therapy Symposium was held in November 2017 and for the first time brought together radiation oncologists, medical physicists and radiation therapists with clinical research groups such as the Trans Tasman Radiation Oncology Group, senior university research leaders, policy makers and technology experts. The challenge now is to develop a roadmap for workforce development and credentialing, patient referral pathways, treatment protocols and to establish research programs which will define ANZ's unique contribution in the particle therapy field.

### P 030: Increased Distance from the Treating Proton Center Correlates with Shorter Follow-Up Time in Consented Registry (PPCR) Participants

#### M. Lawell^1^, B. Bajaj^1^, S. Gallotto^1^, M. Giblin^1^, J. Nartowicz^1^, C.B. Hess^1^, E. Weyman^1^, T.I. Yock^1^

##### ^1^Massachusetts General Hospital, Radiation Oncology, Boston, USA

**Objective**: Obtaining perdurable clinical follow-up (FU) is challenging and resource intensive for proton centers. Here we examined the effect distance between a patient's residency and their treating proton center has on maximum FU time.

**Methods**: Enrollment in the Pediatric Proton Consortium Registry (PPCR) was offered to any patient <22 years receiving protons at MGH. Of the PPCR patients, 143 (43.9%) were co-enrolled on a phase II proton protocol which has intensified methods for FU collection. Patients were excluded if they recurred or died. Distance from MGH was calculated by Great Circle formula, multivariate regression tested the relationship between duration of FU and distance.

**Results**: 326 PPCR patients enrolled between 8/2012 and 11/2016 were analyzed. The median FU is 2.2 years (<1-5.2), median distance away from the proton center is 164 miles (<1-10,532). Multivariate regression controlled for age, treatment delay, FU status, language, and co-enrollment and explained 16% of the variance (R2=.16, F=9.40, p<.0001). Distance from MGH significantly predicted follow-up time: patients living >164 miles from the proton center had average FU that was 0.29 years less compared to those living <164 miles (p=0.0326). Treatment delay was associated with a 0.71 year loss in average FU (p=0.0024). Co-enrollment significantly increased average FU by 0.45 years compared to PPCR enrollment alone (p=0.0012).

**Conclusion**: Patients living further from their treating proton center have shorter FU durations. Increased distance from treating centers may adversely affect clinical outcomes research. Enhanced sharing of medical information amongst providers is needed to effectively evaluate benefits of proton therapy.

### P 031: Proton Therapy in Childhood Pelvic Soft Tissue Sarcoma: Pattern of Failure and Toxicity

#### S. Nagaraja^1,2,3^, T. Steinmeier^2,3^, C. Plass^1,2,3^, S. Plaude^2,3^, S. Peters^1,2,3^, S. Tippelt^4^, D. Geismar^1,2,3^, B. Timmermann^1,2,3^

##### ^1^Clinic for Particle Therapy, University Hospital Essen, Essen, Germany, ^2^West German Cancer Center WTZ, University Hospital Essen, Essen, Germany, ^3^West German Proton Therapy Center Essen WPE, University Hospital Essen, Essen, Germany, ^4^University Hospital of Essen- Pediatrics III, Pediatric Hematology and Oncology, Essen, Germany

**Purpose**: To study the failure pattern and toxicity of proton therapy (PT) in children with pelvic soft tissue sarcomas (STS).

**Methods**: Data from 28 prospectively enrolled children in registry study KiProReg having Rhabdomyosarcoma (n=21) and other types of STS (n=7) treated with PT was analysed. Median follow-up time was 18.4 months (range: 6.3-47 months). Median age of children was 4.2 years (range: 1.5-16.1 years).

**Results**: At last follow up, 64.2% (n=18) of the patients were in complete remission, whereas 17.8% (n=5) and 10.7% (n=3) experienced local recurrence and stable disease, respectively and 7.2% (n=2) developed distant metastasis. Overall survival rate was 85.7% with local progression being the cause of death in 3 out of 4 patients. Initial tumour resection did not show significant benefit (p=0.61). Age less than 7 years (p=0.01) was associated with a poorer overall prognosis, whereas tumour size more than 5 cm (p=0.02) led to worse local control. At the end of PT, one patient with CTCAE °3 urinary toxicity and 4 patients with °3 skin toxicity were observed. Eight patients experienced °3-4 haematological toxicity due to the combined effect of chemotherapy. Early follow up showed good recovery. Eighty five percent of the children retained functioning bladder at last contact. The mean dose received by urinary bladder, rectum, hip joints and small intestines was 29.64Gy, 37Gy, 13Gy and 10Gy respectively.

**Conclusion**: Local control plays an important role in improving overall survival rate. Early data suggests that PT provides a feasible therapy option with a favourable toxicity profile.

### P 032: Prediction of Growth Patterns between Classical 3D Technique, Intensity-Modulated-Radiotherapy (IMRT) and Pencil Beam Scanning Protontherapy (PBS) in Pediatric Abdominal Tumors

#### D. Dumont Lecomte^1^, T. Tessonnier^2^, D. Stefan^1^, J.M. Fontbonne^3^, J.L. Habrand^1^, J. Thariat^4^

##### ^1^Centre François Baclesse, Radiotherapy, Caen, France, ^2^Centre François Baclesse, Physics, Caen, France, ^3^Laboratoire de Physique Corpusculaire, Physics, Caen, France, ^4^Centre Lacassagne, Radiation Oncology, Nice, France

**Introduction**: Irradiation of abdominal tumors can lead to serious spinal deformities, due to highly inhomogenous and assymetrical dose to the vertebrae next to the clinical target volume (CTV). With IMRT, doses to organs at risk (OAR) are reduced, compared to classical techniques, at the price of low dose spillage in this population at risk of second tumors. PBS can reduce the integral dose with similar benefits for the OAR. 3D irradiation, IMRT and PBS were compared in terms of dose homogeneity/symmetry to the vertebrae, and target dose coverage. Late toxicities and growth patterns for the 3 techniques were modelled and compared.

**Material and Methods**: 10 children irradiated with IMRT (Tomotherapy®), for nephroblastoma, neuroblastoma or sarcoma to a large dose range, were selected. In all cases, vertebrae were included in the CTV as recommended. The 3D technique consisted of an anteroposterior beam. IMRT and PBS (two posterior beams) were optimized to protect the kidneys and cord (previous toxic chemotherapy received). Similar dose constraints were applied on the target volume.

**Results**: Preliminary data in a 2yo boy with neuroblastoma showed better craniocaudal dose gradient for vertebrae adjacent to CTV, kidney sparing and much broader low/intermediate dose bath with PBS than IMRT (Figure 1: Dose distribution in PBS, IMRT and 3D irradiation plans). Dose homogeneity in the vertebrae was superior with PBS even when stringent constraints are applied on other OAR. Results of 9 other patients are forthcoming.

**Conclusion**: Proton therapy seems promising for an harmonious growth while ensuring proper dose coverage and sparing OAR.

### P 035: Risks of Cancer Recurrence and Radiation-Induced Late Toxicity after Photon versus Proton Therapy for Locally Advanced Breast Cancer

#### L.B. Stick^1^, J. Yu^2^, M.V. Maraldo^1^, M.C. Aznar^3^, A.N. Pedersen^1^, S.M. Bentzen^4^, I.R. Vogelius^1^

##### ^1^Rigshospitalet, Department of Oncology, Copenhagen, Denmark, ^2^Miami Cancer Institute, Department of Radiation Oncology, Miami, USA, ^3^The Christie, Department of Oncology, Manchester, United Kingdom, ^4^University of Maryland, Greenebaum Comprehensive Cancer Center and Department of Epidemiology and Public Health, Baltimore, USA

**Purpose**: Comprehensive multi-endpoint risk assessment of competing photon and proton therapy plans. Estimate risk of recurrence caused by compromising dose to target and risks of radiation-induced toxicity of coronary heart disease (CHD), secondary lung cancer (SLC) and secondary cancer breast (SBC).

**Methods and Materials**: Forty-one consecutive left-sided breast cancer patients were treated with postlumpectomy locoregional radiotherapy at a single institution during 2015. Thirty-nine patients were treated with 3D-CRT. Two patients were treated with VMAT. Competing spot-scanning proton plans using two en face beams and single-field optimization were created. A model for risk of recurrence was derived based on hazard ratios from published randomized trials. Radiation-induced toxicity models were developed from published dose-response data for CHD, SLC and SBC after fractionated radiotherapy.

**Results**: The median excess absolute risk (EAR) of recurrence or death after 10 years was 0.4% (range, 0.2%-2.0%) with photon therapy and 0.15% (range, 0.11%-0.2%) with proton therapy. Using photons the median EAR of CHD by age 80 without/with cardiac risk factors was 0.5% (range, 0.03%-1.0%)/1.0% (range, 0.2%-2.9%) and using protons 0.06% (range, 0.005%-0.3%)/0.13% (range, 0.02%-0.5%). The median EAR of SLC by age 80 for non-smokers/smokers was 1.3% (range, 0.06%-1.7%)/2.7% (range, 1.2%-3.5%) with photons and 0.7% (range, 0.04%-1.1%)/1.4% (range, 0.8%-2.1 %) with protons. The median EAR of SBC by age 80 was 0.6% (range, 0.01%-3.6%) using photons and 0.04% (range, 0.0%-0.4%) using protons.

**Conclusion**: Risks of recurrence and severe toxicity were modeled with consideration of patient-level risk factors and should be considered when identifying patients for proton therapy.

### P 037 - Robustness and Organ Sparing Potential of Intensity Modulated Proton Therapy for Esophageal Cancer

#### R.M. Anakotta^1^, H.P. van der Laan^1^, E.W. Korevaar^1^, M. Dieters^1^, R. Wijsman^1^, J.A. Langendijk^1^, A.C. Knopf^1^, C.T. Muijs^1^

##### ^1^University Medical Center Groningen, Radiation Oncology, Groningen, Netherlands

**Background and Purpose**: To test the robustness of intensity modulated proton therapy (IMPT) in esophageal cancer patients using repeat 4D-CT-scans. Robustness and organ sparing capabilities of IMPT were compared with those obtained with volumetric modulated arc therapy (VMAT).

**Method**: Eleven esophageal cancer patients underwent a 4D planning-CT (pCT0) and 5 weekly repeat 4D-CT-scans. VMAT and ITV-based robustly planned IMPT plans were optimized and then evaluated on each weekly repeat-CT considering 2 mm setup and 3% range error scenarios. Accumulated dose distributions were deformed and then summed on the reference pCT0.

**Results**: The summed doses from the weekly repeat-CTs on pCT0 resulted in adequate ITV coverage for all 11 VMAT plans and for 9 out of 11 IMPT plans (Table). In 2 patients, IMPT treatment plan adaptation, using a different beam direction to avoid air in the stomach, was required for adequate coverage. On average the D98 was 43.0 Gy (VMAT) and 42.2 Gy (IMPT). The average mean heart dose with VMAT was 20.1 Gy (SD: 3.2; range: 14.9–26.7) and with IMPT 9.2 Gy (SD: 3.2; range: 5.3–15.6). The average mean lung dose with VMAT was 7.8 Gy (SD: 2.3; range: 4.0–10.6) and with IMPT 3.7 Gy (SD: 1.1; range: 1.1–4.9). The spinal cord tolerance dose was never exceeded.

**Conclusions**: IMPT for esophageal cancer, with optional weekly plan adaptation, resulted in adequate target coverage similar to that with VMAT when interfractional variation and various error scenarios were considered. Compared to VMAT, IMPT resulted in clinically relevant dose reductions to both the heart and lungs.

### P 038: Simulation Study of Proton Beam Therapy Using Concomitant Boost Technique for Unresectable Pancreatic Cancers

#### N. Fukumitsu^1^, T. Okumura^1^, Y. Hiroshima^1^, H. Numajiri^1^, K. Murofushi Nemoto^1^, K. Ohnishi^1^, T. Aihara^1^, H. Ishikawa^1^, K. Tsuboi^1^, H. Sakurai^1^

##### ^1^University of Tsukuba, Radiation oncology, Tsukuba, Japan

**Objectives**: The purpose of this study is to investigate the dose distribution of proton beam therapy (PBT) using a concomitant boost technique for unresectable pancreatic cancers.

**Methods**: This simulation study involved 36 patients with unresectable pancreatic cancer. The irradiation dose was set as 67.5 gray equivalent (GyE) with 25 fractions using concomitant boost technique. The irradiation dose was set as 50 GyE to cover the whole target and another posterior beam of 17.5 GyE was added to ensure that 10% isodose line was not delivered to the gastrointestinal (GI) tract. Dose distribution of the gross tumor volume (GTV) and GI tract was examined.

**Results**: V55GyE, 60GyE, 65GyE were 80.8, 66.5, and 42.4%, respectively, and mean dose was 64.1 GyE in all patiets. The distance from the GI tract showed significant difference in dose distribution (P=0.002 in V55GyE, 0.0009 in V60GyE, 0.003 in V65GyE, and 0.02 in mean dose, respectively). Location, tumor diameter, or lymph nodes metastasis did not show any difference.

**Conclusions**: We found that irradiated dose is closely related to the distance from the GI tract. Clinically, this protocol is expected to have outstanding effects on local control of tumors compared to conventional PBT.

### P 039: Clinical Results of Proton Therapy for Primary Liver Cancer in 272 Chinese Patients

#### S. Huang^1^, W. Pan^1^, C. Sun^1^, Q. Lyu^1^, W. Zhu^1^, H. Yang^1^, J. Zhang^1^

##### ^1^Zibo Wanjie Cancer Hospital, radiation oncology, Zibo, China

**Purpose**: To evaluate the clinical outcomes and toxicities of proton therapy for primary liver cancer.

**Patients and Methods**: From December 2004 to August 2016, 272 patients with primary liver cancer were treated with proton therapy at Zibo Wanjie Proton Therapy Center. There were 238 men and 34 women. 176 patients were diagnosed by pathologic conformation, 96 patients were diagnosed on the basis of guidelines on the diagnosis and treatment of primary liver cancer (2011 edition). The patients of hepatocellular carcinoma and cholangiocarcinoma were 246 and 26, respectively. There Child-Pugh category for impairment of liver function was Class A in 145 patients, Class B in 124 patients, and Class C in 3 patients. The prescription dose were 50-66CGE/10-30F.

**Results**: The 5-year LC were 80% for all patients, and 90% for Stage I and II patients. The 5-year OS of Stage I, Stage II, Stage IIIa , Stage IIIb , Stage IVwere 75%, 46.6%, 18.4%, 3.5%, 13% and 0%, respectively. Acute toxicities were transient, upper gastrointestinal toxicities were observed in 61 patients, including 38 with grade 1 and 23 with grade 2; skin toxicities were observed in 149 patients, including 139 with grade 1 and 10 with grade 2. Late toxicities included skin toxicities, liver toxicities, bowel toxicities, rib toxicities.

**Conclusions**: Proton therapy is an effective and safe method for the patients with primary liver cancer.

### P 041: Is It Too Small? The Treatment Outcome of Proton Therapy-Treated Hepatocellular Carcinoma Patient with Small Normal Liver Volume

#### C.H. Lee^1^, B.S. Huang^2^

##### ^1^Chang-Gung Memorial Hospital Linkou medical center, Radiation Oncology, Taoyuan City, Taiwan\r, ^2^Chang Gung Memorial Hospital Linkou medical center, Radiation Oncology, Taoyuan City, Taiwan\r

**Purpose**: Evaluating the result of hepatocellular carcinoma (HCC) patients with small normal liver volume treated by proton beam therapy (PBT).

**Materials and Methods**: HCC Patients with normal liver volume (NLV) less than 800 cm3 without distant metastasis treated in our proton center were included. The doses of proton beam therapy were mainly 72.6GyE in 22fractions and 66GyE in 10 fractions according to tumor location.

**Results**: Twenty-five patients were enrolled between 2015 November and 2016 December. The one-year progression free survival rate was 42.9% (Fig.1) and one-year overall survival rate was 82%. The one-year in-field failure free rate was 96%. Normal liver volume (NLV) was ranged from 483.9 cm3 to 795.8 cm3 (median, 642.9 cm3). Non-irradiated liver volume (NILV) was ranged from 232.9 to 531.6 cm3 (Median, 390.1 cm3). The NLV received less than 30GyE (rV30) was ranged from 319.1 cm3 ∼ 633.3 cm3 (median, 492.3 cm3). None of our patients developed liver failure. One patient with initially abnormal liver enzyme developed non-classic radiation-induced liver disease.

**Conclusion**: Our result revealed high infield failure free survival and low liver toxicity. It is feasible for patients with NILV larger than 500 cm3 to receive proton therapy, but the lower limit of NILV may need a prospective research with larger patient number to determine.

### P 044: Proton Boost for Prostate Cancer Utilizing MR Imaging

#### W. Barrett^1^

##### ^1^University of Cincinnati, Department of Radiation Oncology, Cincinnati, USA

Conventional photon and proton treatment for prostate cancer most often targets the entire prostate for the entirety of the treatment course. This has been reasonable given the common multifocality of cancer within the prostate gland and difficulty associated with identifying and targeting dominant lesions within the gland.

This protocol utilizes photon IMRT to treat the entire prostate and seminal vesicles to adequate dose (5000 cGy in 25 tx's) to have high likelihood of eradicating very small volume disease followed by a proton boost of 3000 cGy in 15 tx's to dominant lesions identified by pretreatment MR. The expectation is to reduce toxicity by decreasing dose to portions of bladder, rectum and urethra that would otherwise be included in whole prostate treatment prescribed to the total dose. Disease control may also be improved by escalating the total dose from 7600 cGy (our institutional standard) to 8000 cGy to dominant lesions. The recruited cohort (50 patients) will be compared for toxicity and efficacy to conventional IMRT photon treated patients matched by age, Gleason score, PSA, clinical stage, IPSS, potency and bowel function. Patient accrual is anticipated to begin in Spring 2018.

### P 045: Initial Report of Rectum and Bladder Dose Constraints for Treatment Planning of Carbon Ion Radiotherapy of Locally Advanced Prostate Cancer

#### L. Hong^1^, Z. Chen^1^, Y. Xing^1^, P. Li^2^, Q. Zhang^2^

##### ^1^Shanghai Proton and Heavy Ion Center, Department of Medical Physics, Shanghai, China, ^2^Shanghai Proton and Heavy Ion Center, Department of Radiation Oncology, Shanghai, China

**Purpose**: To report the acute toxicities associated with hypofractionated carbon ion radiotherapy (CIRT) for patients with local advanced prostate cancer (LAPC) and recommend dose constraints for routine clinical treatment planning.

**Materials and Methods**: 26 patients with LAPC received CIRT using two lateral portals with a total dose of 59.2 Gy (RBE) in 16 daily fractions. All contouring and treatment planning were done by experienced physicians and physicists in Syngo PT Planning VC13 (Siemens). Acute toxicity was scored according to the CTCAE version 4.03. Each plan was quantitatively assessed using dose-volume histograms (DVHs) to assure compliance with the prescribed dose constraints. CTV coverage was also evaluated with the homogeneity index (HI) and inhomogeneity coefficient (IC).

**Results**: The median patient age was 68.5 years (range 50-85). All patients completed the planned treatment. No patient developed grade ≥ 1 rectal or ≥ 3 urinary toxicity; four patients developed grade 2 and three developed grade 1 urinary toxicity. All plans maintained adequate coverage of CTV, with 100% volume receiving ≥95% of the prescribed dose, the mean HI and IC (in %) were 2.67±0.91 and 0.09±0.02. DVHs and dose statistics of bladder and rectum are depicted as in Figure 1 and Table 1.

**Conclusion**: The average DVHs of rectum and bladder can be referred as dose constraints since low rates of acute toxicity were observed. Our results demonstrated the clinical feasibility of CIRT for LAPC and support further investigation to evaluate late toxicities and therapeutic efficacy.

### P 046: A Planning Feasibility Study of Intensity-Modulated Proton Therapy for Re-Irradiation of Large Pelvic In-Field Recurrences

#### G. Eminowicz^1,2^, V. Rompokos^1^, R. Amos^3^, L. Beaton^2^, H. Payne^1,2^, A. Mitra^1^, R. Davda^1,2^, T. Richards^1,2^, B. Seddon^1^, R. Sharma^1,2^

##### ^1^University College London Hospital, Radiotherapy department, London, United Kingdom, ^2^University College London, Cancer institute, London, United Kingdom, ^3^University College London, Department of Medical Physics and Bioengineering, London, United Kingdom

**Background**: Loco-regional failure remains a challenge in the previously irradiated pelvis. Stereotactic body radiotherapy (SBRT) enables high tumour dose delivery with proven safety of 27-45Gy in 3-6 fractions for pelvic re-irradiation. However, for large pelvic relapses not typically suitable for SBRT, intensity-modulated proton therapy (IMPT) may offer superior dosimetry for SBRT delivery. We performed a feasibility study of IMPT-based SBRT for large pelvic relapses, benchmarked against RapidArc (VMAT).

**Method**: Six patients with pelvic nodal/soft tissue recurrences from rectal, endometrial, cervical, urological cancers, or pelvic sarcomas were included. Conservative assumptions were made to estimate OAR recovery based on tissue type and time from previous radiation (range: 20-30%). Highest priority was to not exceed the integral OAR EQD2. Geometric uncertainties were calculated from evaluation of treatment CBCTs. PTV coverage and robustness analysis were assessed for photons and protons respectively to ensure adequate target coverage to deliver 30Gy in 5 fractions.

**Results**: From 3 cases analysed so far, IMPT plans produced acceptable and comparable target coverage to VMAT with superior normal tissue sparing for targets with sufficient separation from OARs. For targets adjacent to OARs, both modalities were equivalent, assuming a uniform proton RBE of 1.1. Importantly, IMPT plans appeared superior at ensuring improved target dose coverage when multiple adjacent OARs were in close proximity (see Table).

**Conclusions**: Clinical application of IMPT is feasible for targets with sufficient separation from OARs. When targets are adjacent to OARs, an individualised approach is essential. Further investigation of this clinical indication is warranted; a formal clinical trial is in development.

### P 047: Late Morbidity after Proton or Photon Boost in Locally Advanced Prostate Cancer

#### E. Khmelevsky^1^, I. Kancheli^2^, N. Fedorenko^1^, A. Kaprin^1^

##### ^1^P.A. Herzen Moscow Scientific and Research Oncological Institute- Health Ministry of the Russian Federation, Radiation oncology, Moscow, Russian Federation, ^2^FSBI “Alikhanov Institute for Theoretical and Experimental Physics” SRC “Kurchatov Institute”, Medical physics, Moscow, Russian Federation

**Purpose**: This study investigated late post irradiation damages and second tumors after proton-photon or photon therapy for locally advanced prostate cancer.

**Materials and Methods**: Results of a randomized study at several fractionation regimes were analyzed in 289 patients with high and intermediate progression risk. Three variants of proton boost were studied: 3.0 (8 daily fractions), 4.0 (5 fractions, 3 or 5 fractions/week), and 5.5 (3 fractions, 3 fractions/week) Gy(RBE).

**Results**: 10 years results are presented in Table.

After protons a significant decrease in the severity of acute and late GI injuries is seen. Late GI and GU toxicity decreased by 30% and 15%. 23 patients (13 vs 10) had metachronous tumors (median 52 mo), and GI tumors prevailed: 4 gastric cancer cases, 3 cases of colon and pancreas cancers each, 2 cases of rectal cancer and 1 case of rectal PNET. Besides, 1-2 cases of lung and larynx cancers, glioblastoma, malignant pleural mesothelioma, HLL, were diagnosed. In the irradiation zone 3 tumors emerged (the proton group, median 50 mo), outside the zone - 20 tumors (10 vs 10, median 60 mo).

**Conclusion**: Proton boost significantly reduces early and late rectitis, but does not influence lower urinary tract injuries. No significant difference in toxicity levels seen between fractionation regimes. 10 years actuarial risk of second malignant tumor after irradiation was 22% and did not differ reliably in both groups. The unreliable increase after proton boost of second tumors rate formed in the zone of the exposure was 2.6% vs 0% (p>0.05).

### P 048: Health Disparities and Inequities in the Utilization of Proton Therapy for Prostate Cancer

#### R. Katipally^1^, C. Deville, Jr^2^

##### ^1^The Warren Alpert Medical School of Brown University, Department of Radiation Oncology, Providence, USA, ^2^The Johns Hopkins Hospital, Department of Radiation Oncology and Molecular Radiation Sciences, Baltimore, USA

**Purpose**: To review and summarize reported health disparities and inequities in the use of proton beam therapy (PBT) for prostate cancer (PCa) in the United States

**Methods**: A comprehensive literature search in the PubMed database with the search query “prostate AND proton AND (disparities OR IMRT OR race OR insurance OR socioeconomic OR inequities)”. Studies were included if they examined PBT in PCa and addressed health inequities.

**Results**: The initial search query returned 91 studies, of which 11 met inclusion criteria. The characteristics of studies, types of health inequities investigated, and outcomes examined are presented in Table 1. Generally, patients with increased age, non-white race, and poorer socioeconomic status (most commonly measured by income) were less likely to receive PBT. For instance, one National Cancer Database (NCDB) study of 187,730 patients with localized PCa from 2004 to 2012 demonstrated that black, Hispanic, and other minority patients were less likely to receive PBT with adjusted ORs of 0.20, 0.57, and 0.59, respectively. However, there is no evidence that race contributes to differences in toxicity after PBT. One single institution, matched-pair analysis of health-related quality of life two years after PBT demonstrated no statistically significant differences in sexual function, urinary incontinence, urinary obstruction, or bowel summary scores between black and white patients. Private insurance and academic practice settings were also associated with increased odds of receiving proton beam therapy.

**Conclusions**: Significant disparities in PBT utilization for PCa exist, most commonly pertaining to age, race, socioeconomic status, insurance status, and practice characteristics.

### P 049: Carbon Ion Radiotherapy Alone for Favorable Intermediate Risk Group Prostate Cancer

#### N. Kubo^1^, H. Kawamura^1^, H. Sato^1^, M. Iwanaga^1^, A. Adachi^1^, T. Ohno^1^, H. Matsui^2^, K. Ito^2^, K. Suzuki^2^, T. Nakano^1^

##### ^1^Gunma university, Heavy Ion Medical Center, Maebashi, Japan, ^2^Gunma University Graduate School of Medicine, Department of Urology, Maebashi, Japan

**Background**: The aim of this study was to evaluate carbon-ion radiotherapy (CIRT) without hormonal therapy for the favorable intermediate risk group prostate cancer.

**Methods**: A subgroup analysis of 50 patients with favorable intermediate risk group prostate cancer were performed among 304 patients enrolled in our prospective trial (GUNMA 0702) on CIRT for prostate cancer. Patients were classified as having favorable intermediate risk disease with clinical stages T1c to T2b, a Gleason score of 3+4, and a pretreatment PSA less than 10 ng/ml. All patients were treated with CIRT (57.6 Gy RBE in 16 fractions over 4 weeks) to the prostate and proximal seminal vesicles between March 2010 and August 2013. These patients were treated without hormonal therapy. Toxicity data were scored according to the Common Terminology Criteria for Adverse Events Version 4.0.

**Results**: The median age was 60 years (range 41-84). The median follow-up period was 60.3 months. The 5-year PSA relapse free rate was 97.6%. Two deaths occurred from other causes. There was no patient experienced with grade 3 or worse genitourinary toxicity. Late grade 2 genitourinary toxicity was seen in 6 cases (12%). There was no patient experienced with grade 2 or worse gastrointestinal toxicity.

**Conclusion**: CIRT without hormonal therapy for favorable intermediate risk group prostate cancer showed the feasible outcome and safety. This data suggests that it is a rational strategy to treat these patients with CIRT alone.

### P 050: The Role of 99mTc-Labeled PSMA-SPECT/CT and Multi-Parametric MRI in the Prediction of Early Response after Particle Therapy for Prostate Cancer

#### P. Li^1^, S. Wu^1^, C. liu^2^, D. Lin^3^, Z. Guang yuan^3^, Z. Qing^1^, F. Shen^1^

##### ^1^Shanghai Proton and Heavy Ion Center, Department of Radiation Oncology, Shanghai, China, ^2^Shanghai Proton and Heavy Ion Center, Department of Nuclear Medicine, Shanghai, China, ^3^Shanghai Proton and Heavy Ion Center, Department of radiology, Shanghai, China

**Purpose**: This purpose of this study was to assess the predictive value of 99mTc-labeled PSMA-SPECT/CT and multi-parametric MRI before and after particle therapy in prostate cancer.

**Methods and Materials**: A total of 101 pathological confirmed prostate cancer patients were treated with carbon ion and/or proton at our institution between June 2014 and September 2017. Of them, 23 patients with pathological confirmed prostate cancer underwent 99mTc-labeled PSMA-SPECT/CT and multi-parametric MRI before and after particle therapy. The mean apparent diffusion coefficient (ADCmean) and tumor/background ratio (TBR) were measured on the tumor and the percentage changes between 2 time points were calculated. And the PSA and Gleason score were also assessed. We divided patients into two groups: PSA response (PSA level < 0.2ng/ml after 6mon treatment) and non PSA response (PSA level ≥0.2ng/ml after 6mon treatment).

**Results**: The median follow up time is 16 months. After particle therapy, there are 4 patients identified as non PSA response (17.4%). The ADCmean was significantly increased as compared with the pretreatment value(0.79×10-3mm2/s vs. 1.18×10-3mm2/s ), while the TBR was significantly decreased compared with the pretreatment value(14.82 vs.5.81). PSA response group showed a significantly greater reduction in TBR after particle therapy than non PSA response. And the ADCmean changes in PSA response group was signifcantly higher than that in non-PSA response group.

**Conclusions**: Our preliminary data indicate that the changes of ADCmean and TBR during particle therapy may be an early bio-marker for predicting prognosis after particle therapy in prostate cancers.

### P 051: Offline PET Signal: An Underestimated Predictor for Acute Adverse Effects on CIRT Prostate Cancer Patients?

#### Y. Zhang^1,2^, Y. Gong^2,3^, J. Meng^4^, P. Li^1^, Q. Yu^1,2^, S. Wu^1,2^, X. Chen^1,2^, S. Fu^1,2,5^

##### ^1^Shanghai Proton and Heavy Ion Center-Fudan University Cancer Hospital, Department of Radiation Oncology, Shanghai, China, ^2^Shanghai Medical College- Fudan University, Department of Oncology, Shangha, China, ^3^Shanghai Proton and Heavy Ion Center-Fudan University Cancer Hospital, Department of Medical Physics, Shanghai, China, ^4^Fudan University Shanghai Cancer Center, Department of Radiation Oncology, Shanghai, China, ^5^Fudan University, Key Laboratory of Nuclear Physics and Ion-beam Application MOE, Shanghai, China

**Background**: Carbon ion radiotherapy(CIRT) is highly normal-tissue protective, however, GU toxicities still exist, probably caused by off-target effect due to the scattering of the beam and organ positions. Offline PET can detect the β+ isotopes produced by atomic reactions, to some extent, relevant to doses. But the relation between PET signal and GU toxicities remains elusive. Our hypothesis was that in-vivo PET signal of organ at risk (OAR) may be correlate to acute adverse effects.

**Materials and Methods**: We retrospectively analyzed offline PET/CT, treatment plans and GU toxicities in 19 localized prostate cancer patients who received CIRT between 06/2014-09/2014. Every patient had 3 PET CT-Scan. The inclusion criterion is total PET signal>5E+04 Bq/ml considering less time-related washout. Finally, 49 PET/CT were included. The bladder was manually segmented on plan CT and PET/CT. We calculated the ratio of the sum to PET signals in bladder and total PET image. As bladder is a volume-relating OAR, volume-division was implemented and we had bladder relative PET signal density (RPSD). DVH-based metrics were extracted for every patient. The RPSD and DVH parameters were compared between patients with/without acute GU toxicities using two-sample t test.

**Result**: Patients with GU toxicities had higher RPSD than those without (Figure 1,2), indicating its potential role as GU toxicities predictor. No significance difference was found in DVH metrics between patients with/without acute GU toxicities (p=0.18312∼0.94318), though the smallest p was found in V52GyE.

**Conclusion**: RPSD may correlate to acute GU adverse effect and be a potential predictor in prostate cancer CIRT.

### P 052: Proton Beam Radiotherapy for Pediatric Soft Tissue Sarcomas: A Single-Institutional Experience on Acute Toxicity and Short-Term Outcomes

#### J.Y. Shin^1^, W. Hartsell^2^, J.H.C. Chang^3^

##### ^1^Rush University Medical Center, Radiation Oncology, Chicago, USA, ^2^Chicago Proton Center, Proton Therapy, Warrenville, USA, ^3^Vanderbilt University, Proton Therapy, Nashville, USA

**Introduction**: The objective of our study is to assess the short-term efficacy and acute toxicity of proton beam radiotherapy (PBT) as part of definitive management in pediatric patients diagnosed with soft tissue sarcomas.

**Methods**: We identified 40 pediatric patients treated between 2011 and 2016 with soft tissue sarcoma. The endpoints analyzed were treatment-related acute toxicity, progression-free survival (PFS), and overall survival (OS).

**Results**: Rhabdomyosarcoma was the most common histology (52.5%) followed by Ewing's sarcoma (30.0%). Most patients (67.5%) underwent primary surgery, and 95% of patients received chemotherapy. The most common proton radiation technique used was three-dimensional uniform scanning (82.5%) while the remaining patients received pencil beam scanning. The median proton radiation prescription was 5040 Cobalt Gray Equivalents (CGyE) in 28 CGyE fractions.The most commonly reported acute toxicity was grade 2 dermatitis in 23 patients (57.5%). Four patients (10.0%) had grade 3 dermatitis. One patient (2.5%) experienced grade 3 nausea. Other grade 2 acute toxicities included pain in 11 patients (27.5%), weight loss in 5 (12.5%), fatigue in 4 (10.0%) and nausea in 3 patients (7.5%).Twenty-nine patients (72.5%) were alive without local failure or distant disease at last follow-up, corresponding to a 2-year OS of 94.4%. The 1- and 2-year local control rates were 91.6% and 81.4%. The 1- and 2-year PFS rates were 88.9% and 67.5%, respectively.

**Conclusions**: PBT for pediatric sarcomas appears to be effective and was delivered with acceptable acute treatment-related toxicity in the setting of definitive or adjuvant irradiation. Further long-term follow-up study is warranted.

### P 053: Definitive Radiation Treatment for Osteosarcoma in the Spine and Pelvis

#### H. Wang^1^, R. Miao^1^, J. Schwab^1^, G. Cote^1^, E. Choy^1^, Y.L. Chen^1^

##### ^1^Massachussets General Hospital, Radiation Oncology, Boston, USA

**Background**: osteosarcoma in the spine and pelvis are rare and challenging tumors to treat especially with surgery causing significant comorbidities. We report a cohort of patients who received definitive radiation therapy without surgery as local treatment.

**Methods**: patients with osteosarcoma in the spine and pelvis were identified from our database. We retrospectively reviewed of the clinical presentation, treatment, outcome, and patterns of failure. Kaplan-Meier analysis was used to analyze survival outcomes.

**Results**: Seven patients were identified who met the criteria for having local disease and receiving definitive radiation (more than 60Gy in total). The median age was 46. Four patients had the primary tumor in the sacrum, two in the pelvis and one in the lumbar spine. Four patients had tumor size more than 8cm and 3 had tumor size less than 8cm. Five patients had high grade tumor and 2 patients had Grade 2 tumor. All of these patients received radiation dose more than or equal to 60 Gy. Four patients received more than 70Gy. Five patients had proton therapy. The median follow up time is 83 months. Four patients were still alive by the end of the follow up without evidence of disease. The 3 and 5 year overall survival is 86% and 69% respectively. The local control rate was 57% both at 3 years, at 5 years, and at 10 years.

**Conclusion**: Definitive radiation treatment for osteosarcoma in the spine and pelvis shows comparable survival results compared to other studies with surgery as part of the local treatment.

### P 054: Dosimetric Comparison of Intensity-Modulated Proton Therapy (IMPT) and Volumetric-Modulated Arc Therapy (VMAT) Treatment Plans for Sacral Chordoma

#### F. Le Grange^1^, P. Lim^1^, V. Rompokos^2^, A. Gosling^2^, C. Gillies^2^, M. Ahmed^1^, B. Seddon^1^

##### ^1^University College London Hospitals, Clinical Oncology, London, United Kingdom, ^2^University College London Hospitals, Radiotherapy Physics, London, United Kingdom

**Purpose**: A dosimetric comparison of IMPT and VMAT for locally advanced sacral chordoma.

**Method**: Ten radiotherapy sacral chordoma patients were selected. The dose prescribed was 70Gy with integrated boost to the GTV of 74.2Gy over 30 fractions for both cases (PTV/CTV for VMAT/IMPT respectively - RBE for protons). Planning objectives were to limit dose to the cauda equina, the lumbosacral plexus and surrounding organ structures. IMPT and VMAT plans were produced for each case. Robustness analysis was performed for IMPT to ensure target coverage and normal structure sparing by assessing the worst case scenario. The assessment for photon plans was performed based on geometrically expanded volumes (e.g. CTV to PTV).

**Results**: The dose to rectum and bowel proximal to the target was similar between the two modalities and independent of bowel movement as assessed on treatment CBCT; however IMPT achieved lower mean dose. IMPT achieved lower doses to all structures anterior to the target (bladder, femoral heads, etc.). The tolerance of the sacral plexus and cauda equina was met in all cases. The target coverage was acceptable both in VMAT and IMPT, although IMPT coverage tended to be better. Our study found that IMPT was superior in delivering dose to those areas of the target in close proximity to the sacral plexus.

**Conclusion**: IMPT offers a dosimetric benefit which may be clinically relevant for a patient subset with advanced sacral chordoma. Location of the tumour, anatomy of surrounding structures and clinical background need to be considered. (Table 1 - Comparative dose VMAT and IMPT).

### P 055: Reductions in cardiac Substructure Dose among Hodgkin Lymphoma Patients through Various Advances in Treatment Technique

#### J. Bates^1^, S. Flampouri^2^, D. Louis^2^, N. Mendenhall^2^, B. Hoppe^2^

##### ^1^University of Florida College of Medicine, Radiation Oncology, Gainesville, USA, ^2^University of Florida, Radiation Oncology, Jacksonville, USA

**Purpose**: Radiotherapy has evolved over time in the management of Hodgkin lymphoma (HL) in an effort to reduce late effects. We investigated the impact or RT field design and treatment technology on radiation dose to the heart and cardiac substructures.

**Materials/Methods**: Nineteen patients with mediastinal HL were treated with involved-site radiotherapy (ISRT) and had 3D conformal (3DCRT), and intensity modulated (IMRT), and proton plans developed, as well as a historical involved field RT (IFRT) plan. Cardiac substructures including both atria, both ventricles, the left anterior descending artery (LAD), the tricuspid, mitral, and aortic valves were contoured for each case. Dosimetric comparisons were calculated as percentage dose relative to the IFRT dose.

**Results**: When compared with IFRT, ISRT delivered with 3DCRT had a 21.3% reduction in mean heart dose. IMRT offered substantial improvement over 3DCRT plans with a 27.1% reduction in mean heart dose and an average reduction of 30.9% to each substructure. PT offered the largest incremental improvement with a 30.0% reduction in mean heart dose over IMRT. The left-sided chambers had more improvement (61.2% and 45.0% reduction in dose to left ventricle and atrium, respectively) relative to right-sided chambers (34.6% and 31.6% reductions in dose to right ventricle and atrium, respectively).

**Conclusions**: Advancing treatment techniques offer progressive benefits in cardiac dose reduction with PT plans resulting both in the lowest doses to the whole heart and cardiac substructures and the largest incremental improvement over prior treatment technique to each.

### P 056: Preoperative Pencil-Beam Proton Therapy Reduces Dose to Surgical Skin Flaps and Uninvolved Bone for Soft-Tissue Sarcomas of the Lower Extremity

#### S. Nurkic^1^, B. Wilke^2^, Z. Li^3^, X. Shen^3^, M. Ho^3^, D. Indelicato^3^, C.P. Gibbs^2^, A. Spiguel^4^, M. Scarborough^5^, M. Rutenberg^3^

##### ^1^University of Florida College of Medicine, Radiation Oncology, Gainesville, USA, ^2^University of Florida College of Medicine, Orthopaedics and Rehabilitation, Gainesville, USA, ^3^University of Florida, Radiation Oncology, Jacksonville, USA, ^4^University of Florida College of Medicine, Department of Orthopaedics and Rehabilitation, Gainesville, USA, ^5^University of Florida College of Medicine, Department of Orthopaedics and Rehabilitation, Jacksonville, USA

**Purpose/Objective(s)**:40% of patients treated with preop radiotherapy for soft tissue sarcoma (STS) of the lower extremity develop severe wound complications. Reduced dose to the surgical flap and uninvolved tissue can reduce rates of severe wound complications. Photon and proton treatment plans were evaluated for target coverage and dose avoidance to surgical skin flaps, uninvolved bone and soft tissues.

**Materials/Methods**: Plans for 5 patients with lower extremity STS were generated using volumetric arc radiation therapy (VMAT), passively scattered protons (PS) and pencil beam scanning protons (PBS). RTOG 0630 guidelines were followed. Plans prioritized: 1) target volume coverage, 2) dose constraints to surgical flap, and 3) avoidance of uninvolved bone. Prescription dose was 50 Gy in 25 fractions.

**Results**: Mean dose to the surgical flap was 38.3 Gy RBE (range, 28.1-45.6), 46.1 Gy RBE (range, 38.1-53.1), and 43.4 Gy RBE (range, 33.4-49.1) for the PBS, PS, and VMAT plans, respectively. Mean V30 of the surgical flap with PBS was 69.4% (range, 48.0- 87.1%) compared to 86.8% (range, 72.7-98.9%) and 82.8% (range, 55.9- 98.4%) with PS and VMAT, respectively. All patients had improved mean flap dose and flap V30 using PBS. Mean uninvolved bone V40 was 6.5% (range, 2.1-10.7%), 22.3% (range, 5.8-43.0%), and 14.6% (range, 2.1- 37.8%) using PBS, PS, and VMAT, respectively.

**Conclusion**: PBS achieved the lowest mean dose and V30 to the surgical flap, and the lowest bone V40 compared to PS and VMAT. Clinical outcomes are needed to determine if these benefits reduce treatment complications.

### P 057: The Impact on Pencil Beam Scanning (PBS) Proton Therapy for Mediastinal Lymphoma from Deep Inspiration Breath-Hold (DIBH) Variability

#### J. Olofsson^1,2^, M. Enmark^2,3^, S. Ceberg^3^, J. Jonsson^1^

##### ^1^Umeå University Hospital, CMTS - Radiation physics, Umeå, Sweden, ^2^Skandionkliniken, Medical physics, Uppsala, Sweden, ^3^Skåne university Hospital, Department of Hematology- Oncology and Radiation Physics, Lund, Sweden

Deep inspiration breath-hold (DIBH) technique used in combination with pencil beam scanning (PBS) proton therapy requires the fractional dose to be delivered during several breath-holds. Small anatomical inter breath-hold variations may therefore disturb the planned dose distribution. The aim of this study was to investigate the impact of such DIBH variability on PBS proton therapy for mediastinal lymphoma.

Ten different breath-hold images, all within a 3 mm range in vertical chest position, were acquired using MR-scanning. The anatomy was then transformed by deformable image registration onto the CT-study used for treatment planning, creating 10 different DIBH CT-studies. Each proton treatment field has been divided into four separate sub-fields to reflect the number of breath-holds needed for the actual treatment delivery sequence in the clinic. Finally, a set of 10 complete treatment series were created by summing up 17 unique fraction doses (in total 29.75 Gy-RBE) generated from sets of randomly assigned breath-holds in each fraction (Figure 1 - “Illustration of the workflow”).

The results show that there generally is a noticeable discrepancy between the planned dose distribution and a single simulated fraction dose. For CTV the D98% decreases and the D2% increases by 3% on average and in rare cases even up to 8% for a single fraction. However, averaged over a complete series of 17 fractions the deviations from the planned dose distribution are small, for CTV within 1%, indicating that the DIBH technique is a suitable delivery mode for PBS proton therapy for mediastinal lymphoma (Figure 2 - “Results”).

### P 058: Proton Radiotherapy for Locally Advanced Nasopharyngeal Cancer: Early Outcomes at a Single Institution

#### B.K. Sasidharan^1^, S. Aljabab^1^, T. Wong^2^, G. Laramore^1^, U. Parvathaneni^1^, J. Liao^1^

##### ^1^University of Washington, Radiation Oncology, Seattle, USA, ^2^Seattle Cancer Care Alliance, Medical Physics, Seattle, USA

**Purpose**: Proton therapy (PT) offers a dosimetric advantage over Intensity Modulated Radiation Therapy (IMRT) in Nasopharyngeal Carcinoma (NPC) due to close proximity of tumor to numerous critical structures. We report our initial clinical outcomes.

**Methods**: We reviewed treatment records of patients enrolled on an IRB-approved prospective clinical registry study who received proton therapy for definitive treatment of NPC. The demographics, dosimetry, acute and late toxicities and clinical outcomes were collected. Analyses were done using descriptive statistics.

**Results**: 14 patients were treated from 2015-2017 and had at least 3 months follow up. Median age was 58. 78% were male, 64% were EBV-associated, 43% WHO type 1 and 50% type 3. Most had locally advanced disease, 93% were stage III/IV (64% with T3-4 and 57% with N2-3). A pencil beam scanning approach was used with 2-5 beams encompassing primary and bilateral neck. Most patients were treated to 69.96 CGE, dose painted, in 33 fractions with concurrent platinum-based chemotherapy. Acute toxicities included 71% grade 3 mucositis, 50% grade 3 dermatitis, no grade 4-5 toxicities. 85% had primary and nodal complete response at first follow up. With a median follow up of 10.2 months (IQR, 5.9-21.2), 12 of 14 patients remain disease free with one local recurrence (T4 primary WHO I) and one distant recurrence. Updated follow up will be presented at the meeting.

**Conclusions**: Proton therapy is feasible and with encouraging early locoregional control and toxicity outcomes in locally advanced NPC.

### P 059: Proton Therapy for Locally Advanced Oropharyngeal Cancer: Initial Clinical Experience

#### S. Aljabab^1^, A. Lui^1^, T. Wong^2^, J. Liao^1^, G. Laramore^1^, U. Parvathaneni^1^

##### ^1^University of Washington, Radiation Oncology, Seattle, USA, ^2^Seattle Cancer Care Alliance, Medical Physics, Seattle, USA

**Purpose**: Proton therapy (PT) offers dosimetric advantages over IMRT for the treatment of oropharyngeal cancers, and has the potential to decrease toxicity.

**Methods**: We retrospectively reviewed our institutional experience with PT in oropharyngeal cancer. Survival, disease control, toxicity and tumor response outcomes were recorded from available medical records. Patients treated with primary or adjuvant proton therapy with or without chemotherapy were included. Descriptive statistics and Kaplan-Meier method were used.

**Results**: Thirty-nine patients were treated from March 2015 to August 2017. Median age was 58 (IQR, 53-67), 94.8% were male, 66.6% non-smokers, 97.4% had stage III/IVA disease, 89.7% were p16 positive. 23 patients received definitive radiotherapy to a total dose of 70-74.4 Gy(RBE) and 16 patients received adjuvant radiotherapy to 60-66 Gy(RBE), 64% received concurrent Chemotherapy . All patients received spot scanning proton therapy (single-field or multi-field optimizations with 3-4 beams in bilateral neck treatment, or 1-2 beams in unilateral neck treatment). At a median follow up time of 16.2 months (IQR, 9.4-22), 1 patient had distant recurrence and no patients had locoregional recurrence. Primary site complete response (CR) was 100%, nodal CR (<1.5cm) was 92.3%, 3 patients had a partial response but were PET/CT and biopsy negative. The 1-year PFS was 97.4%. An updated follow up will be presented at the meeting.

**Conclusions**: Our early clinical outcomes are encouraging with excellent PFS and no locoregional or marginal recurrences to date. Further follow up with greater patient numbers are needed along with comparative toxicity outcomes with IMRT treated patients.

### P 062: A Comparison of the Dose outside the PTV in Brain Gliomas in Proton (PRT) versus Photon (XRT) Radiotherapy

#### E. Pluta^1^, E. Gora^2^, A. Patla1, K. Kisielewicz^2^, D. Martynow^1^, A. Chrostowska^1^, T. Kajdrowicz^3^, D. Kabat^2^, B. Sas-Korczynska^1^

##### ^1^Centre of Oncology- Maria Sklodowska-Curie Memorial Institute Krakow Branch, Department of Oncology, Krakow, Poland, ^2^Centre of Oncology- Maria Sklodowska-Curie Memorial Institute Krakow Branch, Department of Medical Physics, Krakow, Poland, ^3^Institute of Nuclear Physics Polish Academy of Sciences, Bronowice Cyclotrone Centre, Krakow, Poland

**Purpose**: To compare the dose in the brain outside the PTV (B-PTV) in PRT versus XRT in patients with brain gliomas.

**Material**: Between December 2016 and November 2017 at the Maria Sklodowska-Curie Institute – Oncology Centre in Krakow, 26 patients received PRT (total dose 54 Gy(RBE) for brain gliomas (G1- 4 pts and G2 - 22 pts) as adjuvant therapy following surgery (65%) or after biopsy (35%). PRT was administered by pencil beam scanning generated in Proteus-235 cyclotron located at Bronowice Cyclotron Centre.

**Methods**: Two treatment plans were prepared for each patient: for the PRT (which was then executed) and for the XRT (IMRT technique) to compare selected dosimetric parameters: (i) mean dose for the CTV, PTV, B-PTV volumes, and (ii) B-PTV volumes that received 20% and 40% of the dose. Variance analysis with the t-test was conducted (significance level at p<0.05).

**Results**: We noted for PRT vs XRT that mean dose values were comparable: 52.7 Gy (RBE) and 52.8 Gy for the CTV and 52.6 Gy(RBE) and 52.7 Gy for the PTV, respectively. Statistically significant (p<0.05) differences were found for PRT vs XRT: (i) mean dose in the B-PTV: 12.6 Gy (RBE) and 19.7 Gy, respectively), (ii) B-PTV receiving 20% of the dose: 402.2 and 673.3 cm3, respectively), (iii) B-PTV receiving 40% of the dose: 224.3 and 440.7 cm3, respectively)

**Conclusions**: Our own observations in patients with gliomas confirm the benefit from using PRT, as compared to XRT, in terms of significant decrease of the dose in the brain outside the PTV.

### P 063: Can Robust Optimization Reduce Adaptive Planning Requirements Due to Anatomical Changes for Head and Neck IMPT Treatments?

#### M. Zhu^1^, K. Langen^1^, W. Regine^1^, J. Snider^1^, T. Houser^1^, N. Onyeuku^1^

##### ^1^Maryland Proton Treatment Center- Department of Radiation Oncology- University of Maryland School of Medicine, Radiation Oncology, Baltimore, USA

Adaptive planning is often needed for HN patients treated with IMPT due to weight loss or tumor shrinkage. Such planning is resource-intensive and can cause undesirable treatment disruption. This study investigates the potential of using robustness optimization (RO) to reduce adaptive planning frequency in HN patients.

In our center, bilateral HN IMPT usually utilizes four oblique fields: LPO, LAO, RAO, and RPO. Each field covers the ipsilateral target in the neck and superior, midline targets; the inferior part of the neck is covered by the two anterior oblique beams. Weekly rescan and dose evaluation are performed, and a new plan is created if dose distribution degrades significantly. Two patients requiring re-planning due to hot spot caused by weight loss (patient 1) and tumor shrinkage (patient 2) were selected for this study. Including RO accounting for 5mm isocenter position and 3.5% range variations, we retrospectively planned these two patients using two beam arrangements: one with the same 4-field angles as the treated non-RO plan; the other with 3 fields by replacing the two posterior oblique beams with a PA beam. Both plans were optimized to the same target coverage and OAR doses as the non-RO plan.

Table 1 shows substantial reduction of hot-spots versus non-RO planning with RO using 4-field or 3-fields. Figure 1 compares the dose distributions of the 3 plans for patient 2. IMPT plan robustly optimized against setup and range uncertainty can also account for anatomy/tumor changes seen in HN patients and therefore reduce adaptive planning frequency.

### P 066: Solute Ion Linear Alignment (SILA) Particle Acceleration to Replace 200+ Ton Synchrotron Magnets for Particle Therapy and Onsite Power Generation

#### A.N. Fresco^1^

##### ^1^Anthony N Fresco, Solute Ion Linear Alignment Acceleration, Melville New York, USA

Solute ion linear alignment propulsion was presented in ASME ES2010-90396 “Solute Ion Coulomb Force Monopole Motor and Solute Ion Linear Alignment Propulsion”. Solute ion linear alignment (SILA) is a process in which potential energy of the electrostatic fields of like charged solute ions is converted to kinetic energy and is based on the process of capacitive deionization (CDI). See http://contest.techbriefs.com/2014/entries/sustainable-technologies/4465

In ASME ES2010-90396, it was shown that 10E+16 ions of Na+ and Cl-, i.e., could propel themselves under a force of 10E+8 Newtons and a velocity of about 10E+8 m/second.

In ASME IMECE-2016-65930, “Solute Ion Linear Alignment as the Energy Source to Address Aquifer Depletion, Fresh Water Scarcity and Sea Level Rise”, it was shown that SILA is expected to be a power generation method by comparing the 3.6 KJ/liter for capacitive deionization cycle to as much as 5 MJ of energy per planar group of ions that form the SILA ion beams.

Therefore, dramatically reduced construction costs of current day particle therapy facilities compared to 200+ ton magnets and transformation from operating power consumption to onsite power generation may be achieved by SILA, which can be applied to particle therapy in several ways. For example, gaseous CO2 can be ionized by bombardment with Na+ and Cl- between electrode plates having a voltage across so as to separate the C and O2 ions to accumulate at the electrode surfaces. Then the ion linear alignment principle could be applied in the same way to create beams of C and O ions.

### P 067: Spot Position Calibration for Pencil Beam Scanning Commissioning and QA Using a Large Area Sensor at Isocentre

#### J. Gordon^1^, P. Boisseau^1^, D. Watts^1^, A. Dart1, M. Hanright^1^, S. Kollipara^1^, W. Nett^1^

##### ^1^Pyramid Technical Consultants Inc., Engineering, Lexington, USA

Accurate dose delivery for pencil beam scanning demands close control and monitoring of the lateral deflection of the particle beam. The beam spot position on the isocentre plane is affected by numerous factors including actual beam energy at the scanning magnets, incoming beam trajectory variation, non-ideal behaviour of the scan magnets, mechanical shifts and changing relationships between coordinate systems. In the worst case, calibrating the relationship between spot position on the isocentre plane and control settings for the scan magnets at all beam energies and gantry positions can require the collection of very large data sets. This requires considerable time and effort during commissioning and in subsequent QA checks.

We describe the design and use of a very large area (45 cm by 45 cm) position-sensitive ionization chamber with 1.75 mm strip pitch that can be accurately positioned at isocentre and used to sample the whole scan field at high spatial resolution in a single run. Using the nozzle system X-ray imager to locate the chamber in the patient coordinate system allows excellent position accuracy in the appropriate reference frame. Options are discussed for converting the measurements to scan calibration files. Linking the data collection to the scan control system allows the time-dependent evolution of the gamma index to be investigated.

### P 068: Development of Scanning Irradiation with Respiration Gating System for Proton Therapy

#### J. Inoue^1^, N. Hideki^1^, D. Amano^1^, T. Kato^1^, G. Shibagaki^1^, M. Araya^2^, Y. Sugama^2^, Y.Y. Han^3^, S.A. Hwan^3^

##### ^1^Sumitomo Heavy Industries- Ltd., Industrial Equipment Division, Niihama, Japan, ^2^Aizawa Hospital, Proton Therapy Center, Matsumoto, Japan, ^3^Samsung Medical Center, Proton Therapy Center, Seoul, Korea Republic of

**Purpose**: SHI has developed a scanning control system synchronized with respiratory gating for moving targets in collaboration with SHI supplied proton therapy sites at Samsung Medical Center in Korea and Aizawa hospital in Japan. The purpose of this article is to summarize the design and evaluations of the scanning control system adapted to respiration gating system for proton therapy.

**Methods**: SHI has developed the method of continuous line scanning to achieve high dose rate and short irradiation time. To ensure correct dose distribution for moving targets, SHI developed beam scanning control system which scans the continuous beam synchronized with the gating signal from respiratory gating system. And then added the circuit board which improves beam current intensity control especially in case beam on and off timing. After that the dosimetry evaluation has been done by combining re-scanning procedure and appropriate scanning direction to reduce the interplay effect.

**Results**: The performance tests of the beam scanning system have been conducted. The dosimetry evaluation by scanning with gating were performed equivalent to that of scanning without gating. The beam trajectory and intensity properties were same as the condition under non motion target irradiation. After optimizing the scan direction and executing re-scanning method, the gamma-index (3mm/3%) for comparison between dose distribution with target movement and dose distribution without movement indicate more than 95% at pass rate.

**Conclusions**: The scanning control system synchronized with respiratory gating for moving targets has been developed and the performance of the system has been confirmed.

### P 070: Radiance 330 Proton Therapy System Overview

#### D. Lee^1^, M. Amato^2^

##### ^1^ProTOM International, Physics, Wakefield, USA, ^2^ProTom International, Systems and Software, Wakefield, USA

The Radiance 330 is a U. S. FDA 510(k) cleared, proton therapy system, based on compact synchrotron technology, manufactured by ProTom International Holding Corporation. The Radiance 330 is the first proton therapy system specifically purpose-designed and purpose-built for proton therapy. The machine is compact and modular, consisting of 6 subsystems: Beam Production Subsystem, Beam Transport Subsystem, Beam Delivery Subsystem, Gantry Subsystem, Patient Position Subsystem, and Controls Subsystem. This compact modular design allows for a highly configurable machine able to be deployed in a variety of radiation oncology facilities. The compact synchrotron is an efficient, low radiation, economical, accelerator requiring significantly less radiation shielding than other designs; this, coupled with the scanned pencil beam, allows extraordinary treatment precision. The synchrotron is capable of delivering therapeutic proton beams in the clinical range of 70 MeV to 250 MeV, and is currently the only machine capable of delivering protons at up to 330 MeV for proton imaging capability. Future capabilities will include proton radiography and proton tomography.

### P 071: Fast and Accurate Position Fitting Algorithm for Dose Delivery Verification in HUST-PTF Scanning Nozzle System

#### Y. Lin^1^, T. Ping^1^, G. Huidong^1^, Z. Lige^1^, L. Hao^1^, Y. Yecheng^1^, L. Xingyu^1^

##### ^1^Huazhong University of Science and Technology, State Key Laboratory of Advanced Electromagnetic Engineering and Technology, Wuhan-Hubei, China

A proton therapy based on discrete spot scanning is under development in Huazhong University of Science and Technology. A strip ion chamber is adopted to verify the position in our facility; when compared with the pixel ion chamber, it can greatly reduce the amount of processed data and improve the response speed of the system with the same spatial resolution. In this paper, a kind of fitting algorithm is presented, which focuses on faster and more accurate position fitting for proton pencil beam scanning under the condition of big background current. The effects of the strip width and the signal to noise ratio are discussed. The original signal model is an ideal Gaussian signal, which is sampled, digitalized and added with white Gaussian noise. By changing the strip width and the signal to noise ratio, we determine under what circumstances the beam spot position resolution is able to meet the goal of less than 0.1mm. By means of excluding the data points with small amplitude values on the long tail of the Gaussian function curve to suppress the disturbance of the noise, we improve the fitting algorithm. The experiments of fitting algorithm are carried on the Labview platform. The results show that the improved fitting algorithm makes significant progress towards reducing the fitting error caused by the influence of low signal to noise ratio and increases fitting efficiency.

### P 072 - Treatment control implementation and commissioning for spot scanning mode in Shanghai Proton Therapy Facility

#### M. Liu^1^, C. Yin^1^, L. Zhao^1^, H. Shu^1^, K. Chu^1^, X. Dai^1^

##### ^1^Shanghai Institute of Applied Physics- Chinese Academy of Sciences, Department of Beam Instrumentation and Control Technology, Shanghai, China

A treatment control system receives treatment plans and drives nozzles to execute the treatment process. Based on independent research and development, a treatment control system was designed in Shanghai Proton Therapy Facility. After the implementation, the system is under commissioning with the whole treatment complex. The system acquires various physical quantities relative to treatment. Meanwhile, the system conducts irradiation processes, and protects personnel and machine from hazards. The spot scanning mode was realized in the commissioning, and the various physical indexes met the expected target. The system design and preliminary result of commissioning are presented in this contribution. (Picture 1 - “Structure of Treatment Control System”; Picture 2 - “Treatment Control System on Site”).

### P 073: Beam Intensity Dependence of a Secondary Electron Emission Monitor Used for Carbon-Ion Radiotherapy

#### M. Mizota^1^, Y. Tsunashima^1^, T. Himukai^1^

##### ^1^Ion Beam Therapy Center- SAGA HIMAT Foundation, Physics, Tosu, Japan

**Background**: For the control of the dose deposition, a couple of SEMs (secondary electron emission monitor) have been involved in each irradiation port using broad beam method in our facility. The main reason is that the output of the monitor is rather stable at high intensity of the carbon beam. However, the carbon beam in weak intensity have sometimes been used for the irradiation with the spiral-wobbling method and so, the intensity dependence of the SEM was investigated.

**Methods**: The output of an ionization chamber located at isocenter with a polyethylene block was measured for the constant output-count of the SEM in a standard irradiation field. The measurement has been repeated varying the beam intensity. We had analyzed the relationship between the beam intensity (the monitor-count per one beam-spill from the synchrotron) and the monitor-unit value (Gy/count).

**Results**: The results show that the output of the SEM gradually decreases as the beam intensity become weak. It keeps proportionality relation in the region down to the around 40% of the regular intensity used for radiotherapy with broad beam method.

**Conclusion**: There was a linear relationship between the beam intensity and the output of the monitor unit in some limited range, however it turned out that in the case of the weaker intensity it shows a behavior that deviates from that tendency.

### P 074: Automated GATE/Geant4 Beam Optics Modeling for Proton Beam Therapy

#### A. Resch^1^, C. Lee^1^, A. Elia^2^, D. Georg^1^, H. Fuchs^1^

##### *^1^Medical University of Vienna, Radiotherapy, Vienna, Austria*, *^2^EBG MedAustron GmbH, Medical Physics, Wiener Neustadt, Austria*

**Purpose and Introduction**: Beam modeling is a tedious and time consuming work but defines the accuracy of dose calculation. In this work we present an automated method to determine accurate beam model parameters, FWHM, divergence and emittance of a proton beam without user intervention.

**Material and Methods**: FWHMs of a pencil beam (PB) in air were derived from 2D dose distributions measured with a scintillating screen (Lynx) at various distances (10cm spacing) from the isocenter (ISD). Subsequently, FWHMs were derived from Gate/Geant4 Monte Carlo simulations using an initial guess of the initial spot size, divergence and emittance following the beam optics formalism. The best beam parameters minimizing the sum of the squared differences of the measured and simulated FWHMs were optimized using the genetic algorithm in Matlab2016b. While the Nozzle was moved out of the beam path to measure a training set for 5 energies (62-252MeV), measurements with Nozzle yielded as validation set.

**Results**: Figure 1a reveals a good agreement of the simulated FWHMs using the automatically derived beam model for all 5 energies over a 160cm range. The maximum deviation was 8% at ISD40cm. A clinically acceptable agreement was also found for the validation set (Figure 1b) suggesting that the optimized beam parameters were physically meaningful and the Nozzle was correctly modeled in GATE. (Fig 1: Simulated (circles) and measured (crosses) FWHM with and without Nozzle in a) and b), respectively).

**Conclusions**: Beam optics parameters were automatically derived for Gate/Geant4 for a proton beam line, enabling the creation of a beam model within a couple of hours on a conventional CPU.

### P 075: Two Years Results of the Clinical Use of the Proton Therapy Complex “Prometheus"

#### A. Shemyakov^1^, V. Balakin^1^, A. Pryanichnikov^1^, V. Sokunov^1^, N. Strelnikova^1^, T. Belyakova^1^

##### ^1^ZAO “ProTom”, Proton therapy, Protvino, Russian Federation

Proton therapy is one of the most accurate methods of radiotherapy and radiosurgery, but is still quite expensive. To address this, the low-cost compact proton therapy complex “Prometheus” was developed at ZAO “ProTom”. In November 2015, this complex started being used for the clinical treatment of patients with head and neck cancers. The heart of the complex is a compact five meters in diameter synchrotron, which is able to accelerate protons up to 330 MeV.

The main feature of this complex combines a cost effective device with modern advanced treatment technologies: pencil-beam scanning, intensity modulated proton therapy, build-in cone-beam CT (CBCT). Instead of a gantry, the complex includes a robotic armchair rotating to 360 degree, specially designed to fast fixation and patient alignment correction with accuracy of 0.1mm (Picture 1 - “Patient positioning system”). It is possible to use build-in CBCT for proton dose planning.

Today more than 200 patients with different head and neck tumors have been treated in this complex. Average time for one fraction with fixation and verification procedures was 10 minutes (Picture 2 - “Time for one fraction”). The average value of the electric power consumed by synchrotron for the full course of treatment of one patient is 56.8 kWh.

Because such sitting positions are quite new for proton treatment, patient position displacement have been measured during CBCT verification. The obtained results were slight for each of the dimensions: at the X-axis: -0.1±0.8 mm, Y-axis: -0.4±2.0 mm, Z-axis: 0.1±1.5 mm. More than 2000 verification procedures in summary were held for all patients.

### P 076: Simulation of Beam Transport in the Nozzle of HUST-PTF

#### L. Zhang^1^, P. Tan^1^, J. Huang^1^, C. Zuo^1^, H. Guo^1^, Y. Lin^1^, X. Li^1^

##### *^1^Huazhong University of Science and Technology, State Key Laboratory of Advanced Electromagnetic Engineering and Technology, Wuhan- Hubei,* China

HUST-PTF is a proton therapy facility under development in Huazhong University of science and technology now. It delivers the beam to the patients with a pencil beam scanning nozzle. The dose is determined by the accuracy of the beam transport in the nozzle, so particle tracing and Monte Carlo simulation is necessary for the study of the beam transport in the nozzle. In this study, the recently designed nozzle at our institute was simulated to study the beam's profile and energy which considering the magnetic field of the scanning magnet and the beam matter interaction with the gas and monitors inside the nozzle. The designed twiss parameter at the end of the gantry beam line is used as the initial beam conditions, the scanning area is calculated as a range of 300*300mm and the RMS beam size of 3.5-6 mm in the isocenter varying with the energy. The relationship between the beam size and energy is given in an analytical way. The influence of the energy spread and imperfection of the gantry beam line are also taken into consideration. This study is important for the verification and commissioning of the nozzle system.

### P 077: Which Machine to Choose: Comparison of Compact Proton Therapy Systems

#### Y. Zheng^1^, J. Wong^1^

##### ^1^Atlantic Health System, Radiation Oncology, Morristown, USA

**Purpose**: Compact proton therapy systems have become a popular choice for new proton therapy centers due to its affordability, low maintenance cost and potential high throughput per room. The goal of this study is to present a detailed comparison of compact proton units and considerations for selection from a customer's perspective.

**Methods**: Preliminary investigation was done for all major particle therapy vendors, with 5 of them selected for detailed comparison based on our facility need. We compared each proton unit in several major categories including room dimension and layout, capital and operating cost, beam properties, image guidance technique, scalability, potential patient throughput and so on. Considerations on how to choose a proton unit that best fits the customer's needs are also discussed.

**Results**: A comparison of selected compact proton therapy systems is shown in Table 1. The machine cost ranged from $22M to $29M, with additional cost for construction and maintenance. The dimension of each system varies largely with vendor, accelerator type, and gantry rotation angles. All systems provide similar maximum proton energy/treatment depth, but vary largely on dose rate and field size. In room CT is available for all vendors.

**Discussion/Conclusion**: Proton therapy technology has been evolving quickly, with more compact systems of lower cost and high efficiency being available. A comprehensive understanding of true machine performance, future technology upgrade and scalability, both capital and operating costs, and the customer's clinical and space needs is critical to build a proton therapy center that best fits the clinical and financial goals.

### P 078: Performance Simulation of Scanning Magnets on Dose Uniformity Using Geant4 and ELEKTRA

#### X.Y. Li^1^, P. Tan^1^, H.D. Guo^1^, C. Zuo^1^, J. Huang^1^, Y.Y. Hu^1^, Y.J. Lin^1^

##### ^1^Huazhong University of Science and Technology, State Key Laboratory of Advanced Electromagnetic Engineering and Technology, Wuhan- Hubei, China

An active scanning proton therapy facility is being developed at Huazhong University of Science and Technology (HUST). By controlling the deflection position of the beam with scanning magnets at different times, the superposition of discrete spot beams will form a specified shape and dose distribution conformal to the target tumor. In this paper, the ELEKTRA module in Opera3D is used to model and simulate the scanning magnets and obtain the corresponding real magnetic field distribution at different time. Then the magnetic field distribution at different time is imported into Geant4 to establish the accurate scanning magnet model, and the spot dose distribution profile at iso-center plane is calculated. By combining ELEKTRA and Geant4, the corresponding magnetic field distribution and beam trajectory changes under different scanning magnet currents were analyzed. The influence of beam deflection angle on beam spot size was studied. The total lateral dose distribution curve was obtained by superimposing the lateral dose distribution curve on the iso-center plane at different moments. The simulation of 70-230 MeV proton beam shows that the proton therapy facility developed by HUST can effectively change the beam deflection angle and meet the requirement of lateral dose uniformity.

### P 079: Design of Electrostatic Deflector in the Shanghai Advanced Proton Therapy Facility

#### Z.F. He^1^, L.H. Ouyang^1^, D.M. Li^1^

##### ^1^Shanghai Institute of Applied Physics- Chinese Academy of Sciences, Department of Applied Accelerator, Shanghai, China

In this article, we introduce the design of electrostatic deflectors for injection system and extraction system in the Shanghai Advanced Proton Therapy facility (SAPT); describe the choice of electrode materials, the mechanical design and the compact high-voltage feedthrough; and give the calculated results of the distribution of the electric field around the electrode and the simulated results of the proton beam. We also discuss the problems during the commissioning and its solutions.

### P 081: Physical Characterization of the Mevion HYPERSCAN Energy Selector

#### M. Kang^1^, H. Chen^1^, R. Cessac^2^, D. Pang^1^

##### *^1^Georgetown University Hospital, Radiation Medicine, Washington DC, USA*, *^2^Mevion Medical Systems, Engineering Department, Littleton, MA, USA*

**Purpose**: To evaluate the physical and dosimetric characteristics of the HYPERSCAN energy selector (ES), and study how such a radical design may impact the traversing proton beam and any ensuring potential implications in planning for patient treatment.

**Materials and Methods**: The ES (Fig.1) is composed of 18 Lexan plates with various thicknesses from 0.2 to 6.6 cm WET to create a combination of 156 energies with range increment of 2 mm WET. The beams are further trimmed by the Adaptive Aperture (AA) before entering patients. A PTW Markus chamber was used to measure the central axis IDDs of 10x10 cm2 spot map in water for 6 selected energies. In-air spot profiles were measured using EBTs films crossed check with Raven scintillation detector at planes of ISO, +10, +20, -10, and -20 cm away from ISO.

**Results and Discussion**: Fig.2 (a) shows representative IDD curves measured at six select energies. Due to absent of dipole magnets for energy selection the IDD curves, all energies beams have the similar peak shape, with the distal 80%-20% falloff at 4.6mm and 80-80% Bragg peak widths at 8mm. Fig.2 (b) shows that the spot size is comparable to the other proton systems at the highest energy of 227MeV with a beam divergence of 2.5mrad, while for lower energies<100 MeV, the beam divergence become progressively bigger. Therefore minimal air gap and optimal nozzle extension combined with AA are essential to maintain smaller spot size and sharper penumbra to deliver more conformal dose distribution for lower energy beam.

### P 082: Commissioning Status of the First Chinese Domestic Proton Facility in Shanghai

#### H. Kong^1^, Z. Chen^1^, D. Li^1^, X. Li^2^, Y. Pu^1,2^, L. Shen^1^, C. Yin^1^, N. Yan^1^, M. Zhang^1^, Z. Zhao^1^

##### *^1^Shanghai Institute of Applied Physics- Chinese Academy of Sciences, Shanghai Synchrotron Radiation Facility, Shanghai, China*, *^2^Shanghai APACTRON Particle Equipment Co.-Ltd, Research and Development, Shanghai, China*

**Purpose**: The Ruijin hospital proton center, located in Shanghai, is the first domestic proton facility in China. The synchrotron-based therapy system (SAPT), built by Shanghai Institute of Applied Physics, Chinese Academy of Sciences, is now under commissioning. The purpose of this study is to describe the comprehensive commissioning process and the status of the system.

**Methods**: The treatment system delivers clinical beams of protons (70-235 MeV) to one experiment room, one eye-treatment room (double scattering), one fixed-beam treatment room and one Gantry room (both active scanning). The Raystation 6.1 and MOSAIQ are employ as TPS and OIS. Two treatment rooms are equipped with 6D robotic couches and X-ray-based image guidance system. The AAPM reports, IAEA reports and ICRU 78 has been studied as guidelines. Treatment control software, patient positioning and verification system, 6D robotic couch should fulfill the IEC standards.

**Results**: The technical commissioning of SAPT has been started in May 2017.

Beam data from Monte Carlo simulations and measurements are collected for TPS modeling.

**Conclusions**: The preliminary beam data measurements, TPS modeling and Validation are reported. The comprehensive end-to-end tests will start in the second half of 2018.

### P 083: The Treatment Terminal System of HIMM

#### G. Li^1^, J. Shi^2^, X. Liu^3^, R. Mao^4^

##### *^1^Institute of Modern Physics-Chinese Academy of Sciences, Information Techenology Group, Lanzhou, China*, *^2^Institute of Modern Physics-Chinese Academy of Sciences, Accelerator Physics Group, Lanzhou, China*, *^3^Institute of Mordern Physics-Chinese Academy of Sciences, Medical Physics Group, Lanzhou, China*, *^4^Institute of Modern Physics-Chinese Academy of Sciences, Beam Diagnostics Group, Lanzhou, China*

HIMM (Heavy Ion Medical Machine) is the first Chinese heavy ion accelerator facility dedicated to medical purposes and will be ready to open clinical trials in 2018.The treatment terminal of HIMM achieves the treatment process control and treatment security. At present, the treatment terminal provides two modes of treatment: uniform scanning and raster scanning. The treatment terminal contains ten subsystems, namely Oncology Information System, Recording & Verification System, Treatment Control System, Dose Delivery System, Beam Modulation System, Patient Positioning System, Patient Positioning Verification System, Independent Termination System, Medical Interlocking System, and online Energy Verifying System. Treatment Control System will automate the treatment process; Dose Delivery System and Beam Modulation System will achieve accurate dose control and beam position control; Oncology Information System and Recording & Verification System will achieve the treatment plan data and treatment process data validation and recording; Patient Positioning System And Patient Positioning Verification System will achieve the patient's precise positioning, positioning accuracy guaranteed within 1mm; and the Medical Interlocking System, Independent Termination System, and online Energy Verifying System will achieve the safety of the treatment process. This article describes the structure of the treatment terminal and how those subsystems work together under different real-time requirements.

### P 084: Design and Commissioning of the First Worldwide Scanning Proton Beamline for Ocular Treatments

#### E. Mastella^1^, G. Magro^1^, A. Mirandola^1^, S. Molinelli^1^, S. Russo^1^, A. Vai^1^, M.R. Fiore^2^, C. Mosci^3^, F. Valvo^4^, M. Ciocca^1^

##### *^1^CNAO Foundation – National Centre for Oncological Hadron Therapy, Medical Physics Unit, Pavia, Italy*, *^2^CNAO Foundation – National Centre for Oncological Hadron Therapy, Clinical Radiotherapy Unit, Pavia, Italy*, *^3^Galliera Hospital, Ocular Oncology Center, Genoa, Italy*, *^4^CNAO Foundation – National Centre for Oncological Hadron Therapy, Clinical Directorate, Pavia, Italy*

**Purpose**: Worldwide, around 10 centers treat ocular tumors with proton therapy, all with dedicated beamlines. Our facility is equipped with general-purpose fixed beamlines; protons and carbon ions are delivered with pencil beam scanning modality (range in water3÷32cm). Recently, our proton beamline was adapted to treat ocular melanoma.

**Methods**: A 3cm range shifter (RS) was placed along the beamline to shift minimum penetration depth. The position of the RS and patient-specific collimator were simulated using FLUKA code to achieve steep lateral dose gradients. To evaluate lateral penumbra and dose homogeneity, dose profiles were measured with radiochromic films. Depth-dose distributions (DDDs) were measured with the Peakfinder system; the relative weight of each DDD were optimized simulating different targets to obtain uniform dose distributions (SOBPs). Absorbed doses were measured in water with Markus chamber. Neutron dose at the contralateral eye was evaluated with passive bubble dosimeters.

**Results**: FLUKA simulations and experimental results confirmed that maximizing the distance between RS and collimator increases transversal dose homogeneity and reduces lateral penumbra of the collimated beam. RS and patient-specific collimator were placed at 98cm (behind beam monitors) and 6cm from the isocenter, respectively. The lateral penumbra ranged between 1.0÷1.8mm. The measured SOBP doses were in very good agreement with FLUKA simulations (see Figure1). The mean neutron dose at the contralateral eye was (68.8±10.2)μSv/Gy.

**Conclusions**: Our adapted eyeline satisfied the requirements to treat ocular tumors. The first ocular patient was treated in August 2016. So far, around 60 patients have been treated with prescription doses of 52-60GyE.

### P 085: The Christie Proton Therapy Research Facility

#### M. Merchant^1^, M. Taylor^1^, A. Chadwick^1^, N. Kirkby^1^, T. Mee^1^, H. Owen^2^, R. Mackay^3^, K. Kirkby^1^

##### *^1^University of Manchester, Division of Cancer Sciences- Manchester Academic Health Science Centre, Manchester, United Kingdom*, *^2^University of Manchester, School of Physics and Astronomy- Schuster Laboratory, Manchester, United Kingdom*, *^3^The Christie NHS Foundation Trust, Christie Medical Physics and Engineering, Manchester, United Kingdom*

The UK government has committed £250 million to developing high-energy proton beam therapy services in the UK. Two facilities are currently in construction located at the Christie NHS Foundation Trust, Manchester, and University College London Hospitals NHS Foundation Trust.

The Christie proton beam therapy centre will host three clinical treatment rooms featuring a ProBeam Proton Therapy System supplied by Varian Medical Systems. A fourth room at the Christie Proton Therapy Centre will be dedicated to proton beam therapy research.

This research room, will house two static horizontal beam lines with the capability for spot scanning beam delivery; one primarily for radiobiological experiments and the other for physical and technical investigations. The research beamline is currently in the design phase and will be completed in late 2018. The design philosophy for the research room beamlines and layout is to provide modular, adaptable end-stations for each beamline that will allow an extensive range of experiments to be performed. The research room will be a UK national facility for proton therapy research.

We present the design of the research beamlines, modular end-stations, and an overview of the research room experimental capabilities.

### P 086: The interdisciplinary BNCT Project at the Universities Okayama and Nagoya

#### W. Sauerwein^1,2^, K. Tsuchida^3^, Y. Kiyanagi^3^, H. Matsui^4^, K. Igawa^2^, S. Furuya^2^, Y. Ichikawa^2^

##### *^1^University Hospital Essen, NCTeam- Department of Radiation Oncology, Essen, Germany*, *^2^Okayama University, Neutron Therapy Research Center, Okayama, Japan*, *^3^Nagaoya University- Graduate School of Engineering, Research Laboratory of Accelator-based BNCT System, Nagoya, Japan*, *^4^Okayama University Medical School, Dept. of Physiology, Okayama*, *Japan*

The non-availability of hospital based epithermal neutron sources for BNCT has considerably impeded any development of this modality. Only recently, powerful proton accelerators have become available to produce an epithermal neutron flux reaching an intensity similar to reactor-based sources.

Five proton accelerators are installed, of which two are already treating patients in the frame of clinical trials (STGH Koriyama and KURRI). Several other groups are making efforts to build up such a modality. We will present the ongoing project at the universities in Okayama and Nagoya.

The neutron source in Nagoya is based on a Dynamitron from IBA accelerating protons up to 2.8 MeV. Bombarding with high intensity an innovative liquid Li-target neutrons are produced using the 7Li(p,n)7Be threshold reaction. This has the advantage of neutron spectra with maximum energies of less than 1MeV, that is much lower than those obtained by Be-targets or as for the uranium fission spectrum, relevant for BNCT at nuclear reactors. The slowing down of the neutrons to about 10 keV that is needed to treat a deep-seated tumor, is related with less loss of neutrons during the process, less activation of the moderator and therefore less challenges for radiation protection issues.

Previously, no international rules were fixed to guide the regulatory processes. Now a discussion has started how to overcome these challenges.

### P 089: Clinical Commissioning and Acceptance Test for a New Scanning Beam Delivery Room at SAGA HIMAT

#### Y. Tsunashima^1^, T. Himukai^1^, Y. Hara^2^, M. Mizota^1^, H. Sato^3^, T. Furukawa^4^, Y. Shioyama^3^

##### *^1^Ion Beam therapy center- SAGAHIMAT Foundation, Physics, Tosu, Japan*, *^2^B dot Medical, B dot Medical, Chiba, Japan*, *^3^Ion Beam Therapy Center- SAGAHIMAT Foundation, Clinic Section, Tosu, Japan*, *^4^B dot Medical Inc, B dot Medical Inc, Chiba*, *Japan*

SAGA HIMAT started the carbon-ion radiotherapy with a beam port using the broad beam method since 2013. We originally had three treatment rooms. Two of those are successfully in operation and have currently treated more than 2,430 patients. A new scanning beam system project in the third treatment room was begun in 2014. Construction in the treatment room and installation of new beam lines were carried out from midnight after daily operation or on weekends without interrupting regular operation, including QA measurement and treatment in other treatment rooms. There was no major problem influencing daily operation. A machine performance test, which was mainly performed by a vendor, was achieved in October 2017. Since then, an acceptance test and clinical commissioning was started. The new treatment room employs hybrid depth scanning, which utilizes 11 energies from 400MeV/u∼100MeV/u with several rang shifters. There are two fixed beam lines (horizontal and vertical). The beam size at the iso-center is 2 mm ∼ 6 mm with a combination of two ripple filters, 2mm and 1mm. The maximum field size is 240x240mm2. The result of the clinical commissioning of the beam delivery system and treatment planning, as well as the acceptance testing, will be presented.

### P 090: Commissioning Status of the MedAustron Particle Therapy Accelerator

#### P. Urschuetz^1^, G. Kowarik^1^, M. Pivi^1^, M. Stock^2^, L. Grevillot^2^, V. Letellier^2^

##### *^1^EBG MedAustron, Therapy Accelerator, Wiener Neustadt, Austria*, *^2^EBG MedAustron, Medical Physics, Wiener Neustadt, Austria*

MedAustron is a state-of-the-art synchrotron based light-ion beam therapy centre located in the city of Wiener Neustadt in Austria. In addition to three clinical treatment rooms the centre has a dedicated irradiation room to carry out non-clinical research covering radiation biology, medical and experimental physics.

The heart of the facility is the so-called Therapy Accelerator, a CE certified medical device in accordance to the European Medical Device Directive. It is manufactured, maintained and operated by MedAustron. The accelerator design follows the lines of modern light-ion therapy accelerators, designed for providing mainly proton and carbon ion beams with a penetration depth of about up to 37 cm in water-equivalent tissue.

Experts from various fields are continuously working on the operation, maintenance, further commissioning and development of the Therapy Accelerator within the framework of an ISO 13485 certified Quality Management System.

MedAustron started research operation in August 2016. Patient treatment commenced in December 2016 in one irradiation room with a horizontal fixed beamline providing proton irradiation. Currently, two treatment rooms are in operation and commissioning by medical physicists of the vertical beamline is progressing. In full operation the maximum capacity of the facility shall reach up to 1000 patients per year.

The poster gives an overview of the design and status of the Therapy Accelerator and outlines the upcoming commissioning tasks.

### P 092: Reconstruction of a Dosimetric Equivalent Treatment Couch for a New Pencil Beam Scanning Proton Therapy Facility

#### J. Yu^1^, J. Forthomme^2^, D. Wikler^2^, W. Hsi^1^, S. Rana^1^, J. Bennouna^1^, L. Coutinho^1^, A. Gutierrez^1^

##### *^1^Miami Cancer Institute, Radiation Oncology, Miami, USA*, *^2^IBA, Ion Beam Application SA, Louvain-la-Neuve*, *Belgium*

**Purpose**: To reconstruct the treatment couch to be taken into account dosimetrically accurately in the treatment planning system (TPS) via measurements of water equivalent thickness (WET) of the couch.

**Methods**: The treatment couch contains a couch top and a base. The same couch top was installed in the CT scanner and it is scanned with patients. The base is not installed in the CT, but it has to be accounted in dose calculation for the beams going through the base. Measurements of the couch WET was conducted by a multi-layer ionization chamber. Based on the measured WET values, a dosimetric equivalent couch was reconstructed in the TPS. The reconstruction takes into account both material composition and density.

**Results**: The measured WET of the uniform body of the base was 0.69 (+/- 0.01) cm, and the WET of superior boarder has a nonlinear tapering. Figure 1(a) shows the CT in sagittal view for the base, and (b) shows the measured WET at the same longitudinal locations. Figure 1(c) shows the reconstructed base. By applying a uniform equivalent physical density, the geometric profile of the reconstructed base resembles the measured WET profile. Table 1 lists the measured vs. simulated WET.

**Conclusions**: The couch base and its wedge-shaped superior boarder should be included in the dose calculation to account for the range change of the proton beams caused by the base. A dosimetric equivalent couch base can be reconstructed based on the WET measurements, and verified by the simulation of the clinical TPS.

### P 093: Operational Metrics of a Compact Proton Therapy System: Two-Year Experience

#### O. Zeidan^1^, E. Pepmiller^1^, E. Foster^1^, T. Willoughby^1^, S. Meeks^1^, Z. Li^1^, N. Ramakrishna^1^

##### *^1^Orlando Health-UF Health Cancer Center, Radiation Oncology, Orlando*, *USA*

We report on various operational metrics of our Mevion S250 compact double scattering proton system. A total of 227 patients were treated from April 2016 to December 2017 with the following site breakdown: 41% prostates, 17% adult CNS, 17% pediatrics, 9% H&N, 5% thorax/lung, and 6% breast/chest wall. A total of 6900 fractions were delivered. The highest number of fractions treated in a given month was 494. Total number of fractions cancelled due to machine-specific downtime was 174 (2.5% of all delivered fractions) corresponding to an overall uptime of 97.5%. The average (standard deviation) for daily treatment times from end of localization to end of beam delivery was 3.8 (0.8) min for prostates (single beam/day), 13.2 (2.7) min for brain and 15.0 (5.0) min for breast/chest walls. A total of 725 beams were delivered. The maximum diameter projection at isocenter for all small applicator beams (n = 535) was 10.4 cm and 22.1 cm for large applicators (n = 190). The mean air gap for small applicator beams was 8.0 cm and 10.3 cm for large applicators. The majority of gantry angles used for treatments were 90 and 180 degrees (40% of all angles). The most widely used robotic couch angles were 0, 180, and 270 degrees (86% of all couch angles). We were the first proton center to use the Pinnacle treatment planning system and AIRO 32-slice mobile CT for in-room anatomy verification and localization.

### P 094: IPEM Code of Practice for Proton and Ion Beam Dosimetry: Update on Work in Progress

#### S. Green^1^, R. Amos^2^, F. Fiorini^3^, F. Van den Heuvel^4^, A. Kacperek^5^, A. Lourenco^6^, R. MacKay^7^, H. Palmans^6^, J. Pettingell^8^, D. D'Souza^9^, R. Thomas^6^

##### *^1^University Hospital Birmingham NHS Foundation Trust, Medical Physics, Birmingham, United Kingdom*, *^2^University College London, Medical Physics and Biomedical Engineering, London, United Kingdom*, *^3^University of Oxford, CRUK and MRC Oxford Institute for Radiation Oncology, Oxford, United Kingdom*, *^4^Churchill Hospital, Radiotherapy Physics, Oxford, United Kingdom*, *^5^The Clatterbridge Cancer Centre, The National Eye Proton Therapy Centre, Bebington, United Kingdom*, *^6^National Physical Laboratory, Medical Radiation Science, Teddington, United Kingdom*, *^7^Christie Hospital, Medical Physics and Engineering, Manchester, United Kingdom*, *^8^The Rutherford Cancer Centres Limited, Medical Physics, Newport, United Kingdom*, *^9^University College London Hospitals, Radiotherapy Physics, London, United Kingdom*

Current standard methods for reference dosimetry of proton and ion beams typically involve the use of an ionization chamber calibrated in a cobalt-60 beam, with a beam quality correction factor applied to account for the difference between the chamber response in the proton and the calibration beams.

We present an update on the development of a new Code of Practice (CoP) for reference dosimetry of proton beams. Key elements of the proposed protocol are:

1. The use of the Primary Standard NPL portable graphite calorimeter (figure 1) in proton beams at the user facility
2. Calibration in a composite field defined to cover what is termed a Standard Test Volume (STV) of delivered dose for both actively scanned and passively scattered beams
3. The primary or reference STV will have dimensions of 10 × 10 × 10 cm3 and be centred at 15 cm depth.

The proposed key steps for high energy actively scanned and passively scattered beams are shown in Figure 2. (Figure 1 - The NPL graphite calorimeter; Figure 2 - Key steps of the Code of Practice)

For low energy beams, key parameters such as size of the STV and reference depth must be reduced.

The CoP is under development and due for completion during 2018. The presentation will describe the proposed methodology with the aim of stimulating wider debate and comments on the approach.

### P 095: Long-Term Dose-Response of Gafchromic EBT 2 Film Irradiated by Proton Beam and γ- Rays

#### D. Borowicz^1,2^, G. Mytsin^2^, J. Malicki^1,3^

##### *^1^Greater Poland Cancer Centre, Department of Medical Physics, Poznań, Poland*, *^2^Joint Institute for Nuclear Research, Laboratory of Nuclear Problems, Dubna, Russian Federation*, *^3^University of Medical Sciences, Electro-radiology Department, Poznań, Poland*

This study presents the long-term dose-response of dosimetric film after irradiation by γ-rays and proton beam.

The EBT 2 Gafchromic film was used in this work. The detector was irradiated by proton beam produced by a synchrocyclotron with energies 110, 145, 180 and 215 MeV and by γ- rays. The EBT 2 film was cut into small pieces-squares. All small films were irradiated perpendicularly to the central axis beam (CAX) with different doses from 0.0 Gy to 9.0 Gy at intervals of 1.0 Gy. After 24 h, films were scanned by a flatbed Epson Perfection V750-M Pro Scanner (48-bit, 300 dpi). In ImageJ software the pixel value (PV) at the red canal of the RGB bit map was read for each irradiated film. The response of the EBT 2 film to irradiation was articulated by net optical density (netOD). Films were scanned several times over 5 months.

The results of this study show that the time after irradiation affects on the dose-response of the EBT 2 film. The darkening of irradiated film changes and the netOD increases with time after irradiation. We observed that boost of netOD is more evident for high doses of irradiation, 8 Gy, than for low doses, 2 Gy.

There is no significant difference in netOD between γ-ray and proton beam irradiation. The energy of the proton beam also does not affect to the response of the film detector. The length of time after irradiation does not change the correlation between different types of irradiation and energies.

### P 096: A FLUKA Monte Carlo Simulation Model for Scanning Proton Therapy

#### Y. Sheng^1^, J. Zhao^1^, N. Schlegel^1^, W. Wang^1^, J. Sun^1^, S. Guo^1^, Z. Huang^1^, Z. Chen^1^

##### ^1^Shanghai Proton and Heavy Ion Center, Medical Physics, Shanghai, China

**Purpose**: Ion therapy in our center is performed by using the proton and carbon ion scanning beam. The purpose of this study is to design a simulation model of the horizontal treatment nozzle, and to commission the scanning beam model for proton therapy in our hospital.

**Methods and Materials**: FLUKA was used for the simulation of proton transport. The geometry of the beam line was implemented into a FLUKA model. The model contained a vacuum beam pipe, a vacuum window, two beam profile monitor and spot-position monitors that consisted of a MWPC, two plane-parallel ionization chambers, and an intensity monitor chamber. IDD profiles for several proton beam energies of were simulated. The corresponding spot in-air lateral profiles at the isocenter were also simulated.

**Results**: The results of the simulations were compared with measured data of the proton beam. For the IDD profile simulation, a water phantom cylinder with a radius of 4.08 cm was located with its upstream surface at the isocenter plane, 415mm downstream of the surface of vacuum window. The depth dose deviations of the MC compared to measured data is about 2%, however major deviations of up to 6.5% were observed in certain regions. The proton sources were adjusted so that simulated in-air lateral profiles reproduced the measured data. Thus, a good agreement between measured and simulated in-air lateral profile data is expected.

**Conclusions**: The treatment nozzle and proton beam model were implemented by using the FLUKA Monte Carlo code. The model can be used for research purposes to simulate the scanning proton therapy.

### P 097: Impact of Dosimeters on the Accuracy of a Proton Therapy Treatment Planning System (TPS)

#### C.S. Lin^1^, A. Giebeler^2^, Y.C. Tsai^3^, C. Chong^1^, L.H. Lai^4^, C.W. Wang^3^, S.H. Kuo^3^, P. Wong^2^, A. Chang^2^

##### *^1^National Taiwan University Cancer Center, Radiation Science and Proton Therapy, Taipei, Taiwan\r*, *^2^California Protons Therapy Center, California Protons Therapy Center, San Diego, USA*, *^3^National Taiwan University Hospital, Radiation Oncology, Taipei, Taiwan\r*, *^4^National Tsing-Hua University, Biological Engineering and Environmental Sciences, Hsinchu, Taiwan*

**Purpose**: To access the impact of dosimeters, such as OSLD (NanoDot) and TLD-100H, on the accuracy of Eclipse V13.7.

**Materials and Methods**: Five OSLD and TLD-100H chips were placed on the surface of HPDC solid phantom. The evaluation was conducted on EclipseV13.7. A prescribed dose of 2Gy was delivered to a CTV (15 × 5 × 5 cm3) using the active scanning technique. The CTV was set at the depth of 3.3 cm (CTV3.3) and 20 cm (CTV20) to mimic shallow and deeper targets. The dose was delivered in present and absent of a 57 mm WeT range shifter (RS57). Percentage dose difference (%DoseDiff) at a depth of 2 cm WeT and center of SOBP was calculated to assess the perturbation.

**Results**: In absence of the RS57, the dose of the center of SOBP shadowed by the dosimeters was reduced by 0.40%, in general. Perturbation in present of the RS57 varied with the depth. For CTV3.3, The dose was increased by 2.15% (TLD-100H, 2 cm WeT), 2.92% (NanoDot, at 2 cm WeT), 0.98% (TLD-100H, at center of SOBP) and 1.56% (NanoDot, at center of SOBP). For CTV20, the dose was decreased less than 1.1% in both shallow and deeper region (Table I).

**Conclusions**: Both TLD-100H and NanoDot can cause dose perturbation and range shift especially in the presence of RS57. The Perturbation of NanoDot may be attributed by its 10mm x 10mm x 2mm plastic holder. To reduce the impact of the dosimeters on accuracy of the TPS, the number of the dosimeter and measurement should be considered carefully. This is particularly important when a ranger is used.

### P 098: Absolute Dose Calibration of the High-Speed Strip Ionization Chamber CROSS

#### C.H. Lin^1^, F.X. Chang^2^, P.R. Tsai^1^, P.K. Teng^1^, H.C. Huang^3^, C.C. Lee^4^, C.Y. Yeh^3^, A.E. Chen^5^

##### *^1^Academia Sinica, Institute of Physics, Taipei, Taiwan\r*, *^2^Liverage Biomedical Inc., Photonphysics, Taipei, Taiwan\r*, *^3^Linkou Chang Gung Memorial Hospital, Proton and Radiation Therapy Center, Linkou, Taiwan\r*, *^4^Chang Gung University, Department of Medical Imaging and Radiological Sciences, Linkou, Taiwan\r*, *^5^National Central University, Department of Physics, Taoyuan, Taiwan*\r

The high-speed XY strip ionization chamber, CROSS, is designed for the fast pencil beam scanning (PBS) system. The CROSS detector can measure proton beam at 20 kHz sampling rate with a fine spatial resolution. With such high sampling rate, it does not only determine precisely all characteristics (position, shape, amplitude and scanning speed) of a proton pencil beam, but can also determine the 2D dose distribution. In this paper, the absolute dose calibration of CROSS detector is studied by using the Markus detector in the Chang Gung Memorial Hospital. The equivalent water thickness of the CROSS detector is determined carefully to avoid the proton beam energy dependence in the absolute dose calibration. The calibration factor is then obtained, 1 ADC unit = 2.676 mGy at 20kHz sampling rate. The system error from reconstruction is determined to be less than 1%. This work makes the CROSS detector suitable for both machine QA and patient QA in the pencil beam treatment.

### P 099: Importance of Secondary Particles Produced in a Clam-Shell for Proton Therapy

#### S. MossahebiI^1^, M. O'Neil^1^, D. Strauss^1^, M. Mundis^1^, J. Eley^1^, A. Chhabra^1^, J. Snider^1^, K. Langen^1^, U. Langner^1^

##### ^1^University of Maryland School of Medicine, Radiation Oncology, Baltimore, USA

**Purpose**: Clam-shells are commonly being used for photon radiotherapy of para-aortic and abdomino-pelvic regions to protect testis in male patients. The purpose of this study was to investigate the effectiveness of utilizing a clam-shell to protect against primary radiation and secondary particle contamination produced when using proton beams.

**Methods**: A medium-size lead clam-shell was used at the Maryland Proton Treatment Center to evaluate the secondary particle generated by a proton beam. The clam-shell dimensions were 8.89cm outer-diameter and 6.35cm inner-diameter with 1.27cm wall-thickness. Secondary particles (fast and thermal neutron) were measured with photon-neutron dosimeters (Neutrak, Landauer). Two different setups were used delivering a 4×4×4cm3 field of 10Gy dose with dosimeters placed 2cm from the edge of the field (20% isodose-line): 1) shielded and 2) unshielded. Three readings were collected for each setup. We then compared the resulting neutron, deep and shallow dose equivalent.

**Results**: Measured dose equivalents with the dosimeters showed using the clamshell the fast and thermal neutron exposures were reduced to 10.39%±0.89% and 7.78%±2.52% of their original, unshielded values, respectively. The total deep and shallow dose equivalents for the shielded setup were 1.94%±1.88% and 1.99%±2.00% of the unshielded dose equivalent values, respectively.

**Conclusion**: Our preliminary findings suggest that the level of secondary particle contamination to the testis can be reduced using a clam-shell roughly to less than 10% of the unshielded level for proton therapy and it has a potential to be used in the treatment of para-aortic and abdomino-pelvic regions. We are planning to compare our measurements against Monte-Carlo simulations.

### P 100: Water Equivalent Ratios for Various Materials at Proton Energies from 10-500 MeV Using MCNP, FLUKA, and GEANT4 Monte Carlo Codes

#### H. Safigholi^1^

##### ^1^Department of Electronic Engineering-Shiraz Branch- Islamic Azad University, Shiraz, Fars, Iran Islamic Republic of

Dosimetry of proton beams is generally evaluated in liquid water, or alternatively in solid phantoms via water equivalent ratios (WER). WER is defined as the ratio of dose in liquid water to that in a phantom of certain material (Figure 1). Presently, WER is not available in the literature neither for a wide range of energies nor for variety of relevant materials. Thus, the goal of this study is to provide such data through Monte Carlo simulations. WER is calculated for 10-500 MeV energies for compact bone, adipose tissue, polymethyl methacrylate (PMMA), PTFE (teflon), graphite (C), aluminum (Al), copper (Cu), titanium (Ti), and gold (Au) using MCNPX.2.70, GEANT4, and FLUKA Monte Carlo (MC) codes. The MCNPX code was considered as the reference to which other codes were compared. The mean values of WER obtained through the MCNPX simulations for Au, Cu, Ti, Al, PTFE, graphite, PMMA, bone, and adipose tissue were 8.83, 5.40, 3.18, 2.04, 1.87, 1.52, 1.13, 1.71, and 0.96, respectively, for 10-500 MeV energy range. The maximum deviations of WER values between MCNPX and GEANT4 results were about 9.1% for adipose tissue at energies <20 MeV, whereas they were about 9.3% for MCNPX and FLUKA, for Al. Comparing the results to that in the literature, the greatest discrepancy was found to be about 9.5% for Au. Based on the materials evaluated, the PMMA remained the closest to water, for a non-tissue solid material, with an average WER of 1.13, for proton energy ranging 10-500 MeV.

### P 101: Monte Carlo Simulation of a Clinical Prototype 226MeV Protontherapy Beamline Using GATE Code

#### K. Schnuerle^1^, M. Vidal^1^, C. Peucelle^1^, A. Gerard^1^, P. Hofverberg^1^, J. Herault^1^

##### ^1^Institut Méditerranéen de Protonthérapie- Centre Antoine Lacassagne- 227 avenue de la Lanterne – Nice France, Medical Physics, Nice, France

The Centre Antoine Lacassagne (CAL) in Nice started patient treatments in September 2016 with the first Proteus®ONE from IBA equipped with a prototype supraconducting synchro-cyclotron (S2C2®) and a compact gantry which rotates from -35° to 188° and delivers Pencil Beam Scanning proton beams. Monte Carlo simulations of the beam line were performed with GATE, a Geant4 based Monte-Carlo code, using the TPSPencilBeam class implemented by Grevillot et al 2011. The beamline optical parameters, spot sizes in x and y and the corresponding convergence and emittance behavior as well as energy parameters were modelled and validated by experimental measurements.

Spot size measurements were performed for 26 different energies from 100 to 226 MeV at 6 different positions from the nozzle exit and downstream the isocenter with a 2D-scintillator (Lynx, IBA Dosimetry). The corresponding Bragg peaks were acquired in a water tank with a 12 cm diameter plane parallel ionization chamber (StingRay, IBA Dosimetry). The source parameters could reproduce the measured percentage depth-doses within +-2% when evaluating the range, mean point-to-point dose differences and Bragg peak size for all energies. A good agreement was also found between calculated and measured spot sizes in terms of sigma.

The GATE Monte-Carlo simulations of the first Proteus®ONE reproduces reasonably the experimental Bragg peaks and spots. Further validation tests including heterogeneous phantoms are currently ongoing. The simulation will be used for high dose pulsed beam absolute dose estimation and range evaluation.

### P 103: Using FBX and Fricke Dosimeters in Therapeutic Scanning Proton Beams

#### M. Troshina^1^, O. Golovanova^1^, E. Koryakina^1^, V. Potetnya^1^, R. Baikuzina^1^, S. Koryakin^1^, V. Saburov^1^, A. Lychagin^1^, S. Ulyanenko^1^

##### ^1^A. Tsyb Medical Radiological Research Center – branch of the National Medical Research Radiological Center of the Ministry of Health of the Russian Federation, Radiation Biophysics, Obninsk, Russian Federation

Quality assurance checks of proton beam scanning irradiation systems require a series of dosimetric measurements. Standard dosimetry is performed with ionization chambers (IC) according to the TRS398 recommendations. Measurements of dose distributions for large volumes take rather much time. To optimize measurements time in such cases we propose to use FBX and Fricke dosimetric systems, which allow to determine average doses in volumes of any size and shape.

The purpose of the study was to obtain calibration curves for chemical solutions irradiated with protons at the “Prometheus” synchrotron accelerator (“PROTOM”, Russia). Tubes with solutions were exposed in an aqueous phantom, absorbed doses were measured with a cylindrical IC (0.6 cm3). The dose range for the FBX dosimeter were 1-20 Gy, for Fricke – 10-50 Gy. Relative optical densities were measured with a SF-56 spectrophotometer in a standard way.

A FBX dosimeter response was linear up to proton absorbed doses of 10 Gy, at higher doses the calibration curve became less steeper. The calibration curve of the Fricke dosimeter was linear throughout the dose range studied. Comparison of protons doses measured by the IC and the Fricke dosimeter (with G(Fe3+) for 60Co γ-rays) allows to estimate the average LET of protons over volume using G(Fe3+)Fricke-LET relationship known, and subsequently to determine that for the FBX dosimeter. Calibration curves being available for a set of treatment plans and G(Fe3+)-LET relations being known, FBX and Fricke solutions can be used in the dosimetry of scanning proton beams for investigations in medicine and radiobiology.

### P 104: The Lifetime and Spatial Uniformity of Diamond Radiation Detectors for Proton Therapy

#### M. Zou^1^, T. Zhou^1^, J. Distel^2^, J. Bohon^3^, J. Smedley^4^, E. Muller^1^

##### *^1^Stony Brook University, Materials Science and Chemical Engineering, Stony Brook, USA*, *^2^Los Alamos National Laboratory, Space Science & Application Group, Los Alamos, USA*, *^3^Case Western Reserve University, Center for Proteomics and Bioinformatics, Cleveland, USA*, *^4^Brookhaven National Laboratory, Instrumentation Division, Upton, USA*

Currently, the most common dosimeters for particle therapy are gas filled ion chambers, which have issues with varied deviation correction according to radiation conditions and inconvenient installation due to their large-volume. Diamond radiation detectors, known for its high radiation hardness, high spatial and temporal resolution, is becoming more popular for clinical applications using proton beams. To ensure the reliability of dose measurements, tests for the lifetime and spatial uniformity of diamond detectors were performed. We have irradiated several diamond detectors with a 800MeV proton beam with high-flux (Los Alamos National laboratory) and the ion beam induced current (IBIC) was real-time monitored for lifetime measurement. Spatial uniformity was confirmed by response maps of irradiated diamond detectors using small non-destructive X-ray beams. During the irradiation, an exponential decay in detector response was observed, indicating the occurrence of damage from the proton radiation. This was explored further by X-ray beam induced current (XBIC) and SRIM simulation, which reveal that the carbon vacancies introduced from displacement effect act as hole trap centers in charge collection, and a model was built to evaluate the extent of radiation damage. Currently, we are adapting our diamond X-ray imaging technology to measuring proton flux, profiles and center-of-mass, with high spatial resolution (down to 15μm) and high temporal resolution (per pulse, ∼μs scale).

### P 105: Improving Efficiency in PBS Machine QA Using the IBA Sphinx

#### M. Blakey^1^, S. Petro^1^, J. Renegar^1^, S. Hedrick^1^, M. Artz^1^, N. Schreuder^1^

##### ^1^Provision, Proton Therapy, Knoxville, USA

**Purpose**: Our physics team examined how pencil beam scanning (PBS) machine QA can be simplified by using the IBA Sphinx phantom in combination with a scintillation detector. Before the Sphinx, a multi-device method was utilized for the dosimetric portion of the monthly QA. A scintillation detector was used to determine spot size and position, an ion chamber array for coincidence, an MLIC for range, and a parallel plate ion chamber for output. The Sphinx phantom allows for the measurement of each of these beam properties utilizing a single device.

**Method**: At our three-room proton therapy center we compared the time necessary to complete the monthly dosimetric QA, including data analysis and reporting, utilizing both the multi-device and Sphinx methods.

**Results**: For all three treatment rooms combined, the multi-device method took approximately 6 hours and 20 minutes to complete while the Sphinx method took only 2 hours. By utilizing the Sphinx there was a monthly time savings of approximately 4 hours and 20 minutes.

**Conclusions**: The Sphinx creates a simple, cohesive method that can significantly reduce the monthly QA time without compromising measurement quality. Our use of the Sphinx has not only greatly improved the efficiency of monthly dosimetric QA but has also allowed us additional time for patient care.

### P 106: A Modular Daily QA System for Line Scanning Proton Beam Therapy

#### J. Park^1^, K. Chung^2^, H. Pyo^2^

##### *^1^Samsung Medical Center, Dept. of Radiation Oncology, Seoul, Korea Republic of*, *^2^Samsung Medical Center- Sungkyunkwan University School of Medicine, Dept. of Radiation Oncology, Seoul, Korea Republic of*

As one of the most accurate radiation treatment modality, proton beam therapy requires a reliable daily Quality Assurance. At the same time, it is preferred to complete all required daily quality assurance items expeditiously. To fulfill these demands comprehensively, we developed an all-in-one type modular daily Quality Assurance system. We have designed a high density polyethylene device to be mounted on a commercial 2 dimensional ionization chamber array detector. The mountable device is modularized for output measurements, range verifications, and scan pattern measurements. A proton beam delivery plan has been created for the daily Quality Assurance based on the CT scanned image of the device. Each item of the daily Quality Assurance for line scanning nozzle has been tested with the developed system. Using the system, proton beam range verifications and gamma analysis of the scan pattern test have been combined into a measurement with a single beam delivery. The developed system will improve the efficiency of daily Quality Assurance with a flexibility of composing modular system in variations.

### P 107: Uses of System Dosimetry Records from the Delivery of a Pencil Beam Scanning Treatment

#### J. Cooley^1^, K. Huang^1^, D. Catanzano^1^, S. Nyamane^1^

##### ^1^Mevion Medical Systems, Advanced Development, Littleton, USA

In a pencil beam scanning proton therapy system, spots are treated with precise control of position, energy and dose. The proton MLC known as the Adaptive Aperture adds additional elements that must be controlled for each spot delivered. The ordering and repetition of spots can be used to make treatments robust and efficient. The Mevion scanning system utilizes the pulsed nature of the synchrocyclotron and measures the relevant beam parameters for every proton pulse delivered during a treatment. These are recorded in a dosimetry record that include charges and location of each pulse, the configuration of the energy selection system, and the exact locations of every leaf in the Adaptive Aperture proton MLC. As a result, the user has access to a large assortment of data after each treatment that provides a rich environment for post-processing analysis.

For example, the record could be used to develop service tools that track machine performance and predict when servicing might be required. Motion management could be optimized by performing timing analysis of the record to look at both total time delivery and volumetric rescanning possibilities (see Figure 1 for an example timing information from a record). For patient QA, charge and position data can be used to reconstruct the actual delivered treatment dose with Monte Carlo simulations.

We will present data extracted from the records of treatments delivered on the Mevion HYPERSCAN system showing important performance characteristics of the scanning system and provide examples of treatment dose reconstruction from onboard machine diagnostics.

### P 108: Utilization of Dedicated Planar Multi-Element Dosimeter for Patient-Specific Quality Assurance of Pencil-Beam-Scanning Protons

#### W.C. Hsi^1^, L. Coutinho^1^, S. Rana^1^, J. Bennouna^1^, A. Gutierrez^1^

##### ^1^Miami Cancer Institute, Medical Physics, Miami, USA

**Purpose**: To investigate measurement accuracy in our patient-specific quality assurance (PSQA) of pencil-beam-scanning protons due to setup technique.

**Methods**: Before our study, typical dosimetric measurements were performed using planar dosimeter and desired thickness of solid-water; this method might require lateral shifts to improve accuracy of planned dose comparisons and absolute dose measurements in water. In our study, the use of a water-scanning phantom (DigiPhant) and a planar 2D dosimetry array (MatriXX PT) (Fig. 1) provides accurate and efficient measurements. Setup procedure, including X-ray imaging and calibration/track of absolute dose, was established for our PSQA program. Measurements from >15 patients was performed at 5 different depths for each field with and without range shifter (RS). Analysis of each measurement was performed in myQA software program (IBA Dosimetry). We use Gamma index analysis of 3% - 3mm over 10% doses for score >90% and a difference of mean dose-point within 3% as a passing criteria versus calculated doses of pencil-beam algorithm.

**Results**: Examples of data analysis is shown in Fig 2. All measurements without RS pass with scores > 90%, with mostly scores of 100%. This shows the efficiency and accuracy of our PSQA program. However, indexes of measurements with RS can be less than 80% with 1-3% lower mean dose-point. Deviations with RS are strongly related to air-gap and beam range.

**Conclusion**: An efficient PSQA program was developed in this study. The root cause related to the fluence reduction/scatter modeling in planning for lower measured doses with RS will be investigated.

### P 109: Developing and Evaluating Fast Proton Range Verification Using a Step Wedge and 2D Scintillator

#### W. Deng^1^, W. Liu^1^, M. Bues^1^, J. Shen^1^

##### ^1^Mayo Clinic Arizona, Department of Radiation Oncology, Phoenix, USA

**Purpose**: To develop a fast method for proton range quality assurance (QA) using a step wedge and 2D scintillator, and to evaluate the accuracy, reproducibility, and robustness of this method.

**Methods**: An in-house customized step wedge and a 2D scintillator were developed to measure proton ranges (Figure 1a). Proton beams with homogenous fluence were delivered through the wedge, and 2D images with characteristic brightness pattern (Figure 1b) were captured by the scintillator. The locations of the brightest pixels were used to calculate proton ranges by a simple trigonometric method. The range measurements of 97 energies, comprising all clinically available synchrotron energies available at our facility, (ranges varying from 4cm to 32.6cm) were repeated 10 times to evaluate reproducibility. The robustness of the method to setup uncertainty was evaluated by measuring ranges with introduced 2mm setup uncertainties in x, y and z directions.

**Results**: Ranges of all 97 energies were measured in less than 10 minutes. The results were highly reproducible, and the standard deviation of 10 repeated measurements was only 0.037mm (Figure 2a). The method was also very robust to the setup uncertainty. With 2mm setup uncertainties, the measured range constancies were within 0.2mm (Figure 2b).

**Conclusion**: The new method of using step wedge and scintillator for proton range constancy check was efficient, highly reproducible, and robust. This method for proton range QA was feasible and appealing from a workflow point of view.

### P 110: Secondary Neutron Production by Proton and Carbon-Ion Therapeutic Beams

#### A. Di Fulvio^1^, M. Ferrarini^2^, M. Pullia^2^, M. Donetti^2^, S. Clarke^1^, F. Becchetti^3^, S. Pozzi^1^

##### *^1^University of Michigan, Nuclear Engineering and Radiological Sciences, Ann Arbor, USA*, *^2^Italian National Centre of Oncological Hadrontherapy, Technical Department, Pavia, Italy*, *^3^University of Michigan, Department of Physics, Ann Arbor, USA*

We characterized the fast-neutron yield and angular distribution produced by proton and carbon ion beams impinging on a RW3 phantom, using four organic scintillators. The detectors, based on EJ-309 liquid and stilbene, are sensitive to both fast neutrons and gamma rays. Pulse shape discrimination was used to classify neutron and gamma-ray interactions. The experiment was performed at the CNAO (National Centre of Oncological Hadrontherapy), Pavia, Italy, using 155- and 200-MeV proton and 292- and 388-MeV/u carbon-ion beams to irradiate a 30x30x15 cm3 phantom. Neutron production was measured for both pencil beam and spread-out Bragg peak (SOBP) irradiation mode. We performed Monte Carlo simulations of the irradiations. The simulated detector response agrees well with the measured one for proton irradiations, while it tends to underestimate the measured data for carbon-ion irradiations. The yield decreases as the angle between the detector axis and the beam axis increases, as expected from nuclear reaction kinematics (Fig.1 Scatter plot of the ratio between the tail and total integral of pulses vs light output for 292-MeV/u carbon-ion irradiation at an angle of (a) 15o, (b) 45o and (c) 90o. Counts are normalized by fluence and solid angle, in logarithm scale).

The intrinsic neutron detection efficiency of organic scintillators decreases at energies > 20 MeV. We shielded one of the stilbene detectors with lead, to enhance the detector response at high energies through the Pb (n,xn) reactions. We will discuss the spectrometric performance achieved using this approach and using deuterated scintillators, which are particularly suitable for neutron spectrometry.

### P 111: Validation of Quality Assurance Equipment for the New Prototype Supraconducting Synchro-Cyclotron (S2C2TM) PBS Proton Therapy System

#### A. Gerard^1^, M. Vidal^1^, C. Peucelle^1^, C. Delpech^1^, V. Floquet^2^, J. Herault^1^

##### *^1^Centre Antoine Lacassagne, Departement of Radiation Oncology, Nice, France*, *^2^GIE radiothérapie 08 – Clinique du Parc, Department of Radiation Oncology, Charleville Mézière, France*

The Centre Antoine-LACASSAGNE (CAL) hosts the latest PBS proton therapy system from IBA: ProteusOneTM equipped with the new supraconducting synchro-cyclotron S2C2TM. It has a frequency of 1kHz and delivers pulsed proton beam of dose rate between 2.65 μGy/pulse (96MeV) and 230 μGy/pulse (226MeV).

Due to these characteristics and high instantaneous dose rate, choice and use of dosimetric equipment is not trivial. The existing detectors were not necessarily suitable to this kind of beam and investigations had to be made in collaboration with the constructor.

For quality assurance (QA) CAL uses a 2D array Ionization Chamber “MatriXXONETM” and a 2D scintillator “LynxTM” combined with the new QA specific phantom “SphinxTM”. (IBA Dosimetry, Germany).

The MatriXXONETM was specially improved in order to overcome recombination issue in our beam. Measurements were performed in order to validate the prototype. Calibration, symmetry and uniformity were checked. The results were compared with the Lynx, which has already been valided in our beam, as well as with relative film measurements. General QA tests have been made with the Sphinx to see its compatibility with the system in particular with the specific repositioning system (X-ray 45°). The system was evaluated for several controls (positioning, range and spot size measurements) to see the gain in time, in resolution and the feasibility of controls.

The prototype MatriXXONETM gave satisfying results consequently it is now released and used in clinical routine for machine and patient QA. Quality assurance measurements with the Sphinx are in progress and the results will be reported.

### P 112: Automation of Verification Plan Preparation and Measurement Analysis for Proton Patient-Specific Quality Assurance

#### D. Hernandez Morales^1^, J. Shan^2^, W. Liu^1^, K.E. Augustine^1^, M. Bues^1^, M. Fatyga^1^, J. Shen^1^, J.E. Younkin^1^, J.B. Stoker^1^

##### *^1^Mayo Clinic Arizona, Radiation Oncology, Phoenix, USA*, *^2^Arizona State University, Biomedical Informatics, Tempe, USA*

**Purpose**: Physician-approved treatment plans undergo patient-specific quality assurance (PSQA) prior to delivery of the first fraction. For pencil beam scanning proton therapy, quality assurance is complex and time consuming, involving multiple measurements per field. We evaluated the PSQA workflow to identify routine steps that could be automated to improve efficiency.

**Methods**: We used the treatment planning system's (TPS) capability to support C# scripts to develop an Eclipse application programming interface (API) script and automate the preparation of the verification-phantom plan. The API script evaluated the gradient in the target volume of each verification field based on established criteria to identify adequate depth-dose profiles and depths for PSQA measurements. To improve measurement data analysis, a Python script was developed to automatically perform a 2D-3D γ-index analysis between the measurement plane and the TPS in-water volume for each acquired measurement. To quantify changes in efficiency, we evaluated a subset of patient plans for various disease sites with both our previous manual and automated methods.

**Results**: Automated verification plan preparation reduced the task time by more than 50%, decreasing the time from 5-20 minutes per field to 1-3 minutes per field. The γ-index analysis time reduction is more pronounced, being reduced by an order of magnitude for all disease sites. With these automations we observed an average overall PSQA time savings of 57% per patient plan.

**Conclusions**: Automating routine PSQA workflow tasks improves time efficiency, reduces user fatigue and focuses efforts on evaluation of key quality metrics.

### P 113: A System to Indirectly Measure X-Ray and Proton Dose during Patient Positioning and Treatment in Proton Therapy

#### P. Hofverberg^1^, A. Carnicer^1^, J. Hérault^1^

##### ^1^Centre Antoine Lacassagne, Department of radiation therapy, Nice, France

Quality control is particularly challenging in proton therapy since the beam is completely absorbed inside the target. The only means to verify the patient dose is through indirect observation of secondary radiation. It has also been proved difficult to measure the dose from X-ray examinations in a proton therapy setting since the high flux of secondary neutrons during treatments easily damage the detectors.

MEDICYC is a 65 MeV isochronous cyclotron used to treat eye tumours, situated at Centre Antoine Lacassagne (CAL) in Nice. A simple system has been developed for MEDICYC that exploits the secondary radiation emitted during X-ray and proton irradiation to independently estimate the delivered doses. An air ionization chamber, fixed on the wall in the treatment room, is used to continuously measure the ambient photon dose rate. The ionization chamber is integrated in the treatment delivery system and automatically identifies X-ray and proton irradiations and book-keeps the integrated ambient dose for each type. Proton dose is then calculated using a power-law fit between the ambient dose per proton dose (H/D) and the dose rate (D/MU), derived from all earlier treatments. X-ray entrance skin dose is calculated by exploiting the known measured linear correlation between the entrance air kerma and the ambient dose, and conversion factors derived from Monte-Carlo simulations. This system is routinely used at CAL for proton dose verification and has shown to provide an accuracy better than 5%. The accuracy of the X-ray dose estimation is currently being evaluated.

### P 114: Head-and-Neck Patient-Specific QA Experience in Pencil Beam Line-Scanning Nozzle

#### H.C. Huang^1,2^, S.W. Wu^1,3^, S.H. Lee^1^, C.Y. Yeh^1^

##### *^1^Chang Gung Memorial Hospital, Radiation Oncology, Taoyuan, Taiwan\r*, *^2^Chang Gung University, Graduate of Institute of Clinical Medical Sciences- College of Medicine, Taoyuan, Taiwan\r*, *^3^National Tsing Hua University, Department of Biomedical Engineering and Environmental Sciences, Hsinchu, Taiwan*\r

**Purpose**: This work is to describe our head-and-neck patient-specific QA experience in pencil beam line-scanning nozzle in Chang Gung Memorial Hospital (CGMH), Linkou in Taiwan.

**Material and Method**: Pencil beam line-scanning nozzle supplied by Sumitomo Heavy Industries, Inc. (SHI) was commissioned and implemented in clinical service in December 2016. A patient-specific QA program including MU calibration, depth dose and 2D dose distribution at several depths for each treatment field was developed to ensure the dosimetric accuracy. Gamma evaluation with 3%/3mm criteria was used for 2D dose distribution analysis. In addition, an in-house MATLAB program was developed to compare depth dose between TPS and measurements to evaluate not only point dose but also distal depth difference as an index of range deviation.

**Results**: We enrolled 115 head-and-neck patients with 481 portals in this work. The average MU difference between measurement and TPS is 3.08% ± 2.09% and the average gamma passing rate for 1184 measurements is 96.72% ± 3.79%. The distal depth difference also shows good agreement with TPS and the average is 1.2mm ± 0.7mm (absolute value).

**Conclusion**: We established a patient-specific QA program for pencil beam line-scanning technique and the results showed good agreement between delivery and TPS calculation.

### P 115: Development of a Daily Patient Beam Monitoring System in Pencil Beam Scanning Proton Therapy Using EBT3 Film

#### S. Jeong^1^, K. Chung^2^, M. Yoon^1^, J. Son^1^

##### *^1^Korea University, Bio-convergence engineering, Seoul, Korea Republic of*, *^2^Samsung Medical Center, Radiation Oncology, Seoul, Korea Republic of*

Before the first pencil beam scanning proton therapy of a patient, patient Quality Assurance (QA) is performed to confirm output and dose distribution to ensure that the planned beam is correctly exposed. However, it is important to check the daily beam because the confirmed beam from QA may not be accurately exposed due to human error or abnormal operation of nozzle, etc. Therefore, we have developed a method to confirm the daily dose distribution by attaching EBT3 film to the snout. We developed a device capable of attaching EBT3 film to snout so that distribution can be measured. When performing patient specific QA of 5 fields, a reference film, which has confirmed dose distribution from QA results, was obtained by measuring the dose distribution at snout using this device. We then performed the same measurement three times per field using the same method to confirm the consistency of the treatment beam. These were evaluated by comparing with the reference using the 3 % / 3 mm criteria gamma analysis method. The average of gamma passing rates were 98.7, 99.6, 99.4, 99.5, and 99.7%, respectively (Figure 1 – “Gamma comparison results of 5 fields”).

Through this study, we have developed a simple and accurate method for confirming whether the daily scanning proton treatment beam is exactly as planned by attaching an EBT3 film to the snout. It is expected that it would be able to monitor by fraction if the problem of beam delivering is occured by human error or nozzle problem, etc.

### P 116: Development of Method to Improve Efficiency of Range Verification in Routine QA of Proton Scanning Beam

#### S. Jeong^1^, K. Chung^2^, M. Yoon^1^, J. Son^1^

##### *^1^Korea University, Bio-convergence engineering, Seoul, Korea Republic of*, *^2^Samsung Medical Center, Radiation Oncology, Seoul, Korea Republic of*

In the range verification of the routine QA of the scanning proton beam, when one energy is exposed in one field, the efficiency of QA decreases in proportion to the number of energy to be analyzed. We developed a method of analyzing the range and distal fall-off using a multiple Bragg-peaks by exposing all energies in one field to solve this problem (Figure 1 - “Graph of multiple Bragg-peak”).

The energy composition of multiple Bragg-peaks is 40 MeV spacing from 70 MeV to 230 MeV. This was measured using a multi-layered ionization chamber. Five Bragg-peaks were extracted using home-made software which uses three fitting methods (Cubic spline, 8th order polynomial, Landau distribution), and their range and distal fall-off were analyzed. The results were compared with the range and distal fall-off of the single Bragg-peak. When using cubic spline, range was 0.1 mm longer and distal fall-off was 0.06 mm shorter. When fitting with the polynomial and Landau distribution, the range was 0.16, 0.06 mm longer, and the distal fall-off was 0.14 and 0.04 mm shorter, respectively (Table 1 - “Results of comparison between single and multiple Bragg-peak”).

Based on the above results, it was confirmed that the range and distal fall-off were easily calculated while maintaining the accuracy within 1 mm of the QA criteria using this method. At the same time, it was confirmed that the measurement efficiency of range QA can be increased.

### P 117: A Water Equivalent Range Calorimeter for Proton Beam Therapy Quality Assurance

#### S. Jolly^1^, R. Saakyan^1^, A. Basharina-Freshville^1^, L. Kelleter^1^, R. Amos^2^

##### *^1^University College London, Physics & Astronomy, London, United Kingdom*, *^2^University College London, Medical Physics, London, United Kingdom*

At clinical Proton Beam Therapy facilities, a range of essential daily Quality Assurance (QA) checks are carried out to verify a number of aspects of the clinical beam. Existing techniques for range QA are largely based around verifying the position of the Bragg Peak at a handful of energies using ionisation chambers to confirm the Water Equivalent Path Length (WEPL). These measurements can be time consuming to set up and carry out.

A detector is under development at University College London to improve the accuracy and reduce the measurement time for proton beam therapy WEPL QA to around 5 minutes. This detector uses layers of water equivalent plastic scintillator to intercept the beam, with the dose deposition calculated from the light output of each scintillator layer: this system has been adapted from scintillating calorimeter modules developed for the SuperNEMO high energy physics experiment. An overview of the conceptual design and development of the detector will be presented, along with the latest measurements with clinical proton beams.

### P 118: Correction of Prompt-gamma Distribution for Improving Accuracy of Beam Range Determination in Heterogeneous Medium

#### J.H. Park^1^, S.H. Kim^1^, Y. Ku^1^, H.S. Lee^1^, C.H. Kim^1^, D.H. Shin^2^, J.H. Jeong^3^, S.B. Lee^3^

##### *^1^Hanyang University, Nuclear engineering, Seoul, Korea Republic of*, *^2^Electronics and Telecommunications Research Institute, IT Convergence Technology Research Laboratory, Daejeon, Korea Republic of*, *^3^National Cancer Center, Proton Therapy Center, Goyang-si, Korea Republic of*

To fully utilize the advantage of proton therapy, it is required to measure the proton beam range in the patient accurately. One of the most promising methods for determining the proton beam range is to measure prompt gammas from the nuclear reactions of the proton beam in the patient. However, the prompt-gamma distribution depends on the characteristics of the medium (i.e., material and density), and it is difficult to determine the exact beam range in human body. To address this problem, in the present study, a correction method was developed and applied to the prompt-gamma distributions that were obtained by a multi-slit prompt-gamma camera for several configuration of heterogeneous media. The determined ranges, with and without using the correction method, were then compared with the true range. For the soft-tissue phantom with a bone-equivalent-material region inside, the beam ranges were determined within ∼2 mm of error for both before and after the correction, which means that this correction is not really necessary for brain tumors. For the soft-tissue phantom with the lung-equivalent-material region inside, on the other hand, the error was much larger, i.e. up to ∼20 mm, when we did not use the correction method. The error was then reduced down to ∼4 mm as we used the correction method. In addition, it was found that generally the beam range can be determined within 2 or 3 mm of error for a realistic phantom that represents the trunk/lung portion of a patient when we use the correction method.

### P 119: Improvement of the Treatment Plan Verification Procedure for Scanning Proton Beam at the Bronowice Cyclotron Center IFJ PAN in Kraków

#### M. Liszka^1^, A. Pędracka^1^, M. Kłodowska^1^, D. Krzempek^1^, B. Michalec^1^, R. Kopeć^1^, T. Kajdrowicz^1^, L. Stolarczyk^1^, D. Kabat^2^, T. Skóra^3^

##### *^1^The Henryk Niewodniczański Institute of Nuclear Physics Polish Academy of Sciences, The Bronowice Cyclotron Center CCB, Kraków, Poland*, *^2^Maria Skłodowska-Curie Institute - Oncology Center, Department of Medical Physics, Kraków, Poland*, *^3^Maria Skłodowska-Curie Institute - Oncology Center, Department of Oncology, Kraków*, *Poland*

Providing a proper dose distribution during radiotherapy session is essential for successful patient treatment. For this reason each treatment plan is verified dosimetrically. At the Bronowice Cyclotron Center the dosimetric verification of treatment plan used to be performed in two steps: point dose measurements with Markus ionization chamber (PTW Freiburg) and 2D dose spatial distribution measurements with the ionization chamber array MatrixxPT (IBA Dosimetry). This process is very time-consuming. To shorten the patient QA procedure we investigated the MatrixxPT detector cross-calibrated in terms of absorbed dose to water and cross-checked with the reference dose measured by Markus IC as the only tool for fast plan verification. We observed, that doses measured with MatrixxPT and Markus IC agree up to 2% for more than 93% cases. In the paper we present the detailed statistical analysis comparing doses measured with MatrixxPT vs Markus IC as well as our new, almost two times faster patient QA procedure.

### P 121: Implementation of Average Intensity of Isotope Activity of Offline PET/CT in Estimating Accuracy of Delivered Dose with Scanning Particle Beams

#### Y. Li^1^, Y. Gong^1^, P. Li^2^, S. Fu^2^

##### *^1^Shanghai Proton and Heavy Ion Center, Medical Physics, Shanghai, China*, *^2^Shanghai Proton and Heavy Ion Center, Radiation Oncology, Shanghai, China*

**Purpose**: To develop a novel method to monitor dose delivered to patient treated with scanning carbon ion beams, using average intensity of isotope activity (AIIA) of offline PET/CT.

**Methods and Materials**: The AIIA and dose percentage were calculated and analyzed in ten prostate cancer patients with carbon ion therapy. The signals of Offline PET/CT was fused and matched manually with planning CT image by Landmark Registration. From the matched signal, a point-to-point comparison between offline PET/CT signals and dose of planning CT on a beam direction line was obtained (Fig.1). Several isodose lines at certain space interval from 5% to 95% were used to calculate AIIA and normalized. The relationship between the delivery dose areas and the AIIA were acquired.

**Results**: There were unilateral field for 2 patients and contralateral field for 8 patients. The correlation factors of the two kinds of irradiation for AIIA were 1E6 and 8E5 Bq/ml, respectively. The curve (Fig.2) showed that the AIIA was matched with and correlated to delivery dose areas. Also, the gradient of AIIA was sharp when the dose percentage was over 60%. The data of other group are being analyzed. More calculation, verification and static of the estimating accuracy is ongoing.

**Conclusion**: The AIIA in the method affected by registration method, biological washout time, anatomical motion, etc. However after the impacted factors were scaled, the method can predict accuracy of delivered dose with AIIA. The static of estimating accuracy of delivered dose with the method need to be further investigated.

### P 122: Design of a QA Method to Characterize Submillimeter-Sized PBS Beam Properties Using a Two-Dimensional Ionization Chamber Array

#### Y. Lin^1^, H. Bentefour^2^, J. Flanz^3^, H. Kooy^3^, F.F. Yin^1^, B. Clasie^3^

##### *^1^Duke University Medical Center, Radiation Oncology, Durham, USA*, *^2^IBA- Ltd, Proton Therapy, Louvain-la-Neuve, Belgium*, *^3^Massachusetts General Hospital and Harvard Medical School, Radiation Oncology, Boston, USA*

The aim of this study is to investigate whether a 2D ionization chamber array such as the MatriXX detector can be used to characterize submillimeter-sized PBS beam properties precisely. The motivation is to use standard equipment, which may have pixel spacing coarser than the pencil beam size, and simplify QA workflow.

The MatriXX pixels are cylindrical in shape with 4.5 mm diameter and are spaced 7.6 mm from center to center. Two major effects limit the ability of using the MatriXX to measure the spot position and width precisely. The first effect is that too few pixels sample the Gaussian shaped pencil beam profile and the second effect is volume averaging of the Gaussian profile over the pixel sensitive volumes. We designed a line test pattern that overcomes both limitations and hence enables the use of the MatriXX to characterize sub-millimeter-sized PBS beam properties (see Figure 1). This method is designed to increase the number of sampling data points.

With the techniques, the MatriXX detector can be used to characterize the position and width of sub-millimeter (σ = 0.7 mm) sized pencil beams with error better than 3% relative to σ. If one were to not use a line test pattern, then the error in extracting the σ of the beam is reduced to 10% for σ = 3.6 mmn, see Figure 2.

This work helps to simplify periodic QA in proton therapy because more routinely used ionization chamber arrays can be used to characterize narrow pencil beam properties.

### P 125: A Novel Procedure for Generating, Visualizing and Comparing Dose from Commercial Treatment Planning System and Monte Carlo Simulations

#### L. Muller^1^, M. Prusator^1^, S. Ahmad^1^, Y. Chen^1^

##### ^1^University of Oklahoma HSC, Radiation Oncology, Oklahoma City, USA

Proton treatment planning systems (TPS) are used to create treatment plans, and Monte Carlo (MC) simulations are often used to validate those results. For a double scattering proton system, each unique field in a treatment plan has 2 unique components: a compensator and aperture. MC simulations of the dose delivered in treatment plans require these components be included in the simulation geometry. We have developed a workflow using a combination of Matlab and Fusion360 to easily generate importable models of these components from the TPS DICOM plan exports. Compensator models are generated in STL format with Matlab, and aperture models are generated in STL format with Fusion 360 using coordinates extracted and scaled from the DICOM plan file with Matlab.

Additionally, many MC toolkits do not have the built in capability to perform dose visualization, analysis and comparison. We developed a workflow to achieve these tasks using a combination of Matlab and the open source software 3D Slicer. Specific tools for dose normalization, filtering, format conversion, 2D and 3D gamma analysis, and DVH analysis are developed. These tools enable the easy and efficient verification and comparison of dose distributions calculated with treatment planning software and Monte Carlo simulations. The TPS used was Varian Eclipse and the MC toolkits used were Geant4 and TOPAS.

### P 127: Clinical Implementation of MIMI/HexaCheck Phantom to Assess Accuracy and Stability of LEONI 6D-Robotic Couch

#### S. Rana^1^, W. Hsi^1^, J. Bennouna^1^, L. Coutinho^1^, V. Chirinos^1^, A. Gutierrez^1^

##### ^1^Miami Cancer Institute, Radiation Oncology, Miami, USA

**Purpose**: The purpose of this study is to present the clinical implementation of MIMI/HexaCheck phantom for daily 6-D IGRT QA of a proton system and to report on the stability of 6-D positioning with LEONI couch.

**Methods**: A treatment plan with kV/kV and CBCT setup fields was generated in RayStation using CT images of MIMI phantom. The plan isocenter was defined at the center of MIMI. The MIMI was placed in the HexaCheck and indexed to the couch top. The MIMI was aligned to the known lateral (x), longitudinal (y) and vertical (z) offset positions. Rotations of 2.5^0^ were then applied to pitch, roll and yaw. A CBCT was then acquired and the correction vector (CV) was recorded. The CV was applied and kV/kV imaging was used to verify final positioning. Differences (Δ) between CV and known offsets were calculated.

**Results**: Analysis of daily QA measurements (n=35) were categorized into: Group I: Δ<±1mm or ±1^0^; Group II: Δ=±1mm–±1.5mm or 1^0^–±1.5^0^; Group III: Δ>±1.5mm or ±1.5^0^ and <±2.0mm or ±2^0^. The Δx, Δy, and Δz were found to be predominantly in Group I (n=32, n=31, and n=30, respectively) compared to Group II (n=3, n=3, and n=3, respectively) and Group III (n=0, n=1, and n=2, respectively). For rotational shifts, Δ was <1^0^ for all measurements except for roll in one case (1.1^0^).

**Conclusion**: The use of MIMI/HexaCheck served as an accurate and efficient tool to perform daily, 6-D IGRT QA of the IBA adaPT Insight software and LEONI robotic couch.

### P 128: Clinical Implementation and Initial Results of a Novel Commercial Device (Sphinx and Lynx) for Dosimetric PBS Daily QA

#### S. Rana^1^, L. Coutinho^1^, W. Hsi^1^, J. Bennouna^1^, V. Chirinos^1^, A. Gutierrez^1^

##### ^1^Miami Cancer Institute, Radiation Oncology, Miami, USA

**Purpose**: A robust daily quality assurance (QA) program is required to gain confidence in the performance of a proton therapy system. In this study, we focus on the development of the dosimetric component of our daily QA program using a novel commercially-available device (Sphinx) and quantify the stability of certain beam parameters evaluated with it.

**Methods**: Daily QA plans with spot map of 4 different energies were delivered on Proteus®PLUS proton therapy system through ARIA. Dosimetry measurements were performed using a single couch top setup with the Sphinx, Lynx, and PPC05 chamber. Specifically, dosimetric tests were divided into:(1) spot position, size, and skewness, (2) distal and proximal range, width, and distal-fall-off (DFO),(3)radiation and imaging co-incidence, and(4)dose output. For evaluation and analysis, myQA software was utilized for tests #1-3 and an in-house excel sheet was used for test #4.

**Results**: Based on 35 daily QA measurements, the average time for setup, acquisition, and data analysis was 20±5 minutes. The Δavg herein means difference between average measured and baselined values. Results of spot parameters (test #1) were within tolerances (position=±1mm, sigma=±10%,skewness=0.5mm) with ΔavgPosX=-0.10mm, ΔavgPosY=0.25mm,ΔavgSigmaX=-0.02%, and ΔavgSigmaY=2.17% and ΔavgSkewness<0.1mm. Results of range, width, and DFO (test #2) were all Δavg<0.1mm(tolerances=±1.5mm). For coincidence (test #3), ΔConX(range,-0.80mm–1.3mm) and ΔConY(range,-0.70mm–0.90mm) were within tolerance(±1.5mm). The Δoutput (test #4) ranged from -0.52%–1.47% (tolerance=±2%).

**Conclusion**: The use of the Sphinx in conjunction with the Lynx and a parallel-plate ion chamber was shown to provide a robust and efficient method of evaluating PBS beam parameters needed for the dosimetric component of a comprehensive daily QA program.

### P 129: A Simple Implementation of Log File QA for Pencil Beam Scanning Proton Treatment

#### J. Renegar^1^, M. Blakey^1^, S. Hedrick^1^, M. Artz^1^, B. Robison^1^, S. Petro^1^, N. Schreuder^1^

##### ^1^Provision Proton Therapy Center, Medical Physics, Knoxville, USA

**Purpose**: Currently in pencil beam scanning proton therapy, patient QA is performed for all treatment fields. This evaluates machine performance and ensures the correct field is delivered. Given a thorough machine QA program, patient QA can focus only on confirming delivery of the correct field. This confirmation can be accomplished through analysis of log files.

**Method**: Log files are generated with varying degrees of detail, however, to confirm the correct field has been delivered, the simplest proton beam log file should contain layer energies, spot positions, and monitor units. The IBA proton system creates such log files in the form of RTRECORD DICOM files, which mirror the structure of the RTPLAN file sent from the OIS. We have developed software to analyze log files and compare the calculated delivered dose to the planned dose. Using the commissioned spot sizes in air, an in-air dose plane is reconstructed from the spot information contained in the log file. This reconstructed plane is then compared to the TPS-calculated in-air plane using conventional analysis methods, such as the gamma index.

**Results**: We have evaluated delivered fields both with and without intentional errors. For correct fields, fully delivered, we achieve gamma pass rates of at least 90% (2%, 2mm) between the reconstructed plane and the TPS calculated plane. An incorrect field or partial delivery show significantly lower pass rates.

**Conclusions**: Analysis of machine generated RTRECORD DICOM files is an effective way to confirm the correct field delivery for PBS proton therapy.

### P 130: Evaluation of TOPAS Monte Carlo Simulations as a Second Dose Calculation in Pencil Beam Scanning (PBS) Proton Therapy

#### J. Shin^1^, K.W. Jee^1^, B. Clasie^1^, N. Depauw^1^, J. Daartz^1^, E. Batin^1^, T. Madden^1^, J. Schuemann^1^, H. Paganetti^1^, H. Kooy^1^

##### ^1^Massachusetts General Hospital, Radiation Oncology, Boston, USA

Two planar distributions in phantom with field-specific devices are measured at proximal depth (D1) and tumor depth (D2) to validate each PBS field for treatment. This work aims to reduce the number of QA measurements through the use of a Monte Carlo (MC) second check.

TOPAS MC beam model was commissioned to match measurement within 1% for range differences, 2.0% for absolute dose, and 1 mm for in-air spot size around 20 cm from isocenter. A total of 883 QA fields were measured at two depths and compared to the analytical dose algorithm (AA) and MC using ϒ(3mm,3%). Then, MC was evaluated as an alternative reference to AA.

The averaged ϒ-index pass-rates of MC to measurement data (M2D) were 98.81% for D1 and 99.36% for D2 while those of the AA to data (A2D) were 97.86% and 98.63% for D1 and D2, respectively. AA had more outlier fields below 90% pass-rates than MC for both D1 and D2 (Figure1).

Especially 13% of AA and 5% of MC fields at depths less than 2 cm had pass-rates below 90%. With MC as an alternative reference to classify high and low pass-rate fields, the 89.5% and 96.7% of accuracy on predicting D1 and D2 fields were expected. The D1 prediction accuracy was increased to 92.9% by excluding the measurement depths below 2cm (Figure 2). This study confirmed the accuracy of MC experimentally and demonstrated the the use of MC as a second dose calculation to reduce number of measurements.

### P 131: The Mechanical Accuracy of an Iso-Centric Rotating Chair for Treating Patients in Seated Positions

#### J. Sun^1^, W. Hsi^1^, Y. Sheng^1^, F. Yang^2^, X. Zhang^2^, X. Wu^1^

##### *^1^Shanghai Proton and Heavy Ion Center, medical physics, Shanghai, China*, *^2^Sichunan University, College of physical science and technology, Chengdu*, *China*

**Purpose**: To test the mechanical accuracy of an iso-centric rotating chair designed for providing treatment from various beam angles for patients in seated positions.

**Methods and Materials**: The mechanical movement of the chair is achieved by two digital-control motion platforms: 360 rotating platform and 6 degree-of-freedom (6DoF) platform, and a manual XYZ translational platform. The 360 rotating platform performs the rotation around z-axis, perpendicular to the floor plane. The 6DoF platform carries out the corrections following the patient alignment using the X-ray imaging system. Software was developed to manipulate the movement of these two platforms. An optical tracking system was installed to track the movement of the chair. Several motion sequences were used for the test:

1. For the rotating platform, the test includes multiple clockwise and counterclockwise angels with various weights on the chair and with the 6DoF platform set to multiple pitch angles;
2. For the 6DoF platform, the test includes the three translation directions, independent or combined with rotations about three axes.

The deviation in each specific movement was obtained and analyzed.

**Results**: For the 360 rotating platform, while the clockwise rotation demonstrated agreement within 0.01between the measured and the input values, a maximum of 0.1 deviation was observed with counterclockwise rotation. For the movement of 6DoF platform, the deviation between the measured and the input values are all within 1.0 mm in translations and 1.0° in rotations.

**Conclusions**: The positioning accuracy and reproducibility of the rotating-chair is acceptable for clinical application.

### P 132: Mailed Dosimetry Intercomparison of 15 Ocular Proton Therapy Facilities

#### J. Swakoń^1^, C. Bloch^2^, M. Ciocca^3^, P. Cirrone^4^, I. Daftari^5^, C. De Angelis^6^, M. De Saint-Hubert^7^, F. Goudjil^8^, J. Heufelder^9^, J. Hérault^10^, C. Hoehr^11^, T. Horwacik^12^, J. Hrbacek^13^, A. Kacperek^14^, Ž. Knežević^15^, D. Krzempek^12^, J. Kunst^1^, M. Mamalui^16^, B. Michalec^12^, G. Mierzwińska^12^, P. Olko^1^, M. Pankuch^17^, L. Raffaele^4^, B. Reniers^18^, R. Slopsema^16^, A. Toboła^1^, A.V. Trofimov^19^, A. Wochnik^1^, R. Zhu^20^

##### *^1^Institute of Nuclear Physics Polish Academy of Sciences, IFJ PAN Proton Radiotherapy Group, Krakow, Poland*, *^2^University of Washington, SCCA Proton Center, Seattle- Washington, USA*, *3Fondazione CNAO, Unità di Fisica Medica, Pavia, Italy*, *^4^INFN-LNS, Centro di AdroTerapia e Applicazioni Nucleari Avanzate, Catania, Italy*, *^5^University of California, Department of Radiation oncology, San Francisco, USA*, *^6^Istituto Superiore di Sanità, Core Facilities, Rome, Italy*, *^7^SCK CEN Belgian Nuclear Research Centre, Unit Research in Dosimetric Applications, Mol, Belgium*, *^8^Institut Curie, Centre de protonthérapie, Orsay, France*, *^9^Charité - Universitätsmedizin Berlin, Helmholtz-Zentrum Berlin für Materialien und Energie- Protonentherapie, Berlin, Germany*, *^10^Centre Antoine Lacassagne, Institut Méditerranéen De Protonthérapie – IMPT, Nice, France*, *^11^TRIUMF Canada's particle accelerator centre, Proton Therapy, Vancouver, Canada*, *^12^Institute of Nuclear Physics Polish Academy of Sciences, Cyclotron Centre Bronowice, Krakow, Poland*, *^13^Paul Scherrer Institut, Center for Proton Therapy, Villigen, Switzerland*, *^14^The National Eye Proton Therapy Centre, The Clatterbridge Cancer Centre NHS FT, Bebington, United Kingdom*, *^15^Ruđer Bošković Institute, Radiation Chemistry and Dosimetry Laboratory, Zagreb, Croatia*, *^16^University of Florida, Health Proton Therapy Institute, Jacksonville, USA*, *^17^Northwestern Medicine Chicago Proton Center, Medical Physics, Warrenville, USA*, *^18^University Hasselt (UH), Research Group NuTeC, Diepenbeek, Belgium*, *^19^Massachusetts General Hospital, Burr Proton Therapy Center, Boston, USA*, *^20^The University of Texas, MD Anderson Cancer Centre, Houston, USA*

A mailed dosimetry intercomparison of fifteen European and North American proton ocular therapy centres was organized by the Institute of Nuclear Physics, Polish Academy of Sciences, Krakow, Poland (IFJ PAN), together with Working Group 9 of EURADOS and PTCOG Optic Subcommittee.

Its aim was to evaluate consistency in delivering therapeutic dose to patients in each facility and to test future mailed passive detector dosimetry audit procedures. Detectors and phantoms were prepared at IFJ PAN, Belgian Nuclear Research Centre SCK CEN, University Hasselt (UH), Croatian Ruđer Bošković Institute (RBI), and Italian Istituto Superiore di Sanità (ISS). Detectors were calibrated at the IFJ PAN eye treatment facility.

Participants received five (#1-5) PMMA phantoms (4 × 4 × 4 cm3), each containing different sets of detectors supplied by collaborating Partners: Phantom#1 contained nine LiF:Mg,Ti (MTS-N) TL pellet detectors and one 2D LiF:Mg,Cu,P TL foil, from IFJ PAN; Phantom#2 - nine alanine detectors and Gafchromic film, from IFJ PAN; Phantom#3 - nine alanine detectors, from SCK - UH; Phantom#4 - nine alanine detectors, from ISS; Phantom#5 - three RPL detectors, from RBI. Requested irradiation conditions were: range in water-26 mm (90%), SOBP modulation-26 mm, collimator aperture-25 mm, prescribed dose-15 Gy at isocentre. Phantoms with detectors were sent out early November 2017, irradiated November-December 2017, and returned to IFJ PAN January 2018. Final results are expected in 2018.

Acknowledgement: Intercomparison organizers wish to thank all Participants for their excellent collaboration in receiving, irradiating and returning the phantom materials.

### P 133: Experimental Study of Proton Dose Perturbations Due to Self-Expanding Metallic Stents

#### E. Tryggestad^1^, A. Storm^2^, N. Remmes^1^, C. Beltran^1^, M. Haddock^1^, J. Kruse^1^, M. Topazian^2^, C. Hallemeier^1^

##### *^1^Mayo Clinic, Radiation Oncology, Rochester, USA*, *^2^Mayo Clinic, Internal Medicine, Rochester, USA*

**Purpose**: Biliary and luminal self-expanding metallic stents (SEMS) are integral to routine management of patients suffering from Gastro-Intestinal (GI) malignancies. However, SEMS can deleteriously impact proton dose distributions. We sought to characterize this effect for a set of SEMS relevant to our multidisciplinary practice.

**Methods**: A “worst case” experiment was designed employing a uniform field of monoenergetic, spot-scanned protons (119.7 MeV) delivered through a 45mm range shifter. 14 SEMS (varying manufacturers, models, diameters) were individually placed in a water bath proximal to a “sandwich” of EBT3 films (Ashland) interleaved with sheets of paper (Fig1). The water-equivalent spacing of films was ∼1.1mm, determined using a multi-layer ion chamber. The first film was located ∼2.6 mm downstream from the water bath (stent), mimicking a tumor location. The water depth was adjusted to stop protons in the sandwich, the distal-most film located beyond the practical range. Films were digitized and dose-converted using FilmQA Pro (Ashland). Peripheral sampling regions were identified on each film for determining background dose. Dose difference from background was calculated and a rectangular region (covering the stent) identified for quantifying mean dose difference.

**Results**: Proximal films demonstrated small dose enhancements behind the stents, whereas distal films showed dose deficit/shadowing with increasing significance with depth (mean deficits ≤30%) (Fig2). Stent diameter was not predictive of dose difference, whereas similarity was generally observed within a particular stent model (i.e., mesh material or mesh wire density) excepting one luminal outlier.

**Conclusions**: Proton dose perturbations due to SEMS may be clinically significant; follow-up experimentation using spread-out-Bragg Peaks is warranted.

### P 135: Patient Related QA in PTC Czech Prague

#### L. Zámečník^1^, M. Navratil^1^, K. Badraoui-Cuprova^1^, M. Andrlik^1^, S. Stastna^1^, J. Vilimovsky^1^, V. Vondracek^1^

##### ^1^Proton Therapy Centre Czech, Medical Physics, Praha, Czechia

Intensity Modulated Proton Therapy (IMPT) is facilitated by thousands of individually weighted spots. The dose distribution from a field may be inhomogeneous with dose gradients in planes perpendicular to and in the direction of the beam axis, especially using more fields technique. Therefore, the correct delivery of the dose requires measurement for patient related QA.

In PTC, we measure each field in multiple depth for the pencil beam scanning (PBS). For the first two years we verified the relative dose distributions using the detector array and the absolute dose at the points with the plan-parallel ionization chamber. Since February 2015, we've changed our methodology and verified absolute dose distributions in planes using the DigiPhant (IBA Dosimetry, Germany), with MatriXx calibrated in a proton beam.

Detector array measurements are evaluated by gamma analysis with DTA = 3mm and DD = 3%. The pass criteria on gamma<1.0 is 95% and gamma<1.5 is 98%. From February 2015 to January 2018, according to the new methodology, we verified 3,289 clinical plans for 2,217 patients. The total number of measured maps is more than 21,000 for 6,700 fields.

More than 95% of measurements meet the criteria. A total of 4.9% of maps failed of the criteria which we evaluate and analyze. Reasons for failing are predominantly steep dose gradient and the distal fall-off regions, where detectors distance not sufficient.

### P 136: Automatic Measurement of Air Gap for Proton Therapy Using Orthogonal X-ray Imaging with Radiopaque Wires

#### *Y. Zhang^1^, P. Ramesh^1^, W. Song^2^, H. Cao^3^, Y. Zhao^4^, K. Nie^1^, N. Y*ue^1^

##### *^1^Rutgers University, Radiation Oncology, New Brunswick, USA*, *^2^Jiangsu Province Hospital of TCM, Radiation Oncology, Nanjing, China*, *^3^Renji Hospital affiliated to School of Medicine- Shanghai Jiao Tong University, Radiation Oncology, Shanghai, China*, *^4^Sichuan Cancer Center- School of Medicine- University of Electronic Science and Technology of China, Radiation Oncology, Chengdu, China*

**Purpose**: The objective of this study was to develop a technique to determine the air gap between the end of the compensator and the patient in proton radiotherapy.

**Methods**: Orthogonal image-based reconstruction was used to determine the air gap between the patient and the compensator in proton radiotherapy. To identify the patient surface on the orthogonal images, a radiopaque wire was placed on the surface as surrogate. To validate this method, a Rando head phantom was scanned and five plans were generated on a Mevion S250 Proton machine with various air gaps in Varian Eclipse TPS. When setting up the phantom, a solder wire was placed on the phantom closest to the compensator with prior knowledge of the beam geometry. After the phantom positioning was verified using orthogonal kV imaging, the last image pair were used to segment the wire and the wire in-room coordinates were reconstructed using a back-projection algorithm. The air gaps were calculated by finding the minimum distance between the reconstructed wire and the compensator.

**Results**: The derived air gaps were found to be within 1.1mm difference with the planned values (air gaps of 85.0/100.0/150.0/180.0/200.0mm). There was strong agreement between the calculated and the planned values.

**Conclusions**: An image-based automatic method has been developed to conveniently determine the air gap, directly using the orthogonal images for patient positioning without additional imaging dose. This method provides an objective, accurate and efficient way to confirm the target depth at treatment to ensure desired target coverage and normal tissue sparing.

### P 137: Proton PBS Beam Range Mixing to Reduce Lateral and Distal Penumbrae for Shallow Targets

#### J. Alshaikhi^1,2,3^, D. D'Souza^2^, U. Langner^4^, C.G. Ainsley^5^, G. Royle^1^, R.A. Amos^1,2^

##### *^1^University College London, Department of Medical Physics and Biomedical Engineering, London, United Kingdom*, *^2^University College London Hospitals NHS, Department of Radiotherapy Physics, London, United Kingdom*, *^3^Saudi Particles Therapy Center, Proton Therapy, Riyadh, Saudi Arabia*, *^4^University of Maryland School of Medicine, Maryland Proton Treatment Center, Maryland, USA*, *^5^University of Pennsylvania, Roberts Proton Therapy Center, Philadelphia, USA*

**Purpose/Objective**: Proton pencil beam scanning (PBS) systems deliver beam energies varying between maximum and minimum values. Delivering shallow beams with ranges less than those corresponding to the minimum energy requires inserting a range shifter (RS) into the beam path. Consequence of using a RS is increased spot sizes, reducing dose conformity. The aim of this study is to evaluate a range mixing technique to mitigate this effect.

**Materials and Methods**: Calculations performed using Eclipse v13.5, commissioned with beam data from a Varian ProBeam system (minimum energy 70 MeV/range 4.1 cm Weq), and an IBA system (minimum energy 100 MeV/range 7.7 cm Weq). A homogeneous (40x40x40)cm3 phantom containing multiple (10x10x10)cm3 targets was created in silico. Targets were positioned at different depths within the phantom; the shallowest surface positioned at 0.5, 1, 2, 3, and 4 cm depth for Varian beam calculations, and additionally at 5, 6, and 7 cm depth for IBA beam calculations. Two plans were compared for each target using each beam dataset; one plan covering the target with a single spot-scanning portal and RS throughout, the other covering the target using one spot-scanning portal without the RS for a distal portion, and a second spot-scanning portal with the RS for a proximal portion of the target.

**Results**: Presented in Figure 1, and Table 1.

**Conclusions**: Range mixing improved lateral and distal penumbrae for shallow targets in homogeneous media. Further investigation required to evaluate dosimetrically in patient geometry. Treatment time may be a consideration for clinical implementation without automation.

### P 138: Fragmentation Measurements with the FOOT Spectrometer

#### P. Cerello^1^

##### ^1^INFN, Sezione di Torino, Torino, Italy

Beam-induced tissue fragmentation is still an open issue in particle therapy.

Nuclear interactions in the beam entrance channel produce fragments with high Relative Biological Efficiency (RBE), whose contribution to the unwanted damage is hard to evaluate. Accurate data on fragmentation cross sections are needed to improve the RBE models and, ultimately, the clinical treatment plans.

The FOOT (FragmentatiOn Of Target) experiment, now under construction, was designed to identify the fragments produced in the human body during particle therapy and measure their production cross sections, taking advantage of an inverse-kinematic approach: the boost will make the fragments cross the detector, that will measure their mass and momentum.

The detector layout (fig. 1) includes a start counter that provides the trigger and the start of the Time-Of-Flight (TOF) measurement when crossed by a primary particle. Three sets of silicon detectors and two magnets will implement the tracking of fragments generated in the target, separating them, measuring their momentum and matching them with the last two detector elements that provide the particle identification: a thin plastic scintillator and a BGO calorimeter for the ΔE-TOF and the kinetic energy measurement.

In dedicated runs, an emulsion spectrometer will be inserted after the target to measure the production of low Z fragments.

The FOOT experiment will measure differential cross sections for ions beams on different targets (e.g., C, C2H2), with about 5% uncertainty (Fig. 1: Sketch of the FOOT layout. Top: 3D view of the electronic setup. Bottom: 2D view of the target region with the emulsion setup).

### P 139: The Comprehensive Comparison of PPTV-Based IMPT Optimization versus Robust Optimization

#### T.H. Wu^1^, S.H. Lee^1^, C.Y. Yeh^1^, C.Y. Chou^1^

##### *^1^Chang Gung Memorial Hospital- Linkou Branch, Department of Radiation Oncology, Taoyuan, Taiwan\*r

**Purpose**: Robust optimization (RO) leads to IMPT plans that are less sensitive to uncertainties and superior in terms of OARs sparing, target dose coverage, and homogeneity compared to proton planning target volume (PPTV)-based optimized plans. We illustrate comparison results between PPTV-based and RO.

**Methods and Materials**: For traditional IMPT optimization, new planning target volume called PPTV was created. All the uncertainties were accounted by expanding CTV to PPTV via approximate margins. For robust IMPT optimization, field-specific target was created for each individual field to define maximum pencil beam scanning area. During robust optimization, CTV and OARs can be optimized with accounting for CT uncertainties (3.5%) and setup error. H&N, Chest, and Abdomen plans were enrolled in this study and were analyzed by robustness evaluation. Setup error settings were 2mm, 5mm, and 5mm for H&N, Chest, and Abdomen sites respectively.

**Results**: Compared with conventional PPTV-based and RO led to a smaller bandwidth for the targets in the face of uncertainties; CTV bandwidth. At the same time, the RO method irradiated OARs less maximum dose to brainstem and spinal cord in H&N cases. Under the worst-case scenario, the dose in the target remained widely homogeneous and the sparing of the critical organ was preserved.

**Conclusions**: From these comprehensive comparisons, the data show the superiority of RO is not due simply to its inclusion of margins for OARs, but that this is due mainly to the ability of RO to compensate for perturbations in dose distributions within target volumes and normal tissues caused by uncertainties.

### P 140: Practical SBPT Planning Guideline in Lung Cancer for Pencil Beam Scanning Proton Therapy

#### K. Chung^1^, A. Choi^2^, W. Lee^2^, J.M. Noh^1^, H. Pyo^1^, D. Oh^1^, Y.C. Ahn^1^

##### *^1^Samsung Medical Center- Sungkyunkwan University School of Medicine, Dept. of Radiation Oncology, Seoul, Korea Republic of*, *^2^Samsung Medical Center, Dept. of Radiation Oncology, Seoul, Korea Republic of*

To provide a practical guideline in stereotactic body proton therapy of lung cancer for pencil beam scanning (PBS), 12 SBPT cases have been studied for the dosimetric evaluations and analysis. The dose of clinical plans has been optimized and calculated using clinical pencil beam dose calculation (cPBDC) algorithm (RayStation 5.2). The same plans have been re-calculated using Monte Carlo dose calculation (MCDC) algorithm introduced in RayStation 6.2. For each dose calculation, perturbed doses have been calculated by varying density (+/- 5%) and isocenter shift (up to +/- 7 mm). The plan quality has been evaluated with homogeneity index (HI) and conformity index (CI) of target volumes and DVH parameters of organ-at-risk (OAR) have been compared.

The cPBDC algorithm has over-estimated the dose in the CTV volumes in every case. The average dose of the CTV volume calculated in the MCDC was lower than the cPBDC by 4.1 % in average. Compared to the cPBDC, the dose in 95 % of the CTV volume (D95) was lower than the cPBDC by 7.3 % in average. The result of the perturbed doses will be summarized.

We have verified the underestimation of the cPBDC in lung tissues for pencil beam scanning proton beam. On the other hand, the dose in organ-at-risk (OAR) has been benefited from the over-estimated cPBDC. The clinical implication of this result would be validated in a further investigation on long-term follow up. The MCDC for a clinical dose calculation in lung for PBS is highly recommended in SBPT cases.

### P 141: Stereotactic Ablative Body Radiotherapy versus Stereotactic Ablative Body Proton Therapy for Uncresectable Pancreatic Cancer: A Comparative Planning Study

#### R. Dalfsen^1^, H. Le^1^, S. Penfold^1^

##### *^1^Royal Adelaide Hospital, Department of Radiation Oncology, Adelaide*, *Australia*

**Purpose**: Proton Therapy has the ability to spare normal tissue more effectively in comparison with conventional photon therapy. This study dosimetrically compared Stereotactic Ablative Body Radiotherapy (SABR) and Stereotactic Ablative Body Proton Therapy (SABPT) for unresectable pancreatic cancer patients.

**Methods and Materials**: Ten patients who received SABR treatment at the Royal Adelaide Hospital between November 2016 and December 2017 were reviewed. Patients were prescribed 25-30 Gray (Gy) in five fractions to the tumour bed, depending on proximity to Organs At Risk (OAR). Blood vessels within 5mm were given a Simultaneous Integrated Boost (SIB) to 35Gy. SABR plans were generated using a Volumetric Modulated Arc Therapy (VMAT) technique with a 5mm Planning Target Volume (PTV) margin, accounting for inter-fractional and intra-fractional uncertainties. SABPT plans were robustly optimised to target volumes, with a 5mm immobilization uncertainty margin and a 3% proton range uncertainty margin. Dose-metrics for both techniques were utilised, assessing target coverage and (OAR) sparing. Stomach and duodenum Equivalent Uniform Doses (EUDs) were compared using RADBIOMOD, to assess Normal Tissue Complication Probability (NTCP).

**Results**: For both techniques, target coverage of D100%>95% was achieved in all cases. There was reduced radiation dose to OARs with SABPT, with data suggesting significant decrease in median dose to the duodenum, stomach, liver and kidneys.

**Conclusion**: This planning study suggests that SABR and SABPT techniques for the treatment of unresectable pancreatic cancer can offer acceptable dose coverage of target volumes. SABPT is able to reduce dose to OARs, which potentially decrease the risk of normal tissue complications.

### P 142: Influence of Anatomical Changes in Predicted Risk of Swallowing Dysfunction Following Proton and Photon Therapy in Oropharyngeal Cancer

#### G.M. Engeseth^1^, C.G. Boer^1^, M. Brydøy^1^, L.P. Muren^2^, O. Dahl^1^, C.H. Stokkevåg^1^

##### *^1^Haukeland University Hospital, Department of Oncology and Medical Physics, Bergen, Norway*, *^2^Aarhus University Hospital, Department of Medical Physics, Aarhus, Denmark*

**Objective**: Patients with oropharyngeal cancer are at risk of experiencing swallowing dysfunction following radiotherapy. Proton therapy can be considered due to potential reduction in side effects; however, the proton dose distribution may be more sensitive to anatomical changes during treatment than photons. The aim of this study was to investigate how anatomical changes influenced estimated risks of swallowing dysfunction in treatment plans using protons vs. photons.

**Methods**: Eight patients that underwent a repeat CT (rCT) due to anatomical changes during their treatment course with Volumetric Modulated Arc Therapy (VMAT) were selected for this study. Robustly optimised Intensity Modulated Proton Therapy (IMPT) plans were generated on the planning CTs (pCTs). The pCT and rCT were deformable registered, and target volumes and swallowing organs at risks were mapped to the rCT. The VMAT and IMPT plans were then recalculated on the rCTs. Differences in estimated Normal Tissue Complication Probabilities (ΔNTCP) between the pCT and rCT for several dysphagia endpoints were calculated (Table I).

**Results**: Estimated NTCP based on the pCT was generally lower with IMPT than with VMAT, with the largest differences between modalities seen in NTCPDysphagia and NTCPSolid (Figure 1). ΔNTCP was slightly larger for IMPT vs VMAT for two endpoints; median (range) ΔNTCPDysphagiawas 2% (2%-6%)(IMPT) and 0% (-1%-7%) (VMAT), while median (range) ΔNTCPSolid were 2% (-2%-7%) (IMPT) and 0% (-2%-8%) (VMAT) (Figure 2).

**Conclusion**: Anatomical changes had mostly modest impact on estimated NTCP for both proton and photon; however for some patients the increase in risk may justify re-planning.

### P 143: Investigation of TPS Parameters Used During Proton IMPT Optimisation and Their Effect on Dosimetry

#### C. Gillies^1^, A. Gosling^1^, A. Warry^1^, V. Rompokos^1^, A. Poynter^1^, D. D'Souza^1^

##### ^1^University College London Hospitals, Radiotherapy Physics, London, United Kingdom

In related literature it has been shown that the starting point of an IMPT optimisation affects the cost function minima. Within the treatment planning system many different parameters (Spot spacing, target margins for spot positioning, constants in a form of normal tissue control etc.) can be varied in order to give the user the means to optimally treat the target volume. These parameters affect the algorithm, the way it resolves to a final solution and as a result the dosimetry.

Our study involves the iterative delivery of a simple plan to a cylindrical phantom in Eclipse v13.7. Plans were optimised and calculated exploring the parameter space available in order to characterise the effect on the overall dosimetry. Conformity Index (CI) and Homogeneity (HI) index are measured alongside various other reporting metrics in order quantify the dosimetric effect.

Our results are limited to the geometry of the phantom, however a greater understanding of the parameters and their effect on the optimisation process is critical in order to capitalise on the flexibility of the planning system. Using the base set values provided by our analysed data, and from the understanding provided of how each variable affects the optimisation process, it will be possible to achieve an optimal dose distribution for each unique patient geometry in a more rapid time. This will speed up the overall planning process, improving the efficiency of the planning pathway.

### P 144: Development of a Semi-Customized Tongue Displacement Device Using A 3D Printer for Line-Scanning Proton Therapy of Head and Neck Cancer

#### C.S. Hong^1^, S.G. Ju^1^, D. Oh^1^, Y.C. Ahn^1^, C.H. Na^2^, C.C. Kim^1^, W. Lee^1^, K. Jo^1^, E. Chung^1^

##### *^1^Samsung medical center, Radiation oncology, Seoul, Korea Republic of*, *^2^Samsung Advanced Institute for Health Science & Technology- Sungkyunkwan University, Medical Device Management and Research, Seoul, Korea Republic of*

**Purpose**: To reduce radiation doses to the tongue, a patient-specific semi-customized tongue displacement device (SCTDD) was developed using a 3D printer (3DP) for line-scanning proton therapy (LSPT) in head and neck (H&N) cancer patients. Dosimetric characteristics of the SCTDD were compared with standard mouth-piece (SMP).

**Methods and Materials**: The SCTDD consists of three parts: a mouthpiece, connector with an immobilization mask, and tongue displacer, which can displace the tongue to the contra lateral side of the planning target volume (PTV) (Figure 1). Semi-customization was enabled by changing the thickness and length of the SCTDD. It was printed using a 3DP with a biocompatible material. With SCTDD and SMP, Two sets of the planning CT and LSPT plan using single-field optimization (SFO) were obtained for seven H&N cancer patient. Geometric and dosimetric characteristics were compared.

**Results**: Significant reduction was observed in the oral tongue volume (%) within the PTV, in SCTDD compared to SMP (8.7% vs 14.6% respectively, p=0.016). Significant median mean dose reduction was observed in SCTDD (18.3 CGE, IQR; 16.6, 23.1) compared to SMP (22.9 CGE, IQR; 21.2, 25.2). The volumes of tongue receiving a dose of 15, 30, 35, 45, and 60 CGE were significantly lower in SCTDD (37.6%, 27.4%, 24.8%, 19.8%, and 7.3%, respectively) than SMP (46.1%, 36.2%, 33.6%, 28.2%, and 10.3%, respectively) (p<0.05) (Table 1).

**Conclusion**: We developed unique SCTDD for H&N LSPT. The SCTDD significantly decreased the radiation dose to the tongue compared to SMP, which may potentially reduce RT-related toxicity in oral tongue.

### P 145: Generating Proton Stopping Power Images from Dual-Energy CT for Direct Import into Treatment Planning Systems

#### C.H. Hua^1^, F. Pirlepesov^1^, N. Shapira^2^, Y. Yagil^2^, P. Klahr^3^, T. Merchant^1^

##### *^1^St Jude Children's Research Hospital, Radiation Oncology, Memphis, USA*, *^2^Philips Medical Systems, Global Advanced Technology, Haifa, Israel*, *^3^Philips Healthcare, CT Clinical Science, Clevelad, USA*

**Background**: Dual-energy CT holds promise in improving range accuracy by generating proton stopping power ratio (SPR) images and eliminating the requirement of scan-condition-specific calibration curves. We report the implementation of direct calculation and import of SPR images from dual-energy CT scans into a treatment planning system (TPS).

**Methods**: Since 2016, patients were scanned on a CT scanner with dual-layered spectral detectors (Philips IQon Spectral CT) installed in a radiation oncology department. While the clinical workflow remained the same, the system performed projection-based spectral decomposition to derive photoelectric and Compton-scatter basis images, from which electron density (ED), a new development, and effective atomic number (Zeff) images can be synthesized. Zeff was further converted into the mean excitation energy (I value) using the fitted equations. SPR images were calculated with EDs and I values using the Bethe formula and imported into TPS.

**Results**: Beam hardening was reduced in calculated SPR images than those mapped from HU images, particularly in shoulder regions. Differences in SPR between contrast-enhanced and unenhanced scans were also reduced from 6% to ≤2%, which opens the possibility of using SPRs of post-contrast CT for dose calculation. Low-keV monoenergetic images were generated for two-times higher iodine enhancement to facilitate tumor delineation. Subtle differences existed in dose distributions on calculated and mapped SPRs. Patient and phantom data will be examined to determine if currently adopted range uncertainty can be reduced.

**Conclusions**: We demonstrated the workflow and feasibility of utilizing SPR images calculated from dual-energy CT scans for proton dose calculation in TPS.

### P 146: Metal Artifact Reduction Techniques during MR Simulation for Chordoma and Chondrosarcoma Proton Therapy Treatment Planning

#### S. Huh^1^, E. Kryck^1^, D. Indelicato^2^, R. Rotondo^2^, M. Rutenberg^2^, R. Dagan^2^

##### *^1^UF Health Proton Therapy Institute, Physics, Jacksonville- Florida, USA*, *^2^UF Health Proton Therapy Institute, Physicians, Jacksonville- Florida*, *USA*

Proton therapy has been found to be an effective treatment for chordomas and chondrosarcomas. CT-MR image registration and fusion can greatly improve the treatment plan and allow for better target localization. In cases where patients have metal implants near the tumor site, MR artifacts can pose a major problem for defining the tumor volume and organs-at-risk (OAR). Even when metal artifact reduction sequences are used, high-field strength diagnostic MR scanners can still have major artifacts, which make the images unsuitable for target and OAR contouring. Metal-induced susceptibility artifacts can be reduced by using low-field MR along with optimized pulse sequences. In this study, a bone phantom with titanium screws and aluminum plate was fabricated and used to optimize several pulse sequences (THRIVE, T1 FFE3D, and B-FFE3D) in a Philips 0.23T Panorama MR simulator. Several optimized pulse sequence parameters (phase, frequency modulation, and bandwidth) were experimentally determined. The detailed procedures of the artifact reduction techniques and clinical applications to proton therapy will be presented.

Image 1: Home-made phantom consisting of bone, 2 titanium screws, aluminum plate, and bolus material used to optimize pulse sequences. The artifacts in axial and sagittal planes are minimized.

Image 2: Clinical MR images of a patient with metal implants present during scan. 6 implanted rods as well as the spinal canal are able to be seen with minimal artifacts.

### P 147: Optimum Size of a Calibration Phantom for X-Ray CT to Convert Hounsfield Units to Stopping-Power-Ratios in Charged-Particle Therapy Treatment Planning

#### T. Inaniwa^1^, H. Tashima^2^, N. Kanematsu^3^

##### *^1^National Institute of Radiological Sciences- QST, Department of Accelerator and Medical Physics, Chiba, Japan*, *^2^National Institute of Radiological Sciences- QST, Department of Radiation Measurement and Dose Assessment, Chiba, Japan*, *^3^National Institute of Radiological Sciences Hospital- QST, Medical Physics Section, Chiba, Japan*

In charged-particle therapy treatment planning, the volumetric distribution of stopping power ratios (SPRs) of body tissues relative to water is used for patient dose calculation. The distribution is conventionally obtained from computed tomography (CT) images of a patient using predetermined conversion functions from the CT numbers to the SPRs. One of the biggest uncertainty sources of patient SPR estimation is insufficient correction of beam hardening arising from the mismatch between the size of the patient cross section and the calibration phantom for producing the conversion functions. The uncertainty would be minimized by selecting a suitable size of the cylindrical water calibration phantom referred to as an “effective size” of the patient cross section, Leffective. We investigated the Leffective for pelvis, abdomen, thorax, and head and neck regions by simulating an ideal CT system using volumetric models of the reference male and female phantoms. The Leffective values were 23.3, 20.3, 22.7 and 18.8 cm for the pelvis, abdomen, thorax, and head and neck regions, respectively, and the Leffective for whole body was 21.0 cm. Using the conversion function for a 21.0-cm-diameter cylindrical water phantom, we could reduce the root mean square deviation of the SPRs and their mean deviation to ≤ 0.011 and ≤ 0.001, respectively, in the whole body. Accordingly, for simplicity, the effective size of 21.0 cm can be used for the whole body, irrespective of body-part regions for treatment planning in clinical practice.

### P 148: A Software Tool for Analysis of Linear Energy Transfer and Biologically Effective Dose in Carbon-Ion Radiotherapy

#### N. Kanematsu^1^, N. Matsufuji^2^, M. Fukahori^3^

##### *^1^National Institute of Radiological Sciences Hospital- QST, Medical Physics Section, Chiba, Japan*, *^2^National Institute of Radiological Sciences- QST, Department of Accelerator and Medical Physics, Chiba, Japan*, *^3^National Institute of Radiological Sciences- QST, Clinical Research Cluster, Chiba, Japan*

In treatment planning of carbon-ion radiotherapy (CIRT), the relevant dose includes the weighting of relative biological effectiveness (RBE) of the radiation, which is related to linear energy transfer (LET) varying strikingly in a patient's body. Since the RBE is evaluated for a reference biological endpoint, there could remain systematic LET dependence in clinical response depending on disease type. In addition, hypofractionation being pursued in CIRT may also affect the clinical response, which is generally estimated by biologically effective dose (BED). Applying recently proposed formalisms, we have developed an in-house software tool ReXiON using programming language Python with package PyDICOM to convert physical and RBE-weighted dose distributions to LET and BED distributions for a treatment plan. We applied the tool to realistic examples to demonstrate its potential for investigation of the LET and fractionation effects, which would generally interest the CIRT community. Although the tool has been fully configured for our own use, it is basically a simple script and can be easily adjusted for others by themselves. It will potentially be useful for deeper understanding of clinical radiobiology.

### P 149: Algorithm Development for Inclusion of RBEs in Simulation and Treatment Planning

#### C. Keppel^1,2^, P. Ambrozewicz^3^, V. Nazaryan^4^

##### *^1^Thomas Jefferson National Accelerator Facility, Physics Division, Newport News, USA*, *^2^K&N Physics Consulting- LLC, Newport News, USA*, *^3^Eastern Virginia Medical School, Department of Radiation Oncology and Biophysics, Norfolk, USA*, *^4^Hampton University Proton Therapy Institute, Hampton, USA*

While proton therapy is becoming increasingly widespread and efficient, there persists uncertainty regarding the biological effects of proton irradiation, typically expressed in terms of relative biological effectiveness (RBE). A marked variation in the RBE for tumor cell killing along the depth dose profile of proton beams has been shown, while the standard is to apply a uniform factor of 1.1 in treatment planning. The development and implementation of a data-driven algorithm to account for RBE at the treatment planning level could alleviate this concern. To this end, an ongoing project has obtained an expanded RBE data set to implement RBE weighting into the Varian Eclipse treatment planning software product. Achieving this goal has required the development of an RBE model and an application algorithm. We anticipate the introduction of biological weighting factors that are specific for the linear energy transfer, LET, of protons at a given tissue depth to further improve the therapeutic ratio of proton therapy. However, the adopted definition of RBE turns this into a multi-dimensional problem, which can only be solved by rigorous data analysis. For this purpose a relational database has been developed to contain and compare the results of available experimental measurements. This turned out to be a surprisingly challenging undertaking as the available data suffers from often insufficient quantity reporting and statistical analysis. A summary and status of this work will be presented.

### P 150: Dosimetric Evaluation of Using In-house BoS Frame Fixation Tool for the Head and Neck Cancer Patient

#### K. Kim^1^, K.H. Jo^1^, B.K. Choi^1^

##### ^1^Samsung Medical Center, Radiation Oncology, Seoul, Korea Republic of

**Purpose**: We manufactured the fixation tool per se for improving the limits of BoS frame, and evaluated the utility of the manufactured fixation tool throughout this study.

**Materials and Method**: We selected 3 patients of head cancer who have received the proton therapy from our hospital, and also selected the 6 beam angles; for this, we selected the beam angle of the posterior oblique direction. We measured the planned BoS frame and the distance of Snout for each beam which are planned for the treatment of the patient using the BoS frame. After this, we proceeded with the set-up that is above the location, which was recommended by the manufacturer of the BoS frame, at the same beam angle of the same patient, by using our own fixation tool.

**Results**: When comparing the result before using the BoS frame fixation tool that was manufactured for each beam angle with the result after using the fixation tool, we could determine that the snout distance of the patient A reduced by 5.4 cm and 15.4 cm, the snout distance of the patient B reduced by 13.9 cm and 15.0 cm, and that of the patient C reduced by 12.1 cm and 7.3 cm.

**Conclusion**: It was possible to reduce the airgap by using our own manufactured BoS frame fixation tool for proton therapy; as a result, it was possible to figure out that the lateral penumbra reduced.

### P 151: Development of a Radiation Treatment Planning System for Heavy Charged Particle Radiation Therapy

#### M.J. Kim^1,2^, J.H. Park^3^, W. Cho^4^, S. Ahn^1^, T.S. Suh^2^, S.H. Choi^1^

##### *^1^Department of Radiation Oncology, Yonsei Cancer Center, Yonsei University College of Medicine, Yonsei University Health System, Seoul, Korea Republic of*, *^2^Research Institute of Biomedical Engineering- The Catholic University of Korea College of Medicine, Biomedical Engineering, Seoul, Korea Republic of*, *^3^Baylor Scott & White Health- Temple- USA, Radiation Oncology, Temple, USA*, *^4^Boramae Hospital- Seoul National University Hospital, Radiation Oncology, Seoul, Korea Republic of*

The primary goals of this project are to develop and validate heavy-ion radiation treatment planning system (TPS). Treatment scenario and data base/interface of treatment planning system, and graphic user interface has been developed. First of all, a database/interface of TPS was constructed to manage various input data and workflow of TPS and beam data was managed by beam data module for general specification of machine, ridge filter parameter, and measurement data for beam energy from 70-230 MeV in terms of percent depth dose (PDD) and in/cross-line. Also, image module was constructed to utilize computed tomography (CT) and to apply CT to density table and CT to stopping power table. Also, image reconstruction and registration has been developed for adaptive heavy-ion treatment, 2D/3D registration and 3D/3D deformable registration. The TPS and dose calculation engine was designed by C++ dynamic linking library. For dose calculation as a feasibility test of TPS system, pencil beam algorithm was applied to calculate the pencil beam beamlet and spread-out Bragg peak (SOBP) was calculated by using beam modeling of measurement data for SOBP-PDD data, which calculate the SOBP much faster than beam current modulation method using pristine bragg peak calculate.

Furthermore, development of optimization module for scanning beam and continuous feedback of developed treatment planning system has been performed. As a further study, cloud-based radiation dose calculations in both deterministic model-based algorithm and non-deterministic Monte Carlo will develop and provide compatibility with existing phase space sources and computational efficiency handling challengeable treatment planning.

### P 152: Planning Strategies for Treating Lung SBRT with PBS

#### Y. Lin^1^, M. Zhu^2^, C. Smith^3^, A. Waller^4^, Q. Wu^1^, M. Fang^5^, F.F. Yin^1^

##### *^1^Duke University Medical Center, Radiation Oncology, Durham, USA*, *^2^Maryland Proton Treatment Center- University of Maryland School of Medicine, Radiation Oncology, Baltimore, USA*, *^3^Varian Medical Systems, Proton Theray, Palo Alto, USA*, *^4^Varian Medical Systems, Velocity Software, Palo Alto, USA*, *^5^Zhejiang Provincial People's Hospital- People's Hospital of Hangzhou Medical College, Radiation Oncology, Zhejiang, China*

This study aims to develop planning strategies for lung SBRT with proton pencil beam scanning (PBS) technique and to investigate the robustness of the plans.

A typical lung SBRT patient case treated using photon technique was retrospectively used for developing PBS proton planning strategies. The original photon plan was generated to cover the planning target volume (PTV) with 5mm expansion to the internal target volume (ITV).

Five planning strategies were developed to generate proton plans based on a specific planning system (Eclipse protonTM), see Figure1: M1, using two fields to cover the PTV; M2, using one field to cover the field specific target (ITV with 5mm setup and 3.5% range uncertainties); M3, using two fields to cover ITV considering 5mm setup and 3.5% range uncertainties using robust optimization; M4 and M5, the same as M3 but with 5 and 9 fields, respectively.

The robustness of all plans against daily anatomical and setup variations was estimated. The planned doses were recalculated on the planning CT mapped to the daily CBCT through deformable registration to generate the delivered dose.

The photon plan generates the most conformal dose distribution (see Table1). The OAR doses are lower for all proton plans, especially at low does region. Poor M1 results may indicate that the PTV concept used for photon SBRT planning shouldn't be directly applied to proton. Strategies using M4 and M5 generate the most robust proton plans that ensure sufficient ITV coverage without irradiating excess amount of OARs as compared to other proton planning strategies.

### P 153: The Dosimetric Effect of Intravenous Contrast Agent on Passive Scattering Proton Therapy Plans for Head and Neck Cancer

#### B. Liu^1^, S. Kim^1^, N. Yue^1^, R. Parikh^1^, K. Nie^1^, R. Millevoi^1^, Y. Zhang^1^

##### ^1^Rutgers Cancer Institute of New Jersey- Robert Wood Johnson University Hospital, Radiation Oncology, New Brunswick, USA

**Purpose**: To evaluate the impact of an iodine-based intravenous contrast agent (CA) on proton therapy plans for head and neck (HN) cancer.

**Materials and Methods**: Ten HN cancer patients were included in this study. Two sets of computed tomography (CT) images were acquired for each patient before and after the CA injection. Target volumes and organs at risk (OAR) were contoured on the CTs with CA and copied to those without CA after image registration. For each patient, a clinical proton plan was created on the non-CA-enhanced CT with a passive-scattering machine in Varian Eclipse using proton convolution-superposition dose algorithm and the plan was recalculated on CA enhanced CT with identical beam parameters. The prescription dose ranges from 59.4 to 66Gy. The target coverage and OAR doses (left and right parotid, spinal cord and brainstem) from the two CT sets were compared.

**Results**: The difference in the dose received by 95% of PTV (D95%) between two CT sets is -0.16% ± 0.17% of the prescription dose (range, -0.41%-0.07%, n=10). The difference in the mean dose of left and right parotid is 0.00 ± 0.01Gy (range, -0.03Gy-0Gy, n=7) and -0.04 ± 0.06Gy (range, -0.17Gy-0Gy, n=7), respectively. The differences in maximum dose for brainstem and spinal cord are -0.61 ± 1.69Gy (range, -5.20Gy-0.89Gy, n=10) and -2.24 ± 2.31Gy (range, -6.39Gy-0.26Gy, n=10), respectively.

**Conclusion**: The CA has a relatively small impact on the dose coverage of HN proton plans. Additionally, it may cause OAR maximum dose difference up to several Gy.

### P 154: Dosimetric Study of Particle Radiotherapy for Inoperable Thymic Malignancy Patients

#### Y. Lu^1^, M.F. Moyers^1^, Y. Sheng^1^, K. Shahnazi^1^, J. Zhao^1^, W. Wang^1^, J. Chen^2^, J. Mao^2^

##### *^1^Shanghai Proton and Heavy Ion Center, Medical Physics, Shanghai, China*, *^2^Shanghai Proton and Heavy Ion Center, Department Of Radiation Oncology, Shanghai, China*

**Purpose**: To investigate the dosimetric differences between proton, carbon ion, and photon beam radiotherapy for inoperable thymic malignancy patients.

**Methods**: Ten inoperable thymic malignancy patients treated in our center were selected. Proton and photon plans used identical target prescriptions, 66 Gy(RBE) / 33Fx, while carbon plans used 66 Gy(RBE) / 22Fx. Plans were generated to provide same CTV coverage. The proton and carbon plans were generated using the Syngo V13B system while the photon plans used the Eclipse V11.0 system.

**Results**: There was no difference in CTV coverage for the three beam types. There was little difference in DVH data between proton and carbon ion plans; however, compared to proton and carbon ion plans, photon plans had a higher mean dose to heart (20.4±11.1 versus 14.7±5.9, median value ± std.dev, Gy(RBE), p=0.002, statistically significant), mean dose to main bronchi tree (MBT) (39.3±9.2 versus 29.3±17.0, p=0.017), mean dose to esophagus (ESO) (24.4±9.2 versus 10.3±10.9, p=0.000), D1% dose to spinal cord (SC) (38.6±7.5 versus 4.4±12.3, p=0.000), V20 Gy(RBE) of lung excluding CTV ((26.8±13.3) % versus (16.5±7.1) %, p=0.001) and mean dose of lung excluding CTV (15.4±4.6 versus 10.3±3.7, p=0.000). There were only slight differences between carbon ion and proton plans.

**Conclusion**: As shown in the figures, proton and carbon ion plans reduce the dose to SC, ESO, heart, and lung and, if the MBT is not within the target, also to the MBT. More thymoma patients will be studied to understand variation of doses to these normal structures in ion radiotherapy.

### P 156: LEM and Parameterized Approaches for the Estimation of Radiobiological and Clinically Relevant Quantities Using the Geant4 Monte Carlo Toolkit

#### G. Petringa^1,2^, L. Manti^3,4^, L. Pandola^1^, A. Attili^5^, F. Cammarata^6^, G. Cuttone^1^, P. Pisciotta^1,2^, G. Russo^6^, G.A.P. Cirrone^1^

##### *^1^Istituto Nazionale di Fisica Nucleare INFN, Laboratori Nazionali del Sud LNS, Catania, Italy*, *^2^Università degli Studi di Catania, Dipartimento di Fisica e Astronomia, Catania, Italy*, *^3^Istituto Nazionale di Fisica Nucleare INFN, Sezione di Napoli, Catania, Italy*, *^4^Università degli Studi Federico II di Napoli, Dipartimento di Fisica “E. Pancini”, Naple, Italy*, *^5^Istituto Nazionale di Fisica Nucleare INFN, Sezione di Torino, Catania, Italy*, *^6^CNR, Institute of Molecular Bioimaging and Physiology, Cefalu, Italy*

In this work, we will show the potentialities of the Geant4 Monte Carlo toolkit for the calculation of the RBE-weighted dose and cell survival dose-responses in therapeutic proton beams. Two different approaches have been developed to reach this goal: a pure computational method, coupling the Geant4 toolkit results and the version III of the LEM model; a parametrized approach of the Linear-Quadratic model, specifically developed for proton beam irradiations.

Tumor cell lines may vary considerably in them in-vitro response to radiation, which reflects the in-vivo intrinsic radiosensitivity of their tumor of origin. Hence, these approaches were applied to reproduce survival curves obtained during two different experimental campaigns conducted at the CATANA protontherapy facility of INFN-LNS (Catania, I).

The first campaign was conducted by using U87 radioresistant human glioma cells irradiated at six different depths along a 62 MeV of monochromatic and modulated clinical proton SOBP. The second one has been conducted irradiating the more radio-responsive prostate DU145 cells along three points of the same SOBP beam. The whole experimental set-up of this two campaigns, including the transport beamline with the diagnostic elements and the cellular irradiation conditions, were accurately simulated.

We have shown that a LEM-based and a parameterised approach, both performed with Monte Carlo simulation, can adequately reproduce experimentally derived survival curves from in-vitro cancer cell irradiations performed with a clinical proton beam.

### P 157: Decreasing Dose to the Femurs Using Multi-Field Optimization in PBS Prostate Treatment

#### S. Petro^1^, B. Wilkinson^2^, B. Robison^1^, J. Renegar^1^, M. Blakey^1^, M. Artz^1^, N. Schreuder^1^

##### *^1^Provision CARES Proton Therapy Center, Medical Physics, Knoxville- TN, USA*, *^2^Provision CARES Proton Therapy Center, Radiation Oncology, Knoxville- TN*, *USA*

Prostate cancer is typically treated with PBS proton therapy using two opposed lateral beams, which pass through the femurs to treat the whole prostate. Using single field optimization (SFO), each beam treats the entire prostate. Multi-field optimization (MFO) uses complementary beams to conform to the prostate, with each field treating only part of the target. Because each MFO field is not required to treat the entire target, the SOBP width can be decreased for each field. Since the entrance plateau region dose is dependent on the SOBP width, we hypothesized that the femoral head dose could be reduced using MFO.

Five SFO prostate plans were reoptimized using an MFO technique to examine the reduction in femoral head dose. Because each MFO field is dependent on the other treatment field, any shift in anatomy can more significantly affect target coverage than for an SFO plan. To test clinical robustness, the MFO plans were recalculated on QACTs (CTs acquired throughout treatment to monitor anatomical changes) and tumor coverage was assessed.

Utilizing MFO, the femur D50 (dose to 50% of the volume) was decreased by, on average, 10.9±1.3% while maintaining tumor volume coverage and OAR (organ at risk) sparing. The MFO plans were appropriately robust, with less than 1% change in prostate D95 for all QACTs.

In this study, we have shown that an MFO approach to treating prostate cancer with PBS proton therapy can reduce femoral head dose while maintaining robust tumor coverage.

### P 158: Estimation of Phantom RSP Using an X-Ray Flat Panel Detector and a Scanned Proton Beam up to 330MeV

#### M. Petterson^1^, S. Penfold^2^, D. Lee^3^

##### *^1^ProTom International, Physics, Boston, USA*, *^2^Royal Adelaide Hospital, Medical Physics, Adelaide, Australia*, *^3^ProTom International, Physics, Boton,* USA

The benefit of using protons for targeted radiation therapy is well known, but the accuracy of beam delivery to the treatment area is dependent on the accuracy of the predicted relative stopping power (RSP) along the beam trajectory. The 3-5% error incurred during the HU-RSP calculation substantiates the advantages of using proton radiography to directly measure the patient RSP. While most anatomical structures can be imaged with protons of energy 230MeV or less, higher energies are required for regions with a water equivalent path length (WEPL) greater than 30cm. Here, we use beam energies up to 330MeV to produce proton radiographs of phantoms with a WEPL up to 45cm.

A scanning pencil beam with energy ranging from 100MeV to 330MeV was used to generate radiographs of several phantoms. The proton beam energies were controlled by the synchrotron; no energy degraders were needed. An X-ray flat panel was used to record the protons' position and residual range, allowing easy pre-treatment verification of patient positioning without specialized equipment. The phantoms' WEPL was found by calculating the energy resolved dose function (ERDF) for absorbers of known water equivalent thickness (WET) ranging from 50mm-450mm and comparing the ERDF to the X-ray panel response during phantom imaging. The RSP values of the phantoms were then calculated.

The proton doses to generate the radiographs were ∼8mGy. Range estimates using the X-ray panel necessitate choosing a proton energy such that the Bragg peak of the protons occurs near the scintillating material, resulting in a larger absorbed dose.

### P 159: Proton Range Verification Using Radio-Chromic Film in a Head Phantom

#### C. Sarosiek^1^, G. Coutrakon^1^, D. Johnstone^1^, B. Kreydick^2^, A. Panchal^2^, M. Gao^2^, R. Schulte^3^

##### *^1^Northern Illinois University, Physics, Dekalb, USA*, *^2^Northwestern Medicine Chicago Proton Center, Medical Physics, Warenville, USA*, *^3^Loma Linda University, Division of Biomedical Engineering Science, Loma Linda, USA*

Proton range uncertainties have long been a concern for optimal proton dose delivery. A central question is, how much error is attributable to the X-ray CT imaging modality in proton treatment planning? To address this question, a series of proton beam irradiations with film dosimeters embedded in a head phantom have been performed with X-ray CT, dual energy X-ray CT and proton CT imaging. Beam enters perpendicular to the stack of 36 films each having 0.35 mm water equivalent thickness. The beam in treatment planning is designed to stop at a calculated depth in the film stack. The head phantom is then irradiated with the planned beam and we measure the deviation of the measured stopping depth by comparing the dose distributions from treatment planning with actual measurements on the 36 films. We report on the range errors using these three imaging modalities in the commercial treatment planning systems, Xio and Ray Station, in use at Northwestern Medicine Chicago Proton Center.

### P 160: Dosimetric commissioning of a Pencil Beam and a Monte Carlo Dose Calculation Algorithm for a Synchrotron Based Proton PBS Delivery

#### A. Carlino^1^, G. Kragl^1^, T.T. Böhlen^1,2^, J. Osorio^1^, L. Grevillot^1^, S. Vatnitsky^1^, M. Stock^1^

##### *^1^EBG MedAustron GmbH, Medical Physics, Wiener Neustadt, Austria*, *^2^Paul Scherrer Institute PSI, Proton therapy, Villigen, Switzerland*

In this work we report the dosimetric commissioning of a PB and a MC algorithm available for proton PBS delivery in the TPS RayStation v6.1. The work was subdivided into two parts: 1D/2D commissioning consisted of benchmarking both algorithms against measured IDDs, lateral spot profiles in air and measurements in presence of lateral heterogeneities; 3D commissioning consisted of characterization of 3D dose distributions with increasing complexity up to clinical cases. A robotic patient positioning system is used to reduce the air gap between patient and nozzle, therefore special attention was paid to non-isocentric setups especially in presence of Range Shifter (RaShi).

IDDs were acquired with a Bragg peak chamber. Spot profiles in air were acquired with a scintillating screen. Measurements of transverse dose profiles in water in presence of lateral heterogeneities were carried out with a micro-diamond detector. 3D dose distributions were characterized using a 24 PinPoint chambers array.

Calculated ranges for both algorithms agreed within 0.25mm with measured ranges. Better agreement of MC (5%) in comparison to PB (10%) for spot sizes in the presence of RaShi was obtained. The measurements with micro-diamond showed superior performance of MC in comparison to PB in presence of lateral heterogeneities at larger depths in water (table 1). For complex 3D dose distributions with RaShi again MC revealed superior performance compared to PB (fig. 1).

Clinically acceptable results were obtained for beams without RaShi for both algorithms. For plans with RaShi and in presence of lateral heterogeneities, MC showed substantially improved performances compared to PB.

### P 161: Doses to Cognitive Brain substructures In Photon vs Proton Therapy of Pediatric Supratentorial Brain Tumors

#### L. Toussaint^1^, D. Indelicato^2^, Y. Lassen-Ramshad^3^, C. Stokkevåg^4^, C. Pedro^5^, M. Di Pinto^6^, Z. Li^7^, S. Flampouri^7^, M. Høyer^8^, L. Muren^1^

##### *^1^Aarhus University Hospital, Medical Physics, Aarhus, Denmark*, *^2^University of Florida, Radiation Oncology, Jacksonville, USA*, *^3^Aarhus University Hospital, Radiation Oncology, Aarhus, Denmark*, *^4^Haukeland University Hospital, Oncology and Medical Physics, Bergen, Norway*, *^5^Instituto Português de Oncologia de Lisboa Francisco Gentil, Radiotherapy, Lisbon, Portugal*, *^6^University of Florida, Developmental-Behavioral Pediatrics, Jacksonville, USA*, *^7^University of Florida, Medical Physics, Jacksonville, USA*, *^8^Danish Center for Particle Therapy, Oncology, Aarhus, Denmark*

**Purpose**: Supratentorial brain tumors are close to several cognitive brain substructures (CBSs). Proton therapy is therefore used to spare the CBSs from radiation. The aim of this study was to explore the patterns in doses to a broad range of CBSs for photon versus proton therapy, for two supratentorial brain tumor entities.

**Material and methods**: CT/MRI-scans from ten pediatric patients with craniopharyngioma (CP) and ten with supratentorial hemispheric ependymoma (HE, four left/six right cases) were used to delineate critical structures as well as thirty unique CBSs. Volumetric modulated arc therapy (VMAT) and intensity modulated proton therapy (IMPT) plans were generated for a prescribed dose of 54Gy/CGE (CP) and 59.4Gy/CGE (HE). For each CBS, differences in the fraction of volume receiving low (V10-V20Gy), intermediate (V30-V40Gy) and high (V50-V60Gy) doses were analyzed.

**Results**: The volumes of CBSs exposed to low doses were lower with IMPT compared to VMAT. In the CPs, intermediate-to-high dose levels were higher with IMPT for the CBSs located at intermediate distances from the target, but comparable for the lobes and hippocampal structures. In the HEs, the techniques were comparable for all CBSs across the intermediate-to-high dose levels.

**Conclusion**: The volumes of CBSs receiving low doses were consistently lower with IMPT. The differences in the intermediate-to-high dose volumes highly depended on the distance between the CBSs and the tumor for CPs, while for HEs the two techniques were comparable.

**Acknowledgements**: Anne Vestergaard, Jørgen Petersen, Ronni Mikkelsen and Henrik Schrøder (all Aarhus University Hospital) were also involved in this study.

### P 162: Inter-center Variability in CT Number to Stopping-Power Conversion in Particle Therapy: Survey-based Evaluation

#### V. Taasti^1^, L. Muren^1^, P. Jørgen^1^, D. Hansen^1^, C. Richter^2^

##### *^1^Aarhus University / Aarhus University Hospital, Department of Medical Physics- On behalf of the European Particle Therapy Network EPTN- an ESTRO task group, Aarhus, Denmark*, *^2^OncoRay – National Center for Radiation Research in Oncology, Faculty of Medicine and University Hospital Carl Gustav Carus- Technische Universität Dresden- Helmholtz-Zentrum Dresden- On behalf of the European Particle Therapy Network EPTN- an ESTRO task group, Rossendorf- Dresden, Germany*

**Purpose:** Protocols for conversion between CT number and stopping-power ratio (SPR) in treatment planning of particle therapy are not standardized with respect to image acquisition and reconstruction. The CT-to-SPR conversion (Hounsfield Look-Up-Table, HLUT) depends on the scan settings and must be defined by each center. To assess inter-center variability in this conversion, we performed a survey-based evaluation within the framework of the European Particle Therapy Network (EPTN).

**Methods:** A questionnaire asking for details on CT scanners, image acquisition and reconstruction, calibration and definition of the HLUT was sent to twelve centers. The questionnaire also assessed investigations of the influence of beam-hardening and experimental validations of the HLUT. Technological improvements were rated regarding their potential to improve SPR accuracy. The participating centers were: Dresden, Essen, Groningen, Heidelberg, Kraków, Orsay, Pavia, Philadelphia, Rochester, Uppsala, Villigen and Wiener Neustadt.

**Results:** Reconstruction kernels, beam-hardening corrections, and HLUT generation showed large variation between centers. Eight centers used stoichiometric calibration for HLUT definition. All facilities performed a piecewise-linear fit, yet the number of line segments used varied from 2-11. Nine centers had investigated the influence of beam-hardening, and seven had evaluated the patient-size dependence of their HLUT. Ten centers had validated their HLUT experimentally, but the validation schemes varied widely. Dual-energy CT was deemed promising for improving SPR accuracy.

**Conclusions:** The survey gave a detailed overview of current practice in CT-to-SPR conversion, showing a large inter-center variability for all parameters affecting the HLUT. A future standardization would reduce time-intensive institution-specific efforts and variations in treatment quality.

### P 163: Robustness Evaluation of Patient Rotational Orientation for the Anterior-To-Posterior Pencil Beam Scanning Field Used in the Head and Neck Patients

#### S. Turner^1^, C. Chen^1^, H. Liu^1^, P. Fox^1^, N. Ju^1^, S. Kevin^1^, N. Lee^2^, D. Mah^1^

##### *^1^ProCure Proton Therapy Center, Medical Physics, Somerset, USA*, *^2^Memorial Sloan Kettering Cancer Center, Radiation Oncology, New York, USA*

**Background**: The anterior neck nodal volumes of head-and-neck patients are treated by an AP field matched superiorly with posterior beams. We evaluated the robustness of the AP field to patient rotation, roll and pitch in addition to translations in the treatment planning system. The changes result mostly from chin position.

**Materials and Methods**: Eight head-and-neck patients treated with an AP field were evaluated. The rotation and roll of the patients were simulated by changing couch and gantry angles respectively. The pitch was simulated by rotating the couch and corresponding spot fluence by 90° and varying the gantry angle (Fig. 1). Dose variance of the PTV, spinal cord and larynx were evaluated for 3° variations of pitch, roll and rotation (Tab.1).

**Results**: The PTV irradiated by the AP field (AP-PTV) was 48.2% (range: 29.2%, 79.8%) of the total PTV. The AP-PTV coverage was more sensitive to rotation with a mean of -3.0% (range: -9.9%, 5.1%). The increase of the maximum spinal cord point dose could be up to 10.5 Gy(RBE) for changes in pitch. Rotations increased the larynx mean dose by 15.8% in average (range: -10.1%, 157.4%). The dose variations due to patient's roll were between these rotation and pitch.

**Conclusions**: Systematic variations in chin position could cause significant changes in PTV coverage and OAR doses. The PTV coverage and larynx mean dose appear to be more sensitive to rotation and spinal cord maximum dose to pitch. Extra care should be taken to ensure chin position is accurate (Fig. 1 Simulations of patient's rotational orientation; Table 1 Dose variances).

### P 164: Validation of CT Calibration for Proton Treatment Planning by Real Tissue

#### V. Vondracek^1^, M. Navratil^1^, K. Badraoui-Cuprova^1^, M. Andrlik^1^, J. Vilimovsky^1^, S. Stastna^1^

##### ^1^PTC Prague, Medical Physics, Prague, Czech Republic

Range uncertainty is one of main issues in proton radiotherapy. Among all sources of this uncertainty the main role plays calibration of CT scanner. In principle CT scanner provides map of linear attenuation for X-rays and such map must be converted into mass stopping powers of material or mass density. Usual calibration by specific tools with ICRU tissue equivalent inserts are not necessarily precise enough.

In our work we used real animal tissues with different composition and different parts of animal body and provided CT scan and also WET measurement directly with proton beam. The results of this measurements were compared to calculated values from our TPS in the means of gamma analysis. It has shown that some minor adjustments of calibration curve is worth to make against the one obtained by phantom with ICRU inserts

### P 165: Two Years Proton Therapy Experience on Machine Parameters in Operation at Chang Gung Memorial Hospital Proton Center

#### C.Y. Yeh^1^

##### *^1^Chang Gung Memorial Hospital at Linkou, Radiation Oncology, Taoyuan, Taiwan*\r

**Purpose**: The wobbling system with ridge filter or layer stacking technique providing a formation of 3D uniform dose distribution has been introduced at G1 and G2 since Dec. 2015. The pencil beam system with single field uniform dose (SFUD) technique and multiple field optimization (MFO) technique was introduced at G3 and G4 on Dec. 2016. In order to realize the machine operation, we analyzed the machine parameters in term of delivery technique, proton energy, gantry angle, width of SOBP.

**Methods and Materials**: 1,510 fields in 677 patients by using wobbling system and 615 fields in 245 patients by using pencil beam scanning system were analyzed in this report. The treatment parameters including disease site, delivery technique, proton energy, gantry angle and width of SOBP were selected for analysis.

**Results**: In wobbling system, 54.5% of 677 patients were liver and lung cases treated by ridge filter technique. Gantry angle at 180^0^∼210^0^, 250^0^∼320^0^ and 0^0^∼20^0^ were frequently selected in wobbling system. SOBP at 5cm∼10cm and 3cm∼6cm were frequently used for ridge filter and layer stacking technique, respectively. In pencil beam system, 63.2% of 247 patients were brain and head & neck cases. Gantry angle at 180^0^∼184^0^, 210∼230^0^, 40^0^∼60^0^ and SOBP at 6cm∼12cm, 16cm∼19cm were frequently selected in pencil beam system.The range of energy frequently delivered by cyclotron was 155MeV±40MeV for wobbling and pencil beam.

**Conclusions**: From clinical proton therapy experience, we have found that the diversity of tumor shape and location in the body have a significant influenced in determination of delivery technique, proton energy, gantry angle and SOBP.

### P 167: Evaluation of Iterative Metal Artifact Reduction (iMAR) Tool for CT Simulation and Treatment Planning in Particle Radiotherapy

#### J. Zhao^1,2^, X. Wu^1^, Y. Sheng^1^, K. Shanhnazi^1^

##### *^1^Shanghai Proton and Heavy Ion Center, Medical Physics, Shanghai, China*, *^2^Fudan University Shanghai Cancer Center, Radiation Oncology, Shanghai, China*

**Purpose:** The study is to evaluate the iterative metal artifact reduction (iMAR) performance for HU accuracy improvement on CT with metal implants, its effect on treatment planning dosimetry and radiation oncology practice on patient case with metal implants.

**Methods:** Both phantom and patient data were evaluated in this study. Gammex 467 phantom was scanned on a Siemens Definition AS CT simulator in the presence/absence of metal (Al, Ti, TiAlloy and two TiAlloy). The relative HU changes of plugs and dosimetric accuracy on iMAR reconstructed images from the reference CT (no metal inserted) were analyzed. One treatment plan of patient with artificial femur head replacement optimized on the original images with artefact and metal implants overridden was recalculated on the iMAR reconstructed images. DVH comparison and Gamma analysis were performed on the plans.

**Results:** The results of phantom study showed that HU number accuracy was improved in the iMAR reconstructed images. The phantom dosimetric study showed that range calculation difference was improved from 3mm to less than 0.1mm before and after iMAR correction on bilateral metal plugs inserted images compared to reference images. On dosimetric study of patient treatment plans, there is no significant differences of DVHs and pass rate of Gamma analysis is 96.2%.

**Conclusion:** The study indicated that iMAR tool can help to improve the artefact and HU number accuracy in CT images with metal implants. Both phantom and patient plan dosimetric study demonstrated that iMAR reconstructed image can offer better anatomical structure visualization and dose calculation baseline images.

### P 168: Dosimetric Comparison between the Use of the Standard PROBEST-V Range Shifter and Custom-Made Range Shifter Slabs for IMPT

#### L. Zhao^1^, M. Moncion^2^, D. Sobczak^1^, M. Krasin^1^, V. Moskvin^1^, T. Merchant^1^

##### *^1^St. Jude Children's Research Hospital, Radiation Oncology, Memphis, USA*, *^2^St. Jude Children's Research Hospital, Department of Radiation Oncology, Memphis*, *USA*

Nozzle range shifter (RS) may be used to deliver dose to shallow depths in the patient for IMPT. The Hitachi PROBEAT–V spot scanning system has energies in the range of 69.4 MeV-221.3 MeV with non-retractable nozzle. RS with WET of 4 cm was installed on the nozzle with the RS downstream side at a distance of 49cm from the isocenter. The spot size at the isocenter is 34 mm and 4.7 mm at 69.4 and 221.3 MeV with the standard RS. To reduce the spot size, the air gap between patient and RS was reduced by substitution of the standard RS with custom made. The RS slabs made of PMMA with WET of 4 cm were fabricated and used in place of standard RS. The custom made RS slab was located with the downstream side of the slab at 12.4 cm from isocenter. The dosimetric effect between the use of conventional RS and custom RS slabs were compared for proton range between 6 cm to 16 cm in Eclipse TPS (V13.7.15). The lateral penumbra (20%-80%) was reduced from 1.65 cm -2.67 cm to 1.01 cm -1.69 cm, respectively. A patient with the target of the parapharyngeal space was planned using both RS and custom slabs with the same beam orientation (LL and PA). Comparable target coverage was achieved with both plans and meaningful dose reductions to the normal tissues such as spinal cord, brain stem and right optical nerve were observed with the use of custom made RS slabs.

### P 169: Synchrotron-Based Pencil Beam Scanning Nozzle with an Integrated Mini-Ridge Filter: A Dosimetric Study to Optimize Treatment Delivery

#### X. Wang^1^, Y. Li^2^, X. Zhang^2^, H. Li^2^, H. Hkiyama^3^, M. Gillin^2^, D. Grosshans^4^, B. Gunn^4^, S. Frank^4^, R. Zhu^2^

##### *^1^Sichuan Cancer Hospital & Institute-, Radiation Oncology, Chengdu, China*, *^2^MD Anderson Cancer Center, Radiation Physics, Houston, USA*, *^3^Hitachi Ltd., Healthcare Business Unit- Particle Therapy Division-, Ibaraki-ken, Japan*, *^4^MD Anderson Cancer Center, Radiation Oncology, Houston, USA*

To facilitate the clinical use of a mini-ridge filter, we performed a planning study for the feasibility of a mini-ridge filter as an integral part of the synchrotron nozzle (IMRF). Dosimetric characteristics in a homogenous water phantom were compared between plans with and without IMRF for a fixed spread-out Bragg peak width of 4 cm with various distal ranges. Six clinical cases were then used to compare the plan quality between plans. The delivery efficiency was also compared between plans in both the phantom and the clinical cases. The Bragg peak width was increased by 0.18 cm at the lowest energy and by only about 0.04 cm at the highest energy. The IMRF increased the spot size (sigma) by up to 0.1 cm at the lowest energy and by only 0.02 cm at the highest energy. For the phantom, the IMRF negligibly affected dose at high energies but increased the lateral penumbra by up to 0.12 cm and the distal penumbra by up to 0.06 cm at low energies. For the clinical cases, the IMRF slightly increased dose to the organs at risk. However, the beam delivery time was reduced from 18.5% to 47.1% for the lung, brain, scalp, and head and neck cases, and dose uniformities of target were improved up to 2.9% for these cases owing to the reduced minimum monitor unit effect. In conclusion, integrating a mini-ridge filter into a synchrotron nozzle is feasible for improving treatment efficiency without significantly sacrificing the plan quality.

### P 170: Evaluation of New 2D Ripple Filters in Scanned Proton Therapy

#### T. Printz Ringbæk^1^, U. Weber^2^, A. Santiago^1^, Y. Simeonov^3^, A. Wittig^4^, G. Iancu^1^, L. Grzanka^5^, N. Bassler^6^, R. Engenhart-Cabillic^1^, K. Zink^3^

##### *^1^Philipps University Marburg, Department of Radiotherapy and Oncology, Marburg, Germany*, *^2^GSI Helmholtzzentrum für Schwerionenforschung GmbH, Biophysics Group, Darmstadt, Germany*, *^3^University of Applied Science Gießen-Friedberg, Institute for Medical Physics and Radioprotection, Gießen, Germany*, *^4^Jena University Hospital, Radiotherapy and Oncology, Jena, Germany*, *^5^Polish Academy of Sciences, Institute of Nuclear Physics-, Krakow, Poland*, *^6^Stockholm University, Medical Radiation Physics, Stockholm*, *Sweden*

We have shown previously that ripple filters (RiFis) of an improved 6 mm thick design with two-dimensional pins can be used in scanned carbon ion therapy for widening the Bragg peak (BP) to reduce the accelerator energy shifts needed to homogeneously cover the target, thus reducing the irradiation time. This design could potentially be used in proton therapy too, widening the BP to an extent beneficial in treatment planning. RiFis are normally not used with protons due to larger scattering and straggling effects. Measured proton Bragg curves confirm the functionality of the new RiFi. Assuming a multi-ion facility, baseline data for treatment planning were generated with the Monte Carlo code SHIELD-HIT12A and imported in the treatment planning systems TRiP98 and proton Eclipse. Proton plans on spherical targets in water were calculated in TRiP98 for a systematic RiFi performance analysis and for comparisons with carbon ion plans. For a dosimetric evaluation on clinical cases, proton plans for 10 NSCLC lung tumours fixated under high-frequency jet-ventilation were calculated in Eclipse. Slightly worse dose conformity and homogeneity were found with RiFis compared to without with a general increase in conformity and homogeneity as a function of target size and penetration depth. These effects are more pronounced for protons. For certain small superficial targets requiring low beam energies, the 6 mm RiFi might result in an unacceptable large lateral beam broadening. This could be lowered by a combined RiFi and range shifter setup or by reducing the distance between the patient and the RiFi.

### P 172: Optimization of a Monte Carlo Based Robust Optimizer for IMPT on a GPU Cluster for Clinical Use

#### A. Abdel-Rehim^1^, J. Ma^1^, H. Kamal Syed^1^, H.S. Wan Chan Tseung^1^, M. Herman^1^, C. Beltran^1^

##### ^1^Mayo Clinic, Radiation Oncology, Rochester- MN, USA

**Purpose**: Develop an efficient Monte Carlo based robust optimizer for IMPT.

**Methods**: A locally developed Monte Carlo and GPU based robust optimization package for IMPT was used. Based on earlier profiling work, we identified important bottlenecks and how to resolve them. First improvement was to keep data on GPUs as much as possible to avoid memory latency. The second improvement is to perform the optimization on a down-sampled CT data set without compromising the plan quality. The code is tested on our development cluster with 10 compute nodes each with 4 K80 GPUs. Six test cases are used including: head and neck, lung, pubis, pediatric orbit, and prostate. Robust optimization was done on 9 scenarios including positional shifts of +/-3mm and 3% range uncertainty. Scaling of the code is done between 1 and 6 nodes and down-sampling was performed giving voxel sizes from 2.5mm to 8mm for testing.

**Results**: In Figure [1] we show a sample scaling results for the head and neck case showing close to optimal scaling. Encouraging results was obtained with optimization time ranging from few minutes in small cases to about 45 minutes for the hardest case. Figure [2] shows a sample results for the variation of DVH points relative to the native resolution showing less than 10% variation below 3mm in this case. In our tests, a 2.5mm voxel size was used.

**Conclusions**: Efficient robust optimizer for a GPU cluster is developed. Down-sampling to voxel size of 2.5mm didn't lead to plan degradation quality.

### P 174: Small Field Aperture Validation of the Raystation Proton PBS Monte Carlo Algorithm

#### M. Blakey^1^, M. Janson^2^, B. Saad^3^, T. Bald^3^, N. Schreuder^4^, B. Robison^4^, J. Renegar^4^, S. Hedrick^5^, M. Artz^4^, S. Petro^4^

##### *^1^Provision Cares Proton Therapy- Nashville, Proton Therapy, Nashville, USA*, *^2^RaySearch Laboratories, Development, Stockholm, Sweden*, *^3^Vanderbilt University, Radiation Oncology, Nashville, USA*, *^4^Provision Cares Proton Therapy- Knoxville, Proton Therapy, Knoxville, USA*, *^5^Emory University, Proton Therapy, Atlanta, USA*

**Purpose**: The RayStation 6 TPS provides proton PBS users the ability to optimize with an aperture. Previous work at the Northwestern Proton Center validated the algorithm down to an aperture field size of 4x4cm^2^. This study aims to validate the algorithm down to a 1cm diameter aperture. Ion chamber measurements became the focus of this validation testing as radiochromic film experiences significant quenching in proton fields.

**Method**: Three plans with 1cm, 2cm, and 3cm diameter apertures, including a 4.0cm WET range shifter were created in RayStation TPS. Provision's standard beam model was used (reference field = 10x10cm^2^ with no range shifter or aperture present). Longitudinal profiles were measured in a 1D water tank using the IBA PPC05 (sensitive area = 76.9mm^2^). Transverse profiles were measured at mid-SOBP using the MatriXX PT (sensitive area = 15.9mm^2^) in solid water. The measured depth doses and lateral profiles were then compared to the predicted doses from RayStation, considering the lateral extension of the PPC05 and MatriXX ionization chambers.

**Results**: Comparing the calculated depth dose profiles to PPC05 measurements, an average SOBP dose difference (computed-measured) for the 1cm, 2cm, and 3cm diameter apertures was 2.8%, -2.0% and -1.0%, respectively. The range discrepancy between calculated and measured was less than 1mm. The measured transverse profiles at mid-SOBP were in excellent agreement to the calculated profiles, with dose differences of +2.1%, -1.7%, and +2.1% for the 1cm, 2cm, and 3cm apertures, as evaluated for the Matrixx chamber aligned with the central axis.

### P 175: Automated Proton Planning Using Robust Mimicking of the Reference Photon Dose for Proton Therapy Patient Selection

#### R. Kierkels^1^, A. Fredriksson^2^, S. Both^1^, J.A. Langendijk^1^, D. Scandurra^1^, E.W. Korevaar^1^

##### *^1^University Medical Centre Groningen, Radiation Oncology, Groningen, Netherlands*, *^2^RaySearch Laboratories AB, RaySearch Laboratories AB, Stockholm, Sweden*

Patient selection for proton therapy is increasingly based on proton to photon plan comparisons. To improve efficient decision making, we developed a dose mimicking and reduce (DMR) algorithm to automatically generate a robust proton plan from a reference photon dose and target and organs at risk (OAR) delineations.

The DMR algorithm was evaluated in 40 head-and-neck cancer patients. The first step of the DMR algorithm comprises DVH-based mimicking of the photon dose distribution in the clinical target volumes and OARs. Target robustness is included by mimicking the nominal photon dose in 21 perturbed scenarios. The second step of the optimization aims to reduce the OAR doses as much as possible while retaining the robust target coverage as achieved in the first step. We evaluated each DMR plan against the photon plan and a “manually” robustly optimized reference proton plan in terms of plan robustness (voxel-wise minimum dose) and normal tissue complication probabilities (NTCPs) of xerostomia, dysphagia and tube feeding dependence.

The nominal dose of the DMR and reference proton plans were very similar (representative case in figure 1). The difference of the V95% voxel-wise minimum dose between the DMR and reference proton plan was <0.05 in 34/40 cases. Regardless of proton plan (i.e. DMR or reference proton plan) the same treatment modality was selected in 29/40 cases based on the sum of ΔNTCPs between the proton and photon plans (figure 2).

In conclusion, the DMR algorithm automatically optimized robust proton plans from a photon reference dose in head-and-neck cancer patients.

### P 176: Validation and Clinical Implementation of GPU-based Monte Carlo Fast Dose Calculator for Dose Verification in Modulated Scanned Ion Beam Therapy

#### L. Hong^1^, H. Chen^1^, Z. Chen^1^, Q. Wang^2^, P. Yepes^2^, M. Moyers^1^

##### *^1^Shanghai Proton and Heavy Ion Center, Department of Medical Physics, Shanghai, China*, *^2^Rice University, Department of Physics and Astronomy, Houston, USA*

**Purpose:** To validate and implement the clinical use of a GPU-accelerated Monte Carlo (MC) Fast Dose calculator (gFDC) to support routine patient-specific dose verification for proton and carbon ions.

**Materials and Methods:** The GPU-accelerated MC track-repeating algorithm based gFDC was used to simulate the radiation head and patient-specific portal setup according to the treatment delivery system of our facility. Next, it was benchmarked against measurements using a 2-D planar ionization chamber array embedded in a rectangular solid water phantom. Finally, an *in house* developed TPS named TIMPS was used to import from a commercial TPS (Siemens Syngo PT Planning VC13) the CT images, anatomical model and plan parameters, and establish a link to gFDC. The gFDC re-calculated 7 treatment plans (including H&N, Lung, Liver, Prostate, Cervical, Sarcoma cases) and 20 verification portals were validated against both measurements and the pencil-beam-based dose calculation by Syngo.

**Results:** For phantom as well as patient cases, good agreements between gFDC, measurements and Syngo were observed. The 3-D gamma passing rate for the 3%/3mm criterion is over 97.8% in the region with dose greater than 10% maximum dose, and for 2%/2mm criterion, gFDC showed slightly better results as expected. With gFDC it takes only 6 to 17 s to simulate 100 million ions per portal to achieve ∼ 0.3% relative statistical uncertainty, depending on energy.

**Conclusion:** Our GPU-based Fast Dose Calculator embedded in TIMPS can facilitate the routine clinical use of patient-specific ion beam dose verification. The results support continued development for treatment planning.

### P 177: How Accurate Is the Eclipse Proton Convolution Superposition Algorithm in Case of Inhomogeneities?

#### V. Flatten^1^, K. Baumann^1^, R. Engenhart-Cabillic^1^, K. Zink^2^

##### *^1^University Medical Center Giessen-Marburg, Department of Radiotherapy and Radiooncology, Marburg, Germany*, *^2^University of Applied Sciences Giessen, Institute of Medical Physics and Radiation Protection, Giessen, Germany*

**Objective**: Analytical convolution algorithms are known to be fast but more inaccurate in case of inhomogeneities. For proton therapy the uncertainties increase when traversing air, e.g. in the lung or the paranasal sinuses or high density material as implants.

**Material and Methods**: The Eclipse proton algorithm was commissioned for the Marburg Ion Therapy center with base data simulated in TOPAS. The Eclipse TPS (Varian) was chosen as a commercially distributed proton planning system. It was used to optimize treatment plans and to calculate the dose distribution. Additionally, the RT plan was converted into an input file for the GEANT4 based TOPAS Monte Carlo toolkit. There, the plan was recalculated and the dose distribution was compared with the Eclipse results. Plans were calculated for different phantom geometries consisting of water, air and bony structures and additionally for two patient cases.

**Results**: In case of homogenous phantoms, the proton range calculated in Eclipse and TOPAS was the same, but small discrepancies could be observed in the build-up region of the proton beam (within several millimeters). Differences in the dose distribution, but not in the proton range, occured in case of inhomogeneities. In Fig. 1 exemplary results for a phantom setup with two air cavities are shown. A decrease of dose within the Bragg peak behind the air cavities is clearly visible.

**Conclusion**: The analytical dose calculation algorithm used by the Eclipse TPS may over- or underestimate the dose in case of inhomogeneities, therefore Monte Carlo codes for dose calculation are desirable (poster will be presented by K.Zink, Germany).

### P 178: PMRT: A Platform to Modelize RadioTherapy Process and Outcomes for Decision Making, Data Mining and Personalized Medicine

#### J.M. Fontbonne^1^, D. Cussol^1^, J. Hommet^1^, E. Cagniot^2^, C. Fontbonne^1^, A. Chaikh^3^, T. Tessonnier^4^, J. Balosso^5,6^, J. Thariat^5,7^, J. Colin^8^

##### *^1^CNRS/IN2P3, LPC Caen, Caen, France*, *^2^GANIL/CIMAP, Cimap, Caen, France*, *^3^LPC Caen, Applications Médicales et Industrielles, Caen, France*, *^4^Centre François Baclesse, Medical Physics, Caen, France*, *^5^Centre François BACLESSE, Radiation Oncology, Caen, France*, *^6^University Grenoble Alpes, Medicine, Grenoble, France*, *^7^UNICAEN, Radiation Oncology, Caen, France*, *^8^UNICAEN, Physics, Caen, France*

Implementation of particle therapy beside the continuous evolution of photontherapy put into competition multiple types of beams and delivery methods, opening a huge combination of therapeutic possibilities. The optimal choice for specific patients cannot be based only on recommendations issued from prospective clinical studies. A critical need of fast decision-making methods emerges, relying on multidisciplinary modeling. The PMRT project launched in 2014 in Caen (France) aims to contribute to such capabilities, merging different key functions: data mining, treatment simulation, outcome modeling, treatment plan comparison, long term follow-up data and nationwide networking for patient recruitment. This needs both multiparametric input and multi-scale analysis taking careful account of uncertainties of the real treatments to convince health authorities.

Radiobiological models to estimate the tumor control probability and the normal tissue complication probability are major tools to compare radiotherapy plans. A step forward, the secondary cancer risk modeling will become important. However, renewed references are needed for toxicities to tune model parameters according to the present practices.

Thus the specific objectives of this project are: i) to asses and quantify the impact of uncertainties on available models; ii) to develop and qualify an original multiparametric model of tissues response to estimate the real benefit of patient up to quality of life outcomes; iii) to adjust and correlate the predictions to real clinical data gathered from multiple institutions (UNICANCER network). The ultimate goal is to predict the probabilistic outcome of different radiotherapy solutions to give clues to choose the most appropriated treatment for each patient.

### P 180: Assessing the Clinical Impact of TPS Dose Calculation for Proton PBS Treatment Using a Fast Monte Carlo Algorithm

#### S. Huang^1^, K. Souris^2^, S. Li^1^, M. Kang^1^, G. Janssens^3^, A. Lin^1^, E. Garver^1^, C. Ainsley^1^, Y. Xiao^1^, L. Lin^1^

##### *^1^University of Pennsylvania, Radiation Oncology, Philadelphia, USA*, *^2^Université catholique de Louvain,Center for Molecular Imaging and Experimental Radiotherapy, Brussels, Belgium*, *^3^Ion Beam Applications SA, Advanced Technology Group, Louvain-la-Neuve*, *Belgium*

The impact of our commercially-available TPS's analytical dose calculation (ADC) on proton PBS treatment plan quality was assessed using an open-source fast Monte Carlo code (FMC), MCsquare. First, FMC was commissioned and validated using water and tissue-mimicking phantom measurements as well as benchmarked with the general purpose Monte Carlo TOPAS for various representative patient cases. Both Monte Carlo codes dramatically improved the gamma-index analysis (7%/5mm) passing rate in the IROC lung phantom from below the passing threshold of 85% for ADC to over 93%. Figure 1 compares the 1-dimensional dose profiles through the center of PTV between simulation and film measurements. Furthermore, a total of 50 patients were investigated with 10 patients per site (liver, pelvis, brain, H&N and lung) by comparing dose distributions between ADC and FMC. Differences were evaluated using DVH indicators, estimates of tumor control probability (TCP) and a gamma-index analysis as shown in Figure 2. Generally, the impact of approximations in ADC on the plan quality increases with the degree of tissue heterogeneity. The ADC overestimated the target doses on average by up to 1.7% for different sites. The D95s were predicted within 6.5% of the corresponding FMC statistic, while the D02s and V90s were within 2.9%. Dose differences can result in large TCP differences for lung (<10.5%) and head and neck (<7.5%), with smaller differences seen for brain (<2.5%), pelvis and liver (<1.5%). The establishment of the FMC can facilitate patient plan reviews at any institution given its accuracy, speed and open source availability.

### P 181: Power-Law Distribution of Long Tail of Proton Dose Distribution

#### B. Jiang^1^, X. Zhang^1^, X.R. Zhu^1^

##### ^1^The University of Texas MD Anderson Cancer Center, Department of Radiation Physics, Houston, USA

In this paper, statistical methods and mathematical models are used to confirm that proton dose long tail phenomenon (halo) conforms to the power-law relationship. By analyzing 299 measured dose profiles using the least-squares linear regression, ranging from 72.5 to 221.8 Mev and 4.0–30.6 cm in depth, we find all profiles had significant power-law correlations with p <0.01 in the tail. The exponent parameter (slope) was -4.41±0.58, which 95% Confidence interval of -4.64 (low) and -4.19 (high). The square of the correlation coefficient was 0.98±0.021, indicated a strong linear correlation between log-transformed dose and distance. We set up a dual mechanism model: direct and indirect impact mechanisms. The direct mechanism means that the proximal dose deposition is mainly due to the direct impact of proton on the particle (e.g. A). The indirect mechanism means that the proton impact A via the impact other particles (e.g. B) near A, then B impact A. We find that the indirect mechanism leads to a power-law tail since probability of the indirect impacts is proportional to the distance: i.e. longer distance with larger indirect impact probability leads to the “rich (longer distance) get richer” kind of long tail phenomenon. By analyzing the experimental data, we observe that the power-law exponent increases with proton energy (Picture 1: “Power-law relationship between the lateral distance and lateral dose of multiple energy in the tail. Measure data points are shown as dots and best-fit curves as solid lines”; Picture 2: “Power-law exponent plotted against energies for profiles”).

### P 182: Monte Carlo Simulation of the Treatment Nozzle for the First Hyper Scan Proton Treatment System

#### M. Kang^1^, R. Cessac^2^, D. Pang^1^

##### *^1^Georgetown University Hospital, Radiation Medicine, Washington DC, USA*, *^2^Mevion Medical Systems, Enginerring Department, Littleton- MA, USA*

**Purpose:** To investigate the beam characteristics of the Hyper Scan nozzle for spot scanning treatment, the nozzle components have been modeled in a Monte Carlo (MC) platform to perform the end-to-end simulation.

**Method:** Nozzle components, including scanning magnets, strip ion chambers 1 and 2&3, range shifter (RS), adaptive aperture (AA) and exit window have been modeled in TOPAS MC software (Fig1). A single Gaussian proton source model has been used to simulate the beam from the cyclotron. The initial spot sigmas, proton energies and energy spread have been optimized to match the measured spot size and pristine Bragg peak for the highest energy of 227MeV. A constant scanning magnets gradient was used to scan the beam based on the unique feature of nozzle. To further validate the beam model, 5 lower energy beams, 203, 176, 146, 110 and 64MeV, have been benchmarked against with measurements (Fig2).

**Results and Conclusion:** The Hyper Scan nozzle has been successfully implemented in MC simulation platform. The simulation can faithfully reproduce the measured pristine Bragg peak with accuracy of 0.5mm range and spot sigma of 0.7mm, which provide a solid reference for the clinical application of the first Hyper Scanning System.

### P 183: The Advantage of Multi-Layer Shaping Using the Mevion Adaptive Aperture

#### A. Langenegger^1^, K. Huang^2^, S. Rosenthal^1^, J. Cooley^2^, R. Cessac^3^

##### *^1^Mevion Medical Systems, Product Manager, Littleton, USA*, *^2^Mevion Medical Systems, Engineering, Littleton, USA*, *^3^Mevion Medical Systems, Customer Support Services, Littleton, USA*

Mevion Medical System's S250i with HYPERSCAN is the world's most compact proton pencil beam scanning system. With its Adaptive Aperture, it is capable of delivering apertures specifically contoured to each energy layer.

RayStation from RaySearch Laboratories AB supports proton planning for the Mevion S250i with HYPERSCAN and has the capability of customizing apertures for any energy layer. Because the Adaptive Aperture is multi-layer aperture capable, there are significant dosimetric advantages in contouring dose on each energy layer in clinical planning.

As the Adaptive Aperture is fully automated, it requires no therapist interaction; there are dramatic savings in material fabrication costs and improved patient throughput. This feature improves treatments while making proton therapy less costly.

We will show examples of multi-layer dose shaping with the Adaptive Aperture.

### P 184: Dosimetric Analysis of Distal Esophageal Adenocarcinoma Treated by Intensity-Modulated Proton Therapy with Small Spots and Volumetric-Modulated Arc Therapy

#### C. Liu^1^, R.S. Bhangoo^1^, N.Y. Yu^1^, T.T. Sio^1^, J.B. Ashman^1^, W.G. Rule^1^, X. Ding^1^, M. Bues^1^, W. Liu^1^

##### ^1^Mayo Clinic, Radiation oncology, Phoenix, USA

**Purpose**: To investigate whether volumetric-modulated arc therapy (VMAT) is dosimetrically superior to small spot intensity-modulated proton therapy (ssIMPT) for distal esophageal adenocarcinoma (DEA).

**Methods and Materials**: 35 DEA patients were selected. Among them 19 patients received ssIMPT and the remaining 16 received VMAT. Both ssIMPT and VMAT plans were generated by delivering prescription doses to internal target volumes (ITV) on averaged 4D-CTs. The dose-volume-histograms (DVH) band method was used to quantify plan robustness. Software was developed to evaluate interplay effects with randomized starting phases of each field per fraction. DVH indices were compared using Wilcoxon rank sum test.

**Results**: Compared with VMAT, ssIMPT delivered significantly lower liver D_mean_ and V_30Gy[RBE]_, lung D_mean_ and V_5Gy[RBE]_, heart D_mean_ and V_20Gy[RBE]_, with comparable ITV dose coverage, homogeneity, and protection of other OARs. In terms of plan robustness, the IMPT plans were comparable for kidney V_18Gy[RBE]_, liver V_30Gy[RBE]_, stomach V_45Gy[RBE]_, lung D_mean_, V_5Gy[RBE]_ and V_20Gy[RBE]_, cord D_max_ and D_0.03cc_, liver D_mean_, and heart V_20Gy[RBE]_, but statistically worse for D_95%_, D_2cc_, D_5%_-D_95%_, heart D_mean_, V_30Gy[RBE]_ and V_40Gy[RBE]_. The ssIMPT plans still met the standard clinical requirements with interplay effects considered. Early clinical results demonstrate low acute toxicity and encouraging clinical and pathologic response for the DEA patients treated by ssIMPT.

**Conclusions**: ssIMPT protects heart, liver, and lung better than VMAT for DEA. ssIMPT plans are robust to uncertainties and interplay effects. Our results showed that ssIMPT is a good modality to treat DEA. Further study is warranted to compare the clinical outcome between two modalities.

### P 185: BED-based Spatio-Temporal Optimization of Non-Uniform Particle Therapy Treatments for Prostate Cancer

#### L. Manganaro^1,2,3^, A. Attili^3^, T. Bortfeld^2^, H. Paganetti^2^

##### *^1^University of Turin, Physics, Turin, Italy*, *^2^Massachusetts General Hospital & Harvard Medical School, Radiation Oncology, Boston, USA*, *^3^Istituto Nazionale di Fisica Nucleare, Physics, Turin, Italy*

The optimal fractionation scheme is always a compromise between increasing the total dose to achieve tumour control and increasing the number of fractions to spare the healthy tissues. In intensity modulated particle therapy (IMPT), the adoption of spatially and temporally heterogeneous dose distributions allows to decouple the fractionation scheme from the patient anatomy; hence, an hypofractionated schedule can be selectively created inside the tumour, while simultaneously exploiting the fractionation effect in the healthy tissues.

The aim of this contribution is to show the reproducibility of the method on a cohort of prostate patients, quantifying the dependencies of the achievable gain and addressing the issues related to setup errors and range uncertainty.

Non uniform IMPT plans were optimized for a cohort of 9 patients, and compared to standard-optimized treatments, with varying the radioresistance of the tumour. The benefit has been scored in terms of average reduction of the biologically effective dose (BED) in the entrance channel (femurs and skin). A robustness analysis has been carried out to quantify setup errors and range uncertainty.

An average 15.9% to 18% benefit has been shown in the clinically relevant range of tumour radiosensitivity. The non uniform plans result to be robust in terms of setup errors, while a systematic loss of target coverage has been detected as a consequence of range uncertainty. To compensate for this effect, a 4–5% reduction of the benefit is expected.

In conclusion, this technique represents a promising approach to radiation therapy which can significantly improve the treatment outcomes.

### P 186: Cost Effectiveness in Proton Therapy: The Incidence of Technological Evolution

#### A. Mazal^1^, P. Poortmans^2^

##### *^1^Institut Curie, Medical Physics, Paris, France*, *^2^Institut Curie, Radiation Oncology, Paris, France*

Proton therapy has a fast evolution, with slight variations in the market in this last year, partially related to the lack of cost-effectiveness data for a wide number of locations. While the interest of protons is based in their physical characteristics, providing proven or a potential gains in clinical results, the evolution of the cost is based on the development of a competing technology and their incidence in the workflow.

The time to evaluate the effectiveness is rather short, and extreme situations are seen in clinical practice, from rare cases where the in-silico results can make unethical to plan randomized trials (uveal melanoma, pediatrics, base of the skull) up to controversial cases including large volume of patients and/or risky approaches related to yet unsolved uncertainties. The photon solutions also evolve (e.g. MRI-linacs), making difficult to compare protons with a stable reference. New approaches including nanoparticles, micro-beams, very high dose rates and very high electron energies will play a role in the relative position of every tool in radiation therapy in the next years.

We evaluate the existing data on clinical build-up, studies on cost-effectiveness and the incidence of technological evolution on the decision process for the installation of a proton therapy facility in a given environment, including single room solutions, facilities in developing countries and research lines. We present data from our facility, where we improved efficiency as well as dosimetric and clinical optimization since 1991, and prospective data for proton and photon facilities in the world.

### P 187: Which Tissues Could Benefit from the Flash Effect Using Proton Beams?

#### A. Mazal^1^, L. De Marzi^1^, A. Patriarca^1^, C. Nauraye^1^, S. Heinrich^2^, R. Dendale^3^, P. Verrelle^3^, C. Fouillade^2^, P. Poortmans^3^, V. Favaudon^2^

##### *^1^Institut Curie, Medical Physics, Paris, France*, *^2^Institut Curie, Research Center, Orsay, France*, *^3^Institut Curie, Oncological Radiotherapy, Paris, France*

Recently our team demonstrated that very high dose rate irradiation given to a biological target to a high dose (e.g. 30 Gy in less than 100-500 msec) reduces selectively secondary effects in healthy tissues compared to « conventional dose-rate » irradiation. We have shown this in mice where lung fibrosis was reduced when treated with 4.5 MeV electrons beams^1^. While other teams are exploring this effect in other tissues (e.g. brain), we are currently testing whether this effect also exists when using proton beams. The biological mechanisms involved are under study.

In this work we show, through virtual simulation of dose distributions in patients that could be treated with proton beams under flash conditions, which are the clinical volumes of healthy tissue that could potentially benefit of such an approach, by simulating beam per beam and for a full treatment in a hypofractionated scheme. Calculations have been performed using a commercially available clinical treatment planning system (Dosisoft**©** and Varian Eclipse**©**), with a modified dose matrix using external software (e.g. Matlab**©**) before re-introduction in the planning system for interfacing and analysis. The results will be presented as isodoses and equivalent dose differences as well as histograms per critical organs.

^1^Vincent Favaudon et al. Ultrahigh dose-rate FLASH irradiation increases the differential response between normal and tumor tissue in mice. Sci Transl Med 6, 245ra93 (2014*)*

### P 188: Development of a 3D Monte Carlo-based Dose Verification Tool for Scanned Proton Therapy

#### S. Mizutani^1^, K. Hotta^2^, H. Baba^2^, T. Miyashita^3^, T. Yamaguchi^1^, T. Akimoto^2^

##### *^1^Sumitomo Heavy Industries- Ltd., Technology Research Center, Yokosuka-shi, Japan*, *^2^National Cancer Center, Research Center for Innovative Oncology, Kashiwa-shi, Japan*, *^3^Sumitomo Heavy Industries- Ltd., Quantum Equipment Devision, Niihama-shi, Japan*

**Purpose**: Dosimetric verification on geometric phantoms is a commonly performed as part of patient specific quality assurance, but these phantoms do not capture tissue heterogeneities in patients. However, in IMPT in particular, accounting for patient geometries is desired, which prompted us to develop a 3D dose verification tool that employs patient geometries from CT images using the Simplified Monte Carlo (SMC) method.

**Methods**: We built a program capable of reconstructing delivered dose distributions based on treatment log files and validated it against results from a treatment planning system (TPS) for 13 prostate plans treated at National Cancer Center Hospital East (NCCHE) in Japan.

**Results**: Figure 1 shows the dose distributions obtained from our tool and the commercial TPS for a prostate tumor. Both exhibit nearly identical profiles, except that a small difference can be observed in the distal dose fall-off region. This discrepancy arises because the clinical TPS system used at NCCHE employs an analytical dose calculation algorithm that cannot accurately capture the effects of tissue heterogeneity on the dose distribution. Calculation time was per field across 13 plans on CPU (Intel Core i7 3.4GHz). These results show that it is feasible to accurately predict the dose distribution in the actual patient's body within a reasonable amount of time.

**Conclusion**: We have developed MC-based dose verification software using treatment log files. This tool enables reasonably fast and accurate dose calculation on patient images and is easy to implement in clinical environments. We conclude that this tool has the potential to enhance treatment plan quality and improve treatment.

### P 189: Evaluating the Accuracy of Stopping Power Ratio Images Derived from Dual-Energy CT for Proton Dose Calculation

#### S. MossahebiI^1^, U. Langner^1^, H. Chen^1^, M. Zhu^1^, J. Eley^1^, K. Langen^1^, J. Polf^1^

##### *^1^University of Maryland School of Medicine, Maryland Proton Treatment Center- Radiation Oncology, Baltimore*, *USA*

**Purpose**: To investigate the accuracy of stopping power ratio (SPR) images created from electron density (ρ_e_) and effective atomic number (Z_eff_) images constructed from dual-energy CT (DECT) scans.

**Methods**: DECT scans were acquired, using a Siemens SOMOTON Definition Edge CT scanner, of 26 tissue surrogate plugs inserted, individually, into a high-density polyethylene phantom. Using ρ_e_ and Z_eff_ images constructed from the DECT scans (as explained in Figure 1), SPR images were created for each tissue plug. SPR values were extracted from a region of interest (ROI) in the tissue plugs from the SPR images. Next, SPR values were determined from measurements of the water equivalent thickness (WET) for each plug using a multi-layer ionization chamber (MLIC), and compared to the values extracted from SPR images.

**Results**:The DECT derived SPR values agreed with measured values to within 1.5% for all tissue surrogates except brain, muscle, liver (< 2.5% each) and lung (∼16%) as shown in Figure 2. Overall the Hünemohr et al method of calculating SPR showed slightly better (∼0.4%) agreement than that of Yang et al.

**Conclusion**: The method for creating SPR images from DECT scans proved to be accurate for determining the SPR for the standard tissue surrogate materials, except lung, with an uncertainty of less than 2.5%. Based on these preliminary results we believe the use of DECT derived SPR images in the treatment planning process could help to reduce dose calculation uncertainty for regions outside of the lung.

### P 190: Hitachi PROBEAT-V Microbeam Validation Experience with Eclipse Proton Convolution Superposition Algorithm

#### W. Myers^1^, V. Moskvin^1^, L. Zhao^1^

##### ^1^St. Jude Children's Research Hospital, Radiation Oncology, Memphis, USA

**Purpose**: The Hitachi PROBEAT-V Proton Therapy system at our institution has a Microbeam option with a smaller spot size (4.2 and 1.4 mm sigma at 69.4 and 221 MeV at isocenter) than our standard beam (5.3 and 2.2 sigma). Here we describe the Microbeam validation experience using the Proton Convolution Superposition (PCS) algorithm implemented in Eclipse TPS (V13.7.15).

**Methods**: Measured and calculated doses were compared for 55 rectilinear fields with ranges between 6 and 20 cm, modulations between 1 and 6 cm and field sizes between 0.5 and 8 cm. R90s, lateral profiles and 12 patient fields were also validated. The 2D MatriXXPT array, Advanced Markus chamber (model TN34045), and Pinpoint chamber (model TN31014) were used.

**Results**: The results show differences up to +/-6% between measured and calculated doses. A range dependency and a field size dependency at low energies were determined. The depth dose norm calibration factor table (DDNCFT) was adjusted to better align the measured and calculated doses. Applying the DDNCFT brought these differences to within +/-1.7%. Detector volume effects were apparent for fields with modulation or field size smaller than 1 cm. Detector choice was validated by comparing results from the three detectors. Range, profile and patient measurements showed excellent agreement with TPS results.

**Conclusions**: Extensive validation of the PCS algorithm demonstrates the need for the DDNCFT when commissioning beams with submillimeter spot sizes. This correction method is sufficient to bring the calculated doses in close agreement with the measured doses for the Microbeam.

### P 192: Real-time Range Verification during Ion Therapy Utilizing Microbubble Contrast Agents for Ultrasound Imaging – Preliminary Feasibility

#### S. Patch^1^, E. Nikolau^1^, B. Mustapha^2^, J. Nolen^2^

##### *^1^UW-Milwaukee, Physics, Milwaukee, USA*, *^2^Argonne National Laboratory, Division of Physics, Lemont, USA*

Microbubble contrast agents for ultrasound imaging may enable real-time range verification during both pulsed and CW ion delivery, whereas thermoacoustics requires pulsed delivery. Gas-filled microbubbles reflect ultrasound pulses, enhance signal in vasculature, and visualize perfusion in ultrasound images. If stopping ions can destroy microbubbles during treatment, then contrast-enhancement in ultrasound images will decrease in high LET regions but remain unchanged outside.

We present experimental validation that braking helium ions can destroy microbubbles in undiluted solution.

A capillary phantom was created by wrapping 780-micron OD/670-micron ID THV tubing around a straw with a 1 cm diameter. Undiluted Definity microbubbles were injected into the tubing and allowed to aggregate at the top. The phantom was placed in a water-filled container adjacent to a 60-micron entrance window so that the microbubbles were bombarded.

14 enA was applied for 180 seconds in a beam focused to FWHM of 3.9 mm (horizontal) and 6.9 mm (vertical). Beam energy was 15 MeV/u, with range of 2.4 mm in water.

Most destruction occurred within the second turn of the tubing (see figure). Turns 4-6 were unperturbed and the 55-micron tube walls remained water-tight throughout. Further work is needed to confirm reduction of ultrasound contrast of microbubble solutions diluted to *in vivo* concentrations during proton therapy. Finally, microbubble cavitation is capable of destroying capillaries, so rigorous animal studies should precede clinical trials. Bubble destruction was greatest in the second turn (yellow arrow); turns were 4-6 unperturbed.

*Supported by Lantheus under MTA #2017004 and the US Department of Energy under Contract #DE-AC02-06CH11357.

### P 193: Dose Build-Up Effects in Proton Bragg Curves

#### T. Pfuhl^1,2^, F. Horst^1,3^, C. Schuy^1^, J. Stroth^4,5^, U. Weber^1^

##### *^1^GSI Helmholtzzentrum für Schwerionenforschung, Biophysics, 64291 Darmstadt, Germany*, *^2^Goethe University Frankfurt, Biophysics, 60438 Frankfurt, Germany*, *^3^Technische Hochschule Mittelhessen, Institute for medical physics and radiation protection, 35390 Gießen, Germany*, *^4^GSI Helmholtzzentrum für Schwerionenforschung, Hades detector, 64291 Darmstadt, Germany*, *^5^Goethe University Frankfurt, Nuclear physics, 60438 Frankfurt, Germany*

The precise knowledge of the dose deposition of ions in matter is essential for an accurate treatment planning for cancer patients. However, the build-up effects in Bragg curves, especially the one induced by target fragments, are not completely quantified yet. Therefore, in this study the build-up effects were investigated experimentally and theoretically for 220 MeV protons.

The δ-electron build-up effect takes place in the first few millimeters of the target until an equilibrium state of the forwards scattered δ-electrons is reached. The target fragment build-up effect covers the first centimeters of the entrance channel and is a result of target fragments created in inelastic interactions of the beam particles with the target atoms. These fragments have a low kinetic energy and partly high atomic numbers compared to the beam protons which results in increased RBE values. However, the relevant production cross sections for ion beam therapy still have large uncertainties. As the direct measurement of target fragments is extremely difficult the target fragment production is examined indirectly by investigating the target fragment build-up effect in proton Bragg curves.

Very precise Bragg curve measurements with the focus set on the build-up regions were performed at the proton therapy center in Trento, Italy. The results are displayed in figure 1. Extensive FLUKA Monte Carlo simulations were carried out in preparation for the experiment. A comparison of the experimental results with calculated Bragg curves enables the assessment of the precision of the nuclear models used.

### P 194: Microscopic Uncertainties at the Bragg Peak: A Microdosimetric Approach

#### G. Santa Cruz^1^

##### ^1^NATIONAL ATOMIC ENERGY COMMISSION, Gerencia Investigación y Desarrollo en Aplicaciones Nucleares a la Salud, Buenos Aires, Argentina

This work explores the consequences of basic microdosimetric quantities that predict a remarkable reduction of particle events occurring in the last millimeters of a Pristine Bragg Peak (PBP) as a consequence of the important changes in the ionization density that take place in the last micrometers of the proton trajectories. Considering a 200 MeV proton beam in water and simulating particle tracks traversing spheres of 1 micrometer in diameter (the characteristic volume where pairs of Double Strand Breaks combine to form chromosome aberrations) the two first moments of the lineal energy and specific energy probability densities were calculated, as a function of depth. The mean number of events, the standard deviation of the energy distribution and the Poisson probability of occurrence of zero events or 2 or more events were calculated. These results were used to evaluate the dependence of these quantities for a spread-out Bragg Peak (SOBP), covering about 5 cm with a fairly uniform dose of 1 Gy.

Large variations in the mean number of events occur, sharply decreasing from about 10 to less than 0.1 in the last few millimeters of the distal falloff, meaning that more than 90% of the sensitive sites experience no dose deposition at all. Specific energy uncertainties are very significant, achieving relative standard deviations larger than 100% even for a peak dose of 8 Gy. The probability of 2 or more independent events falls to almost zero, precluding the possibility of a quadratic dependence of the effect at the different peak doses.

### P 195: Is the Proton Monte Carlo Dose Calculation Engine of Raystation Suitable for Small Animal Research?

#### E. Traneus^1^, R. Nilsson^1^

##### ^1^RaySearch Laboratories AB, Research, Stockholm, Sweden

Purpose: To assess if the Monte Carlo dose calculation algorithm and proton beam model in the RayStation treatment planning system is applicable to small animal research with sub-millimeter voxel size and proton range down to a few millimeters.

Methods: A small animal version of the system permitting voxel sizes down to 0.05 mm was used to calculate depth and lateral dose and LET profiles in water. This were compared to calculations by the PHITS v2.81 Monte Carlo code. Open field lateral integrated depth doses (mono-energetic 20 - 80 MeV beam in 0.1 mm^3^ voxels) and realistic collimated fields (3x3 – 1x1 mm^2^) were investigated. Collimation was achieved through a model of a small animal irradiator extension (5 cm Lexan energy absorber and 1 cm brass aperture) attached to a typical IBA dedicated nozzle with lowest commissioned energy of 70 MeV and used with a single central spot of energies varying between 70 – 140 MeV.

Results: A technical interpolation issue, distorting the depth dose shape below about 10 MeV remaining energy, was found and resolved. The open IDD profiles are in very good agreement over the investigated energy range down to the smallest voxel size considered. Collimated fields showed similar agreement. First results indicate that also dose and track weighted LET is well reproduced by the RayStation Monte Carlo code.

Conclusion: It is feasible to apply the current Monte Carlo proton dose engine for small animal irradiator based research. Validation of calculations versus measurements is a necessary next step and this is being planned.

### P 196: Comparison Raystation vs. TOPAS of Monte Carlo transport in a Magnetic Field

#### J. Hartman^1^, E.W. Korevaar^2^, E. Traneus^3^, A.C. Knopf^2^, J.J.W. Lagendijk^1^, B.W. Raaymakers^1^

##### *^1^University Medical Center Utrecht, Department of Radiotherapy, Utrecht, Netherlands*, *^2^University Medical Center Groningen, Faculty of Medical Sciences, Groningen, Netherlands*, *^3^Raysearch Laboratories, Sales Particle Therapy, Stockholm, Sweden*

**Purpose**: The purpose of this study is the comparison of RayStation 6R vs. TOPAS (RS-TS) for proton Monte Carlo dose calculation in the presence of a magnetic field for simple field and optimized IMPT fields.

**Methods**: In TOPAS a clinical beam model was developed that was implemented in Raystation 6R, using the auto-modeling system. A single spot for three different nominal proton energies (72.5, 119.9, and 221.8 MeV) was simulated and scored in a homogeneous water phantom without magnetic field and with a homogeneous magnetic field of 1.5 Tesla perpendicular to the beam direction. The spot profiles, integrated depth dose (IDD) and magnetic deflection were compared.

**Results**: The spot and IDD profiles are in good agreement without the presence of a magnetic field (fig. 1a), with minor differences in the entry dose. The spot profiles at phantom entrance also show good agreement (fig 1b). The magnetic deflection of the spot in RS is similar to TOPAS (fig. 2a). There are some dose differences in the entry dose and the range, which is also visible in the IDD profile (fig. 2b).

**Conclusion**: For a single spot RS is in good agreement with TOPAS, both with and without a magnetic field. The spot profiles, IDD profiles and magnetic proton deflection are similar. The observed small difference in entry dose at higher energy is under further investigation. The validated RS model could be used for IMPT studies inside a magnetic field, such as in feasibility studies on MR-guided proton therapy.

### P 197: Investigation of the Optimal Physical Parameters for Precise Proton Irradiation of Small Animals

#### M. Vanstalle^1^, J. Constanzo^1^, C. Finck^1^

##### ^1^Université de Strasbourg- IPHC, Department of Radiobiology- Hadrontherapy and Molecular Imaging, Strasbourg, France

The emergence of *in vivo* preclinical proton irradiation platforms dedicated to radiobiological studies drives the necessity to develop advanced small animal models, mimicking clinical therapy conditions. In this context, accurate irradiations of small target volumes (< 1cm) are conditioned by the physical properties of the proton beam.

This work aims to determine these optimal physical parameters (energy, straggling) using Monte Carlo simulations carried out with Geant4. Simulated spread-out Bragg peaks obtained with different energies of interest (between 25 and 200 MeV) and different energy stragglings will be compared. The associated dose volume histograms will be presented to quantify the homogeneity of the dose delivered in the target volume. Figure 1 shows an example of DVH obtained with different energies to target a 2-mm volume located at 4-mm depth. It will be demonstrated that low energy proton beams (E<50 MeV) with low associated energy spread are more suitable than clinical beam to deliver accurate dose in target volumes smaller than 5 mm (Figure 1: Comparison of the DVH of a 2-mm target volume obtained with 25 MeV, 30 MeV, 50 MeV, 68 MeV, 160 MeV and 200 MeV proton beam. The black dashed lines indicate the accepted dose tolerance around a 1 Gy prescribed dose).

### P 198: Investigation of the Optimal Physical Parameters for Precise Proton Irradiation of Small Animals

#### Y. Xie^1^, S. Li^1^, C. Ainsley^1^, L. Yin^1^, W. Zou^1^, E. Garver^1^, S. O'Reilly^1^, J. Marcel^1^, L. Dong^1^, B.K. Teo^1^

##### ^1^University of Pennsylvania, Department of Radiation Oncology, Philadelphia, USA

**Purpose**: The steps for commissioning a DECT scanner for use in proton therapy are described: (1) determination of optimum scan and image reconstruction parameters; (2) validation of calculated relative electron density (ρ_e_) and effective atomic number (Zeff) images; (3) validation of DECT calculated proton stopping power (SPR) with phantom irradiation.

**Methods and Materials**: Tissue surrogates were scanned on a Siemens Somatom Definition Edge DECT using 120kVp single energy CT (SECT), TwinBeam DECT (TBCT) and 80/140 kVp dual-spiral CT (DECT) modes. Images were reconstructed utilizing iterative reconstruction (ADMIRE) and metal artifact reduction (iMAR). The effect of beam hardening on Hounsfield units (HUs), ρ_e_ and Zeff were evaluated by varying the phantom size. Proton plans were delivered through an anthropomorphic phantom. Proton residual range was recorded using gafchromic EBT3 film placed parallel to the beam direction and compared to planning system calculated range utilizing SECT and DECT calibration.

**Results**: ADMIRE reduces image noise without affecting HUs while iMAR improved HU accuracy but did not completely remove artifacts surrounding metal objects. The Q34bhc kernel which incorporates beam hardening correction was superior to the Q30 kernel, exhibiting the smallest variation of ρ_e_ and Zeff with phantom-size for both TBCT and DECT modes. DECT generated more accurate ρ_e_ and Zeff maps than TBCT. In SPR prediction, DECT had better agreement with film measurement than the SECT calibration.

**Conclusion**: During DECT commissioning, the choice of image reconstruction settings needs to be optimized and the accuracy of DECT derived ρ_e_, Zeff and SPR should be evaluated.

### P 199: Minimization of Systematic Impact of Noise in Dual-Energy CT Based Stopping-Power-Ratio Estimation

#### H.C. Lee^1^, B. Li^2^, X. Duan^3^, L. Zhou^2^, X. Jin^1,^ M. Yang^1^

##### ^1^University of Texas Southwestern Medical Center, Radiation Oncology, Dallas, USA, ^2^Southern Medical University, Biomedical Engineering, Guangzhou, China, ^3^University of Texas Southwestern Medical Center, Radiology, Dallas, USA

There have been concerns with the robustness of DECT-based algorithms to noise in estimating ion stopping-power-ratio (SPR). Several studies have demonstrated that the impact of noise can be reduced, but most of them lacked an analytical approach. This study identifies how noise interacts with DECT-based algorithms and present a way to minimize the mean shift of SPR (ΔSPR) induced by noise. For several simple model-based algorithms, ΔSPR was negligible with low noise (<2%) but abruptly increased with increase in noise level. We found that 1) the nonlinearity in HU to Z conversion and 2) the errors in separating soft and bone tissues in Z to I conversion were the two main causes amplifying ΔSPR in DECT-based algorithms. We resolved these issues by adopting Saito's Z to I conversion (Saito and Sagara, 2017) to the Li's model (Li* et al*., 2017) with a modified threshold, thereby making the HU to SPR conversion fully linear with a better separation of soft and bone tissues. Among the four methods compared – the original Li's method (Li+Yang)(Li* et al*., 2017), Bourque's method (Bourque) (Bourque* et al*., 2014), Li's model with Saito's Z to I conversion (Li+Saito (Original)) (Saito and Sagara, 2017), and Li+Saito with a modified threshold (Li+Saito (Proposed)) – our proposed adaptation was confirmed to be very effective in minimizing ΔSPR caused by noise (Figure 1: ΔSPR with 2% noise in heatmap using (a) Li+Yang and (b) Li+Saito (Proposed); Figure 2: ΔSPR of ICRU44 reference human tissues with (a) 2% and (b) 5% noise).

### P 200: Off-Line Analysis of CTV Dose Coverage Using Daily In-Room CT Images for Bone and Soft Tissue Tumors by Scanning CIRT

#### S. Yoshino^1^, Y. Tokiya^1^, Y. Kusano^2^, S. Minohara^2^, Y. Okuyama^3^, M. Tago^4^, K. Yoshikawa^3^

##### *^1^Division of Radiological Technology, Kanagawa Cancer Center, Yokohama, Japan*, *^2^Section of Medical Physics and Engineering, Kanagawa Cancer Center, Yokohama, Japan*, *^3^Graduate Division of Health Sciences, Komazawa University, Tokyo, Japan*, *^4^Department of Radiology, Teikyo University Mizonokuchi Hospital, Kawasaki, Japan*

**Purpose**: Each treatment room in our facility i-ROCK has the rail-on in-room CT (Aquillion LB; TOSHIBA Medical Systems) in addition with orthogonal X-ray FPD imaging system. Routinely the X-ray images are used for patient positioning, and optionally the CT images are taken for verification of tumor and OAR position.The purpose of this study is to evaluate the target volume coverage for bone and soft tissue by off-line analysis.

**Method and Materials**: In-room CT images were taken at each fractionation for five patients with bone and soft tissue tumors (16 fractions at our current protocol). The initial treatment planning (reference plan) has sufficient PTV margin (≥ 5 mm) to achieve the criteria of CTV V95% ≥ 95%, V90% ≥ 98%.

1. Acquiring in-room CT images after2D/3D bony anatomy alignment at each fraction.

2. At off-line, CT images export to MIM maestro (MIM software Inc.).

3. Reference CTV contour is overlaid on the in-room CTs, and the CTV contour were re-drawn if necessary.

4. Calculate dose distribution with condition of initial beam-parameters of irradiation.

5. Analyze the dose distribution of CTV volume.

**Results**: The tumor volume changed to 101.1 ± 4.4 % during treatment course for 4 weeks. The dose coverage of CTV V95% and V90% for 5 patients were 99.14 % and 99.58 % at initial plan, and were 98.18 ± 1.38 % and 99.04 ± 0.89 % at treatment. There were no significant difference between the initial plan and the irradiated plan.

**Conclusion**: Sufficient dose coverage of CTV was achieved in five patients with bone and soft tissue tumors.

### P 201: Performance of a Semi-Analytical Dose Engine Designed for Spot Scanning Proton Therapy with Small Spot Sizes

#### J. Younkin^1^, D. Hernandez Morales^1^, J. Shan^2^, M. Bues^1^, J. Lentz^1^, S. Schild^1^, J. Stoker^1^, X. Ding^1^, J. Shen^1^, W. Liu^1^

##### *^1^Mayo Clinic Arizona, Radiation Oncology, Phoenix- AZ, USA*, *^2^Arizona State University, Biomedical Informatics, Tempe- AZ, USA*

**Purpose**: To study the clinical performance of a semi-analytical dose engine developed for spot scanning proton delivery systems that feature small spot sizes.

**Methods**: The dose engine used a ray-tracing pencil beam algorithm with three lateral Gaussian components. It was commissioned using Monte Carlo (MC) data generated by an experimentally well-benchmarked Geant4 code and then fine-tuned using point dose measurements with various combinations of energy layers, field sizes, depths, and SOBPs. The dose engine used water-equivalent distance in both the beam direction and the lateral direction to account for inhomogeneity. Ten patients representing different disease sites were randomly selected for validation. Comparisons were done in water with data calculated from a fast MC code and with ionization chamber array measurements for patient-specific QA (including point doses, dose profiles, 2D-3D, and 3D-3D gamma analysis) and in patient geometries with MC data (including dose volume histogram indices and 3D-3D gamma analysis).

**Results**: In-water gamma passing rates using 3%/3mm criteria for these ten patients were greater than 97% when compared to MC and greater than 95% when compared to dose plane measurements. Excellent agreement was observed for dose profiles and point dose measurements in water. Good agreement between the semi-analytical engine and the MC code was also observed in patient geometries. This dose engine has been successfully used at our institution as a fast, independent second check for over two years.

**Conclusion**: A ray-tracing dose engine with three lateral Gaussian components can accurately calculate dose distributions for our proton delivery system.

### P 202: Imagery and Positioning Technology Management for Ophthalmic Treatment in Proton Therapy

#### J. Assuli^1^, S. Meyroneinc^2^

##### *^1^Institut Curie - CPO, Technical - Engineering, Orsay, France*, *^2^Institut Curie - CPO, Technical, Orsay, France*

Ophthalmic treatment has always required a specific attention. As a UFO among the other localizations, it needs suitable solutions that the industry mostly doesn't support. After years of handcrafting, it seems appropriate to take a look at the long-term management of such technologies.

Here we will focus on the imagery and positioning systems and more specifically on the following topics:

- Technology Obsolescence And Switching Time
- Time Between Maintenance And Development
- Innovation Management
- Information Management
- Dealing With IT Service
- Working With Big Industries And Close Partners
- When Technology Is Older Than The Manager

Some examples from concrete projects and situations that appeared in our own ophthalmic treatment room will illustrate these points.

Thanks to 25 years of project feedback from the Curie Institute and the new tools and theory available for the technology management, we will try to highlight some good practices within the ophthalmic field.

### P 203: Proton Range Verification Using Radiochromic Film in a Head Phantom

#### G. Coutrakon^1^

##### ^1^Northern Illinois University, Physics, DeKalb- IL, USA

Proton range uncertainties have long been a concern for optimal proton dose delivery. A central question is, how much range error is attributable to the X-ray CT imaging modality in proton treatment planning? To address this question, a series of proton beam irradiations with film dosimeters embedded in a head phantom have been performed with X-ray CT, dual energy X-ray CT and proton CT imaging. Beam enters perpendicular to the stack of 36 films each having 0.35 mm water equivalent thickness. The beam in treatment planning is designed to stop at a calculated depth in the film stack. The head phantom is then irradiated with the planned beam and we measure the deviation of the measured stopping depth by comparing the dose distributions from treatment planning with measurements on the 36 films. We report on the range errors using these three imaging modalities in the commercial treatment planning systems, Xio and Ray Station, in use at Northwestern Medicine Chicago Proton Center.

### P 204: A Fast Monolithic System for Proton Imaging

#### F. Dejongh^1^, E. DeJongh^1^, V. Rykalin^1^, J. Welsh^2^, M. Pankuch^3^, N. Karonis^4,5,6^, C. Ordonez^6^, K. Duffin^4^, J. Winans^6^, G. Coutrakon^7^

##### *^1^ProtonVDA Inc, Physics, Naperville, USA*, *^2^Stritch School of Medicine Loyola University - Chicago, Radiation Oncology, Maywood, USA*, *^3^Northwestern Medicine Chicago Proton Center, Physics, Warrenville, USA*, *^4^Northern Illinois University, Computer Science, DeKalb, USA*, *^5^Argonne National Laboratory, Mathematics and Computer Science Division, Argonne, USA*, *^6^Northern Illinois University, Center for Research Computing and Data, DeKalb, USA*, *^7^Northern Illinois University, Physics, DeKalb, USA*

**Purpose**: Proton radiography enables proton range verification in addition to anatomical alignment verification currently obtained with X-ray radiography. Design specifications require that a clinical system be simple, lightweight, easily scaled to large field sizes, operate at high speed to maximize patient throughput, and expose the patient to the minimum possible radiation dose for a given resolution. We are developing a system to produce images of proton stopping power by tracking individual protons before and after the patient and measuring the proton residual range after traversing the patient. To achieve optimal spatial resolution, an image reconstruction algorithm must fully exploit the individual three-dimensional proton position information.

**Methodology**: We have constructed a fully functional prototype of a proton radiography system fully exploiting proton path information. A model of the system in a treatment room is shown in Fig. 1.

**Results**: Protons typically scatter by 4 mm after 20 cm of water. Our simulations, (Fig. 2), demonstrate path reconstruction of individual protons to better than 1 mm. Our iterative algorithm successfully produces images with 1 mm sharpness.

**Conclusions**: A proton radiography system optimizing image sharpness and dose to the patient will individually track protons before and after the patient. Commissioning and calibration of our system is in progress (Figure 1: Model of our proton radiography system in a proton treatment room; Figure 2: Simulated proton radiographs from X-ray CT scans of a human patient. Left: Blurred image from multiple Coulomb scattering of protons. Right: With iterative reconstruction using individual proton paths).

### P 206: Lens-Refocused Proton Radiography for Proton Beam Guidance

#### M. Freeman^1^, M. Espy^2^, J. Goett^1^, P. Magnelind^3^, F. Mariam^4^, F. Merrill^5^, R. Sidebottom^6^, F. Trouw^1^, D. Tupa^4^, C. Wilde^1^

##### *^1^Los Alamos National Laboratory, P-23: Neutron Science and Technology, Los Alamos- NM, USA*, *^2^Los Alamos National Laboratory, AET-6: Applied Engineering & Technology, Los Alamos- NM, USA*, *^3^Los Alamos National Laboratory, P-21: Applied Modern Physics, Los Alamos- NM, USA*, *^4^Los Alamos National Laboratory, P-25: Subatomic Physics, Los Alamos- NM, USA*, *^5^Los Alamos National Laboratory, P-DO: Physics, Los Alamos- NM, USA*, *^6^Occidental College, Occidental College, Los Angeles- CA, USA*

Proton radiography could potentially guide proton therapy, enabling real-time feedback on patient position, and dosimetric distribution. However, more familiar proton radiographic techniques are unsuitable for this goal. Images created from proton scatter radiography would be intrinsically low-contrast, and low-resolution, and the human body is too radiographically thick to implement range-straggling proton radiography. However, a method developed at LANL using magnetic-lens refocusing, could visualize the human body with high contrast, 200-μm spatial resolution, and in real time from a beam's-eye-view perspective. This method provides instantaneous, full coverage of the entire field of view. By refocusing the beam, an image is re-created downstream, with a first-order correction to chromatic lens aberrations. Furthermore, the system is designed to reject protons scattered beyond a certain acceptance angle, a property that allows for the object contrast to be selectively specified, enabling visualization of the human body in high contrast. As the technology behind commercial proton accelerators advances, higher energies approaching the 800-MeV proton energy utilized for this work come closer to reality. In addition to enabling lens-refocused proton radiography, this higher energy proton tightly constrains the lateral dose-deposition profile to a full-width half max of just 5.2 mm at a tissue depth of 33.4 cm, which would enable volumetrically-modulated adaptive therapy, potentially realizing higher gains in treatment accuracy. Lens-refocused proton radiography offers the ability to image, or, target and deliver a treatment pulse in a fraction of the time for a single breath or a single heartbeat, enabling proton beam adaptive therapy.

### P 207: An Approach for Optimizing Prompt Gamma Based Range Estimation in Proton Therapy Using Cramér-Rao Theory

#### E. Lens^1^, E. Tolboom^1^, D. Schaart^2^

##### *^1^Delft University of Technology, Radiation Science and Technology, Delft, Netherlands*, *^2^Holland Proton Therapy Centre, HollandPTC, Delft, Netherlands*

Various methods for in vivo range estimation during proton therapy, based on prompt gamma (PG) photon measurements have been proposed. However, optimizing the method of detection can be cumbersome. Here, we investigate the feasibility of using the Cramér-Rao lower bound (CRLB) for this purpose.

Our CRLB provides the smallest possible variance on the proton range obtained from any unbiased estimator, given a statistical model of the observations. The considered observables are the position, energy, and time of detection of the PG photons.

We used the TOPAS Monte Carlo code to simulate a clinical proton pencil beam (N=1·109 –4·109) targeting a cylindrical, soft-tissue equivalent phantom. PG photons were scored on a cylindrical surface (i.e. detector; ø=40 cm) coaxially surrounding the phantom. PG emission profiles corresponding to different proton ranges were generated by changing the initial proton energy. The detected photons were selected and tallied based on location, angle, energy and time of incidence on the detector. From the resulting signals, we calculated the CRLB as a function of the instrumental spatial, energy, and time resolution.

We found comparable CRLB values for range estimation based on (only) spatial, time, and energy information, if the detection parameters were optimized for each case. The figure illustrates this optimization when using PG energy. The table lists the optimal detection parameters for all (combinations of) observables. Combined detection of spatial and energy information yielded the lowest CRLB values.

We conclude that the CRLB is a promising tool for the optimization of PG based range estimation.

### P 208: Neutron Detection for Real-Time Range Verification in Proton Therapy – A Monte Carlo Feasibility Study

#### I. Meric^1^, K. Ytre-Hauge^2^

##### *^1^Western Norway University of Applied Sciences, Department of Electrical Engineering, Bergen, Norway*, *^2^University of Bergen, Department of Physics and Technology, Bergen, Norway*

In cancer treatment, growing numbers of patients are being treated through proton therapy. This is mainly because protons have a finite range in matter that in turn depends on the initial energy of the particle and the physical properties of the target material. However, it is well-known that real-time range verification techniques are needed in order to take full advantage of the finite particle range. In this work, we propose a novel and previously unexplored technique based on detecting and imaging secondary neutrons produced in nuclear interactions during proton therapy. The proposed set-up is similar to a proton recoil telescope arrangement consisting of a hydrogen-rich converter in addition to two tracker planes. Neutrons incident on the converter material produce recoiling protons through elastic collisions with the hydrogen nuclei whereas the tracker planes are used to track these recoiling protons from (n,p) reactions (figure 1). Here, we report and discuss the results of the initial feasibility study carried out using FLUKA Monte Carlo (MC) simulations at different proton beam energies and intensities in homogeneous water phantoms. Implementation of a custom particle tracking routine enabled imaging of the neutron production distribution along the beam axis through an iterative MC acceptance-rejection sampling scheme, utilizing the information available from the tracking routine through back-projection of the recorded recoil proton vectors. The results indicate that the reconstructed neutron production distribution is dependent on, and can be correlated to the particle range (figure 2).

### P 209: Two-step thermoacoustic Range Verification Enabled by MC Treatment Plans and Synchrocyclotrons: A Simulation Study Including Additive White Noise

#### S. Patch^1^, T. Zhao^2^

##### *^1^UW-Milwaukee, Physics, Milwaukee, USA*, *^2^Washington University St Louis, Radiation Oncology, St Louis, USA*

The purpose of this work is to examine robustness of thermoacoustic range verification with respect to beam direction as applied to prostate cancer.

Thermoacoustic emissions are bandlimited below 150 kHz, corresponding to wavelengths exceeding 10 mm. Thermoacoustic imaging resolution is limited to one-half wavelength. To improve accuracy beyond 5 mm and robustness to additive white noise, a priori information was utilized.

TOPAS Monte Carlo simulations modeled energy density deposited by oblique and horizontal pencil beams. For each beam angle, proton range was translated in ±5 mm water equivalent increments. The energy density was computed on the same 1 mm mesh as the planning CT.

Three-dimensional k-wave simulations propagated thermoacoustic pulses throughout the planning CT volume assuming a piecewise constant map of propagation speeds. Pulses were recorded at four virtual transducer locations on a 5-cm side-fire transrectal array. One-way beamforming of thermoacoustic emissions yielded initial range estimates.

Thermoacoustic emissions were compared to those simulated from a synthetic control beam with range nearest the beamformed estimate (Fig. 1). Time shifts between the measured and control thermoacoustic emissions were estimated by applying the Fourier shift theorem. Stepping from the known range of the control beam by a distance determined from the time shifts yielded the final range estimate.

Final estimates for oblique and lateral beams were accurate to within 1.0 and 1.6 mm, respectively. Average errors of final range estimates for oblique beams from data with SNR = 0 dB were no greater than 2.0 mm.

### P 210: Dual-Energy Gamma Computed Tomography for Adaptive Proton Therapy: A Feasible Study

#### S. Penfold^1,2^, J. Zhu^1^

##### *^1^University of Adelaide, Department of Physics, Adelaide, Australia*, *^2^Royal Adelaide Hospital, Department of Medical Physics, Adelaide, Australia*

Online adaptive particle therapy may prove to be an important tool in reducing dose delivery uncertainty in certain tumor sites. This has the potential to reduce the margins needed for particle therapy and thereby increase healthy tissue sparing. Current in-room cone beam computed tomography (CBCT) systems consist of an X-ray tube and an integrating flat panel detector. Due to the uncertainties associated with converting CBCT Hounsfield Units (HUs) to relative stopping powers (RSPs) with this imaging solution, deformable image registration of the planning CT to the CBCT is required for dose of the day calculations. This is a computationally intensive process and brings with it uncertainties in soft tissue deformation due to the limited contrast resolution achievable with existing CBCT systems. A more favorable approach would involve an in-room imaging solution that provides accurate RSP information on which dose could be directly calculated.

In this work we propose a dual-energy CBCT (DECBCT) imaging system consisting of a radioactive Eu-155 source paired with a pixelated single photon counting solid state detector. The properties of Eu-155 that make it attractive for this task will be summarized. A simulation study comparing the proposed imaging system with a conventional CBCT imaging system was performed with the Geant4 Monte Carlo toolkit. Improved RSP accuracy and soft tissue contrast for a head phantom was found with the novel imaging concept.

### P 211: A Semi-Empirical Model for PET-Based Range Verification of Proton Therapy Treatments

#### A. Pourmoghaddas^1^, W. Yao^1^, V. Moskvin^1^

##### ^1^St. Jude Children's Research Hospital, Department of Radiation Oncology, Memphis, USA

**Purpose**: PET imaging of activated positron-emitters after proton therapy treatments has been used as an in-vivo proton range verification method. Estimation of proton range from PET is challenging since the activation yield doesn't directly correlate to proton range. In this work, a semi-empirical model was developed to calculate the proton range using fundamental relationships between the proton Bragg-peak, fluence and activation over depth. This methodology is used to create a range verification model which is less computationally intensive than using a full-MC approach.

**Method**: The model relies on the inflection points (the point of sign change in the curvature) of the proton PDD, fluence and PET curves and their interrelationship, using particle fluence to form a relationship between the extrapolated range of proton dose and the activated PET distribution. PET-activation, proton fluence and absorbed dose distributions were simulated using FLUKA-MC for a selection of 11 pristine energies spaced equally apart in our operating proton energy range (69.7-221.3 MeV) for 4 different materials (water, bone, adipose, muscle). The semi-empirical model was then verified using physical phantom experiments.

**Results and Conclusions**: Our analysis shows that when normalizing for water equivalent length (WET), material-dependent differences between the PET-image extrapolated range and that of the dose normalize, enabling range verification with 1.6-2% accuracy (low to high energies) without the need for MC simulations. Preliminary experiments with physical phantoms show feasibility of the approach. The developed method has the potential to accelerate PET-based range verification processing, improving applicability of this technique in treatment centers.

### P 212: Evaluation of Setup Corrections and Stability during Image-Guided Particle Therapy

#### R. Ricotti^1^, A. Pella^1^, B. Tagaste^1^, A. Giorgetto^1^, F. Valvo^1^, M. Ciocca^1^, G. Baroni^1,2^, R. Orecchia^3^

##### *^1^Centro Nazionale di Adroterapia Oncologica CNAO, Clinical Department, Pavia, Italy*, *^2^Politecnico di Milano University, Dipartimento di Elettronica Informazione e Bioingegneria, Milan, Italy*, *^3^Centro Nazionale di Adroterapia Oncologica CNAO, Scientific Directorate, Pavia, Italy*

**Purpose**: In this study we report patient setup correction vectors assessed by daily orthogonal X-ray images recorded at the Centro Nazionale di Adroterapia Oncologica (CNAO, Italy).

**Materials and Methods**: A total number of 6269 treatment fractions delivered between February and November 2017 at CNAO were retrospectively evaluated including cranial and extra-cranial treatments. The study was designed in order to assess:

1. The overall range of translation and rotational setup correction parameters (Overall Correction Vector, OCV);
2. The frequency and related correction ranges of cases, in which a single correction vector was applied (Single Correction Vector, SCV);
3. The frequency and related correction ranges, which forced an additional X-ray verification (Required Verification, RV);
4. The frequency of cases which required the application of an additional correction vector and related range of additional correction parameters (Multiple Correction Vector, MCV).

In order to investigate setup stability along irradiation, multiple CVs calculated across multi-field sessions (Inter-beam CV, IBCV) were evaluated, when available. Results are reported separately according to the different image guidance hardware available in CNAO treatments rooms.

**Results**: A SCV was applied in the majority of the cases and X-ray verification was required in less than 20% of analysed fractions (Tab1). The MCV was applied in less than 10% and the range of the additional correction was smaller than RV. In 767 fractions inter-beam setup stability was assessed with median correction values less than 0.6mm and 0.2°(Fig1).

**Conclusion**: This quantitative analysis on a large number of treatment fractions gives an overview of current clinical practice in patient positioning procedure

### P 214: Genotoxicity of Proton CT Irradiation

#### L. Yasui^1^, M. Pankuch^2^, J. Edwards^2^, G. Coutrakon^3^, C. Sarosiek^3^, R. Schulte^4^

##### *^1^Northern Illinois University, Biological Sciences, DeKalb, USA*, *^2^Northwestern Medicine, Chicago Proton Center, Warrenville, USA*, *^3^Northern Illinois University, Physics, DeKalb, USA*, *^4^Loma Linda University School of Medicine, Basic Sciences Division of Biomedical Engineering Sciences Division of Physiology, Loma Linda, USA*

Benefits of proton computerized tomography (CT) over conventional X-ray CT for proton therapy planning and image guidance include improved tissue stopping power information, no image streaks from dental fillings, and a reduction by about 10 fold in dose compared to X-ray CT. However, biological effects of proton CT irradiation are unknown. In this report, the genotoxicity of proton CT with 200 MeV and γ irradiation from a 137Cs source were assessed using γH2AX foci, which mark the presence of DNA damage, in particular, DNA double-strand breaks. The loss of γH2AX foci measures DNA damage repair. Normal human astrocytes and normal human umbilical vascular endothelial cells were used. For the proton CT experiments, cells were irradiated behind a mock-setup of a pCT scanner in a tissue equivalent cube inserted into the posterior fossa of a rotating pediatric head phantom. Proton CT dose calibrations performed in this head phantom set-up corroborated standard clinical dosimetry methods. Cells were exposed to 200 MeV protons used for proton CT. A dose-response curve was acquired for cells irradiated with 0, 0.1, 0.5, 1.0, 1.5 or 2 Gy. After a γH2AX foci formation time of 30 minutes, cells were prepared for confocal laser scanning microscope imaging of γH2AX foci. DNA damage repair data was acquired for cells after 0, 30 minutes, 60 minutes, 6 hours, 12 hours and 24 hours after exposure to 1 Gy. Altogether the results of this team effort provide a valuable estimation of any genotoxic effects from exposure to proton CT.

### P 215: Measurement of Cross Sections Relevant for PET Range Verification in Proton and Heavy Ion Therapy

#### F. Horst^1,2^, W. Adi^3^, K.T. Brinkmann^3^, L. Nies^3^, M. Rovituso^4^, C. Schuy^2^, U. Weber^2^, H.G. Zaunick^3^, K. Zink^1,5^

##### *^1^THM University of Applied Sciences, Institute of Medical Physics and Radiation Protection, Giessen, Germany*, *^2^GSI Helmholtz Centre for Heavy Ion Research, Biophysics, Darmstadt, Germany*, *^3^Justus Liebig University, II. Physics Institute, Giessen, Germany*, *^4^INFN, TIFPA Trento Institute for Fundamentals Physics Applications, Trento, Italy*, *^5^University Medical Center Giessen-Marburg, Department of Radiotherapy and Radiooncology, Marburg, Germany*

The analysis of PET activation distributions induced by the therapeutic beam is an established technique for in-vivo range verification in proton and heavy ion therapy. For this method, the distribution of the positron emitters within the irradiated tissue needs to be calculated for each individual treatment plan. Such transport calculations require a precise knowledge of the underlying nuclear reactions, i.e. the production cross sections for the isotopes of interest (mainly 10C, 11C and 15O).

In order to measure such cross sections, a detector system consisting of three BaF2 scintillators was developed and characterized, which allows to detect the annihilation photons emitted by the activation products in coincidence. Two scintillators measure the rate of 511 keV photons emitted in 180° and the third scintillator is used to measure the random coincidence rate. A short proton or heavy ion pulse impinges on a target (e.g. graphite or Beryllium oxide) and activates the material. The initial activities of the generated target fragments (10C, 11C, 15O) can be obtained by fitting the measured decay curve with a superposition of multiple exponential functions with different half-lives corresponding to the different isotopes. Finally, the production cross sections can be calculated from these initial activities.

A first experiment was performed at the Trento proton therapy Center (Italy) to measure 12C (p,pn)11C- and 12C (p,p2n)10C cross sections in the energy range 50–230 MeV with the developed setup. The obtained cross sections are in good agreement with literature data and complement them well. Future measurements with heavier projectiles (e.g. 12C) are planned.

### P 216: The Necessity of 4D-Motion Monitoring for Thoracic Tumors Treated with Pencil Beam Scanning Proton Therapy: A Comprehensive 4D-Imaging Study

#### L. Den Otter^1^, R.M. Anakotta^1^, M. Dieters^1^, C.T. Muijs^1^, S. Both^1^, J.A. Langendijk^1^, A.C. Knopf^1^

##### ^1^University Medical Center Groningen, Radiotherapy, Groningen, Netherlands

**Purpose/Objective**: For pencil beam scanning proton therapy (PBS-PT), moving targets remain challenging due to the interplay effect. Even when using motion mitigation strategies, one needs to be aware of motion variations. We investigated weekly and daily motion variations to define the most optimal motion monitoring protocol for PBS-PT treatments of lung cancer patients.

**Material/Methods**: For 20 stage II-IV (N)SCLC patients 4DCT imaging was performed during treatment simulation (week 0) and weekly during the treatment course. GTVs were delineated on the maximum inspiration and expiration 4DCT phases and the centroid 3D-vector translations were evaluated. For one patient, daily 3D-vector centroid 4DCBCT motion was evaluated additionally.

**Results**: A median initial tumor motion (Figure 1) of 1.3 mm (range: 0.0 – 7.4 mm) was observed. GTV motions varied each week; 7 out of 20 patients showed motion variation >3 mm compared to the motion measured in week 0. Figure 2 shows that motion amplitudes extracted from weekly 4DCTs were not predictive for motion amplitudes extracted from daily 4DCBCTs.

**Conclusion**: For a considerable part of the patients, the motion measured in week 0 based on weekly repeat 4DCT imaging was not predictive for motion in the following weeks. Daily motion measured by 4DCBCT imaging for one patient suggests that weekly measured 4DCT motion is not predictive for the daily motion in between the weekly 4DCTs. This indicates that breathing motion differs from day to day and daily 4D-imaging is therefore needed to assure safe PBS-PT treatments for lung cancer patients.

### P 219: Optical Surface Imaging for Markerless Robot Monitoring and Feedback

#### K. Huang^1^, E. Lee^2^, A. Mascia^3^

##### *^1^University of Cincinnati, Radiation Oncology, Cincinnati, USA*, *^2^UC Health, Radiation Oncology, CIncinnati, USA*, *^3^Cincinnati Children's Hospital / UC Health, Radiation Oncology, Cincinnati, USA*

**Background**: Active scanning proton therapy promises unparalleled potential for dosimetric precision, placing heavier demands on patient setup precision and accuracy. Limitations in robotic positioning, imaging, and motion management result in higher imaging dose, slower patient setup, and lower system accuracy. It is estimated that more than 15% of proton fractions delivered exceed robotic couch tolerance. Markerless optical surface imaging can track system positioning ensure precise and accurate patient positioning while reducing imaging dose and streamlining treatment workflow.

**Methods**: Commodity depth-sensing cameras observed and provided detailed 3D data of the gantry area and treatment couch. A reference template model of the treatment couch was automatically constructed from the observed geometry. Position and pose of the treatment couch was determined with 6 degrees of freedom in near-real time using 3D gaussian mixture modelling. Couch rotations and translations in 6 axes were monitored and tracked.

**Results**: The optical system successfully tracked the relative treatment couch rotations in the yaw, pitch, and roll axes as well as translations ranging from 1mm to 1cm in all axes.

**Conclusions**: Markerless optical surface imaging can track the relative position, pose, and movement of system components in near-real time. Future system calibration and fusion of data from multiple cameras are anticipated to enable robust absolute tracking to confirm robotic couch positioning and to provide dynamic feedback without the time and radiation dose required for additional radiographic imaging.

### P 220: Immobilization Devices for Low Field Strength MR Simulators

#### S. Huh^1^, E. Kryck^1^, D. Indelicato^2^, E. Viviers^1^

##### *^1^UF Health Proton Therapy Institute, Physics, Jacksonville, USA*, *^2^UF Health Proton Therapy Institute, Physicians, Jacksonville, USA*

**Introduction**: Immobilization is crucial for acquiring MR images used for defining GTVs, as well as during mid-treatment surveillance for evaluating tumor progression or changes to normal anatomy. A 0.23T Philips MR simulator has been used to acquire weekly surveillance images of pediatric cancer patients with craniopharyngioma and low-grade glioma, and mid-treatment prostate cancer patients who receive proton therapy. Various immobilization techniques and devices have been fabricated and used to minimize movement clinically without requiring the use of anesthesia/sedation in pediatric patients.

**Methods/Materials**: For head and neck patients, the head is immobilized in the head coil using a custom-made headrest and side compression device which also provides music with volume control. This was partially made by modifying a commercial blood pressure cuff. A gazing device is attached to the top of the head coil which includes three light-emitting LEDs that are controlled remotely. For prostate patients, another compression device was fabricated and used with the body coil to minimize breathing motion to the prostate during long scan times. Our MR images using the immobilization devices were compared with higher field strength diagnostic MR images.

**Results**: The custom-made immobilization devices significantly reduce motion artifacts during relatively long scan times. Using the immobilization devices with our low field strength MR simulator provides better images for contouring and surveillance than diagnostic images.

**Conclusion**: Proton therapy clinics which have low field strength MR simulators can produce valuable images for defining GTVs as well as mid-treatment surveillance for adaptive planning, provided proper immobilization techniques and devices are used.

### P 221: Using Low Field Strength MR Motion Studies to Reduce PTV Margins in Prostate Cancers

#### S. Huh^1^, E. Kryck^1^, N. Mendenhall^2^, W. Mendenhall^2^, R. Henderson^2^

##### *^1^UF Health Proton Therapy Institute, Physics, Jacksonville, USA*, *^2^UF Health Proton Therapy Institute, Physicians, Jacksonville, USA*

**Introduction**: A motion study has been investigated with the goal of reducing PTV margins in prostate cancers. Reduced PTV margins while maintaining tumor coverage could decrease genitourinary (GU) and gastrointestinal (GI) toxicities in patients who receive proton therapy. In our study, we have used a 0.23T MR simulator to evaluate the motion of the prostate.

**Methods**: A Philips 0.23T Panorama MR was used during simulation to evaluate the motion of the prostate, as well as surrounding organs and rectal balloon. Motion studies using THRIVE and T2 TSE pulse sequences were used to define contours of the prostate, seminal vesicles, rectal wall, and large bowels. The scanning protocols and the pulse sequences were optimized so that the fiducials and rectal balloons were clearly visible. 100 sets of sagittal images were acquired over a period of 5 minutes and used to create 2D maximum intensity projections (MIP) and minimum intensity projections (MINIP). The motion of the prostate and other organs was estimated by subtracting the MIP from the MINIP images.

**Results**: The maximum movement of the organs was found to be on the order of 1-2mm, signifying that CTV to PTV margins could be reduced. The clinical applications of this study with data from > 20 patients will be presented.

**Conclusion**: MR motion studies can be used to evaluate the movement of the prostate and other organs, potentially reducing the PTV margins in prostate cancer treatment planning.

### P 222: Characterizing the Pencil Beam Scanning Dosimetric Impact from Symmetric and Asymmetric Respiratory Motion on Target Dose using Conformation Number

#### T. Phan^1^, E. Lee^2^, D. Moyer^1^, A. Mascia^2^

##### *^1^University of Cincinnati, Radiation Oncology, Cincinnati, USA*, *^2^Cincinnati Children's Hospital Medical Center / UC Health Proton Therapy Center, Radiation Oncology, Cincinnati, USA*

Pencil beam scanning proton therapy makes possible intensity modulation, resulting in improved target dose conformity and organ-at-risk (OAR) dose sparring. This benefit, however, results in increased sensitivity to certain clinical and beam delivery parameters, such as respiratory motion. These effects can cause plan degeneration, which could lead to decreased tumor dose or increased OAR dose. This study evaluated measurements made with a 2D ion chamber array and a 1D motion platform in solid water. Respiratory motion was simulated using sine and cosine4 waves representing symmetric and asymmetric breathing motions, respectively. Motion amplitude was 5 mm or 20 mm with 5 seconds breathing cycle. The treatment plans were created to mimic spherical targets of 3 cm or 10 cm diameter with its top surface located at 5 cm or 1 cm from solid water phantom surface. Reference RBE dose of 200 cGy was delivered for each dataset. We evaluated dose conformity at the center plane of targets by using the Conformation Number [van't et al]. Results indicated that dose conformation was more affected by motion for smaller targets. Range shifter with shallow targets reduced the motion effect. Dose conformity was better achieved for symmetric breathing patterns than asymmetric breathing patterns. More fractions generally helped mitigate the degradation of dose conformity due to respiratory motions except the case of small target with range shifter under asymmetric breathing motion. For future work, other metrics of plan degradation will be evaluated, as well as further permutations of parameters affecting the dose-interplay artifacts.

### P 223: Investigating Statistical Models of Deformation for Proton Plan Adaption

#### M. Wilson^1^, S. Holloway^1^, G. Royle^1^

##### ^1^University College London, Medical Physics and Biomedical Engineering, London, United Kingdom

**Introduction**: In proton therapy, knowledge of the sources and magnitudes of uncertainties affecting the proton range is essential for producing plans which are robust to these uncertainties. Despite advances in quantifying set-up uncertainties, there is still a significant challenge in quantifying the residual random proton range uncertainty, and associated probability, arising from patient motion and organ deformation throughout treatment. A feasibility study to produce statistical models of anatomical deformation of patients with head and neck cancer is presented in this work. These patients are known to exhibit significant anatomical changes throughout treatment. Improved understanding of these anatomical changes is important in developing strategies for practical and cost-effective clinical implementation of adaptive radiotherapy.

**Materials and Methods**: Ten patients treated with photon radiotherapy for nasopharyngeal cancer have been retrospectively analysed. In-house and open-source deformable image registration software, NiftyReg, is used to register each patient's planning CT scan to weekly repeat CT scans to the extract deformation vector fields mapping the relative voxel-wise changes of each structure. Principal component analysis is used to extract average and principal modes of deformation of each patient throughout their treatment.

**Results and Conclusion**: Statistical models of organ-at-risk and target volume changes are investigated and the preliminary results presented in this work. Such knowledge will pave the way for predictions of proton plan robustness and adaption accounting for observed deformations of patient anatomy.

### P 224: Clinical Commissioning of Gated Line Scanning Proton Beam for Moving Target Treatment

#### S.H. Ahn^1^, E. Shin^1^, K. Chung^1^, S. Cho^1^, K. Jo^1^, H.C. Park^1^, H. Pyo^1^, Y. Han^1^

##### ^1^Samsung Medical Center, Radiation Oncology, Seoul, Korea Republic of

**Purpose**: Despite the advantages of reducing dose to normal organs with scanning proton therapy, uncertainty due to respiratory motion and interplay effects has limited scanning therapy with moving targets. To overcome such limitations, in Samsung Medical Center (SMC), we commissioned the gated line scanning proton beam using respiratory gating system.

**Methods**: For clinical commissioning of continuous line scanning with gating, we first evaluated the dose accuracy and compared the treatment plan dose, the dose under the gating signal on and off using a two-dimensional array detector on 8 lung and liver patients QA plans. Second, we measured the dose in the sinusoidal motion situation by using in-house moving phantom to check the interplay effect according to the target motion. Third, in order to know the correlation between the target motion and the scanning beam direction, we made the orthogonal and parallel scanning beam pattern to the target motion and compared the dose.

**Results**: Using the same QA plans, compared with the measurements in the without gating signal and with gating on condition, all of them showed 95% or more 2D gamma pass rate at 0.5mm/1.0% criteria. Scanning direction of proton beam in a perpendicular to the target motion produced a large interplay effect than in a parallel direction.

**Conclusions**: The position and dose accuracy of the gated scanning beam is considered to be accurate for clinical application. And gated line scanning method will be applied for Breath-hold and gating treatment of moving target.

### P 225: Novel Rescanning Strategies for Motion Mitigation, Measurements and Clinical Simulations

#### M. Braeuer^1^

##### ^1^Siemens Healthcare GmbH, AT RO PT MC CAP, Erlangen, Germany

For carbon ion systems, the number of tumor sites in the abdomen, requiring motion management is growing. Especially for raster scanning, the rescanning techniques are methods of choice.

For the raster scanning system of the Siemens Healthineers “Iontris” product, simple layer rescanning was developed further, incorporating “random rescanning”. The performance of this system is shown, based on measurements with films, Farmer chambers and a (static) water phantom.

A novel approach, based on detailed considerations of dose built-up in raster scanning was found: Raster scanning as layer scanning leads to an “intrinsic rescanning” for the majority of volume elements, since most of them are irradiated several times with different energies. Only in case of the most distal volume elements, the full, planned physical dose is applied mainly by one energy layer. All other volume elements accumulate doses from several energy layers.

A calculation scheme allows to compute the number of intrinsic rescannings for each volume element. In the so called “relevance rescanning”, only those volume elements are getting rescanning, which get most of their dose from one or few energies.

Combining relevance rescanning with random rescanning and adding an additional algorithm to optimize the beam path in the rescanning process, allows rescanning numbers of above 30 with a very small irradiation time penalty.

Measurements and clinical simulations with dynamic phantoms are presented.

### P 226: Target Motion Compensation By Means Of Audio-Visual Biofeedback for Synchrotron-Based Scanned Heavy-Ion Beam Delivery

#### P. He^1^, Q. Li^1^

##### ^1^Institute of Modern Physics- Chinese Academy of Sciences, Department of Medical Physics, Lanzhou, China

To overcome inefficiencies and interplay effects between the target residual motion and the synchrotron-based scanned heavy-ion beam delivery process, a novel respiratory guidance method was developed to help patients synchronize their breathing patterns with the synchrotron excitation patterns by performing short breath holds (BH) with the aid of personalized audio-visual biofeedback (BFB) system.

The purpose of this study was to evaluate the treatment precision, efficiency and reproducibility of the respiratory guidance method in scanned heavy-ion beam delivery mode. Using 96 15-min respiration traces from eight healthy volunteers who were asked to breathe freely and guided to perform short BHs with the aid of BFB, a series of dedicated 4D dose calculations (4DDC) were performed on a geometric model which was developed assuming a linear relationship between external surrogate and internal tumor motions.

Results showed that with the respiratory guidance method, the treatment efficiency increased by factors of 2.23∼3.94 as compared to FB gating, as shown in figure 1. The magnitude of dose inhomogeneity for the respiratory guidance methods was 7.5 times less than that of the non-gated irradiation and good reproducibility of breathing guidance among different fractions was achieved, as shown in figure 2. Thus, our study indicates that the respiratory guidance method not only improved the overall treatment efficiency of respiratory-gated scanned heavy-ion beam delivery, but also had the advantages of less dose uncertainty and better reproducibility among fractions (Figure 1: Boxplots for the treatment time over four fractions; Figure 2. Values of the HI over four fractions).

### P 227: Evaluation of Interplay Effects and Re-Scanning Technique in the Line Scanning Method

#### Y. Ito^1^, M. Araya^1^, Y. Sugama^1^, N. Yanagisawa^1^, H. Fujimoto^1^, I. Maeshima^1^, G. Shibagaki^2^, T. Nishio^3^, H. Onishi^4^

##### *^1^Aizawa Hospital, Proton Therapy Center, Matsumoto- Nagano, Japan*, *^2^Sumitomo Heavy Industries. Ltd., Industrial Equipment Division, Shinagawa- Tokyo, Japan*, *^3^Tokyo Women's Medical University, Graduate School of Medicine, Shinjyuku- Tokyo, Japan*, *^4^University of Yamanashi, School of Medicine, Kofu- Yamanashi, Japan*

**Purpose**: In order to start the treatment using line scanning method for moving targets with small respiratory motion without gating or breath holding, we evaluated the interplay effects and re-scanning technique.

**Method**: Several plans for delivering a uniform dose to a rectangular target volume were created using Eclipse Version 13.7. Five volumes of targets (Field size: ranging from 160mm x 160mm to 40mm x 40mm, SOBP width: ranging from 80mm to 40mm) were used for plans with energies of 110MeV and 150MeV. These plans were irradiated with volumetric re-scanning technique. The number of re-scans was 1, 3 and 5.OCTAVIUS Detector 729 XDR and solid phantom was used for measurements. In order to provide motion for the measurement system, the Quasar respiratory motion platform was selected. The motion amplitude was increased from 2mm to 5mm.We used the gamma-index (2mm/2%) for comparison between dose distribution with movement and dose distribution without movement.

**Results**: In case of re-scan number of one, the maximum and minimum gamma-passing rates were 97.6% and 41.7%. In the latter case, the gamma-passing rate with re-scan number of five was 96.2%.It seemed that a longer irradiation time and larger amplitude increase interplay effect. We thought that five times re-scanning was sufficient to mitigate the interplay effect in these cases.

**Conclusion**: We evaluated the interplay effects and re-scanning technique in the treatment for moving targets with small respiratory motion without gating or breath holding.

### P 228: Proton Radiotherapy for Cardiac Soft Tissue Sarcoma and Arrhythmia Ablation

#### H. Lee^1^, Y.L. Chen^1^, K.W. Jee^1^, H.M. Lu^1^, J. Pursely^1^

##### ^1^Mass General Hospital, Radiation Oncology, Boston, USA

Conformal delivery of radiation to a cardiac target is challenging due to the cardiac motion, the toxicity and the proximity to radiosensitive organs at risk (OARs). Cardiac sarcomas have been treated with respiratory gating but the benefit of mitigating cardiac motion is unknown. Although the recent studies of cardiac sarcomas and arrhythmia treatments with various radiotherapy modalities have shown benefits, cardiac motion mitigation techniques have been poorly studied. The purpose of this work is to evaluate the dosimetric efficacy of cardiac gating for a motion mitigation method, gating the beam in synchrony with a ECG signal. Cardiac gated 4D CTs were obtained at the end-expiration, resulting in 10 phases. Six targets (mock tumors and ablation lesions) and OARs were contoured on each phase for cardiac gating and 3D target volumes were generated for non-cardiac gating. For each target, beam angles were compared at a reference phase and an optimal direction/combination was selected. Both plans were generated with the double scattering proton treatment modality. Due to the anatomical location of the heart, an angle of incoming beams plays an important role in dose homogeneity and OARs dose. Thus, the beam angles should be an important consideration in cardiac irradiation. Also among various target locations, targets in ventricles benefit the cardiac gating, reducing OARs dose by ∼20% and minimizing the mean heart and left ventricle doses. It demonstrates the beam angle is important to spare OARs and cardiac gating is beneficial for targets (Figure 1: beam angle; Figure 2: gating comparison).

### P 229: Integrating 4D and Simulating CT Imaging to Evaluate Liver Dome Deviations within Different Respiratory Phases and Associated Proton Dosimetry Variations

#### H.K.T. Liang^1,2^, H. Takei^1^, T. Tomita^1^, T. Isobe^1^, T. Okumura^1^, T. Sakae^1^, H. Sakurai^1^

##### *^1^University of Tsukuba, Departments of Radiation Oncology, Tsukuba, Japan*, *^2^National Taiwan University, Radiation Science and Proton Therapy Center, Taipei, Taiwan\r*

**Background**: Proton dosimetry variations caused by respiration for irradiation fields near liver dome remain unclear. We investigated liver dome deviations within different respiratory phases and the associated dosimetry variations.

**Methods**: Amplitude-gated 4D-CT imaging was used to evaluate liver craniocaudal (Z-axis) displacements and lateral (X-axis) differences. Z-axis was the sagittal and coronal plane intersection containing liver dome top at end-expiration (Z=0). Parameter definitions included ΔZmax: Z-displacement of liver dome top between end-expiration and end-inspiration; ΔXex20% or ΔXin20%: X-difference of liver width within 20% phase of end-expiration or end-inspiration measured using the axial sections at Z=-15 and -30 mm; CTV-V95%: clinical target volume percent receiving ≥95% of the prescribed dose; Dmax%: maximum dose percent. The proportion of ΔXex20% or ΔXin20% to ΔZmax was analyzed through linear regression with coefficient of determination (R2). 4D-CT and simulating imaging were integrated to evaluate the proton dosimetry variations using uniformly created tumors (Hitachi-VQA-double-scattering system).

**Results**: Ten patients (33 datasets) were retrospectively analyzed. At Z=-15 and -30 mm, ΔXex20% (R2=0.67 and 0.81) rather than ΔXin20% (R2=0.54 and 0.22) was moderately correlated to ΔZmax, especially when ΔXex20% measured at Z=-15 mm (slope: 2.72) (Figure 1). Three patients with ΔZmaxof 10, 17, 22 mm, the corresponding results at Z=-15 mm were ΔXex20%: 3.9, 10.9, 29.3 mm, ΔCTV-V95%: 0, -16, -15%, spinal cord ΔDmax%: 0, 1.4, 10.9%, respectively (Figure 2).

**Conclusion**: Simulating CT integrated with 4D-CT imaging is suggested to evaluate the proton dosimetry variations for fields near liver dome when irradiating at end-expiration with large respiratory amplitude and at end-inspiration.

### P 230: Technical Workflow of Respiratory Gated Irradiation Using Carbon-Ion Fast-Scanning Beam in Combination with External Respiratory Sensor

#### S. Minohara^1^, Y. Kusano^1^, E. Takeshita^1^, Y. Matsuzaki^1^

##### ^1^Kanagawa Cancer Center, Medical Physics and Engineering, Yokohama, Japan

By combining the carbon-ion pencil-beam fast re-scanning and the gating with respiratory sensor (Anzai, AZ-733VI-IRP) on the body surface, we overcame the interplay-caused inhomogeneous dose distribution that was a weak point of previous pencil-beam scanning methods. Our clinical criteria are to keep the respiratory motion less than 5 mm during the gate-on and to re-scan each layer at six times (fig.).

Using dynamic image data such as 4D-CT and a set of orthogonal X-ray serial images with respiration waveform, GTV, CTV, and PTV are delineated, and the gate level are decided. In the treatment room, the patient is positioned based on bone structures of X-ray FPD images along with our standard setup procedure. And just before the irradiation, the motion of fiducials (Gold Anchor) that are implanted close to the target are checked by the oblique X-ray fluoroscopic images. The acceptable range of the marker motion during gate-on is compared to the template that is reconstructed in the treatment planning system, and the correlation between marker motion and respiration waveform are checked. If the respiration waveform are drift during irradiation, the gate signal is turn off manually and we re-check the range of marker motion and the relation with respiration waveform. If necessary we can verify the detail using in-room 4D-CT images.

We have smoothly treated 27 patients with liver cancer. In our clinical experiences, the respiration waveform by our novel respiratory sensor seems to have a very good correlation with the movement of the internal marker.

### P 231: A GPU-based 4D Dose Calculator for a Pencil-Beam Scanning Proton Therapy System

#### M. Pepin^1^, E. Tryggestad^1^, H. Tseung^1^, J. Johnson^1^, M.G. Herman^1^, C. Beltran^1^

##### ^1^Mayo Clinic, Radiation Oncology, Rochester- MN, USA

**Purpose**: To develop a Monte-Carlo-based tool for the estimation of dose from spot-scanned delivered protons in the presence of respiratory motion.

**Methods**: A GPU-based 4D dose calculation platform was developed to simulate the delivery of a spot-scanned treatment plan while accounting for patient breathing as approximated by 4DCT. The planned spots were divided across the 4DCT phases according to delivery parameters which characterize the Mayo Clinic synchrotron (Hitachi ProBeat). The per-phase subplans were then simulated using a Monte Carlo dose engine. To accumulate doses, deformable image registration was performed to generate DICOM deformation vector fields which map each phase to the end-exhalation phase (EE). The doses were then mapped to the EE using an energy-/mass-transfer mapping technique to obtain the total dose. To accommodate voxel volume/mass changes with deformation, each nominal voxel was divided into n3 subvoxels and the energy and mass for all subvoxels were mapped to the EE. The dose in an EE voxel was computed as the ratio of the summed energy and mass of all subvoxels which map to it.

**Results**: The dose distributions from individual phases, mapped to the EE, vary from each other and the total distribution as shown in Figure 1. The total distribution converges at subvoxel volumes of ∼0.5 mm3 and is degraded, from motion, compared to a 3D delivery on the EE as given in Figure 2.

**Conclusions**: A practical (<10 min) Monte-Carlo-based 4D dose calculation engine was developed and is ready for 4D plan evaluation and for future application to 4D robust optimization.

### P 232: Sensitivity of Intensity Modulated Proton Therapy (IMPT) and Volumetric Modulated Arc Therapy (VMAT) Plans to Breathing Motion in Thoracic Tumors

#### S. Teoh^1^, F. Fiorini^1^, B. George^1^, K.A. Vallis^1^, F. Van den Heuvel^1^

##### ^1^University of Oxford, CRUK and MRC Oxford Institute for Radiation Oncology, Oxford, United Kingdom

**Purpose**: To compare plan sensitivity to breathing motion in planning target volume (PTV) optimized- and robust-optimized (RO) IMPT and VMAT plans in thoracic tumors.

**Materials and Methods**: Ten patients with varying tumor motions (up to 16.4mm) were planned retrospectively. The prescribed dose was 70Gy in 35 fractions. Three nominal plans (Dnom) were created for each patient: VMAT, PTV- and RO-IMPT plans. 4D dose (4DD) evaluation was calculated by accumulating the individual phase dose using deformable registration (DIR). DIR was validated subjectively and objectively by computing the jacobian determinant. 4DD were compared against Dnom. Sensitivity criteria for the target were: CTVD98% deviation of less than 5% between 4DD and Dnom and 4DD CTV V95% >99.9%. Where neither IMPT plans were robust, plans were re-optimized using 4D robust optimization (4DRO-IMPT). All treatment planning and DIR were performed in Raystation 5.0.2 (Raysearch, Sweden).

**Results**: Two VMAT nominal plans failed to meet organs-at-risk constraints at prescribed dose. Following initial DIR validation, 3 patients were found to have physically implausible deformation. Registration was improved by adding segmentations and having focus regions of interest. Overall 8 VMAT, 8 PTV-IMPT and 9 RO-IMPT plans were acceptable. With 4DRO-IMPT, all ten patients had acceptable plans. Smaller tumors tended to be more mobile; however, the degree of motion did not predict plan robustness.

**Conclusion**: Verification of DIR is important to ensure accuracy of 4DD evaluation. Evaluation of thoracic IMPT plans sensitivity to breathing motion is needed to ensure target coverage. VMAT and RO-IMPT plans are generally superior in robustness against breathing motion.

### P 233: Breast Cancer Irradiation Using Proton Pencil Beam Scanning in Deep Inspiration Breath Hold

#### M. Andrlik^1^, V. Vondracek^1^, J. Kubeš^2^, J. Vilímovský^1^, A. Pastorová^2^, M. Navrátil^1^, S. Štastná^1^, K. Badraoui Čuprová^1^, L. Zámečník^1^, S. Horová^1^

##### *^1^Proton Therapy Center Czech S.R.O., Department of Clinical Physic, Praha, Czechia*, *^2^Proton Therapy Center Czech S.R.O., Proton Therapy Department, Praha, Czechia*

Proton therapy using pencil beam scanning (PBS) in the breast cancer irradiation allows to achieve perfect coverage of the target volume and maximize sparing of heart, contralateral breast and lungs. It is necessary to suppress influence of breathing interplay effect on the final dose distribution. More over proton therapy using PBS brings further requirements for patient setup precision. Filling of all of these requirements makes possible to change perfect looking treatment plans to the clinical usage.

The breast cancer of sixty-five patients was treated by the PBS from April 2015 to December 2017. The two-step patient setup protocol was used. Both steps are performed in deep-inspiration-breath hold. This breath hold technique suppresses motion of chest wall and improves treatment-volume-position reproducibility. It was important to use the information about post-surgery marker position in combination with the patient reference surface using two infrared stereoscopic cameras. The reference surface was exported directly from the treatment planning system.

The QA plans were calculated for these treatment planning techniques and robustness was evaluated for 5 mm-field shifts in orthogonal directions.

It was shown that beam scanning proton therapy in deep inspiration breath hold is suitable for left-side breast cancer with acceptable acute and late skin toxicity. It allows minimal doses to the heart, lungs and contralateral breast. This should be beneficial to patients with long life expectancy and also to patients with associated heart diseases. This treatment technique is also enough robust against the position inaccuracy.

### P 234: Feedback Based Voluntary Moderate Breath Hold Proton Pencil Beam Scanning Treatment

#### P. Wang^1^, K. Leach^2^, T. Shikui^3^, J. Sturgeon^4^, A. Lee^4^, V. Mangona^4^, C. Chang^3^

##### *^1^University of Maryland School of Medicine, Maryland Proton Treatment Center, Baltimore, USA*, *^2^Texas Center for Proton Therapy, Dosimetry, Dallas, USA*, *^3^Texas Center for Proton Therapy, Physics, Dallas, USA*, *^4^Texas Center for Proton Therapy, Physician, Dallas, USA*

**Purpose**: To introduce the experiences of our initial feedback based voluntary moderate breath hold (BH) proton pencil beam scanning treatment. The presentation will elaborate the results along with the necessary and desired techniques to implement such treatment in the clinic.

**Methods**: Three CT scanning to build the patient's anatomy model are performed during the patient's voluntary moderate BHs, where the BH plateau reaches 100%, 90% and 110%. All three CT scans are involved in the optimization during the treatment planning process, so that the final nominal plan is valid for a BH range. Both orthogonal kV imaging and pausable CBCT are implemented for patient alignment with BH prior to the treatment. The proton beam delivery can be paused as well by the therapists according to the patient's BH status. The CBCT images are analyzed for the BH reproducibility and virtual delivered dose (VDD) retrospectively. The rigid image registration between CBCT and planning CT (pCT) helps to find the BH reproducibility. To find the VDD, a series of deformable image registrations (DIR) are performed between CBCT and pCT, which avoids dose calculations on CBCT images directly (Fig.1).

**Results**: The values of the mean, standard deviation, maximum and minimum of the tumor location difference between the CBCT and pCT are 1.9, 1.6, 4.7 and 0 mm respectively. The percentage difference in targets D95 of VDD is within 1.5% of nominal plan (Fig.2).

**Conclusion**: BH technique can be implemented in proton pencil beam scanning treatment safely and effectively.

### P 235: Necessity of Manually Adjusting Automatic Deformed CTVs on Repeat CT Scan to Evaluate CTV Coverage in IMPT For Lung Cancer

#### H.P. Van Der Laan^1^, M. Anakotta^1^, E.W. Korevaar^1^, M. Dieters^1^, R. Wijsman^1^, J.F. Ubbels^1^, J.A. Langendijk^1^, A.C. Knopf^1^, C.T. Muijs^1^, S. Both^1^

##### ^1^University of Groningen- University Medical Center Groningen, Department of Radiation Oncology, Groningen, Netherlands

**Background/Introduction**: Manual CTV adjustment after deformable CTV registration on repeat-CT scans (rCT) is time consuming and may delay decisions on whether or not to perform plan adaptations. Our aim was to investigate if omitting such manual CTV adjustments would alter the decisions and/or impact the overall adequacy of treatment.

**Methodology**: Seventeen lung cancer patients underwent a 4D planning-CT (pCT0) and 3-5 weekly 4D rCTs. In total 82 4D-average rCTs were available. Robustly optimized IMPT plans were established on the pCT0 aiming for a CTV D98 ≥ 57 Gy. The plans were evaluated on all rCTs including setup and range error scenarios. CTVs were deformed (intensity based deformable registration) from pCT0 to the rCTs (CTVauto) and manually adjusted by a clinician (CTVman). When rCT CTVauto and/or CTVman D98 < 56.5 Gy (worst case scenario dose) the IMPT plan was adapted.

**Results**: On average, CTVman was smaller than CTVauto (Δ volume in weeks 1-5: -4%, -6%, -9%, -11% and -15% (p < 0.001), respectively. The average CTVauto D98 and CTVman D98 on all rCTs were 55.17 Gy and 55.13 Gy (p = 0.767), respectively. Omitting CTVman on all 82 rCTs would result in 3 ‘unnecessary' plan adjustments and 1 ‘skipped' plan adjustment. In the latter single case, the weekly rCTs dose deformed and summed in the CTV on pCT0 was adequate despite the skipped plan adaptation (58.1 Gy).

**Conclusions**: In 17 lung cancer patients, omitting manual adjustments of deformed CTVs on weekly rCTs did not impact adequate CTV coverage of IMPT treatment.

### P 236: Evaluation of Dual Energy CBCT Accuracy Comparing Different Methods and Reconstructions

#### A. Katsis^1^, E. Abel^1^

##### ^1^Varian Medical Systems, Global Translational Science, Palo Alto, USA

**Purpose**: Stopping power range uncertainty is a main hurdle in fully utilizing the Bragg peak during proton therapy. In addition, the ability to perform treatment planning using an on-board cone beam CT (CBCT) scanner is necessary for future adaptive planning. A dual energy CBCT approach could allow for the generation of accurate stopping power maps at the time of treatment. This study provides a quantitative evaluation of various dual energy methods using different CBCT reconstructions.

**Methods**: Scans of a Catphan 504 phantom, were taken on a benchtop CBCT scanner with ProBeamTM imaging geometry and reconstructed using the Varian default, advanced scatter correction, and iCBCTTM algorithms. Electron density (rho) and effective atomic number (Zeff) maps were then generated using the published methods of Hunemohr, Yang, and Saito. The calculated rho and Zeff accuracy of each material in the phantom was compared to results from the literature which used a standard simulation CT scanner.

**Results**: Using the iCBCTTM reconstruction and the Saito method, clear rho and Zeff maps were generated as shown below (Figure 1: Rho and Zeff maps with iCBCTTM reconstruction and Saito method). In addition, using the advanced scatter correction reconstruction and Saito method, rho and Zeff percent differences of < 1% and < 5% respectively were calculated for each material.

**Conclusion**: We have demonstrated that dual energy methods can be used with CBCT scans for accurate material determination. The improved accuracy is heading in the direction for use of setup CBCT scans to help inform time-of-treatment planning decisions.

### P 237: A New Prompt Gamma-Ray Detection System for 3D Range Verification in Proton Beam Therapy

#### C.M. Panaino^1^, R.I. Mackay^1,2^, M.J. Merchant^1,2^, S. Green^3,4^, B. Phoenix^3^, T. Price^3^, P.H. Regan^5,6^, R. Shearman^5,6^, K.J. Kirkby^1,2^, M.J. Taylor^1,2^

##### *^1^University of Manchester, Institute of Cancer Sciences, Manchester, United Kingdom*, *^2^The Christie NHS Foundation Trust, Christie Medical Physics and Engineering, Manchester, United Kingdom*, *^3^University of Birmingham, School of Physics and Astronomy, Birmingham, United Kingdom*, *^4^University Hospitals Birmingham NHS Foundation Trust - Queen Elizabeth Hospital, Medical Physics Department, Birmingham, United Kingdom*, *^5^National Physical Laboratory, Radioactivity Group - AIR Division, Teddington, United Kingdom*, *^6^University of Surrey, Department of Physics, Guildford, United Kingdom*

Over a fractionated course of treatment anatomical changes can severely impact the delivered dose distribution from that planned; evaluation of these changes on a fraction-by-fraction basis is essential. We report the first results of a new method to determine proton beam range in three dimensions, for pencil-beam scanning systems, with an uncertainty below 3 mm. The range is determined through the reconstruction of the origin of prompt gamma (PG) rays emitted from nuclear de-excitations following proton bombardment.

The prototype system is comprised of 16 symmetrically-spaced LaBr3(Ce) detectors, in a spherical design. Initially the position reconstruction capability of the detector system was examined using Geant4 simulations. To determine the PG-rays emission positions in 3D, the information recorded by each detector is fed into a reconstruction algorithm. The development, testing and laboratory validation of the algorithm has been conducted using a sealed 60Co source.

Preliminary simulation results show that for an ideal detector system the reconstruction algorithm is capable of determining the source position to within 1 mm in 3D space. The algorithm is also able of discriminating between multiple sources with a relative separation of 0.15 mm. Figure 1 shows the reconstructed position of a 60Co source (top 2D, bottom 1D projection) shifted by 3 cm along the hypothetical beam direction. Having obtained proof-of-principle for the reconstruction algorithm with a sealed source the next stage is to investigate how the system performs with a proton beam. (Figure 1: Reconstructed position of a 60Co source in (3,0,0) cm in 2D (top) and along the X-axis – bottom.)

### P 238: Study of Biological Washout of 15O Implanted in Rabbit Using In-Beam Open PET Imaging

#### C. Toramatsu^1,2^, H. Wakizaka^2^, A. Mohammadi^2^, Y. Iwao^2^, F. Nishikido^2^, E. Yoshida^2^, H. Tashima^2^, A. Kitagawa^3^, K. Karasawa^2^, T. Yamaya^2^

##### *^1^Tokyo Women's Medical University, Department of Radiation Oncology, Tokyo, Japan*, *^2^National Institute of Radiological Sciences NIRS at National Institutes for Quantum and Radiological Science and Technology QST, Dept. of Radiation Measurement and Dose Assessment, Chiba, Japan*, *^3^National Institute of Radiological Sciences NIRS at National Institutes for Quantum and Radiological Science and Technology QST, Dept. of Accelerator and Medical Physics, Chiba, Japan*

During particle therapy, positron-emitting radionuclides, such as 15O and 11C are produced through nuclear fragmentation reaction, and positron-emission tomography (PET) imaging has emerged as a practical approach for in-vivo range verification. The biological washout effect must be taken into account for the verification. In this study, biological washout of 15O implanted by radioactive beams in brain and thigh (muscle and bony region) of rabbits was studied and that mechanism was investigated. Measurement was performed by the Open PET prototype which enables three-dimensional (3D) in-beam imaging. The regions of interest (ROIs) were selected based on the hot region, and time activity curves (TACs) were generated. Figure 1 shows TACs of each irradiation data with fit curves. TAC of both brain and thigh were clearly explained by the multiple-component model analysis. The each component ratios (and biological decay constant) were 57% (0.72 min-1) and 43% (0.024 min-1) for brain, 53% (0.344 min-1) and 46% (0.040 min-1) for thigh muscle and almost 100% (0.054 min-1) for bony region in thigh. The biological washout for brain was faster, given brain blood had higher perfusion. Furthermore, the observed positron-emitter distribution in the whole body was concentrated in the regions that had high blood volume (Figure 2). These results suggested that biological washout of 15O can be described as the clearance of 15O-labeled H2O by blood flows. To establish an accurate washout correction model, it is important not only correct assignment of tissue density and composition but also accounting for blood flows in each region.

### P 239: Multiple-CT Optimization: A Method to Account for Anatomical Changes in The Intensity-Modulated Proton Therapy for Nasopharyngeal Cancers

#### Z. Yang^1^, X. Zhang^1^, X. Zhu^1^, Y. Li^1^, H. Li^1^

##### ^1^The University of Texas MD Anderson Cancer Center, Radiation Physics, Houston, USA

**Purpose**: We aimed to determine whether multiple-CT (MCT) optimization of intensity-modulated proton therapy (IMPT) can improve plan robustness to anatomical changes.

**Methods and Materials**: Seven patients with nasopharyngeal cancer who underwent IMPT were included in this retrospective study. Each patient had primary planning CT (PCT), adaptive planning CT 1 (ACT1), and adaptive planning CT 2 (ACT2). Selective robust IMPT plans were generated using each CT data. PCT and ACT1 data were also used in combination to optimize one plan (MCT plan). Dose distributions were re-calculated using four different (PCT, ACT1, ACT2, and MCT) plans on ACT2 data. The doses to the target and organs at risk were compared between optimization strategies.

**Results**: MCT plans for all patients met all target dose and organs-at-risk criteria for all three CTs. Target dose and organs-at-risk dose for PCT and ACT1 plans re-calculated on ACT2 were compromised, indicating the need for adaptive planning on ACT2 if PCT or ACT1 plans are used (Fig. 1 & 2). MCT plan re-calculated on ACT2 had lower chiasm, parotid, and larynx doses than did PCT, ACT1, or ACT2 plans re-calculated on ACT2.

**Conclusions**: MCT plan can improve plan robustness to anatomical change and reduce the number of adaption planning for nasopharyngeal cancers.

